# Chemoselective γ‐Oxidation of β,γ‐Unsaturated Amides with TEMPO

**DOI:** 10.1002/anie.202104023

**Published:** 2021-07-20

**Authors:** Sebastian Heindl, Margaux Riomet, Ján Matyasovsky, Miran Lemmerer, Nicolas Malzer, Nuno Maulide

**Affiliations:** ^1^ Institute of Organic Chemistry University of Vienna Währinger Strasse 38 1090 Vienna Austria

**Keywords:** amides, chemoselectivity, oxidation, radical reactions, regioselectivity

## Abstract

A chemoselective and robust protocol for the γ‐oxidation of β,γ‐unsaturated amides is reported. In this method, electrophilic amide activation, in a rare application to unsaturated amides, enables a regioselective reaction with TEMPO resulting in the title products. Radical cyclisation reactions and oxidation of the synthesised products highlight the synthetic utility of the products obtained.

## Graphical abstract

A direct γ‐oxidation of β,γ‐unsaturated amides is reported. This regioselective reaction with TEMPO proceeds under mild conditions, tolerant of other carbonyl functionality. A rare example of regioselective amide activation of unsaturated substrates; it results in products carrying oxyaminated substitution ripe for remarkably selective radical cyclisation reactions.

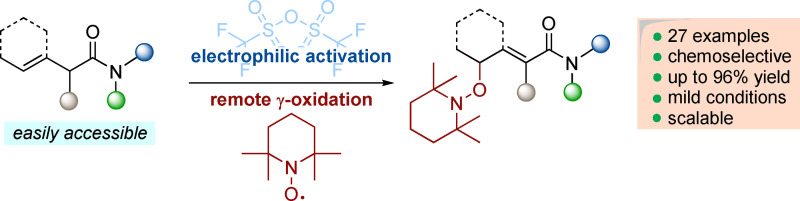

It is well‐documented that amides react only sluggishly with common nucleophiles, a fact typically ascribed to the electron‐releasing effect of the nitrogen center. Activation is therefore often necessary to promote carbonyl‐type reactivity in this family of compounds.^[1]^ After early, successful attempts at amide activation,^[2]^ trifluoromethanesulfonic anhydride (Tf_2_O) has eventually emerged as a general activating reagent following its introduction by Ghosez et al. in 1981.[^3^, ^4^, ^5^] This paved the way for further discoveries, ranging from Movassaghi's heterocycle synthesis to Huang's sequential reductive alkylation or Charette's chemoselective reduction methods.[^6^, ^7^, ^8^, ^9^] Our group has employed this activation mode, as a platform enabling ready access to highly reactive keteniminium ions, for the development of rearrangement‐driven transformations, including α‐arylation[^10^, ^11^] and α‐amination.^[12]^ In combination with *N*‐oxide reagents,^[13]^ a conceptually different Umpolung approach enabled the nucleophilic α‐incorporation of halides^[14]^ and other heteroatoms into amides,^[15]^ as well as the formation of lactams^[16]^ and 1,4‐dicarbonyls.^[17]^

In comparison to this plethora of methods for α‐functionalisation,[^10^, ^11^, ^12^, ^13^, ^14^, ^15^, ^16^, ^17^, ^18^] accessing remote positions has remained largely an unexplored area in amide activation. Two examples of γ‐aminoxylation were reported for conjugated acyloxazolidinone (imide) Ti‐enolates by Romea and Urpi (Scheme 1 a).^[19]^ A Se‐catalysed approach for the synthesis of γ‐alkoxy or γ‐hydroxy‐α,β‐unsaturated carbonyl compounds was developed by Tiecco, although only one amide example was reported and an excess of ammonium persulfate as oxidising agent was required, limiting functional group tolerance (Scheme 1 b).^[20]^ TEMPO addition to ketenes was previously reported, including one sole example of γ‐aminoxylation, albeit in low yield.^[21a]^ γ‐Hydroxylation of carbonyls, however not selective to amides, has also been achieved under copper catalysis.^[21b]^ A general method for direct, chemoselective γ‐oxidation of unsaturated amides has, however, not emerged yet. Our group has previously studied the interception of keteniminium ions in oxidative contexts beyond the use of *N*‐oxides, namely involving the persistent radical TEMPO.[^22^, ^23^] Herein, we present an approach to the chemoselective γ‐oxidation of unsaturated amides—to the best of our knowledge, a rare instance of electrophilic amide activation as applied to unsaturated substrates—as well as the intriguing reactivity that is unlocked when this reactivity manifold is leveraged by single‐electron processes.

**Scheme 1:**
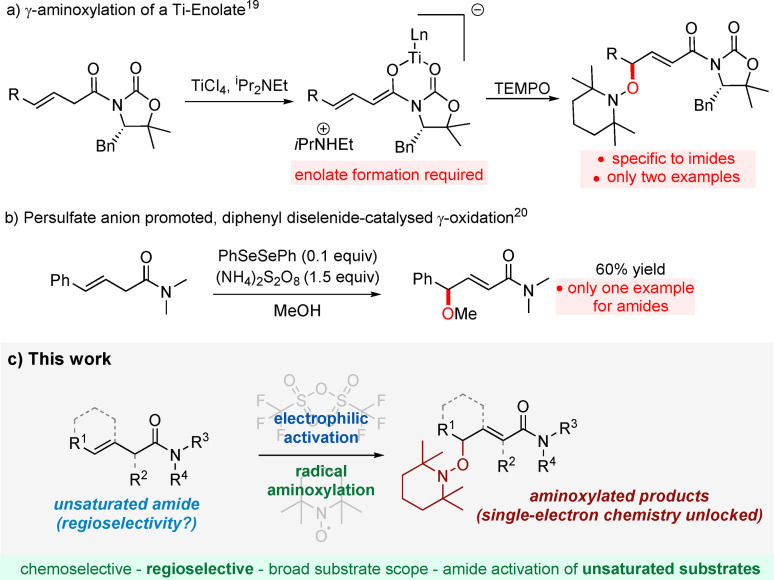
a,b) Strategies for γ‐oxidation of β,γ‐unsaturated amides. c) Proposed approach and the challenge of using unsaturated amides in electrophilic activation.

We focused our attention on model β,γ‐unsaturated amide **1 a**. Even though such substrates are rare features in the context of electrophilic amide activation, from the outset selective γ‐oxidation with concomitant double‐bond migration dominated the reactivity panorama. Further optimisation (see Supporting Information for details) showed that slightly more than two equivalents of TEMPO are required for efficient conversion. Equally relevant appears to be the choice of workup, with saturated aqueous NaHCO_3_ being optimal and enabling the isolation of product **2 a** in 96 % yield. It should be noted that substrate **1 a** is the readily available product of a simple deconjugative Knoevenagel condensation and subsequent amide formation (Scheme 2, see Supporting Information for details).

**Scheme 2:**
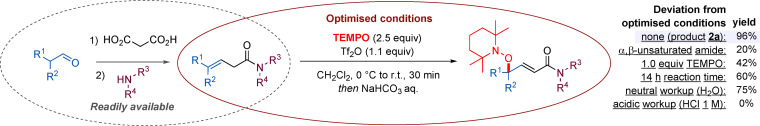
Optimised γ‐aminoxylation of unsaturated amides.

With optimised reaction conditions in hand, we explored the scope of this transformation, first evaluating possible substitution patterns in the carbon chain of the substrate (namely around the olefin, Scheme 3). Several alkyl substituents were tolerated at the terminal position, affording the desired products (**2 a**, **2 b**, **2 c**) in good to excellent yields. A terminal olefin yields the corresponding product **2 d** in 63 % yield. We were pleased to find that a β‐allene was a competent substrate in this transformation and the α,β‐γ,δ‐unsaturated amide **2 e** could be obtained in 55 % yield. Amides bearing a second β‐substituent could also be used (**2 h**), though an α‐methyl group led to sluggish reactivity (**2 f**).

**Scheme 3:**
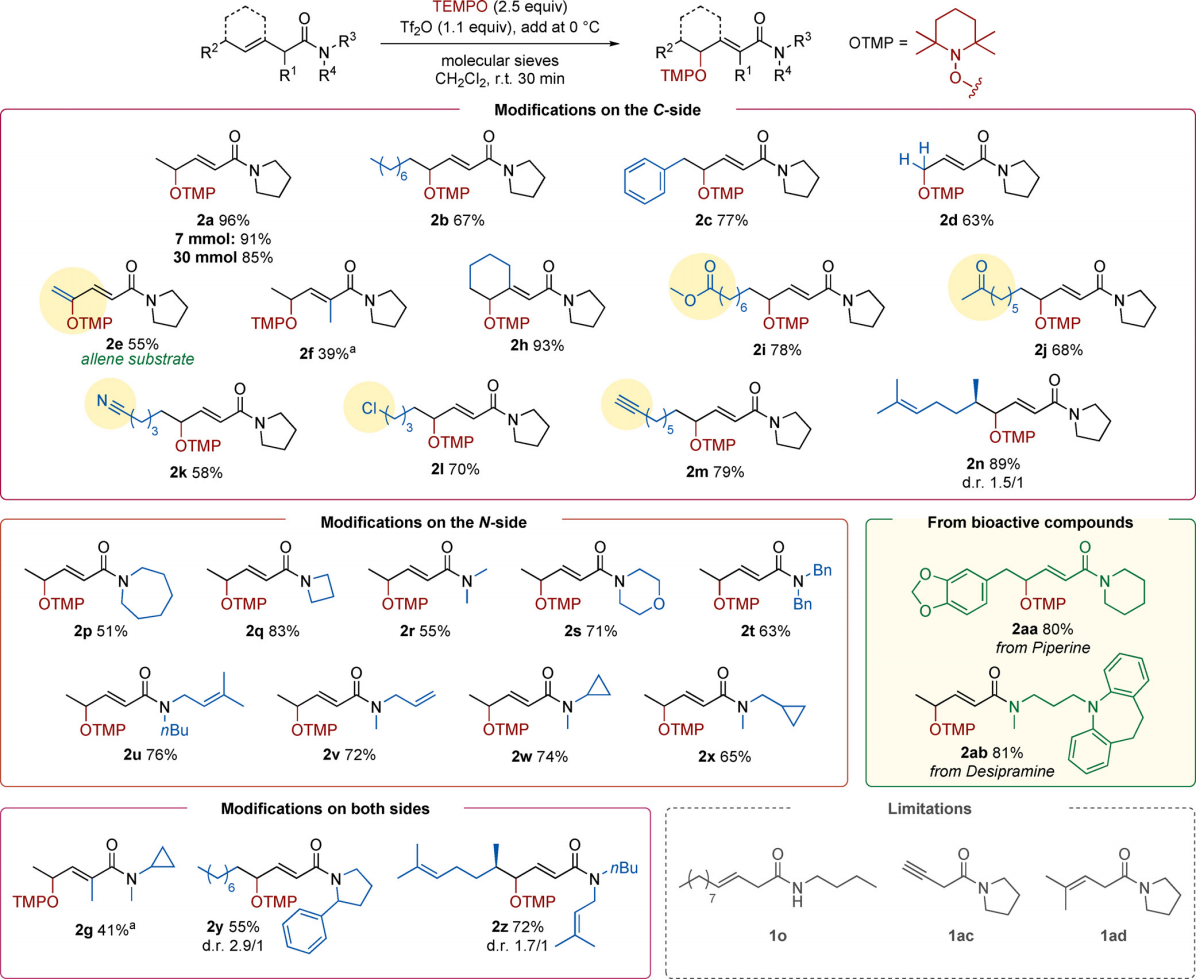
Scope of the γ‐oxidation of unsaturated amides. [a] The reaction was conducted at 40 °C for 4 hours.

Additionally, the reaction showed very good functional group tolerance. As is a common feature in electrophilic amide activation, other carbonyl functionalities such as an ester (**2 i**), a ketone (**2 j**), or a nitrile (**2 k**) were unaffected as well as a halide (**2 l**). Additional unsaturated moieties (**2 m** and **2 n**) remained untouched under the reaction conditions. We then investigated different substituents on the amide nitrogen. Symmetrical (**2 p**, **2 q**, **2 r**, **2 s**, **2 t**) as well as unsymmetrical amides (**2 u**, **2 v**, **2 w**, **2 x**) were well tolerated. In the latter cases, no cyclisation products, potentially originating from radical addition to an olefin/cyclopropane opening could be detected. More elaborate substrates could also be γ‐oxidised with ease (**2 g**, **2 y**, **2 z**). The reaction also proved scalable: when one gram of **1 a** was employed, the product was obtained without erosion of the yield, establishing the robustness of this method. Similar results were obtained with a 100‐fold scale increase. Neither a secondary amide (**1 o**) nor an alkyne (**1 ac**) were competent substrates, both reactions led to decomposition. The use of γ,γ‐disubstituted precursor **1 ad** resulted in no reaction.

At this juncture, we turned our attention to exploring the reactivity of the newly prepared aminoxyl‐amides (Scheme 4). While treatment of **2 a** with *m*CPBA afforded the ketone **3 a** in 78 % yield as a single (*E*)‐isomer, formation of the corresponding (*Z*)‐isomer (albeit in modest yield) was observed when **2 a** was irradiated under O_2_ atmosphere (Scheme 4 a). Such 1,4‐dicarbonyls have been reported to possess antimicrobial properties, and **3 c** is one specific example (**3 d** is a derivative thereof).^[24]^

**Scheme 4:**
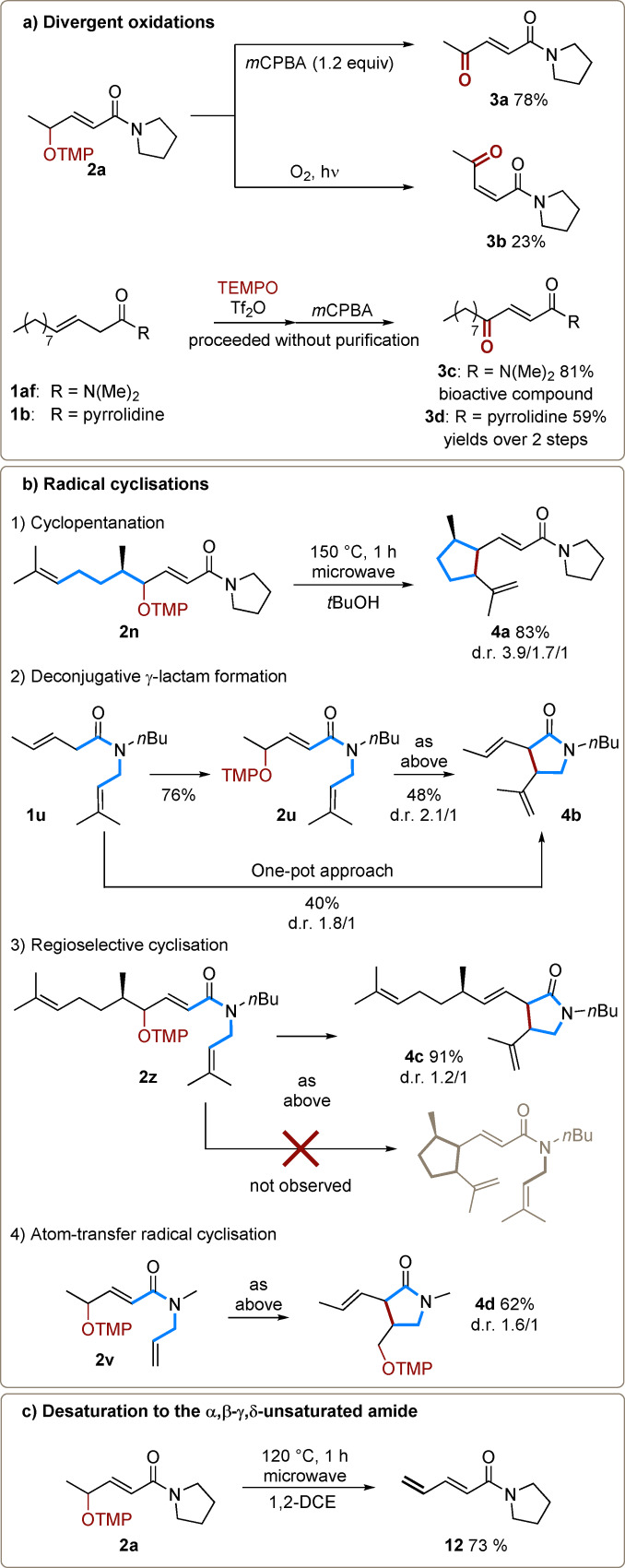
a) Oxidation and synthesis of a bioactive compound out of a γ‐OTMP α,β‐unsaturated amide. b) Thermal 5‐*exo*‐*trig* radical cyclisations of γ‐OTMP α,β‐unsaturated amides. c) Desaturation of the γ‐OTMP α,β‐unsaturated amide.

Furthermore, we hypothesised that compounds **2** might be amenable to C−O homolytic cleavage to generate delocalised allylic radical intermediates.^[25]^ In that regard, isoprenyl‐bearing amides appeared as the ideal substrates to explore the possible capture of a thermally generated radical through cyclisation (Scheme 4). Pleasingly, thermolysis of compound **2 n** at 150 °C under microwave irradiation cleanly delivered product **4 a** in 83 % yield (Scheme 4 b‐1). Interestingly, when compound **2 u**, bearing the isoprenyl substituent on the amide nitrogen, was employed, cyclisation occurred at the α‐position, affording the γ‐lactam **4 b** (Scheme 4 b‐2). This compound could also be accessed starting from amide **1 u** in a one‐pot‐two‐step process with an enhancement of the overall yield. Noteworthy, these products appear to arise from 5‐*exo*‐*trig* cyclisation followed by an oxidative elimination, rather than atom‐transfer. A subsequent competition experiment revealed that γ‐lactam formation outcompetes cyclisation onto the side‐chain, providing **4 c** as the sole product (Scheme 4 b‐3). It is likely that a combination of proximity to the electron‐withdrawing carbonyl (rendering the radical more electrophilic) as well as the rigidity of the amide bond (increasing the availability of favourable conformations) are all beneficial factors in promoting these cyclisations. Intriguingly, when a monosubstituted olefin (**2 v**) was utilised as an acceptor, an OTMP‐transfer cyclisation product (**4 d**) was obtained in 62 % yield rather than the previously observed alkene (Scheme 4 b‐4).[^25a^, ^26^]

When no suitable radical acceptor is present, as in the case of simple substrates like **2 a** (Scheme 4 c), microwave thermolysis at 120 °C results in elimination, as shown by α,β‐γ,δ‐unsaturated amide **12** in 73 % yield.

Mechanistically, we assume that attack of TEMPO radical on the keteniminium intermediate **I**[^22^, ^23^] generates a radical species **II** which rapidly recombines with a second equivalent of TEMPO at the distal γ‐position to form intermediate **III**, suggesting a radical–radical cross‐coupling controlled by the persistent radical effect.^[27]^ Fragmentation thereof results in product **IV** and a ring‐contracted iminium ion **V**.^[23]^ It is noteworthy that the corresponding amine **VI** could be isolated after treatment of the crude reaction mixture with NaBH_4_, in support of this proposal (Scheme 5).

**Scheme 5:**
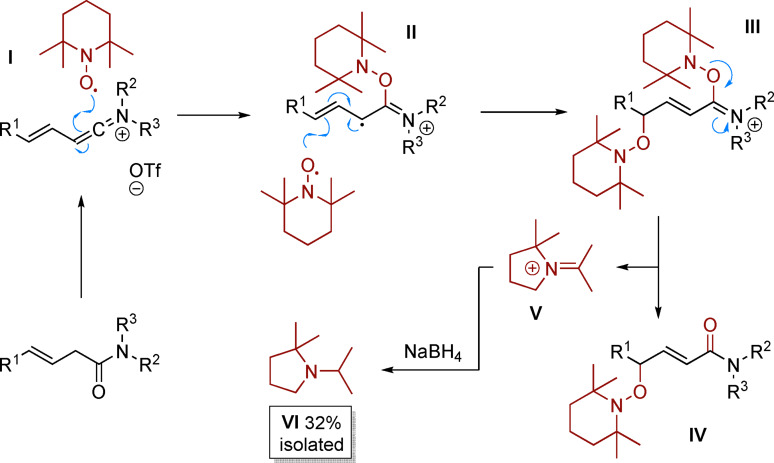
Proposed mechanism.

In summary, we developed a chemoselective method for remote γ‐oxidation of unsaturated amides through electrophilic activation under mild conditions. The obtained compounds open a large range of possible post‐functionalisations, including not only further oxidation but most intriguingly using the introduced aminoxyl functionality as a handle to trigger single‐electron ring‐forming processes. The intersection of electrophilic amide activation with the unique properties of one‐electron chemistry is bound to result in exciting avenues for further research.

## Conflict of interest

The authors declare no conflict of interest.

## Supporting information

As a service to our authors and readers, this journal provides supporting information supplied by the authors. Such materials are peer reviewed and may be re‐organized for online delivery, but are not copy‐edited or typeset. Technical support issues arising from supporting information (other than missing files) should be addressed to the authors.

Supporting Information
