# Supplementary material for: Chemoselective γ‐Oxidation of β,γ‐Unsaturated Amides with TEMPO
Source: Angew Chem Int Ed Engl. 2021 Jul 20;60(35):19123–7. doi: 10.1002/anie.202104023 (PMC8456850; doi:10.1002/anie.202104023)

Angewandte Chemie
Eine Zeitschrift der Gesellschaft Deutscher Chemiker

Supporting Information

Chemoselective γ-Oxidation of β,γ-Unsaturated Amides with TEMPO

*Sebastian Heindl, Margaux Riomet[+], Ján Matyasovsky[+], Miran Lemmerer[+], Nicolas Malzer, and Nuno Maulide**

anie_202104023_sm_miscellaneous_information.pdf

Table of Contents

1. General information ... 2
2. Experimental ... 2
 2.1 Optimization of reaction conditions ... 2
 2.2 Synthesis of starting materials ... 3
  2.2.1 General procedure A: Synthesis of aldehydes ... 3
  2.2.2 General procedure B: Synthesis of β,γ-unsaturated carboxylic acids[5] ... 3
  2.2.3 General procedure C: Amide coupling ... 6
  2.2.4 Additional reactions ... 16
 2.3 General procedure D: Synthesis of α,β-unsaturated γ-OTMP amides ... 17
 2.4 Mechanistic investigations ... 27
 2.5 Functionalization reactions ... 29
  2.5.1 Oxidation/Reduction ... 29
  2.5.2 Cyclizations ... 30
  2.5.3 Synthesis of a bioactive compound according to Omura *et al.*[13] ... 32
  2.5.4 Desaturation to the α,β-γ,δ-unsaturated amide ... 33
3. References ... 34
4. NMR-Spectras ... 35
 4.1 Amides and amines ... 35
 4.2 α,β-unsaturated-γ-OTMP amides ... 68
 4.3. Mechanistic investigations ... 95
 4.3 Post-functionalizations ... 96

1. General information

All reagents were used as received from commercial suppliers unless otherwise stated. Trifluoromethanesulfonic anhydride (Tf_2O) was distilled over P_4O_{10} prior to use and stored under inert atmosphere in the fridge for a maximum of roughly 3 weeks.[1] Reaction progress was monitored by thin layer chromatography (TLC) performed on aluminum plates coated with silica gel F254 with 0.2 mm thickness. Chromatograms were visualized by fluorescence quenching with UV light at 254 nm or by staining using potassium permanganate. Flash column chromatography was performed using silica gel 60 (230-400 mesh, Merck and co.) or pre-packed columns (15–40 µm, Macherey-Nagel) on an Isolera Four flash chromatography instrument (Biotage®). Neat infrared spectra were recorded using a Perkin-Elmer Spectrum 100 FT-IR spectrometer. Wavenumbers (ν_{max}) are reported in cm^{-1}. Mass spectra were obtained using a Finnigan MAT 8200 or (70 eV) or an Agilent 5973 (70 eV) spectrometer, using electrospray ionization (ESI). All ^1H NMR and ^{13}C NMR spectra were recorded using a Bruker AV-400 AV-600, or AV-700 spectrometer at 300K. Chemical shifts are given in parts per million (ppm, δ), referenced to the solvent peak of $CDCl_3$, defined at δ = 7.26 ppm (^1H-NMR) and δ = 77.16 (^{13}C-NMR). Coupling constants are quoted in Hz (J). ^1H NMR splitting patterns are designated as singlet (s), doublet (d), triplet (t), quartet (q) as they appeared in the spectrum. If the appearance of a signal differs from the expected splitting pattern, the observed pattern is designated as apparent (app). Splitting patterns that could not be interpreted or easily visualized are designated as multiplet (m) or broad (br). Reactions under microwave irradiation were performed using a CEM Discover SP microwave-reactor. Reaction under light irradiation was performed in a Rayonet apparatus RPR-100 equipped with 16 RPR-3000A lamps.

2. Experimental

2.1 Optimization of reaction conditions

All listed yields in Table 1 are determined after isolation. To a flame-dried Schlenk flask under an atmosphere of argon and containing molecular sieves, was added amide **1** (1.0 equiv.) and anhydrous solvent (0.1 M). TEMPO was then added and the reaction mixture was cooled in an ice-water bath. Trifluoromethanesulfonic anhydride (Tf_2O) was subsequently added, the cooling bath was removed, and the reaction was stirred at room temperature. Then the reaction mixture was filtered, removing the molecular sieves, and added to a flask containing an aqueous solution (2.5 ml/mmol). The mixture was vigorously stirred at room temperature for 15 min, before the phases were separated, and the aqueous phase was extracted with CH_2Cl_2 (3 × 30 ml/mmol). The combined organic phases were dried over anhydrous magnesium sulfate. The dried solution was filtered, and the filtrate was concentrated under reduced pressure. The crude residue was purified by flash column chromatography (SiO_2, heptane/ethyl acetate) to afford the product **2**.

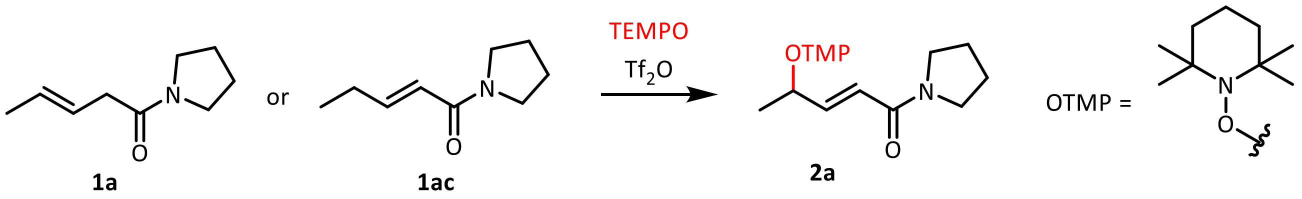

Entry	Substrate	Tf_2O (equiv.)	TEMPO (equiv.)	Temperature	Time	Additive	Workup solution	Solvent	Yield
1	**1a**	1.1	1.0	0 °C → r.t.	14 h	3 Å sieves	H_2O	CH_2Cl_2	37% (50% brsm)
2	**1a**	1.1	3.0	0 °C → r.t.	14 h	3 Å sieves	H_2O	CH_2Cl_2	60%
3	**1a**	1.1	5.0	0 °C → r.t.	14 h	3 Å sieves	H_2O	CH_2Cl_2	42%
4	**1a**	2.0	2.5	0 °C → r.t.	14 h	3 Å sieves	H_2O	CH_2Cl_2	47%
5	**1a**	1.1	2.2	0 °C → r.t.	14 h	3 Å sieves	H_2O	CH_2Cl_2	60%
6	**1a**	1.1	2.2	0 °C → r.t.	14 h	3 Å sieves	H_2O	Toluene	55%
7	**1a**	1.1	2.2	0 °C → r.t.	14 h	3 Å sieves	H_2O	Acetonitrile	55%
8	**1a**	1.1	2.5	0 °C	14 h	3 Å sieves	H_2O	CH_2Cl_2	61%[a]
9	**1a**	1.1	2.5	0 °C → r.t.	1 h	-	H_2O	CH_2Cl_2	65%
10	**1a**	1.1	2.5	0 °C → r.t.	30 min	-	H_2O	CH_2Cl_2	75%
11	**1a**	1.1	2.5	0 °C → r.t.	30 min	4 Å sieves	aq. sat. $NaHCO_3$	CH_2Cl_2	96%
12	**1a**	1.1	1	0 °C → r.t.	30 min	4 Å sieves	aq. sat. $NaHCO_3$	CH_2Cl_2	42%[a] (48% brsm)
13	**1a**	1.1	2.5	0 °C → r.t.	30 min	4 Å sieves	aq. sat. NH_4Cl	CH_2Cl_2	31%
14	**1a**	1.1	2.5	0 °C → r.t.	30 min	4 Å sieves	aq. NaOH (1 M)	CH_2Cl_2	78%
15	**1a**	1.1	2.5	0 °C → r.t.	30 min	4 Å sieves	aq. HCl (1 M)	CH_2Cl_2	0%
16	**1ae**	1.1	2.5	0 °C → r.t.	30 min	4 Å sieves	aq. sat. $NaHCO_3$	CH_2Cl_2	20% (54% brsm)

Table 1: Optimization of reaction conditions: [a]*The reaction was cooled in an ice-water bath throughout the whole reaction time.* [b]*Premixing the amide and Tf_2O.*

2.2 Synthesis of starting materials

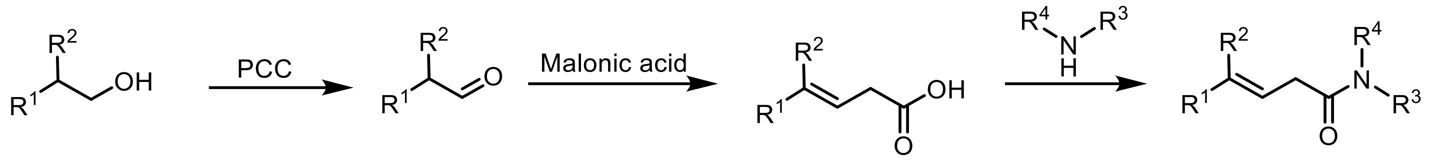

2.2.1 General procedure A: Synthesis of aldehydes

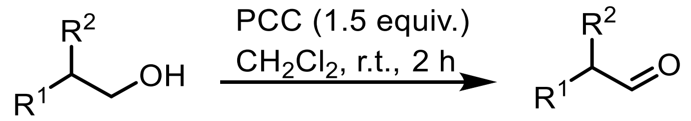

To a solution of pyridinium chlorochromate (PCC, 1.5 equiv.) in CH_2Cl_2 (0.36 M), alcohol (1.0 equiv.) was added slowly. The reaction mixture was stirred at room temperature for 2 h, then the resulting solution/suspension was filtered through a pad of silica gel, eluting with diethyl ether. The solvent was removed under reduced pressure, and the remaining crude residue was purified by flash column chromatography (SiO_2, heptane/ethyl acetate) to afford the compounds **5**.

6-Chlorohexanal (5l)

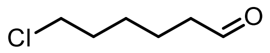

Following general procedure A using 6-chlorohexan-1-ol (3.30 ml; 25.0 mmol; 1.0 equiv.) and PCC (8.08 g; 37.5 mmol; 1.5 equiv.). Purification by flash column chromatography (heptane/ethyl acetate) gave the title compound (2.26 g, 67%) as a yellow oil.

^{1}H NMR (400 MHz, $CDCl_3$): δ 9.77 (s, 1H), 3.53 (t, *J* = 7.0 Hz, 2H), 2.46 (t, *J* = 7.5 Hz, 2H), 1.84–1.74 (m, 2H), 1.70–1.61 (m, 2H), 1.53–1.42 (m, 2H) ppm.

All analytical data were in good accordance with data reported in the literature.[2]

Dec-9-ynal (5m)

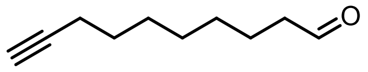

Following general procedure A using 9-decyn-1-ol (2.1 ml; 12.0 mmol; 1.0 equiv.) and PCC (3.88 g; 18.0 mmol; 1.5 equiv.). Purification by flash column chromatography (heptane/ethyl acetate) gave the title compound (1.26 g, 69%) as a yellow oil.

^{1}H NMR (400 MHz, $CDCl_3$): δ 9.75 (s, 1H), 2.41 (dt, *J* = 7.3, 1.6 Hz, 2H), 2.16 (dt, *J* = 7.0, 2.5 Hz, 2H), 1.92 (t, *J* = 2.5 Hz, 1H), 1.67–1.57 (m, 2H), 1.55–1.46 (m, 2H), 1.44–1.25 (m, 6H) ppm.

All analytical data were in good accordance with data reported in the literature.[3]

(*R*)-3,7-Dimethyloct-6-enal (5n)

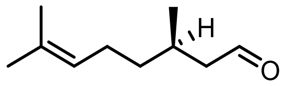

Following general procedure A using citronellol (1.82 ml; 10.0 mmol; 1.0 equiv.) and PCC (3.23 g; 15.0 mmol; 1.5 equiv.). Purification by flash column chromatography (heptane/ethyl acetate) gave the title compound (1.42 g, 92%) as a colourless oil.

^{1}H NMR (400 MHz, $CDCl_3$): δ 9.75 (s, 1H), 5.08 (t, *J* = 7.3 Hz, 1H), 2.40 (dd, *J* = 15.9, 5.9 Hz, 1H), 2.22 (dd, *J* = 16.3, 8.0 Hz, 1H), 2.13–1.92 (m, 3H), 1.68 (s, 3H), 1.60 (s, 3H), 1.41–1.21 (m, 2H), 0.97 (d, *J* = 6.7 Hz, 3H) ppm.

All analytical data were in good accordance with data reported in the literature.[4]

2.2.2 General procedure B: Synthesis of β,γ-unsaturated carboxylic acids[5]

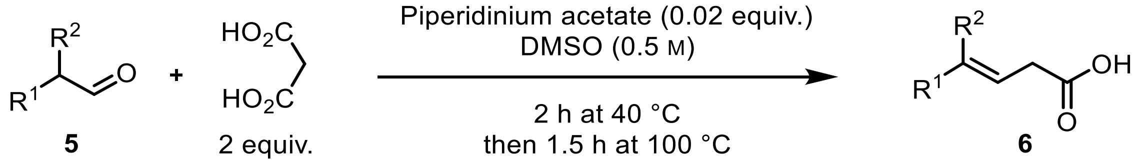

Aldehyde **5** (1.0 equiv.), malonic acid (2.0 equiv.) and piperidinium acetate **7** (0.02 equiv.) were dissolved in DMSO (0.5 M). The reaction mixture was subsequently warmed to 40 °C and stirred at that temperature for 2 hours. After this time, the resulting solution was heated to 100 °C with continued stirring, to ensure decarboxylation. After 1.5 h, CO_2-evolution ceased. The reaction mixture was cooled to r.t. then pouring into water (5 ml/mmol) cooled in an ice-water bath. The resulting biphasic mixture was separated, and the aqueous phase was extracted with diethyl ether (3 × 20 ml/mmol). The combined organic phases were washed with water and brine, then dried over anhydrous magnesium sulfate. The dried solution was filtered, and the filtrate was concentrated under reduced pressure. Synthesis of the amides **1** was conducted using the crude material of β,γ-unsaturated acids **6**.

(*E*)-Dodec-3-enoic acid (6b)

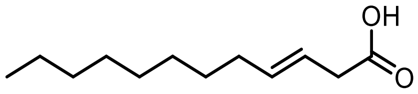

Following general procedure B using decanal (2.0 ml; 10.0 mmol; 1.0 equiv.), malonic acid (2.08 g; 20.0 mmol; 2.0 equiv.) and piperidinium acetate **7** (29.0 mg; 0.2 mmol; 0.02 equiv.). The crude material was used directly in the next step.

(*E*)-5-Phenylpent-3-enoic acid (6c)

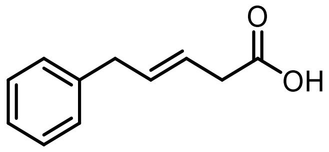

Following general procedure B using 3-phenylpropanal (1.40 ml; 10.0 mmol; 1.0 equiv.), malonic acid (2.08 g; 20.0 mmol; 2.0 equiv.) and piperidinium acetate **7** (29 mg; 0.20 mmol; 0.02 equiv.). The crude material was used directly in the next step.

Penta-3,4-dienoic acid[6] (6e)

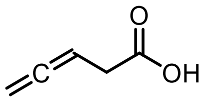

Ethyl penta-3,4-dienoate **11** (315.0 mg; 2.5 mmol; 1.0 equiv.) was dissolved in acetone (1 M; 2.5 ml) and aq. HCl (20%; 3.8 ml) was added. After stirring for 27 hours at room temperature the biphasic mixture was separated, and the aqueous phase was extracted with Et_2O (3 × 10 ml). The combined organic phases were dried over anhydrous magnesium sulfate. The dried solution was filtered, and the filtrate was concentrated under reduced pressure. The crude material was used directly in the next step.

(*E*)-2-Methylpent-3-enoic acid[7] (6f)

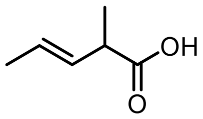

To a flame-dried flask under an argon atmosphere was added diisopropylamine (1.01 g; 10.0 mmol; 2.0 equiv.) and tetrahydrofuran (37 ml). The resulting solution was cooled with a ice-salt bath, and treated with a solution of *n*-BuLi (1.6 M in hexane; 6.25 ml; 10.0 mmol; 2.0 equiv.). After 15 minutes of stirring hexamethylphosphoramide (2.1 ml; 12.0 mmol; 2.4 equiv.) was dropwise added at –78 °C. The mixture was stirred at that temperature for 1 hour. (*E*)-3-pentenoic acid (0.51 ml; 5.0 mmol; 1.0 equiv.) dissolved in tetrahydrofuran (10 mL) was added over 30 minutes with a syringe pump. After complete addition, the solution was stirred for 1 hour before iodomethane (0.31 ml; 5.0 mmol; 1.0 equiv.) was rapidly added at –78 °C. After 1 hour the reaction was diluted with tetrahydrofuran and stopped with aqueous HCl (1.6 M; 100 ml). The biphasic mixture was then separated, and the aqueous phase was extracted with Et_2O (3 × 30 ml). The combined organic phases were washed with HCl (5%, 3 × 20 ml), water (1 × 20 ml) and brine (1 × 20 ml). The combined organic phases were dried over anhydrous magnesium sulfate. The dried solution was filtered, and the filtrate was concentrated under reduced pressure. The crude residue was purified by flash column chromatography (SiO_2, heptane/ethyl acetate/acetic acid) to afford the title compound (0.30 g, 53%).

^{1}H NMR (400 MHz, $CDCl_3$): δ 11.85 (s, 1H), 5.67–5.46 (m, 2H), 3.17–3.67 (m, 1H), 1.69 (d, *J* = 6.2 Hz, 3H), 1.26 (d, *J* = 7.1 Hz, 3H) ppm.

All analytical data were in good accordance with data reported in the literature.[7]

2-(Cyclohex-1-en-1-yl)-acetic acid (6h)

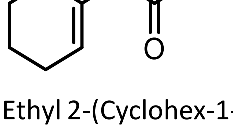

Ethyl 2-(Cyclohex-1-en-1-yl) acetate **10** (1.85 g; 11.0 mmol; 1.0 equiv.) was dissolved in ethanol (2 M) and aqueous 1 M NaOH (15.4 ml) was added. The reaction mixture was stirred at room temperature for 24 hours, then it was adjusted to a pH-value of 6 with conc. HCl. The biphasic mixture was separated, and the aqueous phase was extracted with ethyl acetate (3 × 10 ml). The combined organic phases were dried over anhydrous magnesium sulfate. The dried solution was filtered, and the filtrate was concentrated under reduced pressure. The crude material was used directly in the next step.

(*E*)-12-Methoxy-12-oxododec-3-enoic acid (6i)

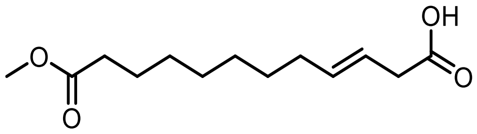

Following general procedure B using methyl 9-formylnonanoate (0.52 ml; 2.5 mmol; 1.0 equiv.), malonic acid (0.52 g; 5.0 mmol; 2.0 equiv.) and piperidinium acetate **7** (8 mg; 0.05 mmol; 0.02 equiv.). The crude material was used directly in the next step.

(*E*)-8-Chlorooct-3-enoic acid (6l)

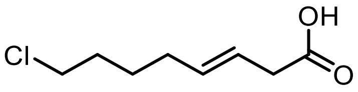

Following general procedure B using 6-chlorohexanal **5l** (2.02 g; 15.0 mmol; 1.0 equiv.), malonic acid (3.12 g; 30.0 mmol; 2.0 equiv.) and piperidinium acetate **7** (43.6 mg; 0.3 mmol; 0.02 equiv.). The crude material was used directly in the next step.

(*E*)-Dodec-3-en-11-ynoic acid (6m)

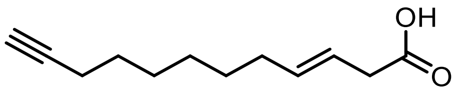

Following general procedure B using dec-9-ynal **5l** (0.91 g; 6.0 mmol; 1.0 equiv.), malonic acid (1.25 g; 12.0 mmol; 2.0 equiv.) and piperidinium acetate **7** (17.4 mg; 0.12 mmol; 0.02 equiv.). The crude material was used directly in the next step.

(*R*,*E*)-5,9-Dimethyldeca-3,8-dienoic acid (6n)

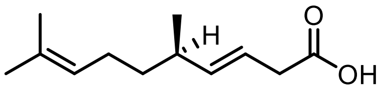

Following general procedure B using (*R*)-3,7-dimethyloct-6-enal **5m** (1.23 g; 8.0 mmol; 1.0 equiv.), malonic acid (1.67 g; 16.0 mmol; 2.0 equiv.) and piperidinium acetate **7** (23.0 mg; 0.16 mmol; 0.02 equiv.). The crude material was used directly in the next step.

4-Methyl-2-pentenoic acid (6u)

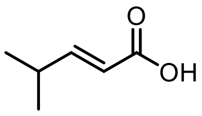

Following general procedure B using isobutyraldehyde (0.91 ml; 10.0 mmol; 1.0 equiv.), malonic acid (2.08 g; 20.0 mmol; 2.0 equiv.) and piperidinium acetate **7** (29.0 mg; 0.2 mmol; 0.02 equiv.). The crude material was used directly in the next step (for this substrate the α,β-unsaturated product was obtained).

4-Methylpent-3-enoic acid (6ab), mixture with 4-methyl-2-pentenoic acid (6u)

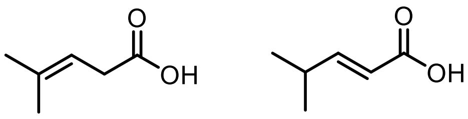

88 : 12

6ab **6u**

4-Methyl-2-pentenoic acid **6u** (92.0 mg; 0.81 mmol; 1.0 equiv.) was dissolved in an aq. KOH-solution (6 M; 1 ml) and heated at 105 °C for 48 h. After cooling to room temperature, the reaction mixture was acidified to pH 1 with aq. HCl (1 M). The mixture was extracted with CH_2Cl_2, (3 × 5 ml) and the combined organic phases were dried over anhydrous magnesium sulfate. The dried solution was filtered, and the filtrate was concentrated under reduced pressure to afford a crude mixture of alkene **6ab** and **6u** (88:12), used directly in the next step.

2.2.3 General procedure C: Amide coupling

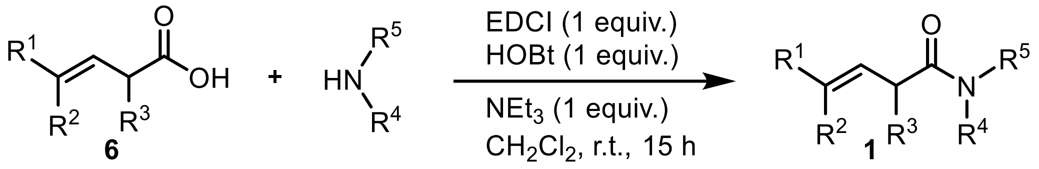

Carboxylic acid **6** (1.0 equiv.) was dissolved in CH_2Cl_2 (0.2 M), after which the corresponding amine (1.0 equiv.), triethylamine (1.0 equiv.), 1-hydroxybenzotriazole hydrate (HOBt, 1.0 equiv.) and 1-(3-dimethylaminopropyl)-3-ethylcarbodiimide hydrochloride (EDCI, 1.0 equiv.) were added and the reaction mixture was stirred for 15 h at room temperature. After this time, ethyl acetate (7.0 ml/mmol) and aq. HCl (1 M; 3.6 ml/mmol) were added, the resulting biphasic mixture was separated, and the organic phase was sequentially washed with a saturated aqueous solution of $NaHCO_3$ (1 × 5 ml/mmol)and brine (1 × 5 ml/mmol). The washed solution was then dried over anhydrous magnesium sulfate, filtered, and the filtrate was concentrated under reduced pressure. The crude residue was purified by flash column chromatography (SiO_2, heptane/ethyl acetate) to afford the compounds **1**.

(*E*)-1-(Pyrrolidin-1-yl)-pent-3-en-1-one (1a)

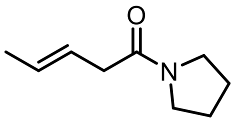

Following general procedure C using (*E*)-3-pentenoic acid (1.02 ml; 10.0 mmol; 1.0 equiv.), pyrrolidine (0.82 ml; 10.0 mmol; 1.0 equiv.), triethylamine (1.39 ml; 10.0 mmol; 1.0 equiv.), 1-hydroxybenzotriazole hydrate (1.35 g; 10.0 mmol; 1.0 equiv.) and 1-(3-dimethylaminopropyl)-3-ethylcarbodiimide hydrochloride (1.92 g; 10.0 mmol; 1.0 equiv.). Purification by flash column chromatography (heptane/ethyl acetate 20-100%) gave the title compound (1.43 g, 93%) as a slightly yellow oil.

^{1}H NMR (400 MHz, CDCl$_3$): δ 5.61–5.44 (m, 2H), 3.40 (dt, *J* = 11.5, 6.8 Hz, 4H), 2.97 (d, *J* = 4.8 Hz, 2H), 1.96-1.85 (m, 2H) 1.85-1.76 (m, 2H), 1.66 (d, *J* = 4.5 Hz, 3H) ppm.

^{13}C NMR (150 MHz, CDCl$_3$): δ 170.1, 128.5, 124.0, 46.7, 45.8, 39.2, 26.2, 24.5, 18.1 ppm.

HRMS (ESI+): Calculated for $[M+Na]^+$ $C_9H_{15}ONNa^+$: 176.1046, found: 176.1048.

IR (cm^{-1}): 2972, 2872, 1627, 1425, 1340, 1169, 1105, 1078, 1064, 1038, 965, 722, 684, 599.70.

(*E*)-1-(Pyrrolidin-1-yl)-tridec-3-en-1-one (1b)

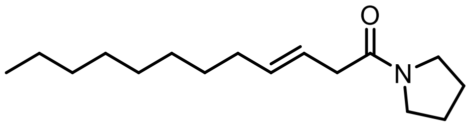

Following general procedure C using (*E*)-dodec-3-enoic acid **6b** (0.99 g; 5.0 mmol; 1.0 equiv.), pyrrolidine (0.41 ml; 5.0 mmol; 1.0 equiv.), triethylamine (0.70 ml; 5.0 mmol; 1.0 equiv.), 1-hydroxybenzotriazole hydrate (0.68 g; 5.0 mmol; 1.0 equiv.) and 1-(3-dimethylaminopropyl)-3-ethylcarbodiimide hydrochloride (0.96 g; 5.0 mmol; 1.0 equiv.). Purification by flash column chromatography (heptane/ethyl acetate 20-100%) gave the title compound (0.53 g; 42% over 2 steps) as a slightly yellow oil.

^{1}H NMR (400 MHz, CDCl$_3$): δ 5.61–5.46 (m, 2H), 3.49–3.37 (m, 4H), 3.04–2.98 (br s, 2H), 2.02 (app q, *J* = 6.1 Hz, 2H), 1.98–1.88 (m, 2H), 1.87–1.78 (m, 2H), 1.32–1.18 (m, 12H), 0.87 (t, *J* = 6.7 Hz, 3H) ppm.

^{13}C NMR (150 MHz, CDCl$_3$): δ 170.2, 134.2, 122.7, 46.7, 45.8, 39.4, 32.7, 32.0, 29.6, 29.4 (2C), 29.3, 26.3, 24.5, 22.8, 14.2 ppm.

HRMS (ESI+): Calculated for $[M+H]^+$ $C_{16}H_{29}ONH^+$: 252.2322, found: 252.2320.

IR (cm^{-1}): 2924, 2852, 2163, 1641, 1422, 1341, 1253, 1226, 1193, 1115, 1070, 1036, 967, 914, 859, 722.

(*E*)-5-Phenyl-1-(pyrrolidin-1-yl)-pent-3-en-1-one (1c)

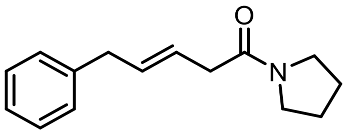

Following general procedure C using (*E*)-5-phenylpent-3-enoic acid **6c** (0.88 g; 5.0 mmol; 1.0 equiv.), pyrrolidine (0.41 ml; 5.0 mmol; 1.0 equiv.), triethylamine (0.70 ml; 5.0 mmol; 1.0 equiv.), 1-hydroxybenzotriazole hydrate (0.68 g; 5.0 mmol; 1.0 equiv.) and 1-(3-dimethylaminopropyl)-3-ethylcarbodiimide hydrochloride (0.96 g; 5.0 mmol; 1.0 equiv.). Purification by flash column chromatography (heptane/ethyl acetate 20-100%) gave the title compound (0.73 g; 64% over 2 steps) as a slightly yellow oil.

^{1}H NMR (400 MHz, CDCl$_3$): δ 7.31–7.25 (m, 2H), 7.21–7.15 (m, 3H), 5.75–5.63 (m, 2H), 3.47 (t, *J* = 6.9 Hz, 2H), 3.43–3.36 (m, 4H), 3.06 (d, *J* = 4.3 Hz, 2H), 1.97–1.78 (m, 4H) ppm.

^{13}C NMR (150 MHz, CDCl$_3$): δ 169.9, 140.5, 132.4, 128.6 (2C), 128.5 (2C), 126.1, 124.5, 46.7, 45.8, 39.11, 39.06, 26.2, 24.5 ppm.

HRMS (ESI+): Calculated for $[M+Na]^+$ $C_{15}H_{19}ONNa^+$: 252.1359, found: 252.1362.

IR (cm^{-1}): 3025, 2872, 1634, 1494, 1423, 1341, 1226, 1192, 1080, 1029, 967, 931, 914, 859, 748, 698.

1-(Pyrrolidin-1-yl)but-3-en-1-one (1d)

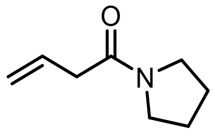

Following general procedure C using 3-butenoic acid (0.43 ml; 5.0 mmol; 1.0 equiv.), pyrrolidine (0.41 ml; 5.0 mmol; 1.0 equiv.), triethylamine (0.70 ml; 5.0 mmol; 1.0 equiv.), 1-hydroxybenzotriazole hydrate (0.68 g; 5.0 mmol; 1.0 equiv.) and 1-(3-dimethylaminopropyl)-3-ethylcarbodiimide hydrochloride (0.96 g; 5.0 mmol; 1.0 equiv.). Purification by flash column chromatography (heptane/ethyl acetate 20-100%) gave the title compound (0.47 g; 68% as a colourless oil.

^{1}H NMR (400 MHz, $CDCl_3$): δ 6.04–5.89 (m, 1H), 5.20–5.07 (m, 2H), 3.50–3.40 (m, 4H), 3.08 (d, *J* = 6.7 Hz, 2H), 1.99–1.80 (m, 4H) ppm.

^{13}C NMR (100 MHz, $CDCl_3$): δ 169.5, 131.7, 117.8, 46.8, 45.9, 40.4, 26.3, 24.5 ppm.

HRMS (ESI+): Calculated for $[M+Na]^+$ $C_8H_{13}ONNa^+$: 162.0889, found 162.0885.

IR (cm^{-1}): 3523, 2973, 2874, 1623, 1435, 993, 911.

1-(Pyrrolidin-1-yl)-penta-3,4-dien-1-one (1e)

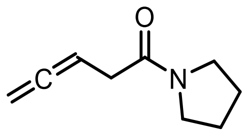

Following general procedure C using penta-3,4-dienoic acid **6e** (0.29 g; 3.0 mmol; 1.0 equiv.), pyrrolidine (0.25 ml; 3.0 mmol; 1.0 equiv.), triethylamine (0.42 ml; 3.0 mmol; 1.0 equiv.), 1-hydroxybenzotriazole hydrate (0.41 g; 3.0 mmol; 1.0 equiv.) and 1-(3-dimethylaminopropyl)-3-ethylcarbodiimide hydrochloride (0.58 g; 3.0 mmol; 1.0 equiv.). Purification by flash column chromatography (heptane/ethyl acetate 20-100%) gave the title compound (0.20 g; 45% over 3 steps) as a yellow oil.

^{1}H NMR (400 MHz, $CDCl_3$: δ 5.39–5.31 (m, 1H); 4.73 (dt, *J* = 6.7, 3.0 Hz, 2H); 3.45 (dt, *J* = 12.7, 6.0 Hz, 4H); 3.03 (dt, *J* = 7.2, 3.0 Hz, 2H), 1.99–1.90 (m, 2H), 1.89–1.80 (m, 2H) ppm.

^{13}C NMR (150 MHz, $CDCl_3$): δ 209.3, 169.2, 84.5, 75.5, 46.7, 45.9, 35.3, 26.3, 24.5 ppm.

HRMS (ESI+): Calculated for $[M+Na]^+$ $C_9H_{13}ONNa^+$: 174.0889, found: 174.0901.

IR (cm^{-1}): 2969, 2869, 1957, 1635, 1426, 1340, 1227, 1189, 1169, 1117, 914, 849.

All analytical data were in good accordance with data reported in the literature.[8]

(*E*)-2-Methyl-1-(pyrrolidin-1-yl)-pent-3-en-1-one (1f)

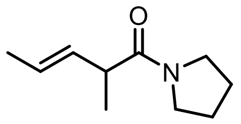

Following general procedure C using (*E*)-2-Methylpent-3-enoic acid **6f** (0.23 g; 2.0 mmol; 1.0 equiv.), pyrrolidine (0.16 ml; 2.0 mmol; 1.0 equiv.), triethylamine (0.28 ml; 2.0 mmol; 1.0 equiv.), 1-hydroxybenzotriazole hydrate (0.27 g; 2.0 mmol; 1.0 equiv.) and 1-(3-dimethylaminopropyl)-3-ethylcarbodiimide hydrochloride (0.38 g; 2.0 mmol; 1.0 equiv.). Purification by flash column chromatography (heptane/ethyl acetate 20-100%) gave the title compound (0.28 g; 84%) as a slightly yellow oil.

^{1}H NMR (400 MHz, $CDCl_3$): δ 5.53–5.41 (m, 2H), 3.52–3.35 (m, 4H), 3.19–3.09 (m, 1H), 1.96–1.76 (m, 4H), 1.64 (t, *J* = 4.9 Hz, 3H), 1.17 (t, *J* = 6.7 Hz, 3H) ppm.

^{13}C NMR (150 MHz, $CDCl_3$): δ 173.1, 131.3, 126.0, 46.3, 45.9, 42.1, 26.2, 24.3, 18.0 (2C) ppm.

HRMS (ESI+): Calculated for $[M+Na]^+$ $C_{10}H_{17}ONNa^+$: 190.1202, found: 190.1206

IR (cm^{-1}): 2968, 2872, 1633, 1455, 1339, 1253, 1225, 1191, 1170, 1116, 1097, 1040, 966, 915, 854, 765.

(*E*)-*N*-cyclopropyl-*N*,2-dimethylpent-3-enamide (1g)

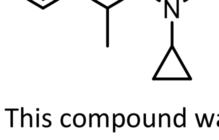

This compound was obtained as a by-product of **1w** (see below) as a colourless oil (84.0 mg, 25% over 2 steps).

^{1}H NMR (600 MHz, $CDCl_3$) δ 5.48–5.37 (m, 2H), 3.87–3.69 (m, 1H), 2.84 (s, 3H), 2.69–2.59 (m, 1H), 1.59 (d, *J* = 4.4 Hz, 3H), 1.12 (d, *J* = 6.8 Hz, 3H), 0.86–0.77 (m, 2H), 0.75–0.70 (m, 1H), 0.67–0.62 (m, 1H) ppm.

^{13}C NMR (151 MHz, $CDCl_3$) δ 177.6, 131.8, 125.7, 40.4, 34.2, 31.0, 18.4, 17.9, 9.6, 8.7 ppm.

HRMS (ESI) (m/z): calculated for $[M+Na]^+$ $C_{10}H_{17}ONNa^+$ requires 190.1202, found 190.1199.

IR (cm^{-1}): 2967, 2932, 1651, 1456, 1422, 1385, 1269, 1116, 969.

2-(Cyclohex-1-en-1-yl)-1-(pyrrolidin-1-yl)-ethan-1-one (1h)

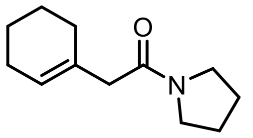

Following general procedure C using 2-(cyclohex-1-en-1-yl)-acetic acid **6h** (0.42 g; 3.0 mmol; 1.0 equiv.), pyrrolidine (0.25 ml; 3.0 mmol; 1.0 equiv.), triethylamine (0.42 ml; 3.0 mmol; 1.0 equiv.), 1-hydroxybenzotriazole hydrate (0.41 g; 3.0 mmol; 1.0 equiv.) and 1-(3-dimethylaminopropyl)-3-ethylcarbodiimide hydrochloride (0.58 g; 3.0 mmol; 1.0 equiv.). Purification by flash column chromatography (heptane/ethyl acetate 20–100%) gave the title compound (88.6 mg; 15% over 2 steps) as a yellow oil.

^{1}H NMR (400 MHz, $CDCl_3$): δ 5.50–5.45 (br s, 1H); 3.47 (dt, *J* = 22.1, 6.9 Hz, 4H); 2.94 (s, 2H); 2.05–1.96 (m, 4H); 1.95–1.80 (m, 4H); 1.71–1.52 (m, 4H) ppm.

^{13}C NMR (150 MHz, $CDCl_3$): δ 169.9, 132.0, 124.2, 46.9, 45.8, 44.8, 28.8, 26.4, 25.4, 24.6, 22.9, 22.3 ppm.

HRMS (ESI+): Calculated for $[M+Na]^+$ $C_{12}H_{19}ONNa^+$: 216.1359, found: 216.1357.

IR (cm^{-1}): 2924, 2870, 1631, 1420, 1340, 1288, 1226, 1191, 1168, 1117, 1039, 916, 860, 786, 644.

All analytical data were in good accordance with data reported in the literature.[9]

Methyl (*E*)-12-oxo-12-(pyrrolidin-1-yl)-dodec-9-enoate (1i)

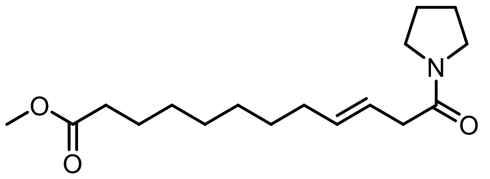

Following general procedure C using (*E*)-12-ethoxy-12-oxododec-3-enoic acid **6i** (0.46 g; 2.0 mmol; 1.0 equiv.), pyrrolidine (0.16 ml; 2.0 mmol; 1.0 equiv.), triethylamine (0.28 ml; 2.0 mmol; 1.0 equiv.), 1-hydroxybenzotriazole hydrate (0.27 g; 2.0 mmol; 1.0 equiv.) and 1-(3-dimethylaminopropyl)-3-ethylcarbodiimide hydrochloride (0.38 g; 2.0 mmol; 1.0 equiv.). Purification by flash column chromatography (heptane/ethyl acetate 20-100%) gave the title compound (494 mg; 84% over 2 steps) as a slightly yellow oil.

^{1}H NMR (400 MHz, $CDCl_3$): δ 5.59–5.54 (m, 2H), 3.65 (s, 3H), 3.44 (m, 4H), 3.00 (d, *J* = 5.4 Hz, 2H), 2.28 (t, *J* = 7.6 Hz, 2H), 2.01 (app q, *J* = 6.5 Hz, 2H), 1.97–1.88 (m, 2H), 1.87–1.78 (m, 2H), 1.65–1.54 (m, 2H), 1.38–1.22 (m, 8H) ppm.

^{13}C NMR (150 MHz, $CDCl_3$): δ 174.4, 170.2, 134.0, 122.8, 51.5, 46.7, 45.8, 39.3, 34.2, 32.6, 29.3, 29.2 (2C), 29.1, 26.3, 25.0, 24.5 ppm.

HRMS (ESI+): Calculated for $[M+Na]^+$ $C_{17}H_{29}O_3NNa^+$: 318.2040, found: 318.2041.

IR (cm^{-1}): 2927, 2856, 1735, 1640, 1424, 1342, 1194, 1169, 969, 914, 857, 813, 725, 702.

(*E*)-1-(Pyrrolidin-1-yl)dodec-3-ene-1,11-dione (1j)

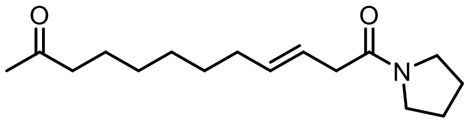

(*E*)-1-(Pyrrolidin-1-yl)-dodec-3-en-11-yn-1-one **1m** (99.0 mg; 0.4 mmol; 1.0 equiv.) and mercuric acetate (12.7 mg; 0.04 mmol; 0.1 equiv.) were dissolved in methanol (12 ml) and two drops of sulfuric acid (98%) were added. The reaction mixture was stirred at 60 °C for 3 hours. The mixture was allowed to cool to r.t., then aq. 1 M HCl (0.5 mL) was added, and the mixture stirred for 2 minutes. Then it was neutralized with sat. aq. solution of $NaHCO_3$ (1 ml). After extraction with diethyl ether the combined organic phases were dried over anhydrous magnesium sulfate. The dried solution was filtered, and the filtrate was concentrated under reduced pressure. The crude residue was purified by flash column chromatography (heptane/ethyl acetate 20-100%) to give the title compound (90.0 mg; 85%) as a slightly yellow oil.

^{1}H NMR (400 MHz, $CDCl_3$): δ 5.60–5.43 (m, 2H), 3.48–3.37 (m, 4H), 3.00 (d, *J* = 5.1 Hz, 2H), 2.35 (t, *J* = 7.5 Hz, 2H), 2.10 (s, 3H), 2.04–1.96 (m, 2H), 1.96–1.88 (m, 2H), 1.87–1.78 (m, 2H), 1.58–1.48 (m, 2H), 1.39–1.21 (m, 6H) ppm.

^{13}C NMR (150 MHz, $CDCl_3$): δ 209.4, 170.3, 134.0, 122.7, 46.7, 45.9, 43.8, 39.2, 32.5, 29.9, 29.12, 29.06, 29.0, 26.2, 24.5, 23.9 ppm.

HRMS (ESI+): Calculated for $[M+Na]^+$ $C_{16}H_{27}O_2NNa^+$: 288.1934, found: 288.1942.

IR (cm^{-1}): 2926, 2855, 1712, 1638, 1426, 1357, 1276, 1260, 1167, 968, 750.

(*E*)-9-Oxo-9-(pyrrolidin-1-yl)-non-6-enenitrile (1k)

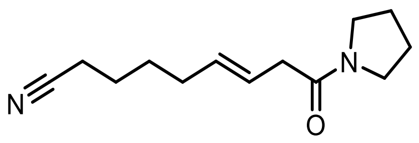

(*E*)-8-Chloro-1-(pyrrolidin-1-yl)-oct-3-en-1-one **1l** (0.46 g; 2.0 mmol; 1.0 equiv.), sodium iodide (79.4 mg; 0.50 mmol; 0.25 equiv.) and potassium cyanide (156 mg; 2.4 mmol; 1.2 equiv.) were combined in a 2:1 mixture of ethanol and water (0.2 M) and stirred for 18 h at 70 °C. The solvents were removed under reduced pressure and the residue was passed through a short column of silica gel, eluting with ethyl acetate. After removal of the solvent under reduced pressure, purification by flash column chromatography (heptane/ethyl acetate 20-100%) gave the title compound as a slightly yellow oil (216 mg, 50%).

^{1}H NMR (400 MHz, CDCl$_3$): δ 5.66–5.43 (m, 2H), 3.43 (dt, *J* = 14.5, 7.0 Hz, 4H), 3.01 (d, *J* = 6.4 Hz, 2H), 2.33 (t, *J* = 7.2 Hz, 2H), 2.08 (app q, *J* = 7.0 Hz, 2H), 1.98–1.89 (m, 2H), 1.88–1.79 (m, 2H), 1.70–1.60 (m, 2H), 1.58–1.45 (m, 2H) ppm.

^{13}C NMR (150 MHz, CDCl$_3$): δ 169.9, 132.5, 124.1, 119.8, 46.7, 45.8, 39.0, 31.7, 28.2, 26.3, 24.9, 24.5, 17.1 ppm.

HRMS (ESI+): Calculated for $[M+Na]^+$ $C_{13}H_{20}ON_2Na^+$: 243.1468, found: 243.1467.

IR (cm^{-1}): 2931, 2871, 2245, 1626, 1427, 1341, 1227, 1191, 1169, 969, 915, 859, 729.

(*E*)-8-Chloro-1-(pyrrolidin-1-yl)-oct-3-en-1-one (1l)

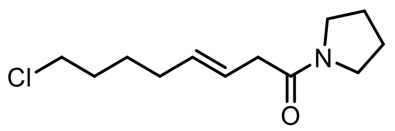

Following general procedure C using (*E*)-8-chlorooct-3-enoic acid **6l** (1.77 g; 10.0 mmol; 1.0 equiv.), pyrrolidine (0.82 ml; 10.0 mmol; 1.0 equiv.), triethylamine (1.39 ml; 10.0 mmol; 1.0 equiv.), 1-hydroxybenzotriazole hydrate (1.35 g; 10.0 mmol; 1.0 equiv.) and 1-(3-dimethylaminopropyl)-3-ethylcarbodiimide hydrochloride (1.92 g; 10.0 mmol; 1.0 equiv.). Purification by flash column chromatography (heptane/ethyl acetate 20-100%) gave the title compound (1.24 g; 54% over 2 steps) as a slightly yellow oil.

^{1}H NMR (400 MHz, CDCl$_3$): δ 5.62–5.42 (m, 2H), 3.50 (t, *J* = 6.6 Hz, 2H), 3.46–3.36 (m, 4H), 2.99 (d, *J* = 6.3 Hz, 2H), 2.05 (app q, *J* = 7.0 Hz, 2H), 1.96–1.87 (m, 2H), 1.86–1.70 (m, 4H), 1.54–1.44 (m, 2H) ppm.

^{13}C NMR (150 MHz, CDCl$_3$): δ 169.9, 133.0, 123.6, 46.7, 45.8, 45.0, 39.1, 32.1, 31.8, 26.5, 26.2, 24.4 ppm.

HRMS (ESI+): Calculated for $[M+Na]^+$ $C_{12}H_{20}ONClNa^+$: 252.1126, found: 252.1124.

IR (cm^{-1}): 2934, 2868, 1635, 1425, 1341, 1226, 1191, 1169, 967, 914, 859, 749, 722, 645.

(*E*)-1-(Pyrrolidin-1-yl)-dodec-3-en-11-yn-1-one (1m)

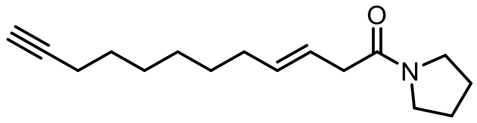

Following general procedure C using (*E*)-dodec-3-en-11-ynoic acid **6m** (0.78 g; 4.0 mmol; 1.0 equiv.), pyrrolidine (0.33 ml; 4.0 mmol; 1.0 equiv.), triethylamine (0.56 ml; 4.0 mmol; 1.0 equiv.), 1-hydroxybenzotriazole hydrate (0.54 g; 4.0 mmol; 1.0 equiv.) and 1-(3-dimethylaminopropyl)-3-ethylcarbodiimide hydrochloride (0.77 g; 4.0 mmol; 1.0 equiv.). Purification by flash column chromatography (heptane/ethyl acetate 20-100%) gave the title compound (776 mg; 78% over 2 steps) as a slightly yellow oil.

^{1}H NMR (400 MHz, CDCl$_3$): δ 5.59–5.44 (m, 2H), 3.47–3.35 (m, 4H), 2.99 (d, *J* = 5.6 Hz, 2H), 2.14 (dt, *J* = 7.0, 2.3 Hz, 2H), 2.01 (app q, *J* = 5.6 Hz, 2H), 1.96–1.87 (m, 3H), 1.86–1.77 (m, 2H), 1.54–1.44 (m, 2H), 1.42–1.20 (m, 6H) ppm.

^{13}C NMR (150 MHz, CDCl$_3$): δ 170.1, 133.9, 122.8, 84.8, 68.2, 46.7, 45.8, 39.3, 32.5, 29.2, 28.7, 28.6, 28.5, 26.2, 24.5, 18.4 ppm.

HRMS (ESI+): Calculated for $[M+Na]^+$ $C_{16}H_{25}ONNa^+$: 270.1828, found: 270.1830.

IR (cm^{-1}): 3310, 3228, 2968, 2857, 1738, 1634, 1425, 1372, 1342, 1227, 1192, 1169, 1038, 969, 914, 860, 626.

(*R*,*E*)-5,9-Dimethyl-1-(pyrrolidin-1-yl)-deca-3,8-dien-1-one (1n)

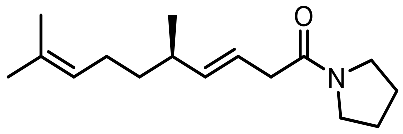

Following general procedure C using (*R*,*E*)-5,9-dimethyldeca-3,8-dienoic acid **6n** (0.39 g; 2.0 mmol; 1.0 equiv.), pyrrolidine (0.16 ml; 2.0 mmol; 1.0 equiv.), triethylamine (0.28 ml; 2.0 mmol; 1.0 equiv.), 1-hydroxybenzotriazole hydrate (0.27 g; 2.0 mmol; 1.0 equiv.) and 1-(3-dimethylaminopropyl)-3-ethylcarbodiimide hydrochloride (0.38 g; 2.0 mmol; 1.0 equiv.). Purification by flash column chromatography (heptane/ethyl acetate 20-100%) gave the title compound (0.32 g; 65% over 2 steps) as a yellow oil.

^{1}H NMR (400 MHz, CDCl$_3$): δ 5.52 (dt, *J* = 15.4, 6.4 Hz, 1H), 5.39 (dd, *J* = 15.4, 7.3 Hz, 1H), 5.07 (t, *J* = 7.0 Hz, 1H), 3.44 (dt, *J* = 12.3, 7.0 Hz, 4H), 3.02 (d, *J* = 6.5 Hz, 2H), 2.18–2.08 (m, 1H), 1.98–1.89 (m, 4H), 1.83 (dt, *J* = 13.8, 6.4 Hz, 2H), 1.66 (s, 3H), 1.57 (s, 3H), 1.29 (app q, *J* = 8.1 Hz, 2H), 0.97 (d, *J* = 6.7 Hz, 3H) ppm.

^{13}C NMR (150 MHz, CDCl$_3$): δ 170.2, 139.7, 131.4, 124.8, 121.3, 46.7, 45.8, 39.5, 37.2, 36.4, 26.3, 25.9, 25.8, 24.5, 20.6, 17.8 ppm.

HRMS (ESI+): Calculated for $[M+Na]^+$ $C_{16}H_{27}ONNa^+$: 272.1985, found: 272.1986.

IR (cm^{-1}): 2959, 2910, 2871, 1639, 1422, 1341, 1254, 1226, 1192, 1116, 969, 859, 828, 733.

(*E*)-*N*-Butyldodec-3-enamide (1o)

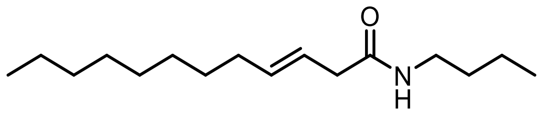

Following general procedure C using (*E*)-dodec-3-enoic acid **6b** (0.40 g; 2.0 mmol; 1.0 equiv.), *n*-butylamine (0.20 ml; 2.0 mmol; 1.0 equiv.), triethylamine (0.28 ml; 2.0 mmol; 1.0 equiv.), 1-hydroxybenzotriazole hydrate (0.27 g; 2.0 mmol; 1.0 equiv.) and 1-(3-dimethylaminopropyl)-3-ethylcarbodiimide

hydrochloride (0.38 g; 2.0 mmol; 1.0 equiv.). Purification by flash column chromatography (heptane/ethyl acetate 0-50%) gave the title compound (0.25 g; 49% over 2 steps) as a colourless oil.

^{1}H NMR (400 MHz, CDCl$_3$): δ 5.67–5.56 (m, 1H), 5.54–5.39 (m, 1H), 3.29–3.18 (m, 2H), 2.92 (d, *J* = 6.9 Hz, 1H), 2.14 (t, *J* = 7.3, 1H), 2.04 (app q, *J* = 6.8 Hz, 1H), 1.61 (t, *J* = 6.8 Hz, 1H), 1.52–1.41 (m, 2H), 1.41–1.18 (m, 15H), 0.95–0.83 (m, 6H) ppm.

^{13}C NMR (150 MHz, CDCl$_3$): δ 171.4, 136.8, 122.8, 40.7, 39.4, 37.1, 32.7, 32.0, 31.8, 29.6, 29.3, 26.0, 22.8, 20.2, 14.2, 13.9 ppm.

HRMS (ESI+): Calculated for $[M+Na]^+$ $C_{16}H_{31}ONNa^+$: 276.2298 found: 276.2298.

IR (cm^{-1}): 3284, 2923, 2873, 2853, 1642, 1550, 1466, 1437, 1378, 1228, 1157, 968, 722.

(*E*)-1-(Azepan-1-yl)-pent-3-en-1-one (1p)

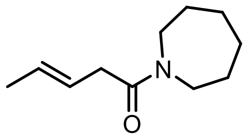

Following general procedure C using (*E*)-3-pentenoic acid (0.51 ml; 5.0 mmol; 1.0 equiv.), hexamethyleneimine (0.56 ml; 5.0 mmol; 1.0 equiv.), triethylamine (0.70 ml; 5.0 mmol; 1.0 equiv.), 1-hydroxybenzotriazole hydrate (0.68 g; 5.0 mmol; 1.0 equiv.) and 1-(3-dimethylaminopropyl)-3-ethylcarbodiimide hydrochloride (0.96 g; 5.0 mmol; 1.0 equiv.). Purification by flash column chromatography (heptane/ethyl acetate 20-100%) gave the title compound (0.82 g, 90%) as a slightly yellow oil.

^{1}H NMR (400 MHz, CDCl$_3$): δ 5.56–5.49 (m, 2H), 3.51 (t, *J* = 5.9 Hz, 2H), 3.43 (t, *J* = 5.9 Hz, 2H), 3.07 (d, *J* = 6.0 Hz, 2H), 1.75–1.67 (m, 7H), 1.60–1.52 (m, 4H) ppm.

^{13}C NMR (150 MHz, CDCl$_3$): δ 171.3, 128.3, 124.6, 48.1, 46.1, 37.9, 29.4, 27.7, 27.3, 27.0, 18.2 ppm.

HRMS (ESI+): Calculated for $[M+Na]^+$ $C_{11}H_{19}ONNa^+$: 204.1359, found: 204.1360.

IR (cm^{-1}): 2922, 1632, 1432, 1279, 1169, 1099, 963.

(*E*)-1-(Azetidin-1-yl)-pent-3-en-1-one (1q)

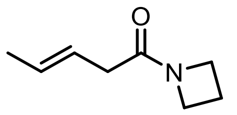

Following general procedure C using (*E*)-3-pentenoic acid (0.17 g; 3.0 mmol; 1.0 equiv.), azetidine hydrochloride (0.28 g; 3.0 mmol; 1.0 equiv.), triethylamine (0.84 ml; 6.0 mmol; 2.0 equiv.), 1-hydroxybenzotriazole hydrate (0.41 g; 3.0 mmol; 1.0 equiv.) and 1-(3-dimethylaminopropyl)-3-ethylcarbodiimide hydrochloride (0.58 g; 3.0 mmol; 1.0 equiv.). Purification by flash column chromatography (heptane/ethyl acetate 20-100%) gave the title compound (0.12 g; 29%) as a yellow oil.

^{1}H NMR (400 MHz, CDCl$_3$): δ 5.60–5.47 (m, 2H), 4.14 (t, *J* = 7.6 Hz, 2H), 4.02 (t, *J* = 7.6 Hz, 2H), 2.81 (d, *J* = 4.5 Hz, 2H), 2.30–2.20 (m, 2H), 1.68 (d, *J* = 3.9 Hz, 3H) ppm.

^{13}C NMR (150 MHz, CDCl$_3$): δ 171.5, 129.0, 123.6, 50.4, 48.1, 36.0, 18.1, 15.2 ppm.

HRMS (ESI+): Calculated for $[M+Na]^+$ $C_8H_{13}ONNa^+$: 162.0889, found: 162.0891.

IR (cm^{-1}): 2967, 2883, 1639, 1423, 1299, 1240, 1155, 1116, 1079, 1017, 966.

(*E*)-*N*,*N*-Dimethylpent-3-en-amide (1r)

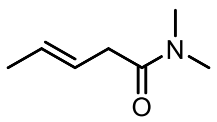

Following general procedure C using (*E*)-3-pentenoic acid (0.15 g; 1.5 mmol; 1.0 equiv.), dimethylamine hydrochloride (0.12 g; 1.5 mmol; 1.0 equiv.), triethylamine (0.42 ml; 3.0 mmol; 2.0 equiv.), 1-hydroxybenzotriazole hydrate (0.20 g; 1.5 mmol; 1.0 equiv.) and 1-(3-dimethylaminopropyl)-3-ethylcarbodiimide hydrochloride (0.29 g; 1.5 mmol; 1.0 equiv.). Purification by flash column chromatography (heptane/ethyl acetate 0-70%) gave the title compound (0.07 g; 37%) as a yellow oil.

^{1}H NMR (600 MHz, CDCl$_3$): δ 5.82–5.28 (m, 2H), 3.06 (d, *J* = 5.4 Hz, 2H), 3.00 (s, 3H), 2.93 (s, 3H), 1.69 (d, *J* = 4.7 Hz, 3H) ppm.

^{13}C NMR (150 MHz, CDCl$_3$): δ 171.8, 128.6, 124.0, 37.9, 37.5, 35.6, 18.1 ppm.

HRMS (ESI$^+$): Calculated for $[M+Na]^+$ $C_7H_{13}ONNa^+$: 150.0889, found: 150.0891.

IR (cm^{-1}): 3476, 2928, 1633, 1495, 1446, 1396, 1142, 1094, 1059, 966, 810, 602.

(*E*)-1-Morpholinopent-3-en-1-one (1s)

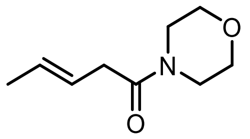

Following general procedure C using (*E*)-3-pentenoic acid (0.15 g; 1.5 mmol; 1.0 equiv.), morpholine (0.13 ml; 1.5 mmol; 1.0 equiv.), triethylamine (0.21 ml; 1.5 mmol; 1.0 equiv.), 1-hydroxybenzotriazole hydrate (0.20 g; 1.5 mmol; 1.0 equiv.) and 1-(3-dimethylaminopropyl)-3-ethylcarbodiimide hydrochloride

(0.29 g; 1.5 mmol; 1.0 equiv.). Purification by flash column chromatography (heptane/ethyl acetate 0-70%) gave the title compound (0.17 g; 67%) as a yellow oil.

^{1}H NMR (400 MHz, $CDCl_3$): δ 5.62–5.44 (m, 2H), 3.65 (d, *J* = 4.1 Hz, 4H), 3.62–3.58 (m, *J* = 4.7 Hz, 2H), 3.47–3.43 (m, 2H), 3.06 (d, *J* = 3.5 Hz, 2H), 1.70 (d, *J* = 3.4 Hz, 3H) ppm.

^{13}C NMR (150 MHz, $CDCl_3$): δ 170.3, 129.0, 123.7, 67.0, 66.8, 46.3, 42.1, 37.6, 18.1 ppm.

HRMS (ESI$^+$): Calculated for $[M+Na]^+$ $C_9H_{15}O_2NNa^+$: 192.0995, found: 192.0997.

IR (cm^{-1}): 2962, 2916, 2855, 2223, 1636, 1429, 1226, 1110, 1065, 1033, 962, 702.

(*E*)-*N*,*N*-Dibenzylpent-3-en-amide (1t)

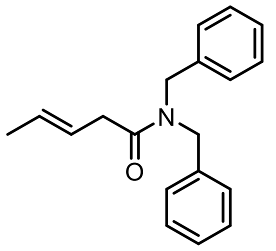

Following general procedure C using (*E*)-3-pentenoic acid (0.15 g; 1.5 mmol; 1.0 equiv.), dibenzylamine (0.30 ml; 1.5 mmol; 1.0 equiv.), triethylamine (0.21 ml; 1.5 mmol; 1.0 equiv.), 1-hydroxybenzotriazole hydrate (0.20 g; 1.5 mmol; 1.0 equiv.) and 1-(3-dimethylaminopropyl)-3-ethylcarbodiimide hydrochloride (0.29 g; 1.5 mmol; 1.0 equiv.). Purification by flash column chromatography (heptane/ethyl acetate 0-20%) gave the title compound (0.31 g; 74%) as a yellow oil.

^{1}H NMR (400 MHz, $CDCl_3$): δ 7.45–7.05 (m, 10H), 5.76–5.58 (m, 1H), 5.58–5.43 (m, 1H), 4.59 (s, 2H), 4.45 (s, 2H), 3.17 (d, *J* = 6.5 Hz, 2H), 1.69 (dd, *J* = 6.3, 1.3 Hz, 3H).

^{13}C NMR (150 MHz, $CDCl_3$): δ 172.4, 137.5, 136.6, 129.1 (2C), 129.0, 128.7 (2C), 128.5 (2C), 127.8, 127.5, 126.6 (2C), 124.1, 50.1, 48.3, 37.8, 18.1 ppm.

HRMS (ESI$^+$): Calculated for $[M+Na]^+$ $C_{19}H_{21}ONNa^+$: 302.1515, found: 302.1521.

IR (cm^{-1}): 3029, 2922, 1641, 1492, 1422, 1358, 1299, 1077, 1027, 958, 734, 696, 620.

(*E*)-*N*-Butyl-*N*-(3-methylbut-2-en-1-yl)-pent-3-enamide (1u)

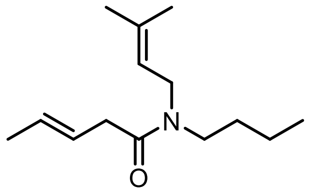

Following general procedure C using (*E*)-3-pentenoic acid (0.25 g; 2.5 mmol; 1.0 equiv.), *N*-butyl-3-methylbut-2-en-1-amine **8** (0.35 ml; 2.5 mmol; 1.0 equiv. – see below for the synthesis), triethylamine (0.35 ml; 2.5 mmol; 1.0 equiv.), 1-hydroxybenzotriazole hydrate (0.34 g; 2.5 mmol; 1.0 equiv.) and 1-(3-dimethylaminopropyl)-3-ethylcarbodiimide hydrochloride (0.48 g; 2.5 mmol; 1.0 equiv.). Purification by flash column chromatography (heptane/ethyl acetate 20-100%) gave the title compound (0.37 g; 66%) as a yellow oil.

2 rotamers are present in the NMR (1:0.8, only differentiable in the ^{13}C-spectrum).

^{1}H NMR (400 MHz, $CDCl_3$): δ 5.65 (m, 2H), 5.10 (dtt, *J* = 15.5, 7.0, 1.4 Hz, 1H), 3.95 (d, *J* = 7.0 Hz, 1H), 3.83 (d, *J* = 6.4 Hz, 1H), 3.28 (t, *J* = 7.5 Hz, 1H), 3.17 (t, *J* = 7.7 Hz, 1H), 3.04 (t, *J* = 6.7 Hz, 2H), 1.75–1.65 (m, 9H), 1.57–1.44 (m, 2H), 1.36–1.23 (m, 2H), 0.92 (dt, *J* = 10.6, 7.3 Hz, 3H) ppm.

^{13}C NMR (150 MHz, $CDCl_3$):

Major: δ 171.1, 135.3, 128.2, 124.7, 120.8, 46.0, 43.0, 37.9, 29.9, 25.8, 20.3, 18.1, 17.95, 13.97 ppm.

Minor: δ 171.2, 135.4, 128.2, 124.7, 120.6, 47.1, 45.6, 37.6, 31.1, 25.9, 20.4, 18.1, 18.0, 14.03 ppm.

HRMS (ESI+): Calculated for $[M+H]^+$ $C_{14}H_{25}ONH^+$: 224.2009, found: 224.2027.

IR (cm^{-1}): 2959, 2928, 2874, 1637, 1445, 1376, 1168, 1128, 1096, 965, 927, 845, 773, 731.

(*E*)-*N*-Allyl-*N*-methylpent-3-en-amide (1v)

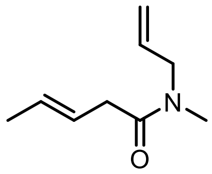

Following general procedure C using (*E*)-3-pentenoic acid (0.15 g; 1.5 mmol; 1.0 equiv.), *N*-allylmethylamine (0.15 ml; 1.5 mmol; 1.0 equiv.), triethylamine (0.21 ml; 1.5 mmol; 1.0 equiv.), 1-hydroxybenzotriazole hydrate (0.20 g; 1.5 mmol; 1.0 equiv.) and 1-(3-dimethylaminopropyl)-3-ethylcarbodiimide hydrochloride (0.29 g; 1.5 mmol; 1.0 equiv.). Purification by flash column chromatography (heptane/ethyl acetate 0-60%) gave the title compound (0.15 g; 65%) as a yellow oil.

2 rotamers are present in the NMR (1:0.9).

^{1}H NMR (400 MHz, $CDCl_3$):

Major: δ 5.85–5.65 (m, 1H), 5.64–5.44 (m, 2H), 5.26–5.05 (m, 2H), 3.90 (d, *J* = 5.2 Hz, 2H), 3.04 (d, *J* = 5.2 Hz, 2H), 2.91 (s, 3H), 1.69 (m, 3H).

Minor: δ 5.85–5.65 (m, 1H), 5.64–5.44 (m, 2H), 5.26–5.05 (m, 2H), 3.98 (d, *J* = 5.2 Hz, 2H), 3.08 (d, *J* = 5.2 Hz, 2H), 2.94 (s, 3H), 1.69 (m, 3H).

^{13}C NMR (150 MHz, $CDCl_3$):

Major: δ 172.0, 133.2, 128.7, 124.3, 117.4, 52.4, 38.0, 34.9, 18.1 ppm.

Minor: δ 171.5, 132.8, 128.6, 124.0, 116.9, 50.2, 37.5, 33.7, 18.1 ppm.

HRMS (ESI$^+$): Calculated for $[M+Na]^+$ $C_9H_{15}ONNa^+$: 176.1051, found: 176.1046.

IR (cm^{-1}): 2924, 2349, 2027, 1636, 1400, 1267, 1219, 1136, 1089, 965, 922, 816, 752.

(*E*)-*N*-cyclopropyl-*N*-methylpent-3-enamide (1w)

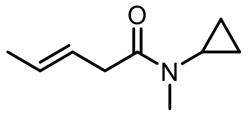

Following general procedure C using 3-pentenoic acid (0.20 g; 2.0 mmol; 1.0 equiv.), cyclopropylamine (0.14 ml; 2.0 mmol; 1.0 equiv.), triethylamine (0.28 ml; 2.0 mmol; 1.0 equiv.), 1-hydroxybenzotriazole hydrate (0.27 g; 2.0 mmol; 1.0 equiv.) and 1-(3-dimethylaminopropyl)-3-ethylcarbodiimide hydrochloride (0.38 g; 2.0 mmol; 1.0 equiv.). This crude product was dissolved in tetrahydrofuran (10 ml), after which sodium hydride (60% in paraffin oil, 160 mg, 4.0 mmol, 2.0 equiv.) was added at 0 °C and the mixture was stirred for 2 h. Subsequently, methyl iodide (126 µL, 2.0 mmol, 1.0 equiv.) was slowly added to the mixture and stirred for 12 h at room temperature. The mixture was poured into 50 ml of water, the aqueous layer extracted with CH_2Cl_2 (3 x 25 ml) and the organic phases were combined and dried over anhydrous sodium sulfate. The dried solution was filtered, and the filtrate was concentrated under reduced pressure. The crude residue was purified by flash column chromatography (SiO_2, heptane/ethyl acetate) to afford the product as a colourless oil (27.0 mg, 9% over 2 steps).

^{1}H NMR (600 MHz, $CDCl_3$) δ 5.63–5.57 (m, 1H), 5.56–5.48 (m, 1H), 3.23 (d, *J* = 6.4 Hz, 2H), 2.88 (s, 3H), 2.69–2.63 (m, 1H), 1.67 (dd, *J* = 6.2, 1.2 Hz, 3H), 0.88–0.80 (m, 2H), 0.76–0.68 (m, 2H) ppm

^{13}C NMR (151 MHz, $CDCl_3$) δ 174.6, 128.3, 124.6, 38.2, 34.1, 31.4, 18.1, 9.2 (2C) ppm.

HRMS (ESI) (m/z): Calculated for $[M+Na]^+$ $C_9H_{15}ONNa^+$: 176.1046, found 176.1046.

IR (cm^{-1}): 2918, 1653, 1425, 1386, 1284, 1125, 967, 732.

(*E*)-*N*-(cyclopropylmethyl)-*N*-methylpent-3-enamide (1x)

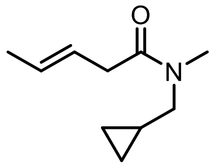

Following general procedure C using 3-pentenoic acid (0.20 g; 2.0 mmol; 1.0 equiv.), (aminomethyl)cyclopropane (0.17 ml; 2.0 mmol; 1.0 equiv.), triethylamine (0.28 ml; 2.0 mmol; 1.0 equiv.), 1-hydroxybenzotriazole hydrate (0.27 g; 2.0 mmol; 1.0 equiv.) and 1-(3-dimethylaminopropyl)-3-ethylcarbodiimide hydrochloride (0.38 g; 2.0 mmol; 1.0 equiv.). This crude product was dissolved in tetrahydrofuran (10 ml), after which sodium hydride (60% in paraffin oil, 160 mg, 4.0 mmol, 2.0 equiv.) was added at 0 °C and the mixture was stirred for 2 h. Subsequently, methyl iodide (126 µL, 2.0 mmol, 1.0 equiv.) was slowly added to the mixture and stirred for 12 h at room temperature. The mixture was poured into 50 ml of water, the aqueous layer extracted with CH_2Cl_2 (3 x 25 ml) and the organic phases were combined and dried over anhydrous sodium sulfate. The dried solution was filtered, and the filtrate was concentrated under reduced pressure. The crude residue was purified by flash column chromatography (SiO_2, heptane/ethyl acetate) to afford the product as a colourless oil (53.0 mg, 16%).

2 rotamers are present in the NMR (1:1).

^{1}H NMR (600 MHz, $CDCl_3$)

δ 5.69–5.37 (m, 2H), 3.26 (d, *J* = 7.0 Hz, 2H), 3.12–3.05 (m, 2H), 3.04 (s, 3H), 1.72–1.67 (m, 3H), 1.02–0.87 (m, 1H), 0.60–0.55 (m, 1H), 0.51–0.46 (m, 1H), 0.27–0.18 (m, 2H) ppm.

δ 5.69–5.37 (m, 2H), 3.17 (d, *J* = 6.7 Hz, 2H), 3.12–3.05 (m, 2H), 2.98 (s, 3H), 1.72–1.67 (m, 3H), 1.02–0.87 (m, 1H), 0.60–0.55 (m, 1H), 0.51–0.46 (m, 1H), 0.27–0.18 (m, 2H) ppm.

^{13}C NMR (151 MHz, $CDCl_3$):

δ 171.4, 128.4, 124.2, 51.8, 38.1, 35.6, 18.2, 9.5, 3.5 (2C) ppm.

δ 171.3, 128.4, 124.5, 54.4, 37.7, 33.6, 18.1, 10.3, 3.7 (2C) ppm.

HRMS (ESI) : Calculated for $[M+Na]^+$ $C_{10}H_{17}ONNa^+$: 190.1202, found 190.1199.

IR (cm^{-1}): 1635, 1400, 1152, 1089, 1018, 965.

(*E*)-1-(2-phenylpyrrolidin-1-yl)-dodec-3-en-1-one (1y)

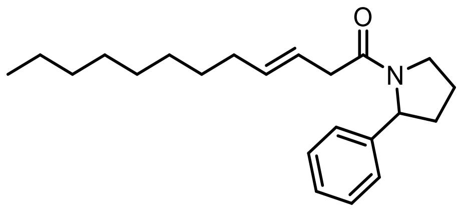

Following general procedure C using (*E*)-dodec-3-enoic acid **6b** (0.40 g; 2.0 mmol; 1.0 equiv.), 2-phenylpyrrolidine (0.29 ml; 2.0 mmol; 1.0 equiv.), triethylamine (0.28 ml; 2.0 mmol; 1.0 equiv.), 1-hydroxybenzotriazole hydrate (0.27 g; 2.0 mmol; 1.0 equiv.) and 1-(3-dimethylaminopropyl)-3-ethylcarbodiimide hydrochloride (0.38 g; 2.0 mmol; 1.0 equiv.). Purification by flash column chromatography (heptane/ethyl acetate 20-100%) gave the title compound (0.25 g; 38% over 2 steps) as a slightly yellow oil.

2 rotamers are present in the NMR (1:0.4).

^{1}H NMR (400 MHz, CDCl$_3$):

Major: δ 7.37–7.23 (m, 3H), 7.21–7.11 (m, 2H), 5.31–5.19 (m, 1H), 4.96 (dd, *J* = 16.7, 7.7 Hz, 1H), 3.82–3.67 (m, 2H), 2.78 (ddd, *J* = 59.9, 15.5, 6.8 Hz, 1 H), 2.44–2.30 (m, 1H), 2.00–1.81 (m, 5H), 1.35–1.08 (m, 14H), 0.87 (t, *J* = 5.9 Hz, 3H) ppm.

Minor: δ 7.37–7.23 (m, 3H), 7.21–7.11 (m, 2H), 5.61–5.56 (m, 1H), 5.50–5.40 (m, 1H), 3.66–3.56 (m, 2H), 3.16–3.06 (m, 1H), 2.30–2.18 (m, 1H), 2.19–2.00 (m, 4H), 1.74–1.60 (m, 1H), 1.58–1.41 (m, 2H), 1.35–1.08 (m, 12H) 0.87 (t, *J* = 5.9 Hz, 3H) ppm.

^{13}C NMR (150 MHz, CDCl$_3$):

Major: δ 171.3, 143.5, 134.0, 128.8, 128.8, 127.4, 126.7, 125.6, 122.8, 61.5, 47.2, 39.0, 36.4, 34.7, 34.1, 32.7, 32.0, 29.5, 29.4, 25.1, 21.8, 14.2 ppm.

Minor: δ 173.0, 143.7, 134.4, 128.9, 127.3, 126.7, 125.6, 122.7, 60.5, 47.8, 39.5, 35.1, 32.7, 32.0, 29.62, 29.59, 29.4, 29.3, 25.0, 22.8, 21.8, 14.2 ppm.

HRMS (ESI+): Calculated for $[M+Na]^+$ $C_{22}H_{33}ONNa^+$: 350.2454, found: 350.2452.

IR (cm^{-1}): 2922, 2854, 1739, 1644, 1451, 1413, 1321, 1303, 1240, 1075, 1046, 1029, 969, 749, 722, 699.

(*R*,*E*)-*N*-Butyl-5,9-dimethyl-*N*-(3-methylbut-2-en-1-yl)-deca-3,8-dienamide (1z)

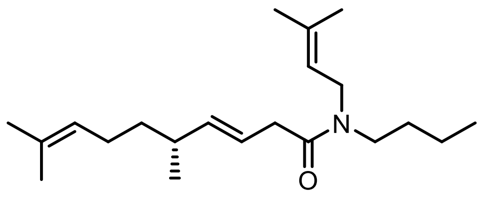

Following general procedure C using (*R*,*E*)-5,9-dimethyldeca-3,8-dienoic acid **6n** (0.49 g; 2.5 mmol; 1.0 equiv.), *N*-butyl-3-methylbut-2-en-1-amine **8** (0.35 ml; 2.5 mmol; 1.0 equiv.), triethylamine (0.35 ml; 2.5 mmol; 1.0 equiv.), 1-hydroxybenzotriazole hydrate (0.34 g; 2.5 mmol; 1.0 equiv.) and 1-(3-dimethylaminopropyl)-3-ethylcarbodiimide hydrochloride (0.48 g; 2.5 mmol; 1.0 equiv.). Purification by flash column chromatography (heptane/ethyl acetate 20-100%) gave the title compound (0.16 g; 20% over 2 steps) as a yellow oil.

2 rotamers are present in the NMR (1:0.9, only differentiable in the ^{13}C-spectrum).

^{1}H NMR (400 MHz, CDCl$_3$): δ 5.59–5.48 (m, 1H), 5.37 (dd, *J* = 7.5 Hz, 1H), 5.15–5.04 (m, 2H), 3.96 (d, *J* = 6.6 Hz, 1H), 3.84 (d, *J* = 6.2 Hz, 1H), 3.28 (t, *J* = 7.4 Hz, 1H), 3.18 (t, *J* = 7.7 Hz, 1H), 3.05 (t, *J* = 6.6 Hz, 2H), 2.18–2.08 (m, 1H), 1.94 (app q, *J* = 7.4 Hz, 2H), 1.72 (d, *J* = 9.0 Hz, 3H), 1.67 (s, 6H), 1.58 (s, 3H), 1.56–1.44 (m, 2H), 1.37–1.22 (m, 4H), 1.00–0.87 (m, 6H) ppm.

^{13}C NMR (150 MHz, CDCl$_3$):

Major: δ 171.3, 139.3, 135.4, 131.4, 124.8, 122.0, 120.6, 46.0, 42.9, 38.1, 37.2, 36.42, 29.9, 25.9, 25.84 (2C), 20.58, 20.56, 20.4, 17.8, 14.02 ppm.

Minor: δ 171.1, 139.3, 135.2, 131.4, 124.8, 121.9, 120.9, 47.1, 45.5, 37.8, 37.2, 36.38, 31.1, 25.9, 25.78 (2C), 20.3, 18.01, 17.96, 17.8 13.96 ppm.

HRMS (ESI+): Calculated for $[M+Na]^+$ $C_{21}H_{37}ONH^+$: 342.2767, found: 342.2771.

IR (cm^{-1}): 2961, 2926, 2867, 1636, 1448, 1376, 1275, 1267, 1261, 970, 923, 765, 749, 730, 645.

(*E*)-*N*-(3-(10,11-dihydro-5*H*-dibenzo[b,f]azepin-5-yl)propyl)-*N*-methylpent-3-enamide (1ab)

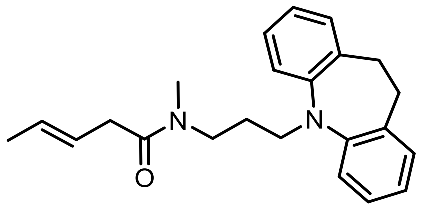

Following general procedure C using (*E*)-3-pentenoic acid (81 µl; 0.8 mmol; 1.0 equiv.), desipramine hydrochloride (247 mg; 0.8 mmol; 1.0 equiv.), triethylamine (0.22 ml; 1.6 mmol; 2.0 equiv.), 1-hydroxybenzotriazole hydrate (108 mg; 0.8 mmol; 1.0 equiv.) and 1-(3-dimethylaminopropyl)-3-ethylcarbodiimide hydrochloride (153 g; 0.8 mmol; 1.0 equiv.). Purification by flash column chromatography (heptane/ethyl acetate 20-100%) gave the title compound (254 mg, 91%) as a thick colourless oil.

2 rotamers are present in the NMR (1:0.95).

^{1}H NMR (600 MHz, CDCl$_3$):

Major: δ 7.19–7.03 (m, 6H), 6.98–6.89 (m, 2H), 5.57–5.48 (m, 1H), 5.48–5.41 (m, 1H), 3.76 (t, *J* = 6.2 Hz, 2H), 3.33–3.27 (m, 2H), 3.18 (s, 4H), 2.80 (s, 3H), 2.77 (d, *J* = 6.5 Hz, 2H), 1.87–1.82 (m, 2H), 1.64 (dd, *J* = 6.4, 1.4 Hz, 3H) ppm.

Minor: δ 7.19–7.03 (m, 6H), 6.98–6.89 (m, 2H), 5.57–5.48 (m, 1H), 5.33–5.26 (m, 1H), 3.74 (t, *J* = 7.0 Hz, 2H), 3.42–3.37 (m, 2H), 3.16 (s, 4H), 3.02–2.99 (m, 2H), 2.83 (s, 3H), 1.79 (dt, *J* = 14.2, 7.2 Hz, 2H), 1.68 (d, *J* = 4.9 Hz, 3H) ppm.

^{13}C NMR (100 MHz, $CDCl_3$):

Major: δ 171.54, 147.9 (2C), 134.3 (2C), 130.2 (2C), 128.2, 126.7 (2C), 124.4, 123.1 (2C), 119.8 (2C), 48.1, 47.5, 37.0, 33.5, 32.26 (2C), 26.6, 18.10 ppm.

Minor: δ 171.51, 148.3 (2C), 134.4 (2C), 130.0 (2C), 128.6, 126.6 (2C), 124.1, 122.7 (2C), 120.1 (2C), 48.2, 45.8, 38.1, 35.6, 32.33 (2C), 26.1, 18.14 ppm.

HRMS (ESI$^+$): Calculated for $[M+Na]^+$ $C_{23}H_{28}N_2ONa^+$: 371.2094, found: 371.2090.

IR (cm^{-1}): 2917, 1637, 1486, 1399, 1229, 1109, 945, 743, 600.

1-(Pyrrolidin-1-yl)-but-3-yn-1-one (1ac)

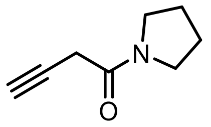

3-Butynoic acid (0.21 g; 2.5 mmol; 1.0 equiv.) was dissolved in CH_2Cl_2 (2.5 ml, 1 M) and a 5 drops of dimethylformamide were added. Oxalyl chloride (0.23 ml; 2.75 mmol; 1.10 equiv.) was added and the reaction was stirred at room temperature for 1 h. Then, pyrrolidine (0.51 ml; 6.25 mmol; 5.0 equiv.) and triethylamine (0.73 ml; 6.25 mmol; 2.5 equiv.) were slowly added, after which the reaction was stirred at room temperature for 30 min. Subsequently, the mixture was filtered through a pad of silica gel, eluting with diethyl ether. The solvent was removed under reduced pressure to give a crude product, which was purified by flash column chromatography (SiO_2, heptane/ethyl acetate) to afford the title compound together with an inseparable impurity (36.8 mg, estimated 95% purity). The mixture was used in the next step directly.

^{1}H NMR (400 MHz, $CDCl_3$): δ 3.55–3.45 (m, 3H), 3.89 (dt, *J* = 15.7, 6.6 Hz, 2H), 2.29 (s, 2H), 2.00–1.84 (m, 4H) ppm.

^{13}C NMR (150 MHz, $CDCl_3$): δ 165.1, 88.9, 51.5, 47.4, 46.0, 45.0, 26.2, 24.6 ppm.

HRMS (ESI+): Calculated for $[M+Na+H_2O]^+$ $C_8H_{11}ONNa^+ + H_2O$: 178.0838, found: 178.0851.

IR (cm^{-1}): 2968, 2873, 1718, 1631, 1434, 1381, 1357, 1226, 1193, 1159, 1021, 960, 933, 914, 860, 776, 719.

4-Methyl-1-(pyrrolidin-1-yl)-pent-3-en-1-one (1ad)

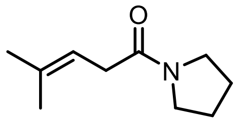

Following general procedure C using 4-methylpent-3-enoic acid **6ab** (84.0 mg; 0.74 mmol; 1.0 equiv.), pyrrolidine (0.06 ml; 0.74 mmol; 1.0 equiv., triethylamine (0.10 ml; 0.74 mmol; 1.0 equiv.), 1-hydroxybenzotriazole hydrate (0.99 g; 0.74 mmol; 1.0 equiv.) and 1-(3-dimethylaminopropyl)-3-ethylcarbodiimide hydrochloride (0.14 g; 0.74 mmol; 1.0 equiv.). Purification by flash column chromatography (heptane/ethyl acetate 20-100%) gave the title compound (72.0 mg; 58% as a mixture of α,β- and β,γ- double bond regioisomers: 1/0.16 in favour of the title compound) as a slightly yellow oil.

^{1}H NMR (400 MHz, $CDCl_3$): δ 5.37–5.29 (m, 1H), 3.48–3.38 (m, 4H), 3.00 (d, *J* = 6.8 Hz, 2H), 1.98–1.89 (m, 2H), 1. 87–1.78 (m, 2H), 1.73 (s, 3H), 1.64 (s, 3H) ppm.

^{13}C NMR (150 MHz, $CDCl_3$): δ 170.6, 134.5, 117.2, 46.7, 45.8, 35.0, 26.3, 24.5, 21.8, 18.2 ppm.

HRMS (ESI+): Calculated for $[M+Na]^+$ $C_{10}H_{17}ONNa^+$: 190.1202, found: 190.1209.

IR (cm^{-1}): 2967, 2871, 1634, 1420, 1346, 1277, 1268, 1256, 1192, 1168, 1116, 983, 936, 914, 845, 764, 750.

(*E*)-1-(Pyrrolidin-1-yl)pent-2-en-1-one (1ae)

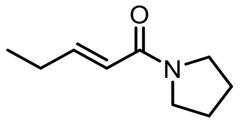

Following general procedure C using 2-pentenoic acid (0.50 g; 5.0 mmol; 1.0 equiv.), pyrrolidine (0.41 ml; 5.0 mmol; 1.0 equiv.), triethylamine (0.70 ml; 5.0 mmol; 1.0 equiv.), 1-hydroxybenzotriazole hydrate (0.68 g; 5.0 mmol; 1.0 equiv.) and 1-(3-dimethylaminopropyl)-3-ethylcarbodiimide hydrochloride (0.96 g; 5.0 mmol; 1.0 equiv.). Purification by flash column chromatography (heptane/ethyl acetate 20-100%) gave the title compound (0.62 g; 81%) as a colourless oil.

^{1}H NMR (600 MHz, $CDCl_3$) δ 6.95–6.85 (m, 1H), 6.04 (d, *J* = 15.1 Hz, 1H), 3.47 (t, *J* = 6.8 Hz, 4H), 2.19–2.15 (m, 2H), 1.91 (m, 2H), 1.81 (m, 2H), 1.02 (t, *J* = 7.4 Hz, 3H) ppm.

^{13}C NMR (151 MHz, $CDCl_3$) δ 165.0, 147.1, 120.8, 46.5, 45.8, 26.2, 25.5, 24.4, 12.7 ppm.

HRMS (ESI+): Calculated for $[M+Na]^+$ $C_9H_{15}ONNa^+$: 176.1046, found: 176.1045.

IR (cm^{-1}): 2965, 2872, 1660, 1611, 1417, 974.

(*E*)-*N*,*N*-Dimethyldodec-3-enamide (1af)

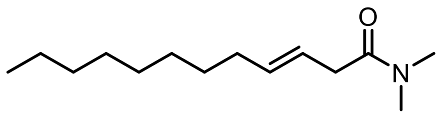

Following general procedure C using (*E*)-dodec-3-enoic acid **6b** (0.50 g; 2.5 mmol; 1.0 equiv.), dimethylamine hydrochloride (0.20 g; 2.5 mmol; 1.0 equiv.), triethylamine (0.70 ml; 5 mmol; 2.0 equiv.), 1-hydroxybenzotriazole hydrate (0.34 g; 2.5 mmol; 1.0 equiv.) and 1-(3-dimethylaminopropyl)-3-ethylcarbodiimide hydrochloride (0.48 g; 2.5 mmol; 1.0 equiv.). Purification by flash column chromatography (heptane/ethyl acetate 20-100%) gave the title compound (0.44 g; 78%) as a yellow oil.

^{1}H NMR (400 MHz, CDCl$_3$): δ 5.59–5.46 (m, 2H), 3.07 (d, *J* = 4.7 Hz, 2H), 3.00 (s, 3H), 2.93 (s, 3H), 2.06–1.99 (m, 2H), 1. 32–1.20 (br s, 12H), 0.87 (t, *J* = 6.7 Hz, 3H) ppm.

^{13}C NMR (150 MHz, CDCl$_3$): δ 171.7, 134.2, 122.8, 38.1, 37.5, 35.6, 32.7, 32.0, 29.6, 29.4 (2C), 29.3, 22.8, 14.2 ppm.

HRMS (ESI+): Calculated for $[M+Na]^+$ $C_{14}H_{27}ONNa^+$: 248.1985, found: 248.1982.

IR (cm^{-1}): 2952, 2923, 2852, 1645, 1491, 1465, 1394, 1265, 1141, 1061, 967, 722, 602.

1-(Pyrrolidin-1-yl)hex-3-yn-1-one (1ag)

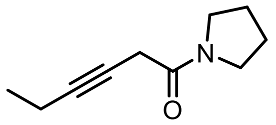

Following general procedure C using hex-3-ynoic acid[14] (168 mg; 1.5 mmol; 1.0 equiv.), pyrrolidine (0.12 ml; 1.5 mmol; 1.0 equiv.), triethylamine (0.21 ml; 1.5 mmol; 1.0 equiv.), 1-hydroxybenzotriazole hydrate (0.20 g; 1.5 mmol; 1.0 equiv.) and 1-(3-dimethylaminopropyl)-3-ethylcarbodiimide hydrochloride (0.29 g; 1.5 mmol; 1.0 equiv.). Purification by flash column chromatography (SiO_2, heptane/ethyl acetate 20-100%) gave the title compound (205 mg; 83%) as a yellow oil.

^{1}H NMR (400 MHz, CDCl$_3$): δ 3.47 (app dt, *J* = 17.9, 6.7 Hz, 4H), 3.16 (t, *J* = 2.4 Hz, 2H), 2.17 (tq, *J* = 7.6, 2.4 Hz, 2H), 2.00–1.90 (m, 2H), 1.89–1.80 (m, 2H), 1.09 (t, *J* = 7.5 Hz, 3H) ppm.

^{13}C NMR (150 MHz, CDCl$_3$): δ 166.3, 85.0, 71.8, 47.0, 46.2, 27.5, 26.2, 24.5, 14.0, 12.6 ppm.

HRMS (ESI+): Calculated for $[M+Na]^+$ $C_{10}H_{15}ONNa^+$: 188.1046, found: 188.1046.

IR (cm^{-1}): 2973, 2875, 1642, 1427, 1276, 1260, 1228, 1191, 750.

2.2.4 Additional reactions

Piperidinium acetate[10] (7)

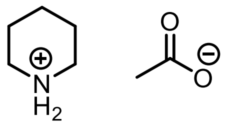

To a stirred mixture of piperidine (0.99 ml; 10.0 mmol; 1.0 equiv.) in diethyl ether (10.0 ml) at 0 °C, acetic acid (0.57 ml; 10.0 mmol; 1.0 equiv.) was added dropwise. The reaction was stirred at 0 °C for additional 5 min. The resulting crystals were filtered off using a Büchner funnel, washed with diethyl ether (2.0 ml) and dried under *vacuum* to afford the title compound (1.28 g, 88%).

^{1}H NMR (400 MHz, $CDCl_3$): δ 7.83–7.53 (m, 2H), 2.97 (t, *J* = 5.4 Hz, 4H), 1.97 (d, *J* = 2.1 Hz, 3H), 1.74 (br s, 4H), 1.65–1.57 (m, 2H) ppm.

All analytical data was in good accordance to the literature.[10]

***N*-Butyl-3-methylbut-2-en-1-amine (8)**

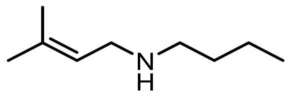

3-Methyl-2-butenal (0.62 g; 7.0 mmol; 1.0 equiv.) and magnesium sulfate (1.69 g; 14.0 mmol; 2.0 equiv.) were suspended in CH_2Cl_2 (0.5 M) and *n*-butylamine (0.56 g; 7.7 mmol; 1.1 equiv.) was added dropwise. The reaction was stirred at room temperature for 30 min, before the resulting suspension was filtered and evaporated under reduced pressure. The residue was dissolved in methanol (0.5 M) and sodium borohydride (0.40 g; 10.5 mmol; 1.5 equiv.) was added in portions at 0 °C. The mixture was stirred at room temperature for 5 h, after which time water (3.5 ml) was added. Then, roughly half of the solvent was removed under reduced pressure and CH_2Cl_2 (28 ml) was added to the residue. The organic phase was washed with a saturated aqueous solution of sodium bicarbonate (10 ml), followed by brine (10 ml). The organic phase was dried over anhydrous magnesium sulfate. The dried solution was filtered, and the filtrate was concentrated under reduced pressure to give the title product as a white oil (952 mg; 96%).

^{1}H NMR (400 MHz, $CDCl_3$): δ 5.30–5.22 (m, 1H), 3.21 (d, *J* = 6.8 Hz, 2H), 2.31 (t, *J* = 7.2 Hz, 2H), 1.71 (s, 3H), 1.65 (s, 3H), 1.54–1.43 (m, 2H), 1.40–1.28 (m, 2H), 0.91 (t, *J* = 7.3 Hz, 4H) ppm.

^{13}C NMR (150 MHz, $CDCl_3$): δ 134.2, 123.2, 49.4, 47.5, 32.4, 25.9, 20.7, 18.0, 14.1 ppm.

HRMS (ESI+): Calculated for $[M+H]^+$ $C_9H_{19}NH^+$: 142.1590, found: 142.1614.

IR (cm^{-1}): 2957, 2927, 2894, 2873, 2859, 2812, 1450, 1376, 1312, 1124, 1100, 1067, 983, 836, 780, 768, 734.

Ethyl 2-(1-hydroxycyclohexyl)-acetate[11] (9)

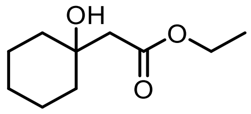

Under argon, zinc powder (6.13 g; 93.8 mmol; 1.25 equiv.) and iodine (0.38 g; 1.5 mmol; 0.02 equiv.) were added to a flame-dried round-bottom flask, after which tetrahydrofuran (75 ml) was added. The resulting suspension was heated at 70 °C, followed by the addition of ethyl bromoacetate (10.4 ml; 93.8 mmol; 1.25 equiv.) and cyclohexanone (7.77 ml, 75.0 mmol; 1.0 equiv.), which was added slowly. After 2 h of stirring at 70 °C, the reaction was heated at 85 °C for an additional 2 h. After cooling to room temperature, aq. HCl (1 M) was added and the resulting biphasic mixture was separated. The aqueous phase was extracted with diethyl ether (3 × 40 ml) and the combined organic phases were washed with water (2 × 60 ml), followed by drying over anhydrous magnesium sulfate. The dried solution was filtered, and the filtrate was concentrated under reduced pressure. The crude residue was purified by flash column chromatography (SiO_2, heptane/ethyl acetate) to afford the title compound (4.65 g, 33%).

^{1}H NMR (400 MHz, $CDCl_3$): δ 4.17 (q, *J* = 7.1 Hz, 2H), 3.41 (s, 1H), 2.46 (s, 2H), 1.75–1.60 (m, 4H), 1.59–1.49 (m, 1H), 1.49–1.36 (m, 4H), 1.28 (t, *J* = 7.0 Hz, 4H) ppm.

All analytical data were in good accordance with data reported in the literature.[11]

Ethyl 2-(cyclohex-1-en-1-yl)-acetate[11] (10)

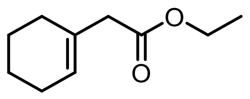

A solution of ethyl 2-(1-hydroxycyclohexyl)-acetate **9** (3.73 g, 20.0 mmol; 1.0 equiv.) and pyridine (3.24 ml; 40.0 mmol; 2.0 equiv.) in anhydrous CH_2Cl_2 (1.6 M) contained in a flame-dried flask was cooled in an ice bath. Thionyl chloride (1.62 ml; 22.0 mmol; 1.1 equiv.) was then added over a period of 5 minutes, after which the resulting solution was stirred at the same temperature for 1 h. The reaction mixture was subsequently poured into a flask containing water (100 ml), placed in an ice bath. The biphasic mixture was separated, and the aqueous phase was extracted with diethyl ether (3 × 30 ml), then the combined organic phases were washed with water (2 × 20 ml) and subsequently dried over anhydrous magnesium sulfate. The dried solution was filtered, and the filtrate was concentrated under reduced pressure. The crude residue was purified by flash column chromatography (SiO_2, heptane/ethyl acetate) to afford the title compound (2.28 g, 68%) as a colourless oil.

^{1}H NMR (400 MHz, $CDCl_3$): δ 5.61–5.53 (m, 1H), 4.13 (q, *J* = 7.0 Hz, 2H), 2.93 (s, 2H), 2.06–1.96 (m, 3H), 1.68–1.52 (m, 4H), 1.29–1.23 (m, 4H) ppm.

All analytical data were in good accordance with data reported in the literature.[11]

Ethyl penta-3,4-dienoate[6] (11)

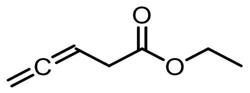

In a two-neck flask equipped with a still head (and the second neck sealed with a rubber septum), propargyl alcohol (0.29 ml; 5.0 mmol; 1.0 equiv.) and triethyl orthoacetate (1.79 g; 11.0 mmol; 2.2 equiv.) were heated to 100 °C. Then, propionic acid (7.4 µl; 0.1 mmol; 0.02 equiv.) was added and the temperature was increased to 160 °C. The formed ethanol was removed through distillation. When the ethanol release ceased, additional propargyl alcohol (0.29 ml; 5.0 mmol; 1.0 equiv.) was added over 15 min at 160 °C. The reaction was then stirred for additional 2 h at 160 °C. Propionic acid (22.5 µl; 0.3 mmol; 0.06 equiv.) was slowly added and the reaction was cooled to room temperature. Aqueous HCl (2 M; 20.0 ml) was added, the biphasic mixture was separated, and the aqueous phase was extracted with Et_2O (3 × 15 ml). The combined organic phases were washed with sat. aq. $NaHCO_3$ (1 × 15 ml) and dried over anhydrous magnesium sulfate. The dried solution was filtered, and the filtrate was concentrated under reduced pressure. The crude material was used directly in the next step.

2.3 General procedure D: Synthesis of α,β-unsaturated γ-OTMP amides

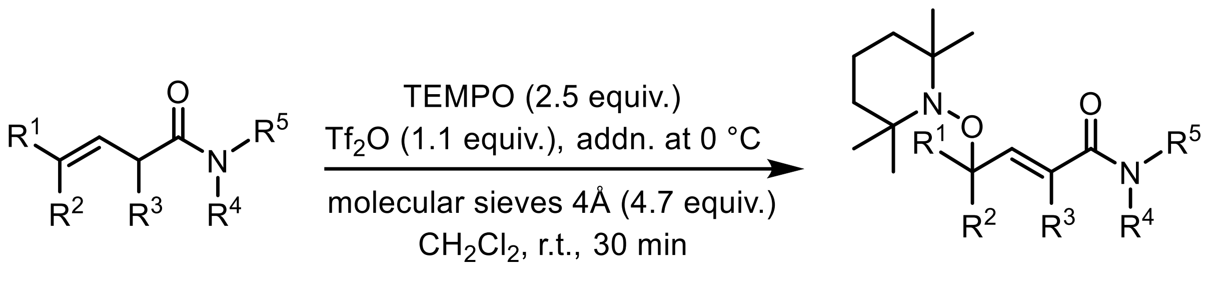

1 **2**

To a flame-dried Schlenk flask under an atmosphere of argon containing molecular sieves (4 Å, 4.7 equiv.), were added amide **1** (1.0 equiv.) and anhydrous CH_2Cl_2 (0.1 M). TEMPO (2.5 equiv.) was then added and the reaction mixture was cooled in an ice-water bath. Trifluoromethanesulfonic anhydride (Tf_2O, 1.1 equiv.) was subsequently added, the cooling bath was removed, and the reaction was stirred at r.t. for 30 min. Then, the reaction mixture was filtered, removing the molecular sieves, and added to a flask containing a saturated aqueous solution of $NaHCO_3$ (2.5 ml/mmol). The mixture was vigorously stirred at r.t. for 15 min, before the phases were separated, and the aqueous phase was extracted with CH_2Cl_2 (3 × 30 ml/mmol). The combined organic phases were dried over anhydrous magnesium sulfate. The dried solution was filtered, and the filtrate was concentrated under reduced pressure. The crude residue was purified by flash column chromatography (SiO_2, heptane/ethyl acetate) to afford the compounds **2**.

(*E*)-1-(Pyrrolidin-1-yl)-4-((2,2,6,6-tetramethylpiperidin-1-yl)-oxy)-pent-2-en-1-one (2a)

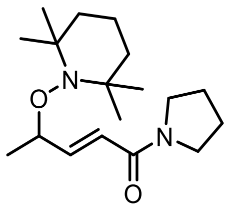

Following general procedure D using (*E*)-1-(pyrrolidin-1-yl)-pent-3-en-1-one **1a** (46.0 mg; 0.3 mmol; 1.0 equiv.), TEMPO (0.12 g; 0.75 mmol; 2.5 equiv.), 4 Å molecular sieves (0.20 g; 1.41 mmol; 4.7 equiv.) and trifluoromethanesulfonic anhydride (55.5 µl; 0.33 mmol; 1.1 equiv.). Purification by flash column chromatography (heptane/ethyl acetate 0-80%) gave the title compound as slightly orange solid (89.0 mg, 96%).

Gram-scale approach: Following general procedure D using (*E*)-1-(pyrrolidin-1-yl)-pent-3-en-1-one (1.07 g; 7.0 mmol; 1.0 equiv.), TEMPO (2.79 g; 17.5 mmol; 2.5 equiv.), 4 Å molecular sieves (4.66 g; 32.8 mmol; 4.7 equiv.) and trifluoromethanesulfonic anhydride (1.30 ml; 7.7 mmol; 1.1 equiv.). Purification by flash column chromatography (heptane/ethyl acetate 0-80%) gave the title compound as slightly orange crystals (1.96 g; 91%).

100-fold approach: Following general procedure D using (*E*)-1-(pyrrolidin-1-yl)-pent-3-en-1-one (4.6 g; 30 mmol; 1.0 equiv.), TEMPO (12 g; 75 mmol; 2.5 equiv.), 4 Å molecular sieves (20 g; 141 mmol; 4.7 equiv.) and trifluoromethanesulfonic anhydride (5.6 ml; 33 mmol; 1.1 equiv.). Purification by flash column chromatography (heptane/ethyl acetate 0-80%) gave the title compound as slightly orange crystals (7.85 g; 85%).

^{1}H NMR (400 MHz, $CDCl_3$): δ 6.89 (dd, *J* = 15.3, 6.5 Hz; 1H), 6.21 (d, *J* = 15.3 Hz, 1H), 4.50–4.40 (m, 1H), 3.38–3.47 (m, 4H), 2.01–1.92 (m, 2H), 2.91–1.82 (m, 2H), 1.61–1.37 (m, 6H), 1.28 (d, *J* = 6.7 Hz, 3H), 1.17 (br s, 3H), 1.10 (br s, 9H) ppm.

^{13}C NMR (150 MHz, $CDCl_3$): δ 164.6, 147.5, 120.0, 79.7, 59.6 (2C), 46.4, 45.7, 40.1 (2C), 34.4, 34.1, 26.1, 24.3, 20.6, 20.3 (2C), 17.2 ppm.

HRMS (ESI+): Calculated for $[M+H]^+$ $C_{18}H_{32}O_2N_2H^+$: 309.2537, found: 309.2546.

IR (cm^{-1}): 2990, 2972, 2868, 1663, 1612, 1372, 1361, 1290, 1258, 1242, 1208, 1129, 1066, 1043, 972, 957, 914, 906, 789, 764, 721, 698.

(*E*)-1-(Pyrrolidin-1-yl)-4-((2,2,6,6-tetramethylpiperidin-1-yl)-oxy)-dodec-2-en-1-one (2b)

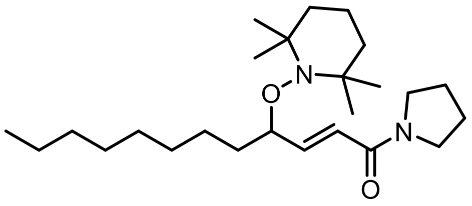

Following general procedure D using (*E*)-1-(pyrrolidin-1-yl)-tridec-3-en-1-one **1b** (75.0 mg; 0.3 mmol; 1.0 equiv.), TEMPO (0.12 g; 0.75 mmol; 2.5 equiv.), 4 Å molecular sieves (0.20 g; 1.41 mmol; 4.7 equiv.) and trifluoromethanesulfonic anhydride (55.5 µl; 0.33 mmol; 1.1 equiv.). Purification by flash column chromatography (heptane/ethyl acetate 0-80%) gave the title compound as a slightly yellow oil (82.3 mg, 67%).

^{1}H NMR (400 MHz, CDCl$_3$): δ 6.86 (dd, *J* = 15.3, 8.0 Hz, 1H), 6.14 (d, *J* = 15.3 Hz, 1H), 4.26 (dt, *J* = 7.8, 4.8 Hz, 1H), 3.71–3.37 (m, 4H), 2.04–1.80 (m, 4H), 1.63–1.36 (m, 6H), 1.34–1.20 (m, 14H), 1.20–1.04 (m, 12H), 0.87 (t, *J* = 6.3 Hz, 3H) ppm.

^{13}C NMR (150 MHz, CDCl$_3$): δ 164.6, 146.9, 121.4, 84.0, 60.1, 59.5, 46.5, 45.8, 40.2 (2C), 34.9, 34.11, 34.05, 31.9, 29.8, 29.5, 29.3, 26.1, 25.1, 24.3, 22.7, 20.4 (2C), 17.2, 14.1 ppm.

HRMS (ESI+): Calculated for $[M+H]^+$ $C_{25}H_{46}O_2N_2H^+$: 407.3632, found: 407.3633.

IR (cm^{-1}): 2924, 2856, 1663, 1621, 1417, 1375, 1360, 1337, 1133, 985, 973, 764, 749.

(*E*)-5-phenyl-1-(pyrrolidin-1-yl)-4-((2,2,6,6-tetramethylpiperidin-1-yl)-oxy)-pent-2-en-1-one (2c)

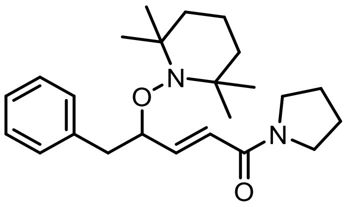

Following general procedure D using (*E*)-5-phenyl-1-(pyrrolidin-1-yl)-pent-3-en-1-one **1c** (68.8 mg; 0.3 mmol; 1.0 equiv.), TEMPO (0.12 g; 0.75 mmol; 2.5 equiv.), 4 Å molecular sieves (0.20 g; 1.41 mmol; 4.7 equiv.) and trifluoromethanesulfonic anhydride (55.5 µl; 0.33 mmol; 1.1 equiv.). Purification by flash column chromatography (heptane/ethyl acetate 0-80%) gave the title compound as a colourless oil (88.5 mg, 77%).

^{1}H NMR (400 MHz, CDCl$_3$): δ 7.31–7.26 (m, 2H), 7.22–7.16 (m, 3H), 6.85 (dd, *J* = 15.3, 8.1 Hz, 1H), 5.90 (d, *J* = 15.3 Hz, 1H), 4.52 (dt, *J* = 8.1, 5.5 Hz, 1H), 3.51 (t, *J* = 6.8 Hz, 2H), 3.45–3.37 (m, 1H), 3.32–3.20 (m, 2H), 2.73 (dd, *J* = 13.3, 8.6 Hz, 1H), 1.93–1.80 (m, 4H), 1.66–1.39 (m, 5H), 1.37–1.24 (m, 1H), 1.19 (br s, 3H), 1.16–1.09 (m, 9H) ppm.

^{13}C NMR (150 MHz, CDCl$_3$): δ 164.3, 145.1, 138.0, 129.8 (2C), 128.0 (2C), 126.0, 122.0, 84.9, 60.16, 59.7, 46.3, 45.6, 41.0, 40.2 (2C), 35.0, 34.1, 26.0, 24.2, 20.5, 20.3, 17.2 ppm.

HRMS (ESI+): Calculated for $[M+H]^+$ $C_{24}H_{36}O_2N_2H^+$: 385.2850, found: 385.2849.

IR (cm^{-1}): 2999, 2929, 2870, 1663, 1619, 1419, 1374, 1360, 1336, 1132, 1042, 1030, 988, 925, 750, 698.

(*E*)-1-(pyrrolidin-1-yl)-4-((2,2,6,6-tetramethylpiperidin-1-yl)oxy)but-2-en-1-one (2d)

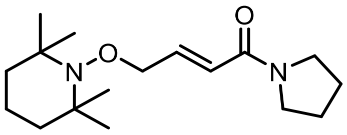

Following general procedure D using 1-(pyrrolidin-1-yl)but-3-en-1-one **1d** (0.42 g; 3 mmol; 1.0 equiv.), TEMPO (1.20 g; 7.5 mmol; 2.5 equiv.), 4 Å molecular sieves (2.0 g; 14.1 mmol; 4.7 equiv.) and trifluoromethanesulfonic anhydride (0.56 ml; 3.3 mmol; 1.1 equiv.). Purification by flash column chromatography (heptane/ethyl acetate 0-80%) gave the title compound as an orange solid (554 mg, 63%).

^{1}H NMR (400 MHz, CDCl$_3$): δ 6.88 (dt, *J* = 15.3, 4.1 Hz, 1H), 6.40 (d, *J* = 15.3 Hz, 1H), 4.51–4.44 (m, 2H), 3.60–3.50 (m, 4H), 2.02–1.83 (m, 4H), 1.66–1.42 (m, 5H), 1.37–1.27 (m, 1H), 1.17–1.09 (m, 12H) ppm.

^{13}C NMR (100 MHz, CDCl$_3$): δ 164.5, 140.4, 120.6, 76.1, 59.7 (2C), 46.4, 45.7, 39.5 (2C), 32.7 (2C), 26.0, 24.3, 20.0 (2C), 17.0 ppm.

HRMS (ESI$^+$): calculated for $[M+Na]^+$ $C_{17}H_{30}O_2N_2Na^+$: 295.2380, found 295.2370.

IR (cm^{-1}): 2972, 2931, 2870, 1665, 1620, 1425, 1359.

(*E*)-1-(Pyrrolidin-1-yl)-4-((2,2,6,6-tetramethylpiperidin-1-yl)-oxy)-penta-2,4-dien-1-one (2e)

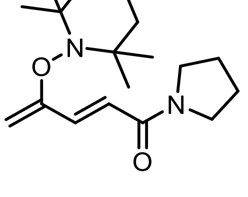

Following general procedure D using 1-(pyrrolidin-1-yl)-penta-3,4-dien-1-one **1e** (45.0 mg; 0.3 mmol; 1.0 equiv.), TEMPO (0.12 g; 0.75 mmol; 2.5 equiv.), 4 Å molecular sieves (0.20 g; 1.41 mmol; 4.7 equiv.) and trifluoromethanesulfonic anhydride (55.5 µl; 0.33 mmol; 1.1 equiv.). Purification by flash column chromatography (heptane/ethyl acetate 0-80%) gave the title compound as an orange solid (50.9 mg, 55%).

^{1}H NMR (400 MHz, CDCl$_3$: δ 7.01 (d, *J* = 15.2 Hz, 1H), 6.53 (d, *J* = 15.2 Hz, 1H), 5.16 (s, 1H), 4.50 (s, 1H), 3.57 (dt, *J* = 8.8, 7.0 Hz, 4H); 2.04–1.84 (m, 4H), 1.71–1.49 (m, 5H), 1.27–1.12 (m, 8H), 1.05–1.02 (m, 5H) ppm.

^{13}C NMR (150 MHz, CDCl$_3$): δ 164.9, 159.5, 136.3, 117.7, 97.2, 60.5 (2C), 46.6, 46.0, 39.7 (2C), 32.3 (2C), 26.2, 24.5, 20.7 (2C), 17.1 ppm.

HRMS (ESI+): Calculated for $[M+H]^+$ $C_{18}H_{30}O_2N_2H^+$: 307.2380, found: 307.2380.

IR (cm^{-1}): 2974, 2935, 2869, 1650, 1613, 1595, 1416, 1361, 1117, 1047, 1011, 946, 917, 841.

(*E*)-2-Methyl-1-(pyrrolidin-1-yl)-4-((2,2,6,6-tetramethylpiperidin-1-yl)-oxy)-pent-2-en-1-one (2f)

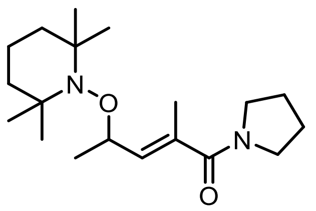

Following general procedure D using (*E*)-2-methyl-1-(pyrrolidin-1-yl)-pent-3-en-1-one **1f** (50.0 mg; 0.3 mmol; 1.0 equiv.), TEMPO (0.12 g; 0.75 mmol; 2.5 equiv.), 4 Å molecular sieves (0.20 g; 1.41 mmol; 4.7 equiv.) and trifluoromethanesulfonic anhydride (55.5 µl; 0.33 mmol; 1.1 equiv.). For this substrate, the reaction was heated to 40 °C for 2 hours. Purification by flash column chromatography (heptane/ethyl acetate 0-80%) gave the title compound as an orange oil (37.6 mg; 39%) isolated as a mixture (4:1 in favor of the title compound) with remaining starting material.

^{1}H NMR (400 MHz, CDCl$_3$): δ 5.71 (d, *J* = 8.9 Hz, 1H), 4.61 (dt, *J* = 14.9, 4.7 Hz, 1H), 3.54–3.41 (m, 4H), 1.94–1.84 (m, 4H), 1.83 (s, 3H), 1.47–1.38 (m, 4H), 1.24 (d, *J* = 6.6 Hz, 3H), 1.22–1.00 (m, 14H) ppm.

^{13}C NMR (150 MHz, CDCl$_3$): δ 171.7, 135.5, 130.9, 77.3, 59.6 (2C), 49.0, 45.7, 40.3 (2C), 34.4 (2C), 26.4, 24.5, 20.7, 20.3 (2C), 17.4, 14.2 ppm.

HRMS (ESI+): Calculated for $[M+Na]^+$ $C_{19}H_{34}O_2N_2Na^+$: 345.2512, found: 345.2508.

IR (cm^{-1}): 2972, 2929, 2873, 1617, 1420, 1374, 1360, 1260, 1133, 1043, 956, 923, 750, 728, 674, 644.

(*E*)-*N*-Cyclopropyl-*N*,2-dimethyl-4-((2,2,6,6-tetramethylpiperidin-1-yl)oxy)pent-2-enamide (2g)

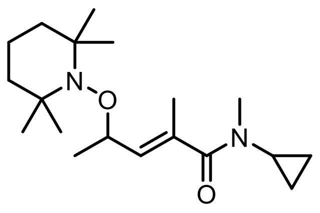

Following general procedure D using (*E*)-*N*-cyclopropyl-*N*,2-dimethylpent-3-enamide **1g** (50.0 mg; 0.3 mmol; 1.0 equiv.), TEMPO (0.12 g; 0.75 mmol; 2.5 equiv.), 4 Å molecular sieves (0.20 g; 1.41 mmol; 4.7 equiv.) and trifluoromethanesulfonic anhydride (55.5 µl; 0.33 mmol; 1.1 equiv.). For this substrate, the reaction was heated to 40 °C for 2 h. Purification by flash column chromatography (heptane/ethyl acetate 0-80%) gave the title compound as a slightly orange oil (40.0 mg, 41%).

^{1}H NMR (400 MHz, CDCl$_3$) δ 5.68 (dd, *J* = 8.8, 1.2 Hz, 1H), 4.61 (dq, *J* = 13.1, 6.5 Hz, 1H), 2.94 (s, 3H), 2.76–2.68 (m, 1H), 1.82 (d, *J* = 1.3 Hz, 3H), 1.57–1.48 (m, 1H), 1.46–1.38 (m, *J* = 4.5 Hz, 4H), 1.32–1.28 (m, 1H), 1.24 (d, *J* = 6.5 Hz, 3H), 1.21–1.14 (m, 3H), 1.14–1.02 (m, 9H), 0.79–0.72 (m, 2H), 0.62–0.57 (m, 2H) ppm.

^{13}C NMR (101 MHz, CDCl$_3$) δ 174.8, 135.4, 130.6, 77.0, 59.7, 59.5, 40.2, 34.9, 34.4, 34.3, 32.3, 20.39, 20.35, 20.27, 17.3 (2C), 14.4, 8.9, 8.5 ppm.

HRMS (ESI) (m/z): calculated for $[M+H]^+$ $C_{19}H_{34}O_2N_2H^+$ requires 323.2693, found 323.2695.

IR (cm^{-1}): 2928, 1630, 1453, 1359, 1132, 1043, 955, 709.

(*E*)-1-(Pyrrolidin-1-yl)-2-(2-((2,2,6,6-tetramethylpiperidin-1-yl)-oxy)-cyclohexylidene)-ethan-1-one (2h)

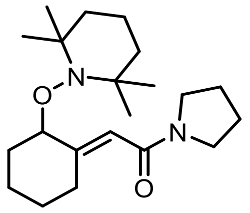

Following general procedure D using 2-(cyclohex-1-en-1-yl)-1-(pyrrolidin-1-yl)-ethan-1-one **1h** (58.0 mg; 0.3 mmol; 1.0 equiv.), TEMPO (0.12 g; 0.75 mmol; 2.5 equiv.), 4 Å molecular sieves (0.20 g; 1.41 mmol; 4.7 equiv.) and trifluoromethanesulfonic anhydride (55.5 µl; 0.33 mmol; 1.1 equiv.). Purification by flash column chromatography (heptane/ethyl acetate 0-80%) gave the title compound as a slightly yellow oil (97.6 mg, 93%).

^{1}H NMR (600 MHz, CDCl$_3$): δ 6.10 (s, 1H), 4.09 (dd, *J* = 8.4, 3.7 Hz, 1H), 3.50 (t, *J* = 6.8 Hz, 2H), 3.49 (dt, *J* = 6.7, 3.4 Hz, 2H), 3.15–3.06 (m, 1H), 2.32–2.23 (m, 1H), 2.20–2.12 (m, 1H), 1.97–1.91 (m, 2H), 1.88–1.82 (m, 2H), 1.82–1.75 (m, 1H), 1.70–1.37 (m, 9H), 1.37–1.22 (m, 1H), 1.16 (s, 6H), 1.10 (s, 6H) ppm.

^{13}C NMR (150 MHz, CDCl$_3$): δ 166.8, 155.3, 114.5, 85.4, 60.4, 59.5, 47.1, 45.5, 40.4 (2C), 35.5, 34.7, 34.0, 29.1, 28.1, 26.3, 24.5, 24.0, 20.6 (2C), 17.3 ppm.

HRMS (ESI+): Calculated for $[M+H]^+$ $C_{21}H_{36}O_2N_2H^+$: 349.2850, found: 349.2842.

IR (cm^{-1}): 2973, 2928, 2868, 1654, 1612, 1421, 1360, 1132, 957, 922, 728.

Methyl (*E*)-12-oxo-12-(pyrrolidin-1-yl)-9-((2,2,6,6-tetramethylpiperidin-1-yl)-oxy)-dodec-10-enoate (2i)

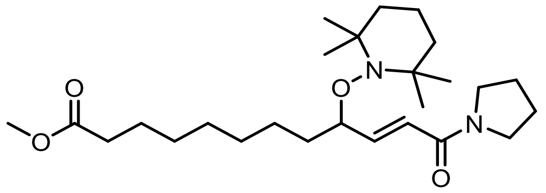

Following general procedure D using methyl (*E*)-12-oxo-12-(pyrrolidin-1-yl)-dodec-9-enoate **1i** (89.0 mg, 0.3 mmol; 1 equiv.), TEMPO (0.12 g; 0.75 mmol; 2.5 equiv.), 4 Å molecular sieves (0.20 g; 1.41 mmol; 4.7 equiv.) and trifluoromethanesulfonic anhydride (55.5 µl; 0.33 mmol; 1.1 equiv.). Purification by flash column chromatography (heptane/ethyl acetate 0-80%) gave the title compound as a slightly yellow oil (106 mg, 78%).

^{1}H NMR (400 MHz, $CDCl_3$): δ 6.85 (dd, *J* = 15.3, 8.0 Hz, 1H), 6.14 (d, *J* = 15.3 Hz, 1H), 4.25 (dt, *J* = 7.8, 4.7 Hz, 1H), 3.65 (s, 3H), 3.57–3.47 (m, 4H), 2.28 (t, *J* = 7.5 Hz, 2H), 2.01–1.91 (m, 2H), 1.90–1.81 (m, 2H), 1.64–1.49 (m, 4H), 1.47–1.36 (m, 4H), 1.33–1.20 (m, 10H), 1.18–1.02 (m, 12H) ppm.

^{13}C NMR (150 MHz, $CDCl_3$): δ 174.3, 164.5, 146.8, 121.5, 84.0, 60.1, 59.6, 51.4, 46.5, 45.8, 40.2 (2C), 34.9, 34.14, 34.09, 34.0, 29.6, 29.2, 29.1, 26.1, 25.0, 24.9, 24.3, 20.42, 20.37, 17.3 ppm.

HRMS (ESI+): Calculated for $[M+H]^+$ $C_{26}H_{46}O_4N_2H^+$: 451.3530, found: 451.3532.

IR (cm^{-1}): 2928, 2856, 1737, 1663, 1620, 1418, 1374, 1360, 1337, 1170, 1133, 983.

(*E*)-1-(Pyrrolidin-1-yl)-4-((2,2,6,6-tetramethylpiperidin-1-yl)oxy)dodec-2-ene-1,11-dione (2j)

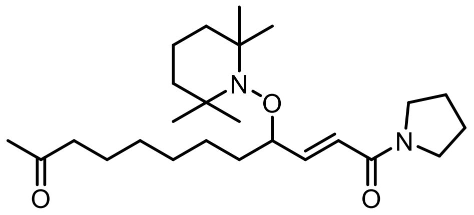

Following general procedure D using (*E*)-1-(Pyrrolidin-1-yl)dodec-3-ene-1,11-dione **1j** (79.6 mg; 0.3 mmol; 1.0 equiv.), TEMPO (0.12 g; 0.75 mmol; 2.5 equiv.), 4 Å molecular sieves (0.20 g; 1.41 mmol; 4.7 equiv.) and trifluoromethanesulfonic anhydride (55.5 µl; 0.33 mmol; 1.1 equiv.). Purification by flash column chromatography (heptane/ethyl acetate 0-80%) gave the title compound as a slightly yellow oil (85.4 mg, 68%).

^{1}H NMR (400 MHz, $CDCl_3$): δ 6.85 (dd, *J* = 15.3, 7.9 Hz, 1H), 6.14 (d, *J* = 15.3 Hz, 1H), 4.30–4.21 (m, 1H), 3.60–3.46 (m, 4H), 2.40 (t, *J* = 7.3 Hz, 2H), 2.21 (s, 3H), 2.01–1.96 (m, 2H), 1.91–1.81 (m, 2H), 1.79–1.67 (m, 1H), 1.62–1.79 (m, 4H), 1.48–1.36 (m, 4H), 1.34–1.21 (m, 7H), 1.19–1.02 (m, 12H) ppm.

^{13}C NMR (150 MHz, $CDCl_3$): δ 209.3, 164.5, 146.8, 121.5, 83.9, 60.1, 59.6, 46.6, 45.8, 43.8, 40.2 (2C), 34.9, 34.1, 34.0, 29.9, 29.5, 29.1, 26.2, 24.9, 24.3, 23.8, 20.4 (2C), 17.3 ppm.

HRMS (ESI+): Calculated for $[M+H]^+$ $C_{25}H_{44}O_3N_2H^+$: 421.3425, found: 421.3426.

IR (cm^{-1}): 2973, 2931, 2870, 1714, 1661, 1613, 1428, 1359, 915, 726, 645.

(*E*)-9-Oxo-9-(pyrrolidin-1-yl)-6-((2,2,6,6-tetramethylpiperidin-1-yl)-oxy)-non-7-enenitrile (2k)

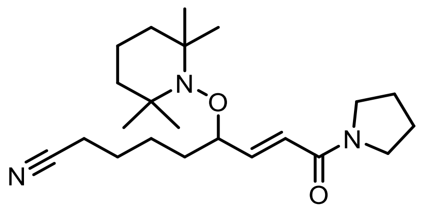

Following general procedure D using (*E*)-9-oxo-9-(pyrrolidin-1-yl)-non-6-enenitrile **1k** (71 mg; 0.35 mmol; 1 equiv.), TEMPO (0.14 g; 0.875 mmol; 2.5 equiv.), 4 Å molecular sieves (0.20 g; 1.41 mmol; 4.7 equiv.) and trifluoromethanesulfonic anhydride (64.8 µl; 0.385 mmol; 1.1 equiv.). Purification by flash column chromatography (heptane/ethyl acetate 0-80%) gave the title compound as a slightly yellow oil (76.8 mg, 58%).

^{1}H NMR (400 MHz, $CDCl_3$): δ 6.84 (dd, *J* = 15.3, 7.9 Hz, 1H), 6.17 (d, *J* = 15.3, 1H), 4.31 (app q, *J* = 5.4 Hz, 1H), 3.61–3.48 (m, 4H), 2.33 (t, *J* = 6.9 Hz, 2H), 2.02–1.82 (m, 4H), 1.82–1.36 (m, 12H), 1.20–1.02 (m, 12H) ppm.

^{13}C NMR (150 MHz, $CDCl_3$): δ 164.2, 145.9, 122.0, 119.6, 83.3, 60.1, 59.6, 46.5, 45.8, 40.1 (2C), 34.7, 34.1, 33.2, 26.1, 25.5, 24.24, 24.16, 20.3 (2C), 17.13, 17.10 ppm.

HRMS (ESI+): Calculated for $[M+H]^+$ $C_{22}H_{37}O_2N_3H^+$: 376.2959, found: 376.2961.

IR (cm^{-1}): 2893, 2868, 1662, 1613, 1422, 1375, 1360, 1133, 1081, 1044, 1010, 985, 917, 730.

(*E*)-8-Chloro-1-(pyrrolidin-1-yl)-4-((2,2,6,6-tetramethylpiperidin-1-yl)-oxy)-oct-2-en-1-one (2l)

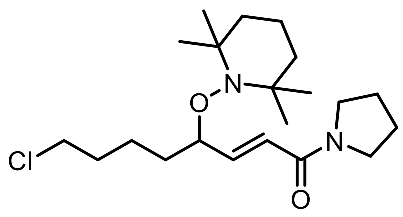

Following general procedure D using (*E*)-8-chloro-1-(pyrrolidin-1-yl)-oct-3-en-1-one **1l** (68.9 mg; 0.3 mmol; 1 equiv.), TEMPO (0.12 g; 0.75 mmol; 2.5 equiv.), 4 Å molecular sieves (0.20 g; 1.41 mmol; 4.7 equiv.) and trifluoromethanesulfonic anhydride (55.5 µl; 0.33 mmol; 1.1 equiv.). Purification by flash column chromatography (heptane/ethyl acetate 0-80%) gave the title compound as a slightly yellow oil (81.3 mg, 70%).

^{1}H NMR (400 MHz, CDCl$_3$): δ 6.85 (dd, *J* = 15.3, 7.9 Hz, 1H), 6.17 (d, *J* = 15.3, 1H), 4.30 (dt, *J* = 7.6, 4.9 Hz, 1H), 3.60–3.46 (m, 6H), 2.02–1.92 (m, 2H), 1.91–1.82 (m, 2H), 1.82–1.71 (m, 3H), 1.66–1.37 (m, 8H), 1.34–1.24 (m, 13H) ppm.

^{13}C NMR (150 MHz, CDCl$_3$): δ 164.4, 146.3, 121.9, 83.6, 60.1, 59.6, 46.6, 45.8, 45.0, 40.3 (2C), 34.9, 34.2, 33.3, 32.8, 26.2, 24.4, 22.5, 20.4 (2C), 17.2 ppm.

HRMS (ESI+): Calculated for $[M+H]^+$ $C_{21}H_{37}O_2N_2ClH^+$: 385.2616, found: 385.2615.

IR (cm^{-1}): 2931, 2868, 1663, 1618, 1418, 1374, 1360, 1132, 985, 748.

(*E*)-1-(Pyrrolidin-1-yl)-4-((2,2,6,6-tetramethylpiperidin-1-yl)-oxy)-dodec-2-en-11-yn-1-one (2m)

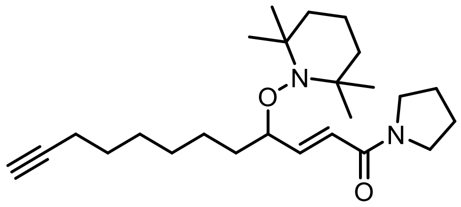

Following general procedure D using (*E*)-1-(pyrrolidin-1-yl)-dodec-3-en-11-yn-1-one **1m** (74.2 mg; 0.3 mmol; 1.0 equiv.), TEMPO (0.12 g; 0.75 mmol; 2.5 equiv.), 4 Å molecular sieves (0.20 g; 1.41 mmol; 4.7 equiv.) and trifluoromethanesulfonic anhydride (55.5 µl; 0.33 mmol; 1.1 equiv.). Purification by flash column chromatography (heptane/ethyl acetate 0-80%) gave the title compound as a slightly yellow oil (95.2 mg, 79%).

^{1}H NMR (400 MHz, CDCl$_3$): δ 6.85 (dd, *J* = 15.3, 7.8 Hz, 1H), 6.14 (d, *J* = 15.3 Hz, 1H), 4.26 (app q, *J* = 5.0 Hz, 1H), 3.63–3.50 (m, 4H), 2.16 (t, *J* = 6.5 Hz, 2H), 2.01–1.78 (m, 5H), 1.62–1.22 (m, 16H), 1.19–1.02 (m, 12H) ppm.

^{13}C NMR (150 MHz, CDCl$_3$): δ 164.6, 146.8, 121.5, 84.8, 84.0, 68.1, 60.1, 59.6, 46.6, 45.8, 40.3 (2C), 34.9, 34.2, 34.0, 29.3, 28.7, 28.5, 26.2, 24.9, 24.4, 20.4 (2C), 18.4, 17.3 ppm.

HRMS (ESI+): Calculated for $[M+H]^+$ $C_{25}H_{42}O_2N_2H^+$: 403.3319, found: 403.3320.

IR (cm^{-1}): 3310, 3231, 3003, 2969, 2859, 1663, 1618, 1419, 1374, 1360, 1132, 988, 957, 915, 628.

(5*R*,*E*)-5,9-Dimethyl-1-(pyrrolidin-1-yl)-4-((2,2,6,6-tetramethylpiperidin-1-yl)-oxy)-deca-2,8-dien-1-one (2n)

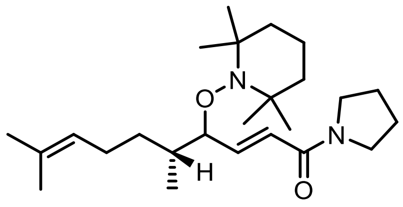

Following general procedure D using (*R*,*E*)-5,9-dimethyl-1-(pyrrolidin-1-yl)-deca-3,8-dien-1-one **1n** (74.8 mg; 0.3 mmol; 1.0 equiv.), TEMPO (0.12 g; 0.75 mmol; 2.5 equiv.), 4 Å molecular sieves (0.20 g; 1.41 mmol; 4.7 equiv.) and trifluoromethanesulfonic anhydride (55.5 µl; 0.33 mmol; 1.1 equiv.). Purification by flash column chromatography (heptane/ethyl acetate 0-80%) gave the title compound as a slightly yellow oil (108 mg, 89%, d.r. 1.5/1).

^{1}H NMR (400 MHz, CDCl$_3$):

Major: δ 6.90 (dd, *J* = 13.8, 8.3 Hz, 1H), 6.16 (d, *J* = 15.2 Hz, 1H), 5.10 (app q, *J* = 7.5, 1H), 4.24–4.15 (m, 1H), 3.62–3.44 (m, 4H), 2.12–1.79 (m, 8H), 1.67 (s, 3H), 1.60 (s, 3H), 1.47–1.22 (m, 7H), 1.21–1.02 (m, 12H), 0.91 (d, *J* = 6.7 Hz, 3H) ppm.

Minor: δ 6.93 (dd, *J* = 15.2, 8.6 Hz, 1H), 6.12 (d, *J* = 15.0 Hz, 1H), 5.10 (app q, *J* = 7.5, 1H), 4.18–4.09 (m, 1H), 3.62–3.44 (m, 4H), 2.12–1.79 (m, 8H), 1.67 (s, 3H), 1.60 (s, 3H), 1.47–1.22 (m, 7H), 1.21–1.02 (m, 12H), 0.91 (d, *J* = 6.7 Hz, 3H) ppm.

^{13}C NMR (150 MHz, CDCl$_3$):

Major: δ 164.4, 144.2, 131.3, 124.6, 122.9, 87.0, 60.1, 59.7, 46.5, 45.7, 40.3 (2C), 36.4, 34.8, 34.2, 33.6, 25.9, 25.70, 25.68, 24.3, 20.4 (2C), 17.2, 16.1, 14.4 ppm.

Minor: δ 164.4, 144.8, 131.2, 124.8, 122.8, 87.8, 60.1, 59.7, 46.5, 45.7, 40.3 (2C), 36.9, 34.8, 34.2, 31.7, 26.1, 25.9, 25.68, 24.3, 20.4 (2C), 17.2, 16.1, 14.4 ppm.

HRMS (ESI+): Calculated for $[M+H]^+$ $C_{25}H_{44}O_2N_2H^+$: 405.3476, found: 405.3467.

IR (cm^{-1}): 2967, 2930, 2893, 1663, 1620, 1417, 1375, 1360, 1133, 984, 750.

(*E*)-1-(Azepan-1-yl)-4-((2,2,6,6-tetramethylpiperidin-1-yl)-oxy)-pent-2-en-1-one (2p)

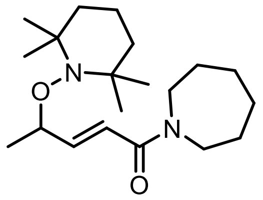

Following general procedure D using (*E*)-1-(azepan-1-yl)-pent-3-en-1-one **1p** (54.0 mg; 0.3 mmol; 1.0 equiv.), TEMPO (0.12 g; 0.75 mmol; 2.5 equiv), 4 Å molecular sieves (0.20 g; 1.41 mmol; 4.7 equiv.) and trifluoromethanesulfonic anhydride (55.5 µl; 0.33 mmol; 1.1 equiv.). The reaction was quenched with water, instead of aqueous sodium bicarbonate. The crude residue was dissolved in CH_2Cl_2 (1 ml) and was stirred with silica gel (0.25 g) for 15 min. Purification by flash column chromatography (heptane/ethyl acetate 0-80%, dry load) gave the title compound as a slightly yellow oil (51.6 mg, 51%).

^{1}H NMR (400 MHz, $CDCl_3$): δ 6.87 (dd, *J* = 15.3, 6.7 Hz, 1H), 6.36 (d, *J* = 15.3 Hz, 1H), 4.49–4.40 (m, 1H), 3.65–3.45 (m, 4H), 1.81–1.67 (m, 4H), 1.64–1.51 (m, 6H), 1.49–1.39 (m, 4H), 1.30 (d, *J* = 6.7 Hz, 3H), 1.18 (br s, 3H), 1.10 (br s, 9H) ppm.

^{13}C NMR (150 MHz, $CDCl_3$): δ 166.4, 147.9, 119.2, 79.9, 59.8 (2C), 47.9, 46.4, 40.3 (2C), 34.6, 34.4, 29.4, 27.7, 27.1, 26.6, 20.7, 20.4 (2C), 17.3 ppm.

HRMS (ESI$^+$): Calculated for $[M+H]^+$ $C_{20}H_{36}O_2N_2H^+$: 337.2850, found: 337.2852.

IR (cm^{-1}): 2971, 2926, 2857, 1661, 1617, 1448, 1423, 1374, 1360, 1275, 1259, 1132, 1043, 956, 729.

(*E*)-1-(Azetidin-1-yl)-4-((2,2,6,6-tetramethylpiperidin-1-yl)-oxy)-pent-2-en-1-one (2q)

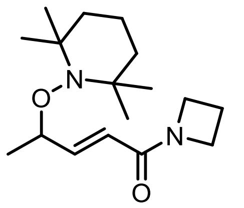

Following general procedure D using (*E*)-1-(azetidin-1-yl)-pent-3-en-1-one **1q** (41.8 mg; 0.3 mmol; 1.0 equiv.), TEMPO (0.12 g; 0.75 mmol; 2.5 equiv.), 4 Å molecular sieves (0.20 g; 1.41 mmol; 4.7 equiv.) and trifluoromethanesulfonic anhydride (55.5 µl; 0.33 mmol; 1.1 equiv.). Purification by flash column chromatography (heptane/ethyl acetate 0-80%) gave the title compound as a slightly yellow oil (73.6 mg, 83%).

^{1}H NMR (600 MHz, $CDCl_3$): δ 6.85 (dd, *J* = 15.4, 6.7 Hz, 1H), 5.95 (d, *J* = 15.4, 1.1 Hz, 1H), 4.45–4.39 (m, 1H), 4.22 (app q, *J* =7.4 Hz, 2H), 4.09 (t, *J* = 7.7 Hz, 2H), 2.35–2.24 (m, 2H), 1.48–1.38 (m, 4H), 1.35–1.30 (m, 1H), 1.28 (d, *J* = 6.7 Hz, 3H), 1.20–1.04 (m, 13H) ppm.

^{13}C NMR (150 MHz, $CDCl_3$): δ 166.0, 147.3, 117.0, 79.8, 59.9, 59.7, 50.1, 47.9, 40.2 (2C), 34.6, 34.3, 20.7, 20.4, 20.3, 17.3, 15.3 ppm.

HRMS (ESI$^+$): Calculated for $[M+H]^+$ $C_{17}H_{30}O_2N_2H^+$: 295.2380, found: 295.2376.

IR (cm^{-1}): 2974, 2927, 1667, 1621, 1454, 1434, 1381, 1372, 1129, 1068, 957, 790, 764, 751, 710.

(*E*)-*N*,*N*-Dimethyl-4-((2,2,6,6-tetramethylpiperidin-1-yl)oxy)pent-2-enamide (2r)

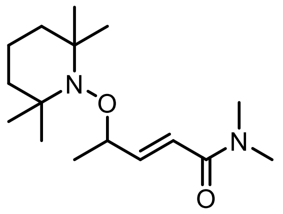

Following general procedure D using (*E*)-*N*,*N*-dimethylpent-3-en-amide **1r** (25.4 mg; 0.2 mmol; 1.0 equiv.), TEMPO (80 mg; 0.5 mmol; 2.5 equiv.), 4 Å molecular sieves (0.13 g; 0.94 mmol; 4.7 equiv.) and trifluoromethanesulfonic anhydride (37.0 µl; 0.22 mmol; 1.1 equiv.). Purification by flash column chromatography (heptane/ethyl acetate 0-45%) to give the title compound as an orange oil (31.2 mg; 55%).

^{1}H NMR (600 MHz, $CDCl_3$): δ 6.85 (dd, *J* = 15.1, 6.5 Hz, 1H), 6.37 (d, *J* = 14.2 Hz, 1H), 4.48–4.41 (m, 1H), 3.09 (s, 3H), 3.01 (s, 3H), 1.50–1.24 (m, 6H), 1.30 (d, *J* = 6.3 Hz, 3H), 1.17 (s, 3H), 1.10 (br s, 9H) ppm.

^{13}C NMR (150 MHz, $CDCl_3$): δ 166.8, 148.2, 118.8, 79.9, 60.0, 40.3 (2C), 37.5, 35.8, 34.7, 34.4, 20.8, 20.5, 20.4, 17.4 (2C) ppm.

HRMS (ESI$^+$): Calculated for $[M+Na]^+$ $C_{16}H_{30}O_2N_2Na^+$: 305.2199, found: 305.2198.

IR (cm^{-1}): 2970, 2928, 1658, 1622, 1459, 1390, 1256, 1130, 1044, 949, 852, 788, 627.

(*E*)-1-Morpholino-4-((2,2,6,6-tetramethylpiperidin-1-yl)oxy)pent-2-en-1-one (2s)

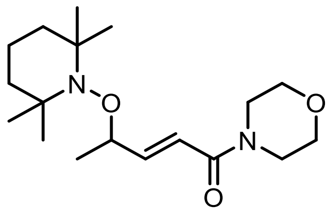

Following general procedure D using (*E*)-1-morpholinopent-3-en-1-one **1s** (33.8 mg; 0.2 mmol; 1.0 equiv.), TEMPO (80 mg; 0.5 mmol; 2.5 equiv.), 4 Å molecular sieves (0.13 g; 0.94 mmol; 4.7 equiv.) and trifluoromethanesulfonic anhydride (37.0 µl; 0.22 mmol; 1.1 equiv.). Purification by flash column chromatography (heptane/ethyl acetate 0-45%) to give the title compound as an orange oil (45.8 mg; 71%).

^{1}H NMR (600 MHz, CDCl$_3$): δ 6.86 (dd, *J* = 15.2, 6.9 Hz, 1H), 6.29 (dd, *J* = 15.2, 1.0 Hz, 1H), 4.42 (app pd, *J* = 6.6, 0.8 Hz, 1H), 3.75–3.48 (m, 8H), 1.46–1.22 (m, 6H), 1.27 (d, *J* = 6.6 Hz, 3H), 1.20–1.04 (m, 12H) ppm.

^{13}C NMR (151 MHz, CDCl$_3$): δ 165.5, 148.9, 117.9, 79.7, 66.8 (3C), 59.7, 46.1, 42.2, 40.1 (2C), 34.6, 34.2, 20.4, 20.3, 20.2, 17.1 ppm.

HRMS (ESI$^+$): Calculated for $[M+Na]^+$ $C_{18}H_{32}O_3N_2Na^+$: 347.2305, found: 347.2303.

IR (cm^{-1}): 2969, 2925, 2860, 1655, 1621, 1429, 1364, 1254, 1042, 952, 789, 705.

(*E*)-*N*,*N*-Dibenzyl-4-((2,2,6,6-tetramethylpiperidin-1-yl)oxy)pent-2-en-amide (2t)

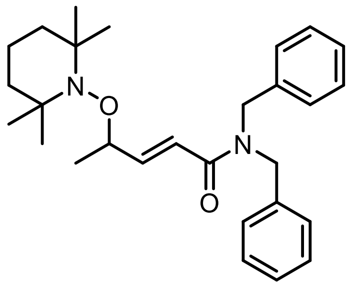

Following general procedure D using (*E*)-*N*,*N*-dibenzylpent-3-en-amide **1t** (56.0 mg; 0.2 mmol; 1.0 equiv.), TEMPO (80 mg; 0.5 mmol; 2.5 equiv.), 4 Å molecular sieves (0.13 g; 0.94 mmol; 4.7 equiv.) and trifluoromethanesulfonic anhydride (37.0 µl; 0.22 mmol; 1.1 equiv.). Purification by flash column chromatography (heptane/ethyl acetate 0-20%) to give the title compound as an orange oil (55.1 mg; 63%).

^{1}H NMR (600 MHz, CDCl$_3$): δ 7.38–7.26 (m, 8H), 7.19 (d, *J* = 7.4 Hz, 2H), 7.04 (dd, *J* = 15.1, 6.6 Hz, 1H), 6.42 (dd, *J* = 15.1, 0.9 Hz, 1H), 4.74–4.48 (m, 4H), 4.46–4.40 (m, 1H), 1.57–1.25 (m, 6H), 1.28 (d, *J* = 6.6 Hz, 3H), 1.17–0.94 (m, 12H) ppm.

^{13}C NMR (151 MHz, CDCl$_3$): δ 167.3, 149.6, 137.5, 136.8, 128.9 (2C), 128.6 (2C), 128.4 (2C), 127.6, 127.4, 126.6 (2C), 118.7, 79.7, 59.8, 59.7, 50.0, 48.6, 40.2 (2C), 34.5, 34.3, 20.6, 20.3 (2C), 17.2.

HRMS (ESI$^+$): Calculated for $[M+H]^+$ $C_{28}H_{38}O_2N_2H^+$: 435.3006, found: 435.3009.

IR (cm^{-1}): 2970, 2926, 1809, 1656, 1622, 1422, 1253, 1198, 1131, 1038, 948, 846.

(*E*)-*N*-Butyl-*N*-(3-methylbut-2-en-1-yl)-4-((2,2,6,6-tetramethylpiperidin-1-yl)-oxy)-pent-2-enamide (2u)

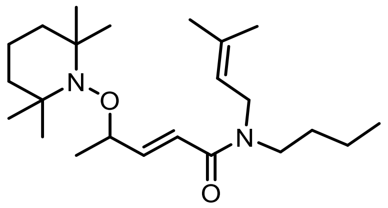

Following the general procedure, using (*E*)-*N*-butyl-*N*-(3-methylbut-2-en-1-yl)-pent-3-enamide **1u** (67.0 mg; 0.3 mmol; 1.0 equiv.), TEMPO (0.12 g; 0.75 mmol; 2.5 equiv.), 4 Å molecular sieves (0.20 g; 1.41 mmol; 4.7 equiv.) and trifluoromethanesulfonic anhydride (55.5 µl; 0.33 mmol; 1.1 equiv.). Purification by flash column chromatography (heptane/ethyl acetate 0-80%) gave the title compound as a slightly yellow oil (86.0 mg; 76%).

2 rotamers are present in the NMR (1:0.9, only differentiable in the ^{13}C-spectrum).

^{1}H NMR (400 MHz, CDCl$_3$):

δ 6.87 (ddd, *J* = 15.2, 11.3, 6.2 Hz, 1H), 6.32 (dd, *J* = 27.6, 15.2 Hz, 1H), 5.21–5.10 (m, 1H), 4.51–4.38 (m, 1H), 4.09–4.01 (m, 1H),), 3.98–3.90 (m, 1H) 3.38 (t, *J* = 7.8 Hz, 1H), 3.33–3.24 (m, 1H), 1.75–1.66 (m, 6H), 1.63–1.39 (m, 7H), 1.38–1.24 (m, 6H), 1.20–1.05 (m, 12H), 0.97–0.89 (m, 3H) ppm.

^{13}C NMR (150 MHz, CDCl$_3$):

Major: δ 165.7, 147.5, 135.0, 120.0, 118.5, 79.2, 59.5, 59.2, 45.6, 43.1, 39.8 (2C), 34.0 (2C), 31.2, 29.5, 25.3, 20.3, 20.2, 19.7, 17.4, 16.9, 13.4 ppm

Minor: δ 165.7, 147.3, 135.0, 120.8, 119.1, 79.3, 59.5, 59.2, 46.7, 45.7, 39.8 (2C), 34.0 (2C), 31.2, 29.5, 25.2, 20.3, 20.2, 20.0, 17.6, 16.9, 13.5 ppm

HRMS (ESI+): Calculated for $[M+H]^+$ $C_{23}H_{42}O_2N_2H^+$: 379.3319, found: 379.3329.

IR (cm^{-1}): 2967, 2928, 2873, 1661, 1622, 1440, 1422, 1375, 1360, 1296, 1260, 1132, 1044, 956, 750.

(*E*)-*N*-Allyl-*N*-methyl-4-((2,2,6,6-tetramethylpiperidin-1-yl)oxy)pent-2-en-amide (2v)

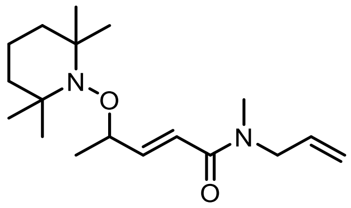

Following general procedure D using (*E*)-*N*-allyl-*N*-methylpent-3-en-amide **1v** (30.6 mg; 0.2 mmol; 1.0 equiv.), TEMPO (80 mg; 0.5 mmol; 2.5 equiv.), 4 Å molecular sieves (0.13 g; 0.94 mmol; 4.7 equiv.) and trifluoromethanesulfonic anhydride (37.0 µl; 0.22 mmol; 1.1 equiv.). Purification by flash column chromatography (heptane/ethyl acetate 0-50%) to give the title compound as an orange oil (44.3 mg; 72%).

2 rotamers are present in the NMR (1:0.8).

^{1}H NMR (400 MHz, CDCl$_3$):

Major: δ 6.94–6.80 (m, 1H), 6.27 (d, *J* = 15.2 Hz, 1H), 5.84–5.72 (m, 1H), 5.28–5.08 (m, 2H), 4.51–4.38 (m, 1H), 4.10–3.88 (m, 2H), 2.99 (s, 3H), 1.59–1.37 (m, 6H), 1.28 (d, *J* = 6.7 Hz, 3H), 1.22–1.02 (m, 12H) ppm.

Minor: δ 6.94–6.80 (m, 1H), 6.39 (d, *J* = 15.2 Hz, 1H), 5.84–5.72 (m, 1H), 5.28–5.08 (m, 2H), 4.51–4.38 (m, 1H), 4.10–3.88 (m, 2H), 3.03 (s, 3H), 1.59–1.37 (m, 6H), 1.30 (d, *J* = 6.7 Hz, 3H), 1.22–1.02 (m, 12H) ppm.

^{13}C NMR (150 MHz, CDCl$_3$):

Major: δ 167.2, 148.7, 133.2, 118.9, 117.4, 79.9, 60.0 (2C), 52.4, 40.3 (2C), 34.9, 34.7, 34.4, 20.7, 20.5, 20.4, 17.4 ppm.

Minor: δ 166.5, 148.2, 132.8, 118.8, 116.9, 79.9, 60.0 (2C), 50.4, 40.3 (2C), 34.1, 34.7, 34.4, 20.7, 20.5, 20.4, 17.4 ppm.

HRMS (ESI$^+$): Calculated for $[M+Na]^+$ $C_{18}H_{32}O_2N_2Na^+$: 331.2356, found: 331.2351.

IR (cm^{-1}): 2971, 2928, 2349, 2112, 2018, 1658, 1624, 1458, 1395, 1257, 1207, 1130, 1081, 1043, 941, 851, 788, 754, 709.

(*E*)-*N*-Cyclopropyl-*N*-methyl-4-((2,2,6,6-tetramethylpiperidin-1-yl)oxy)pent-2-enamide (2w)

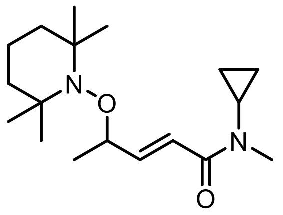

Following general procedure D using (*E*)-*N*-cyclopropyl-*N*-methylpent-3-enamide **1w** (27.0 mg; 0.18 mmol; 1.0 equiv.), TEMPO (70:2 mg; 0.44 mmol; 2.5 equiv.), 4 Å molecular sieves (0.12 g; 0.825 mmol; 4.7 equiv.) and trifluoromethanesulfonic anhydride (32.6 µl; 0.19 mmol; 1.1 equiv.). Purification by flash column chromatography (heptane/ethyl acetate 0-50%) to give the title compound as an orange oil (40.0 mg; 74%).

^{1}H NMR (600 MHz, CDCl$_3$) δ 6.88 (dd, *J* = 15.3, 6.4 Hz, 1H), 6.73 (d, *J* = 15.3 Hz, 1H), 4.48—4.45 (m, 1H), 2.98 (s, 3H), 2.76–2.71 (m, 1H), 1.56 (s, 1H), 1.50–1.40 (m, *J* = 11.7 Hz, 4H), 1.34–1.31 (m, *J* = 6.6 Hz, 1H), 1.30 (d, *J* = 6.6 Hz, 3H), 1.20–1.16 (m, 3H), 1.13–1.08 (m, *J* = 8.2 Hz, 9H), 0.89 (d, *J* = 6.7 Hz, 2H), 0.80–0.71 (m, *J* = 9.3 Hz, 2H) ppm.

^{13}C NMR (151 MHz, CDCl$_3$) δ 168.4, 147.5, 120.1, 79.6, 59.9, 59.6, 40.2 (2C), 34.5, 34.4, 34.2, 30.8, 20.6, 20.4, 20.3, 17.2, 9.32, 9.28 ppm.

HRMS (ESI+): Calculated for $[M+Na]^+$ $C_{18}H_{32}O_2N_2Na^+$: 331.2356, found: 331.2361

IR (cm^{-1}): 2972, 1662, 628, 1376, 1360, 1131, 975, 708

(*E*)-*N*-(Cyclopropylmethyl)-*N*-methyl-4-((2,2,6,6-tetramethylpiperidin-1-yl)oxy)pent-2-enamide (2x)

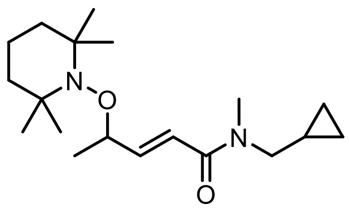

Following general procedure D using (*E*)-*N*-(cyclopropylmethyl)-*N*-methylpent-3-enamide **1x** (34 mg; 0.20 mmol; 1.0 equiv.), TEMPO (80 mg; 0.50 mmol; 2.5 equiv.), 4 Å molecular sieves (0.13 g; 0.94 mmol; 4.7 equiv.) and trifluoromethanesulfonic anhydride (37 µl; 0.22 mmol; 1.1 equiv.). Purification by flash column chromatography (heptane/ethyl acetate 0-50%) to give the title compound as an orange oil (42.2 mg; 65%).

2 rotamers are present in the NMR (1:1.25).

^{1}H NMR (400 MHz, CDCl$_3$)

Major: δ 6.85 (dd, *J* = 15.2, 6.6 Hz, 1H), 6.36 (d, *J* = 15.2 Hz, 1H), 4.47—4.41 (m, 1H), 3.37–3.23 (m, 2H), 3.04 (s, 3H), 1.59–1.49 (m, 1H), 1.38–1.47 (m, 4H), 1.28 (app d, *J* = 6.6 Hz, 4H), 1.16 (s, 3H), 1.09 (s, 9H), 1.01–0.93 (m, 1H), 0.58–0.45 (m, 2H), 0.28–0.17 (m, 2H) ppm.

Minor: δ 6.85 (dd, *J* = 15.2, 6.6 Hz, 1H), 6.36 (d, *J* = 15.2 Hz, 1H), 4.47—4.41 (m, 1H), 3.37–3.23 (m, 2H), 3.12 (s, 3H), 1.59–1.49 (m, 1H), 1.38–1.47 (m, 4H), 1.28 (app d, *J* = 6.6 Hz, 4H), 1.16 (s, 3H), 1.09 (s, 9H), 1.01–0.93 (m, 1H), 0.58–0.45 (m, 2H), 0.28–0.17 (m, 2H) ppm.

^{13}C NMR (101 MHz, CDCl$_3$)

Major: δ 166.6, 147.9, 119.0, 79.8, 59.9, 59.7, 54.3, 40.2 (2C), 34.6, 34.4, 34.2, 20.7, 20.4, 20.3, 17.3, 10.5, 3.54, 3.4 ppm.

Minor: δ 166.4, 148.0, 119.1, 79.8, 59.9, 59.7, 51.9, 40.2 (2C), 35.5, 34.6, 34.4, 20.7, 20.4, 20.3, 17.3, 9.4, 3.48, 3.4 ppm.

HRMS (ESI) (m/z): calculated for $[M+H]^+$ $C_{19}H_{34}O_2N_2H^+$ requires 323.2693, found 323.2682.

IR (cm^{-1}): 2929, 1660, 1619, 1453, 1399, 1256, 1131, 1043, 955

(*E*)-1-(2-Phenylpyrrolidin-1-yl)-4-((2,2,6,6-tetramethylpiperidin-1-yl)-oxy)-dodec-2-en-1-one (2y)

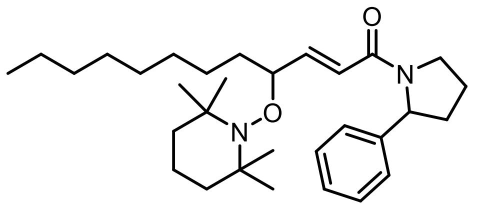

Following general procedure D using (*E*)-1-(2-phenylpyrrolidin-1-yl)-dodec-3-en-1-one **1y** (74.8 mg; 0.3 mmol; 1.0 equiv.), TEMPO (0.12 g; 0.75 mmol; 2.5 equiv.), 4 Å molecular sieves (0.20 g; 1.41 mmol; 4.7 equiv.) and trifluoromethanesulfonic anhydride (55.5 µl; 0.33 mmol; 1.1 equiv.). Purification by flash column chromatography (heptane/ethyl acetate 0-60%) gave the title compound as a slightly yellow oil (80.0 mg, 55%, d.r. 2.9/1).

Rotamers and diastereomers are present in NMR.

^{1}H NMR (400 MHz, $CDCl_3$):

Major: δ 7.41–7.24 (m, 2H), 7.24–7.12 (m, 3H), 6.79 (dd, *J* = 14.8 Hz, *J* = 7.4 Hz, 1H), 5.85 (d, *J* = 15.4 Hz, 1H), 5.14–5.01 (m, 1H), 4.09–3.98 (m, 1H), 3.91–3.65 (m, 2H), 2.48–1.32 (m, 1H), 2.04–1.81 (m, 3H), 1.70–0.77 (m, 35H) ppm.

Minor: δ 7.41–7.24 (m, 2H), 7.24–7.12 (m, 3H), 6.93–6.84 (m, 1H), 6.34–6.22 (m, 1H), 5.34–5.26 (m, 1H), 4.34–4.24 (m, 1H), 3.91–3.65 (m, 2H), 2.32–1.19 (m, 1H), 2.04–1.81 (m, 3H), 1.70–0.77 (m, 35H) ppm.

^{13}C NMR (150 MHz, $CDCl_3$):

Major: δ 165.5, 146.9, 143.6, 128.6, 128.4, 127.11, 125.49, 121.5, 121.3, 84.0, 61.4, 60.2 (2C), 47.0, 40.2 (2C), 36.3, 34.6 (2C), 33.8, 31.9, 29.5, 29.3, 24.9, 24.6, 22.6, 21.7, 20.3 (2C), 17.2, 14.1 ppm.

Minor: δ 165.5, 146.7, 143.8, 128.7, 128.4, 127.14, 125.51, 122.0, 121.3, 83.3, 61.5, 60.7 (2C), 47.1, 40.1 (2C), 36.4, 34.6 (2C), 34.0, 31.9, 29.6, 29.4, 25.0, 24.6, 22.6, 21.9, 20.3 (2C), 17.2, 14.1 ppm.

HRMS (ESI+): Calculated for $[M+Na]^+$ $C_{31}H_{50}O_2N_2Na^+$: 505.3764, found: 505.3755.

IR (cm^{-1}): 2923, 2855, 1741, 1663, 1622, 1408, 1208, 1133, 1029, 750, 699.

(5*R*,*E*)-*N*-Butyl-5,9-dimethyl-*N*-(3-methylbut-2-en-1-yl)-4-((2,2,6,6-tetramethylpiperidin-1-yl)-oxy)-deca-2,8-dienamide (2z)

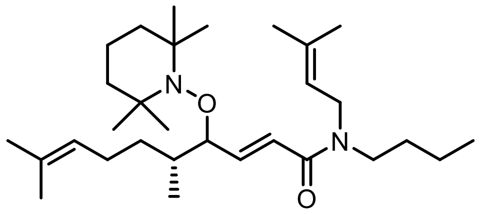

Following general procedure D using (*R*,*E*)-*N*-butyl-5,9-dimethyl-*N*-(3-methylbut-2-en-1-yl)-deca-3,8-dienamide **1z** (95.8 mg; 0.3 mmol; 1.0 equiv.), TEMPO (0.12 g; 0.75 mmol; 2.5 equiv.), 4 Å molecular sieves (0.20 g; 1.41 mmol; 4.7 equiv.) and trifluoromethanesulfonic anhydride (55.5 µl; 0.33 mmol; 1.1 equiv.). Purification by flash column chromatography (heptane/ethyl acetate 0-60%) gave the title compound as an orange oil (102 mg; 72%, d.r. 1.2/1).

Rotamers and diastereomers are present in NMR.

^{1}H NMR (400 MHz, $CDCl_3$):

Major: δ 6.96–6.81 (m, 1H), 6.34–6.13 (m, 1H), 5.23–5.05 (m, 2H), 4.14–3.86 (m, 3H), 3.34–3.21 (m, 2H), 2.13–1.99 (m, 3H), 1.74 (s, 3H), 1.73–1.67 (m, 6H), 1.64–1.50 (m, 6H), 1.49–1.38 (m, 4H), 1.33 (app q, *J* = 7.2 Hz, 4H), 1.23–1.03 (m, 13H), 0.93 (app q, *J* = 7.0 Hz, 6H) ppm.

Minor: δ 6.96–6.81 (m, 1H), 6.34–6.13 (m, 1H), 5.23–5.05 (m, 2H), 4.26–4.14 (m, 3H), 3.45–3.34 (m, 2H), 1.99–1.88 (m, 3H), 1.74 (s, 3H), 1.73–1.67 (m, 6H), 1.64–1.50 (m, 6H), 1.49–1.38 (m, 4H), 1.33 (app q, *J* = 7.2 Hz, 4H), 1.23–1.03 (m, 13H), 0.93 (app q, *J* = 7.0 Hz, 6H) ppm.

^{13}C NMR (150 MHz, $CDCl_3$):

Major: δ 165.7, 144.3, 135.2, 131.2, 124.4, 122.3, 120.4, 86.9, 59.9, 59.4, 45.9, 43.3, 40.1 (2C), 36.7, 36.2, 34.5, 34.2, 33.5, 31.5, 29.8, 25.6, 20.3, 20.0 (2C), 17.6, 17.1, 16.0, 14.3, 13.7 ppm.

Minor: δ 165.9, 144.1, 134.5, 131.0, 124.7, 122.4, 121.2, 87.6, 59.8, 59.6, 46.7, 46.0, 40.1 (2C), 36.7, 36.2, 34.5, 34.2, 33.5, 31.7, 29.8, 25.7, 20.2, 20.0 (2C), 17.6, 17.1, 16.0, 14.3, 13.8 ppm.

HRMS (ESI+): Calculated for $[M+H]^+$ $C_{30}H_{54}O_2N_2H^+$: 475.4258, found: 475.4268.

IR (cm^{-1}): 2962, 2927, 2873, 1659, 1622, 1440, 1422, 1375, 1360, 1258, 1132, 984, 749.

(*E*)-5-(benzo[d][1,3]dioxol-5-yl)-1-(piperidin-1-yl)-4-((2,2,6,6-tetramethylpiperidin-1-yl)oxy)pent-2-en-1-one (2aa)

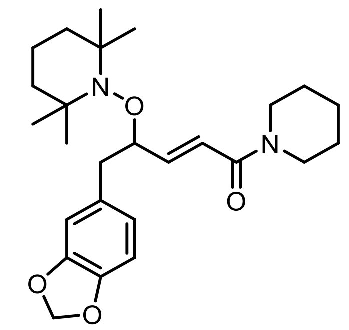

Following general procedure D using (*E*)-5-(benzo[d][1,3]dioxol-5-yl)-1-(piperidin-1-yl)pent-3-en-1-one[15] (45.6 mg; 0.16 mmol; 1.0 equiv.), TEMPO (63.3 mg; 0.4 mmol; 2.5 equiv.), 4 Å molecular sieves (0.1 g; 0.75 mmol; 4.7 equiv.) and trifluoromethanesulfonic anhydride (29.4 µl; 0.18 mmol; 1.1 equiv.). Purification by flash column chromatography (heptane/ethyl acetate 0-30%) to give the title compound as a yellow oil (56.2 mg; 80%).

^{1}H NMR (400 MHz, CDCl$_3$): δ 6.72–6.58 (m, 4H), 6.03 (d, *J* = 15.3 Hz, 1H), 5.90 (s, 2H), 4.45–4.37 (m, 1H), 3.67–3.43 (m, 2H), 3.29 (bs, 2H), 3.17 (dd, *J* = 13.4, 5.4 Hz, 1H), 2.65 (dd, *J* = 13.4, 8.3 Hz, 1H), 1.67–1.37 (m, 11H), 1.34–1.24 (m, 1H), 1.19–1.05 (m, 12H) ppm.

^{13}C NMR (100 MHz, CDCl$_3$): δ 165.3, 145.0, 131.9, 122.9, 121.7, 110.3, 108.0, 100.8, 85.4, 77.5, 77.2, 76.8, 60.4, 46.9, 43.0, 40.7, 40.3 (2C), 35.1, 34.2, 26.7, 25.6, 24.7, 20.6, 20.5, 17.3 ppm.

HRMS (ESI$^+$): Calculated for $[M+H]^+$ $C_{26}H_{38}N_2O_4H^+$: 443.2904, found: 443.2912.

IR (cm^{-1}): 2933, 1657, 1610, 1489, 1440, 1245, 1039, 908, 727.

(*E*)-*N*-(3-(10,11-dihydro-5*H*-dibenzo[b,f]azepin-5-yl)propyl)-*N*-methyl-4-((2,2,6,6-tetramethylpiperidin-1-yl)oxy)pent-2-enamide (2ab)

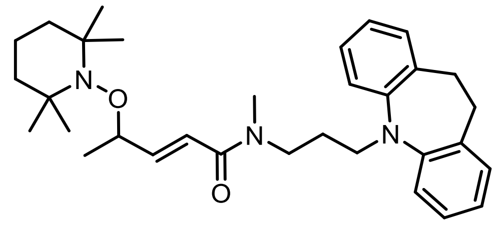

Following general procedure D using (*E*)-*N*-(3-(10,11-dihydro-5*H*-dibenzo[b,f]azepin-5-yl)propyl)-*N*-methylpent-3-enamide **1ab** (105 mg; 0.3 mmol; 1.0 equiv.), TEMPO (120 mg; 0.75 mmol; 2.5 equiv.), 4 Å molecular sieves (0.2 g; 1.41 mmol; 4.7 equiv.) and trifluoromethanesulfonic anhydride (55.5 µl; 0.33 mmol; 1.1 equiv.). Purification by flash column chromatography (heptane/ethyl acetate 0-60%) to give the title compound as a yellow oil (123 mg; 81%).

2 rotamers are present in the NMR (1:0.8).

^{1}H NMR (600 MHz, CDCl$_3$):

Major: δ 7.17–7.04 (m, 6H), 6.98–6.90 (m, 2H), 6.89–6.82 (m, 1H), 6.07 (d, *J* = 15.1 Hz, 1H), 4.31 (p, *J* = 6.5 Hz, 1H), 3.77 (bs, 2H), 3.51 – 3.42 (m, 2H), 3.19 (s, 4H), 2.87 (s, 3H), 1.91–1.80 (m, 2H), 1.60–1.38 (m, 4H), 1.34–1.24 (m, 3H), 1.23–0.96 (m, 14H) ppm.

Minor: δ 7.17–7.04 (m, 6H), 6.98–6.90 (m, 2H), 6.89–6.82 (m, 1H), 6.30 (d, *J* = 15.2 Hz, 1H), 4.48–4.40 (m, 1H), 3.77 (bs, 2H), 3.36–3.26 (m, 2H), 3.17 (s, 4H), 2.92 (s, 3H), 1.91–1.80 (m, 2H), 1.60–1.38 (m, 4H), 1.34–1.24 (m, 3H), 1.23–0.96 (m, 14H) ppm.

^{13}C NMR (100 MHz, CDCl$_3$):

Major: δ 166.4, 148.8, 147.8 (2C), 134.2 (2C), 130.1 (2C), 126.6 (2C), 123.00 (2C), 119.7 (2C), 118.2, 80.0, 59.8 (2C), 48.0, 47.5, 40.3 (2C), 34.8, 34.4, 34.1, 32.2 (2C), 27.1, 20.8, 20.5, 20.3, 17.30 ppm.

Minor: δ 166.4, 148.2, 148.2 (2C), 134.3 (2C), 129.9 (2C), 126.5 (2C), 122.6 (2C), 120.1 (2C), 118.9, 79.8, 59.8 (2C), 48.2, 46.0, 40.3 (2C), 35.6, 34.6, 34.4, 32.3 (2C), 26.0, 20.6, 20.5, 20.3, 17.29 ppm.

HRMS (ESI$^+$): Calculated for $[M+Na]^+$ $C_{32}H_{45}N_3O_2Na^+$: 526.3404, found: 526.3400.

IR (cm^{-1}): 2927, 1660, 1616, 1486, 1230, 1042, 955, 748.

2.4 Mechanistic investigations

(*E*)-1-(Pyrrolidin-1-yl)-4-((2,2,6,6-tetramethylpiperidin-1-yl)-oxy)-pent-2-en-1-one (2a)

Evaporation of the reaction mixture

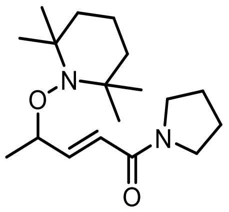

Following general procedure D using (*E*)-1-(pyrrolidin-1-yl)-pent-3-en-1-one **1a** (46.0 mg; 0.3 mmol; 1.0 equiv.), TEMPO (0.12 g; 0.75 mmol; 2.5 equiv.), 4 Å molecular sieves (0.20 g; 1.41 mmol; 4.7 equiv.) and trifluoromethanesulfonic anhydride (55.5 µl; 0.33 mmol; 1.1 equiv.). After 30 min, the solvent was evaporated under reduced pressure. No defined species could be elucidated, but the product has not yet formed prior to quenching.

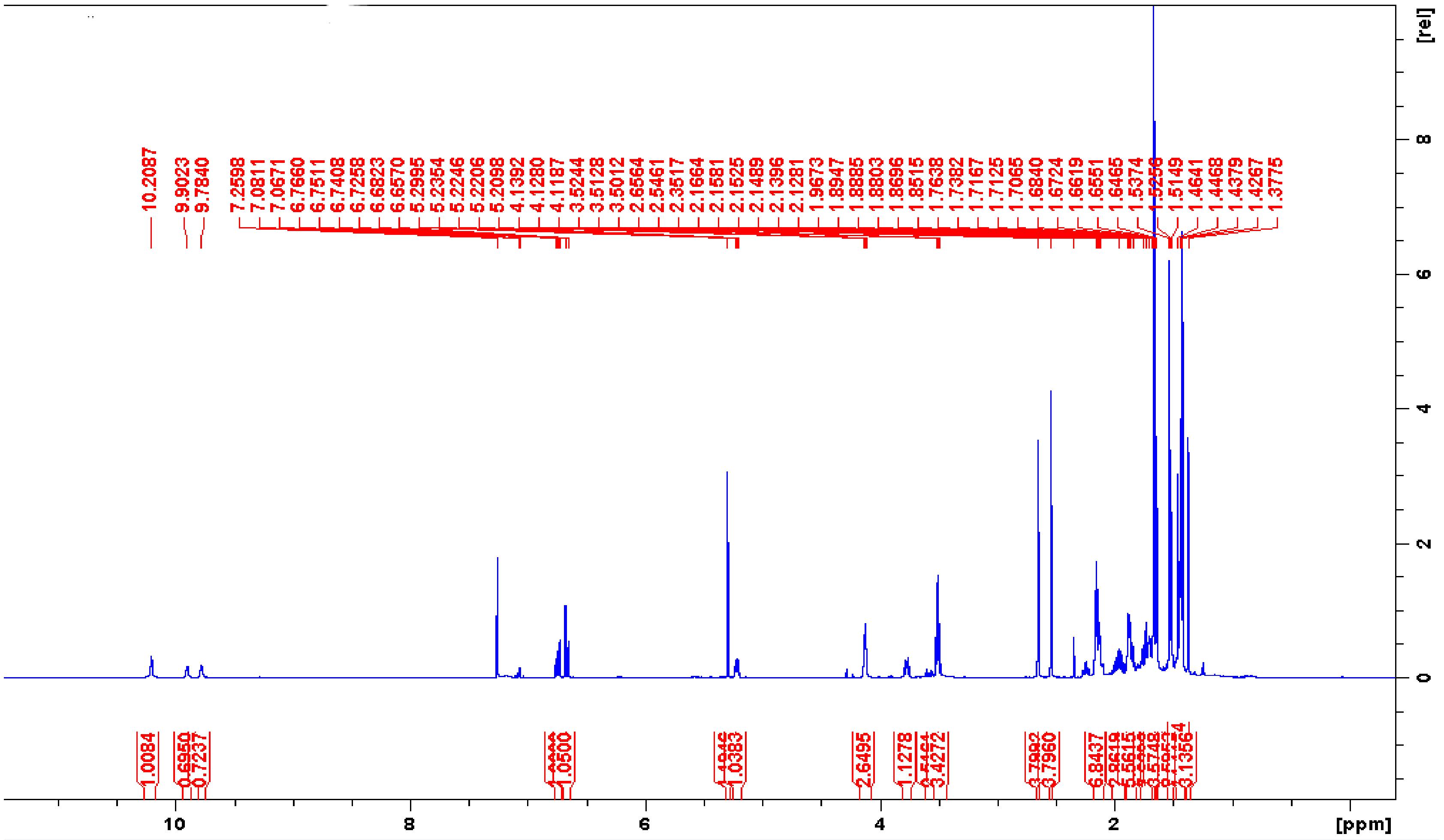

Red: amide **2a**, Blue: obtained mixture

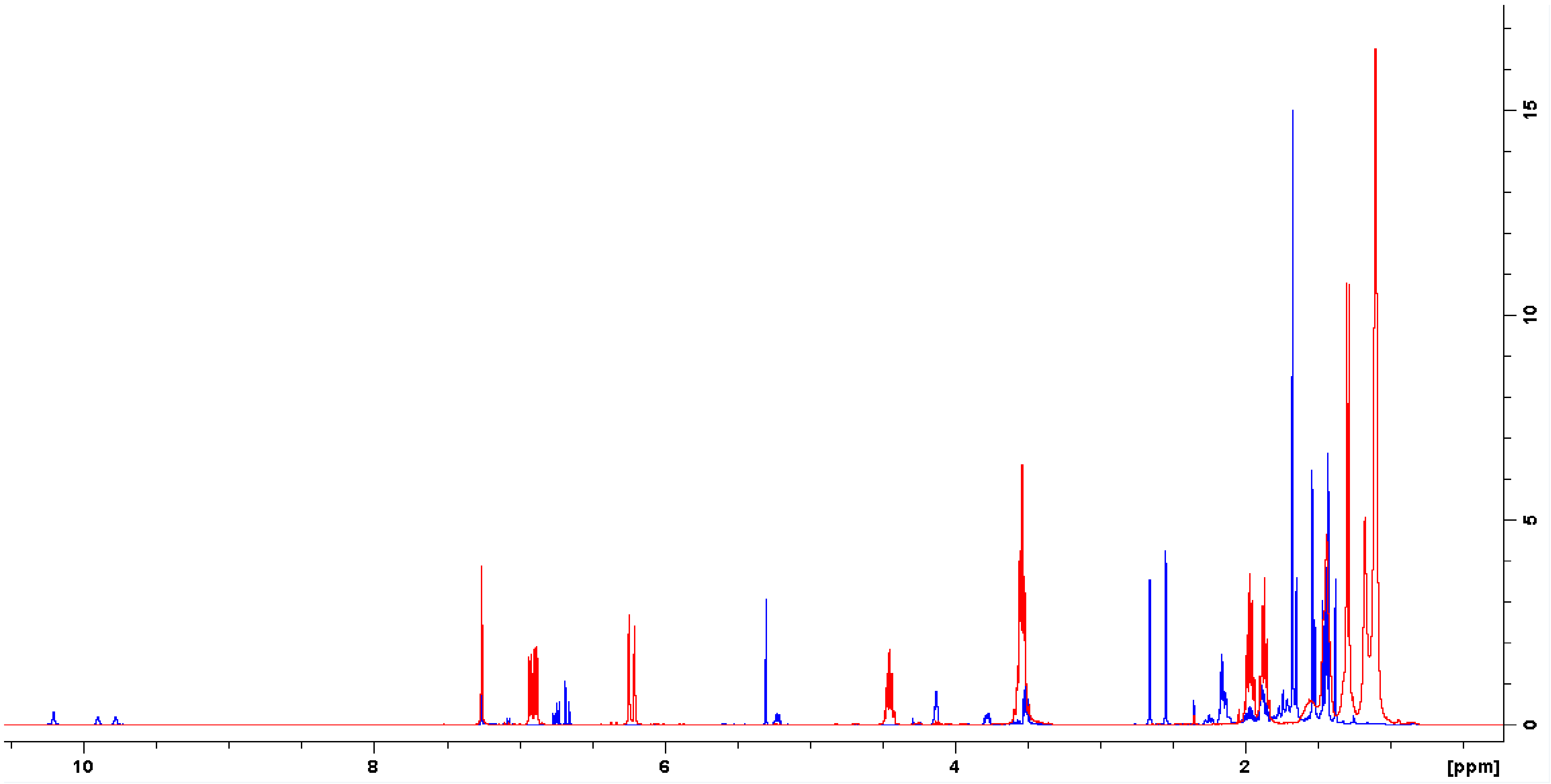

1-

Isopropyl-2,2-dimethylpyrrolidine (VI)

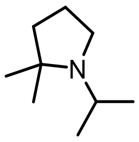

Following general procedure D using (*E*)-1-(pyrrolidin-1-yl)-pent-3-en-1-one **1a** (30.6 mg; 0.2 mmol; 1.0 equiv.), TEMPO (79.7 mg; 0.50 mmol; 2.5 equiv.), and trifluoromethanesulfonic anhydride (37 µl; 0.22 mmol; 1.1 equiv.). After 30 min, the mixture was cooled to –78 °C and sodium borohydride (30.3 mg, 0.80 mmol, 4.0 equiv.) was added. The mixture was allowed to warm to room temperature and was further stirred for 16 h. A saturated aqueous solution of sodium bicarbonate (2 ml) was then added and the biphasic mixture was diluted with CH_2Cl_2 (4 ml). The organic layer was separated, washed with further aqueous sodium bicarbonate solution (3 ml), and dried over anhydrous magnesium sulfate. The dried solution was filtered, and the filtrate was concentrated under reduced pressure. The crude residue was purified by flash column chromatography (SiO_2, heptane/ethyl acetate 0-80%) to afford **VI** as a colourless oil (9.1 mg, 32% yield).

^{1}H NMR (700 MHz, $CDCl_3$) δ 3.50 (td, *J* = 10.7, 2.5 Hz, 1H), 3.09 (sept, *J* = 6.5 Hz, 1H), 2.88 (app q, *J* = 10.0 Hz, 1H), 2.49 (td, *J* = 12.4, 7.1 Hz, 1H), 2.07 (ttd, *J* = 12.6, 9.5, 3.3 Hz, 1H), 1.78–1.71 (m, 1H), 1.69–1.63 (m, 1H), 1.56 (s, 3H), 1.37 (d, *J* = 6.4 Hz, 3H), 1.34 (d, *J* = 6.6 Hz, 3H), 1.26 (s, 3H) ppm.

^{13}C NMR (176 MHz, $CDCl_3$) δ 71.2, 63.0, 59.2, 40.4, 26.6, 23.6, 21.3, 19.6, 19.1 ppm.

HRMS (ESI+): Calculated for $[M+H]^+$ $C_9H_{19}NH^+$: 142.1590, found: 142.1592.

IR (cm^{-1}): 2959, 2869, 2405, 2357, 1458, 1381, 1359, 1187, 1144, 860.

2.5 Functionalization reactions

2.5.1 Oxidation/Reduction

(*E*)-1-(Pyrrolidin-1-yl)-pent-2-ene-1,4-dione (3a)

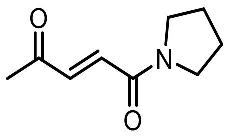

(*E*)-1-(Pyrrolidin-1-yl)-4-((2,2,6,6-tetramethylpiperidin-1-yl)-oxy)-pent-2-en-1-one **2a** (92.5 mg; 0.3 mmol; 1.0 equiv.) was dissolved in CH_2Cl_2 (0.2 M). At 0 °C, *m*-CPBA (80.7 mg; 0.36 mmol; 1.2 equiv.) was added over a period of 10 minutes and the reaction was stirred for an additional 30 min, before the addition of a saturated aqueous solution of sodium thiosulfate (7.5 ml). The biphasic mixture was separated, and the aqueous phase was extracted sequentially with CH_2Cl_2 (2 × 10 ml) and ethyl acetate (2 × 10 ml). The combined organic phases were washed sequentially with a saturated aqueous solution of sodium bicarbonate (1 × 10 ml) and brine (1 × 10 ml) and dried over anhydrous magnesium sulfate. The dried solution was filtered, and the filtrate was concentrated under reduced pressure. The crude residue was purified by flash column chromatography (SiO_2, heptane/ethyl acetate 0-80%) to afford the title compound as white crystals (39.0 mg, 78%).

^{1}H NMR (400 MHz, $CDCl_3$): δ 7.11 (d, *J* = 15.3 Hz, 1H), 7.04 (d, *J* = 15.3 Hz, 1H), 3.58 (dt, *J* = 11.8, 6.6 Hz, 4H), 2.35 (s, 3H), 1.96 (ddt, *J* = 37.7, 13.4, 6.6 Hz, 4H) ppm.

^{13}C NMR (150 MHz, $CDCl_3$): δ 197.9, 163.1, 137.0, 132.0, 47.0, 46.4, 29.6, 26.2, 24.4 ppm.

HRMS (ESI+): Calculated for $[M+Na]^+$ $C_9H_{13}O_2NNa^+$: 190.0838, found: 190.0843.

IR (cm^{-1}): 2977, 2878, 1662, 1646, 1604, 1424, 1369, 1252, 976866, 750, 703, 590.

All analytical data were in good accordance with data reported in the literature.[12]

(*E*)-1-(Pyrrolidin-1-yl)-pent-3-en-1-one (1a) through reduction of 2a

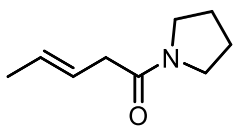

(*E*)-1-(Pyrrolidin-1-yl)-4-((2,2,6,6-tetramethylpiperidin-1-yl)-oxy)-pent-2-en-1-one **2a** (61.7 mg; 0.2 mmol; 1.0 equiv.) was dissolved in CH_2Cl_2 (0.1 M) and treated with activated zinc powder (0.13 g; 2.0 mmol; 10 equiv.), water (2.0 ml) and acetic acid (2.0 ml). The resulting mixture was stirred vigorously at 50 °C for 2 h. After that time, the reaction was allowed to cool to room temperature and subsequently excess acid was quenched by the careful addition of a saturated aqueous solution of sodium bicarbonate (5 ml). After gas evolution had ceased, a saturated aqueous solution of Rochelle's salt (5 ml) was added and the resulting solution was stirred for 30 min. The biphasic mixture was then separated, and the aqueous phase was extracted with CH_2Cl_2 (3 × 10 ml). The combined organic phases were dried over anhydrous magnesium sulfate, the dried solution was filtered, and the filtrate was concentrated under reduced pressure. The crude residue was purified by flash column chromatography (SiO_2, heptane/ethyl acetate 20-100%) to afford the title compound **1a** as a yellowish oil (29.1 mg, 95%).

Preparation of activated zinc powder:

Zinc powder was stirred in aqueous HCl (1 M) for 1 minute, then the acid was removed by filtration and the remaining zinc was washed 3 times with water, twice with ethanol and twice with diethyl ether. Then the powder was dried under reduced pressure.

Analytical data is provided in chapter 2.2.3.

(*Z*)-1-(pyrrolidin-1-yl)pent-2-ene-1,4-dione (3b)

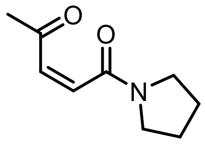

(*E*)-1-(Pyrrolidin-1-yl)-4-((2,2,6,6-tetramethylpiperidin-1-yl)-oxy)-pent-2-en-1-one **2a** (92.5 mg; 0.3 mmol; 1.0 equiv.) was dissolved in acetonitrile (3 ml). Oxygen was bubbled through the solution for 5 min and the reaction was subsequently irradiated in a Rayonet apparatus for 4 days under an oxygen atmosphere. After this time, the reaction mixture was concentrated under reduced pressure and the crude residue was purified by flash column chromatography (SiO_2, heptane/ethyl acetate 20-100%) to afford the title compound as a yellowish oil (11.6 mg, 23%).

^{1}H NMR (700 MHz, $CDCl_3$) δ 6.33 (d, *J* = 12.0 Hz, 1H), 6.27 (d, *J* = 12.0 Hz, 1H), 3.53 (t, *J* = 6.8 Hz, 2H), 3.40 (t, *J* = 6.7 Hz, 2H), 2.33 (s, 3H), 1.97–1.94 (m, 2H), 1.93–1.89 (m, 2H) ppm.

^{13}C NMR (176 MHz, $CDCl_3$) δ 200.4, 164.8, 134.6, 131.2, 47.0, 45.6, 30.2, 26.1, 24.4 ppm.

HRMS (ESI) (m/z): calculated for $[M+Na]^+$ $C_9H_{13}O_2NNa^+$ requires 190.0838, found 190.0835.

IR (cm^{-1}): 2971, 2876, 1710, 1623, 1437, 1340, 1164, 730.

2.5.2 Cyclizations

(*E*)-3-((2R)-2-Methyl-5-(prop-1-en-2-yl)-cyclopentyl)-1-(pyrrolidin-1-yl)-prop-2-en-1-one (4a)

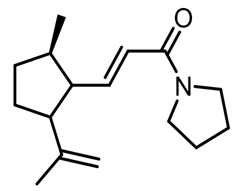

(*R*,*E*)-5,9-Dimethyl-1-(pyrrolidin-1-yl)-4-((2,2,6,6-tetramethylpiperidin-1-yl)-oxy)-deca-2,8-dien-1-one **2n** (60.7 mg; 0.15 mmol; 1.0 equiv.) was dissolved in *tert*-butanol (1.41 ml). The mixture was heated to 150 °C in a microwave reactor (ramping time 20 min.). After cooling, the solution was diluted with diethyl ether (5 ml) and washed with water (3 × 5 ml). The organic phase was dried over anhydrous magnesium sulfate. The dried solution was filtered, and the filtrate was concentrated under reduced pressure. The crude residue was purified by flash column chromatography (SiO_2, heptane/ethyl acetate) to afford the title compound as an orange solid (30.7 mg, 83%, d.r. 3.9/1.7/1).

2 diastereomers are present in NMR.

^{1}H NMR (400 MHz, $CDCl_3$):

Major: δ 6.73 (dd, *J* = 14.8, 9.0 Hz, 1H), 6.03 (d, *J* = 15.0 Hz, 1H), 4.67 (s, 2H), 3.50 (app q, *J* = 4.9 Hz, 4H), 2.45 (q, *J* = 9.6 Hz, 1H) 2.06–1.75 (m, 7H), 1.66 (s, 3H), 1.63–1.51 (m, 1H), 1.40–1.16 (m, 2H), 1.02–0.94 (m, 3H) ppm.

Minor 1: δ 6.79 (dd, *J* = 14.9, 8.1 Hz, 1H), 5.99 (d, *J* = 14.9 Hz, 1H), 4.72–4.68 (m, 2H), 3.50 (app q, *J* = 4.9 Hz, 4H), 2.59–2.53 (m, 1H), 2.36–2.28 (m, 3H), 2.06–1.75 (m, 9H), 1.63–1.51 (m, 1H), 0.92–0.85 (m, 3H) ppm.

Minor 2: δ 6.89–6.81 (m, 1H), 6.03 (d, *J* = 15.0 Hz, 1H), 4.78–4.72 (m, 2H), 3.50 (app q, *J* = 4.9 Hz, 4H), 2.73–2.65 (m, 1H), 2.28–2.20 (m, 3H), 2.06–1.75 (m, 9H), 1.63–1.51 (m, 1H), 0.92–0.85 (m, 3H) ppm.

^{13}C NMR (150 MHz, $CDCl_3$):

Major: δ 164.9, 148.0, 146.80, 122.0, 110.31, 55.5, 53.7, 46.6, 45.9, 40.8, 33.2, 30.1, 26.2, 24.4, 20.1, 18.9 ppm.

Minor 1: δ 164.9, 146.83, 146.80, 121.0, 111.0, 53.3, 50.2, 46.6, 45.9, 38.4, 33.9, 30.4, 26.2, 24.4, 20.5, 17.0 ppm.

Minor 2: δ 164.9, 146.89, 146.80, 121.0, 110.27, 55.5, 50.4, 46.6, 45.9, 39.9, 33.6, 29.5, 26.2, 24.4, 20.5, 17.0 ppm.

HRMS (ESI+): Calculated for $[M+Na]^+$ $C_{16}H_{25}ONNa^+$: 270.1828, found: 270.1822.

IR (cm^{-1}): 2948, 2867, 1659, 1613, 1419980, 884, 750.

(*E*)-1-Butyl-3-(prop-1-en-1-yl)-4-(prop-1-en-2-yl)-pyrrolidin-2-one (4b)

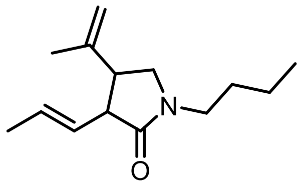

(*E*)-*N*-Butyl-*N*-(3-methylbut-2-en-1-yl)-4-((2,2,6,6-tetramethylpiperidin-1-yl)-oxy)-pent-2-enamide **2u** (34.8 mg; 0.09 mmol; 1.0 equiv.) was dissolved in *tert*-butanol (0.86 ml). The mixture was heated to 150 °C in a microwave reactor (ramping time 20 min.). After cooling, the solution was diluted with diethyl ether (5 ml) and washed with water (3 × 5 ml). The organic phase was dried over anhydrous magnesium sulfate. The dried solution was filtered, and the filtrate was concentrated under reduced pressure. The crude residue was purified by flash column chromatography (SiO_2, heptane/ethyl acetate) to afford the title compound as a yellow oil (9.6 mg, 48%, d.r. 2.1/1).

One-pot approach: (*E*)-*N*-butyl-*N*-(3-methylbut-2-en-1-yl)-pent-3-enamide **1u** (67.0 mg; 0.3 mmol; 1.0 equiv.), TEMPO (0.12 g; 0.75 mmol; 2.5 equiv.) and trifluoromethanesulfonic anhydride (55.5 µl; 0.33 mmol; 1.1 equiv.) were mixed in CH_2Cl_2 (0.1 м) in a flame-dried flask suited for the microwave. The resulting mixture was stirred 30 min at room temperature, before being heated in the microwave for 1 h at 150 °C with 20 minutes ramping. After cooling to room temperature, the solution was diluted with diethyl ether (15 ml) and washed with water (3 × 15 ml). The organic phase was dried over anhydrous magnesium sulfate. The dried solution was filtered, and the filtrate was concentrated under reduced pressure. The crude residue was purified by flash column chromatography (SiO_2, heptane/ethyl acetate) to afford the title compound as a yellow oil (26.5 mg, 40%, d.r. 1.8/1).

2 diastereomers are present in NMR.

^{1}H NMR (400 MHz, $CDCl_3$):

Major: δ 5.67–5.57 (m, 1H), 5.46–4.40 (m, 1H), 4.87–4.81 (m, 2H), 3.43–3.35 (m, 1H), 3.32–3.24 (m, 2H), 3.11 (t, *J* = 8.9 Hz, 1H), 2.80–2.71 (m, 1H), 1.77–1.67 (m, 6H), 1.55–1.44 (m, 2H), 1.36–1.25 (m, 3H), 0.92 (t, *J* = 7.4 Hz, 3H) ppm.

Minor: 5.82–5.71 (m, 1H), 5.32–5.21 (m, 1H), 4.87–4.81 (m, 2H), 3.43–3.35 (m, 1H), 3.32–3.24 (m, 2H), 2.99 (t, *J* = 8.9 Hz, 1H), 2.80–2.71 (m, 1H), 1.77–1.67 (m, 6H), 1.55–1.44 (m, 2H), 1.36–1.25 (m, 3H), 0.92 (t, *J* = 7.4 Hz, 3H) ppm.

^{13}C NMR (150 MHz, $CDCl_3$):

Major: δ 174.4, 143.4, 129.4, 127.7, 112.5, 49.8, 49.7, 46.6, 42.5, 29.5, 20.29, 20.2, 18.3, 13.9 ppm.

Minor: δ 174.4, 143.4, 129.1, 127.4, 112.0, 49.7, 49.6, 45.6, 42.6, 29.5, 20.26, 20.2, 18.3, 13.6 ppm.

HRMS (ESI+): Calculated for $[M+Na]^+$ $C_{14}H_{23}ONNa^+$: 244.1672, found: 244.1671.

IR (cm^{-1}): 2959, 2929, 2864, 1687, 1648, 1489, 1427, 1377, 1317, 1258, 959, 890, 750.

1-Butyl-3-((*R*,*E*)-3,7-dimethylocta-1,6-dien-1-yl)-4-(prop-1-en-2-yl)-pyrrolidin-2-one (4c)

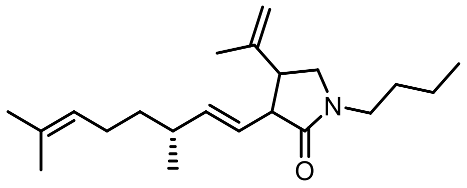

(5*R*,*E*)-*N*-Butyl-5,9-dimethyl-*N*-(3-methylbut-2-en-1-yl)-4-((2,2,6,6-tetramethylpiperidin-1-yl)-oxy)-deca-2,8-dienamide **2z** (71.2 mg; 0.15 mmol; 1.0 equiv.) was dissolved in *tert*-butanol (1.41 ml). The mixture was heated to 150 °C in a microwave reactor (ramping time 20 min.). After cooling, the solution was diluted with diethyl ether (5 ml) and washed with water (3 × 5 ml). The organic phase was dried over anhydrous magnesium sulfate. The dried solution was filtered, and the filtrate was concentrated under reduced pressure. The crude residue was purified by flash column chromatography (SiO_2, heptane/ethyl acetate) to afford the title compound as a yellow oil (43.4 mg, 91%, d.r. 1.2/1).

2 diastereomers are present in NMR.

^{1}H NMR (600 MHz, $CDCl_3$:

Major: δ 5.50–5.41 (m, 2H), 5.11–5.05 (m, 1H), 4.85 (br s, 2H), 3.42–3.36 (m, 1H), 3.29 (t, *J* = 7.4 Hz, 2H), 3.13 (t, *J* = 8.9 Hz, 1H), 2.99 (t, *J* = 7.9 Hz, 1H), 2.80–2.72 (m, 1H), 2.19–2.10 (m, 1H), 2.01–1.86 (m, 2H), 1.74 (s, 3H), 1.67 (s, 3H), 1.58 (d, *J* = 5.9 Hz, 3H), 1.54–1.46 (m, 2H), 1.35–1.27 (m, 4H), 0.98 (app dd, *J* = 12.2, 6.7 Hz, 3H), 0.93 (t, *J* = 7.4 Hz, 3H) ppm.

Minor: δ 5.41–5.34 (m, 2H), 5.11–5.05 (m, 1H), 4.84 – 4.81 (m, 2H), 3.42–3.36 (m, 1H), 3.29 (t, *J* = 7.4 Hz, 2H), 3.13 (t, *J* = 8.9 Hz, 1H), 2.99 (t, *J* = 7.9 Hz, 1H), 2.80–2.72 (m, 1H), 2.19–2.10 (m, 1H), 2.01–1.86 (m, 2H), 1.74 (s, 3H), 1.67 (s, 3H), 1.58 (d, *J* = 5.9 Hz, 3H), 1.54–1.46 (m, 2H), 1.35–1.27 (m, 4H), 0.98 (app dd, *J* = 12.2, 6.7 Hz, 3H), 0.93 (t, *J* = 7.4 Hz, 3H) ppm.

^{13}C NMR (150 MHz, $CDCl_3$):

Major: δ 174.3, 143.3, 140.4, 131.3, 125.0 (2C), 112.6, 49.8, 49.7, 46.9, 42.5, 37.2, 36.6, 29.5, 25.9, 25.8, 20.7, 20.4, 20.2, 17.8, 13.9 ppm.

Minor: δ 174.4, 143.5, 139.9, 131.3, 124.9 (2C), 112.4, 50.0, 49.8, 46.5, 42.5, 37.2, 36.4, 29.5, 25.84, 25.82, 20.7, 20.4, 20.2, 17.8, 13.9 ppm.

HRMS (ESI+): Calculated for $[M+Na]^+$ $C_{21}H_{35}ONNa^+$: 340.2611, found: 340.2624.

IR (cm^{-1}): 2959, 2927, 2867, 1692, 1426, 1376, 1275, 1259, 965, 890, 750.

(*E*)-1-Methyl-3-(prop-1-en-1-yl)-4-(((2,2,6,6-tetramethylpiperidin-1-yl)-oxy)-methyl)-pyrrolidin-2-one (4d)

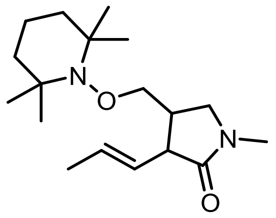

(*E*)-*N*-Allyl-*N*-methyl-4-((2,2,6,6-tetramethylpiperidin-1-yl)-oxy)-pent-2-enamide **2v** (36.2 mg; 0.12 mmol; 1.0 equiv.) was dissolved in *tert*-butanol (1.1 ml). The mixture was heated to 150 °C in a microwave reactor (ramping time 20 min.). After cooling, the solution was diluted with diethyl ether (5 ml) and washed with water (3 × 5 ml). The organic phase was dried over anhydrous magnesium sulfate. The dried solution was filtered, and the filtrate was concentrated under reduced pressure. The crude residue was purified by flash column chromatography (SiO_2, heptane/ethyl acetate) to afford the title compound as a yellow oil (22.9 mg, 62%, d.r. 1.6/1).

2 diastereomers are present in NMR.

^{1}H NMR (400 MHz, $CDCl_3$):

Major: δ 5.70–5.60 (m, 1H), 5.45–5.35 (m, 1H), 3.86–3.75 (m, 2H), 3.48–3.37 (m, 1H), 3.30–3.21 (m, 1H), 2.88–2.84 (m, 4H), 2.41–2.28 (m, 1H), 1.71 (d, *J* = 6.7 Hz, 3H), 1.47–1.40 (m, 4H), 1.36–1.22 (m, 2H), 1.17–1.05 (m, 12H) ppm.

Minor: δ 5.82–5.72 (m, 1H), 5.35–5.24 (m, 1H), 3.77–3.61 (m, 2H), 3.48–3.37 (m, 1H), 3.30–3.21 (m, 1H), 2.88–2.84 (m, 4H), 2.78–2.61 (m, 1H), 1.71 (d, *J* = 6.7 Hz, 3H), 1.47–1.40 (m, 4H), 1.36–1.22 (m, 2H), 1.17–1.05 (m, 12H) ppm.

^{13}C NMR (150 MHz, $CDCl_3$):

Major: δ 174.6, 129.5, 127.7, 76.7, 60.0 (2C), 51.0, 48.3, 39.7 (2C), 38.9, 33.3, 33.2, 30.0, 20.2 (2C), 18.2, 17.1 ppm.

Minor: δ 174.8, 129.2, 127.2, 76.5, 60.0 (2C), 51.3, 48.5, 40.0, 39.7 (2C), 33.3, 33.2, 30.1, 20.2 (2C), 18.3, 17.1 ppm.

HRMS (ESI+): Calculated for $[M+H]^+$ $C_{18}H_{32}O_2N_2H^+$: 309.2537, found: 309.2537.

IR (cm^{-1}): 2974, 2928, 2875, 1688, 1402, 1359, 1263, 1244, 1132, 1097, 1068, 1047, 958, 923, 752, 729.

2.5.3 Synthesis of a bioactive compound according to Omura *et al.*[13]

(*E*)-*N*,*N*-Dimethyl-4-oxododec-2-enamide (3c)

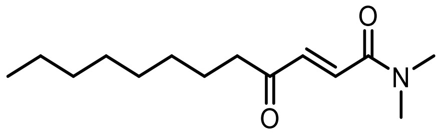

Following the general procedure D using (*E*)-*N*,*N*-dimethyldodec-3-enamide **1ad** (114.0 mg; 0.3 mmol; 1.0 equiv.), TEMPO (0.12 g; 0.75 mmol; 2.5 equiv.), 4 Å molecular sieves (0.20 g; 1.41 mmol; 4.7 equiv.) and trifluoromethanesulfonic anhydride (55.5 µl; 0.33 mmol; 1.1 equiv.). The crude material was dissolved in CH_2Cl_2 (0.2 M) and, at 0 °C, *m*-CPBA (80.7 mg; 0.36 mmol; 1.2 equiv.) was added over a period of 10 minutes. The reaction was stirred for an additional 30 min, before it was stopped with the addition of a saturated aqueous solution of $Na_2S_2O_3$ (7.5 ml). The biphasic mixture was separated, and the aqueous phase was extracted sequentially with CH_2Cl_2 (2 × 10 ml) and ethyl acetate (2 × 10 ml). The combined organic phases were washed sequentially with a saturated aqueous solution of sodium bicarbonate (10 ml) and brine (10 ml) and dried over anhydrous magnesium sulfate. The dried solution was filtered, and the filtrate was concentrated under reduced pressure. The crude residue was purified by flash column chromatography (SiO_2, heptane/ethyl acetate 0-80%) to afford the title compound as light yellow crystals (58.0 mg, 81%).

^{1}H NMR (400 MHz, CDCl$_3$: δ 7.25 (d, *J* = 15.1 Hz, 1H), 7.08 (d, *J* = 15.2 Hz, 1H), 3.04 (s, 3H), 2.93 (s, 3H), 2.60 (t, *J* = 7.33 Hz, 2H), 1.35–1.20 (m 12H), 0.87 (t, *J* = 6.5 Hz, 3H) ppm.

^{13}C NMR (150 MHz, CDCl$_3$): δ 200.3, 165.4, 136.8, 130.5, 43.0, 37.7, 36.0, 31.9, 29.5, 29.3, 29.2, 23.9, 22.8, 14.2 ppm.

HRMS (ESI+): Calculated for $[M+Na]^+$ $C_{14}H_{25}O_2NNa^+$: 262.1778, found: 262.1770.

IR (cm^{-1}): 2921, 2852, 1696, 1647, 1611, 1392, 1268, 1139, 983, 751, 721, 701, 606.

(*E*)-1-(Pyrrolidin-1-yl)dodec-2-ene-1,4-dione (3d)

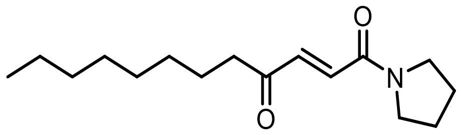

Following the general procedure D using (*E*)-1-(pyrrolidin-1-yl)-tridec-3-en-1-one **1b** (75.0 mg; 0.3 mmol; 1.0 equiv.), TEMPO (0.12 g; 0.75 mmol; 2.5 equiv.), 4 Å molecular sieves (0.20 g; 1.41 mmol; 4.7 equiv.) and trifluoromethanesulfonic anhydride (55.5 µl; 0.33 mmol; 1.1 equiv.). The crude material was dissolved in CH_2Cl_2 (0.2 M), at 0 °C, *m*CPBA (80.7 mg; 0.36 mmol; 1.2 equiv.) was added over a period of 10 minutes. The reaction was stirred for an additional 30 minutes, before it was stopped with the addition of a saturated aqueous solution $Na_2S_2O_3$ (7.5 ml). The biphasic mixture was separated, and the aqueous phase was extracted sequentially with CH_2Cl_2 (2 × 10 ml) and ethyl acetate (2 × 10 ml). The combined organic phases were washed sequentially with a saturated aqueous solution of sodium bicarbonate (10 ml) and brine (10 ml) and dried over anhydrous magnesium sulfate. The dried solution was filtered, and the filtrate was concentrated under reduced pressure. The crude residue was purified by flash column chromatography (SiO_2, heptane/ethyl acetate 0-80%) to afford the title compound as light yellow crystals (47.1 mg, 59%).

^{1}H NMR (400 MHz, CDCl$_3$): δ 7.16 (d, *J* = 15.4 Hz, 1H), 7.08 (d, *J* = 15.4 Hz, 1H), 3.63–3.53 (m, 4H), 2.60 (t, *J* = 7.1 Hz, 2H), 2.03–1.83 (m, 4H), 1.70–1.75 (m, 2H), 1.36–1.19 (m, 10H), 0.91–0.83 (m, 3H) ppm.

^{13}C NMR (150 MHz, CDCl$_3$): δ 200.5, 163.2, 136.5, 131.3, 47.0, 46.4, 43.1, 31.9, 29.5, 29.31, 29.25, 26.2, 24.4, 23.9, 22.8, 14.2 ppm.

HRMS (ESI+): Calculated for $[M+Na]^+$ $C_{16}H_{27}ON_2Na^+$: 288.1934 found: 288.1939.

IR (cm^{-1}): 2922, 2854, 1692, 1636, 1608, 1425, 1400, 1253, 1194, 1088, 982, 723, 648.

2.5.4 Desaturation to the α,β-γ,δ-unsaturated amide

(*E*)-1-(Pyrrolidin-1-yl)penta-2,4-dien-1-one (12)

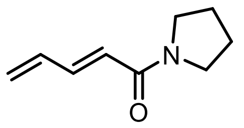

(*E*)-1-(Pyrrolidin-1-yl)-4-((2,2,6,6-tetramethylpiperidin-1-yl)-oxy)-pent-2-en-1-one **2a** (46 mg, 0.15 mmol, 1 equiv.) was placed in a microwave tube, which was subsequently sealed with a rubber septum. The tube was evacuated and back-filled with argon five times, before 1,2-dichloroethane (1.5 ml) was added and the resulting solution was deoxygenated by sparging with argon for 5 min. The sealed tube was then heated at 120 °C for 1 h under microwave irradiation. After cooling, volatiles were removed under reduced pressure and the residue was purified by flash column chromatography (SiO_2, heptane/ethyl acetate 0-50%), yielding the title compound as a colourless oil (16.5 mg, 73%).

^{1}H NMR (600 MHz, $CDCl_3$): δ 7.27 (dd, *J* = 14.9, 11.0 Hz, 1H), 6.51–6.36 (m, 1H), 6.21 (d, *J* = 14.9 Hz, 1H), 5.56 (d, *J* = 16.9 Hz, 1H), 5.41 (d, *J* = 10.0 Hz, 1H), 3.61–3.45 (m, 4H), 1.96 (p, *J* = 6.8 Hz, 2H), 1.87 (p, *J* = 6.8 Hz, 2H) ppm.

^{13}C NMR (150 MHz, $CDCl_3$): δ 164.8, 142.0, 135.3, 124.4, 123.0, 46.6, 46.1, 26.2, 24.5 ppm.

HRMS (ESI$^+$): Calculated for $[M+Na]^+$ $C_9H_{13}ONNa^+$: 174.0889, found: 174.0889.

IR (cm^{-1}): 2969, 2871, 1650, 1607, 1593, 1430, 1402, 1043, 914, 858, 727.

3. References

[1] P. J. Stang, T. E. Dueber, *Org. Synth.* **1988**, VI, 757.

[2] K. Lagerblom, J. Keskiväli, A. Parviainen, J. Mannisto, T. Repo, *ChemCatChem* **2018**, *10*, 2908–2914.

[3] S. Raghavan, S. G. Subramanian, *Tetrahedron* **2011**, *67*, 7529–7539.

[4] M. Fujii, K. Nakamura, S. Yasui, S. Oka, A. Ohno, *BCSJ* **1987**, *60*, 2423–2427.

[5] G. Wang, X. Liang, L. Chen, Q. Gao, J. Wang, P. Zhang, Q. Peng, S. Xu, *Angew. Chem. Int. Ed.* **2019**, *24*, 8187–8191.

[6] S. Desrat, P. J. Gray, M. R. Penny, W. B. Motherwell, *Chem. Eur. J.* **2014**, *20*, 8918–8922.

[7] R. H. Van der Veen, H. Cerfontain, *J. Org. Chem.* **1985**, *50*, 342–346.

[8] S. Tsuboi, Y. Nooda, A. Takeda, *J. Org. Chem.* **1984**, *49*, 1204–1208.

[9] G. Cook, J. L. Waddle, *Tetrahedron Letters* **2003**, *44*, 6923–6925.

[10] C. K. Jadhav, A. S. Nipate, A. V. Chate, A. P. Patil, Charansingh. H. Gill, *J Heterocyclic Chem* **2020**, *57*, 4291–4303.

[11] S. P. Chavan, K. Shivsankar, K. Pasupathy, *Synthesis* **2005**, 1297–1300.

[12] J. L. Zigterman, J. C. S. Woo, S. D. Walker, J. S. Tedrow, C. J. Borths, E. E. Bunel, M. M. Faul, *J. Org. Chem.* **2007**, *72*, 8870–8876.

[13] S. Omura, M. Katagiri, J. Awaya, T. Furukawa, I. Umezawa, N. Oi, M. Mizoguchi, B. Aoki, M. Shindo, *Antimicrobial Agents and Chemotherapy* **1974**, *6*, 207–215.

[14] W. Fang, B. Breit, *Angew. Chem. Int. Ed.* **2018**, *57*, 14817–14821.

[15] B. Das, A. Kashinatham, B. Venkataiah, *Synthetic Communications* **1999**, *29*, 3799–3804.

4. NMR-Spectras

4.1 Amides and amines

(*E*)-1-(Pyrrolidin-1-yl)-pent-3-en-1-one (1a)

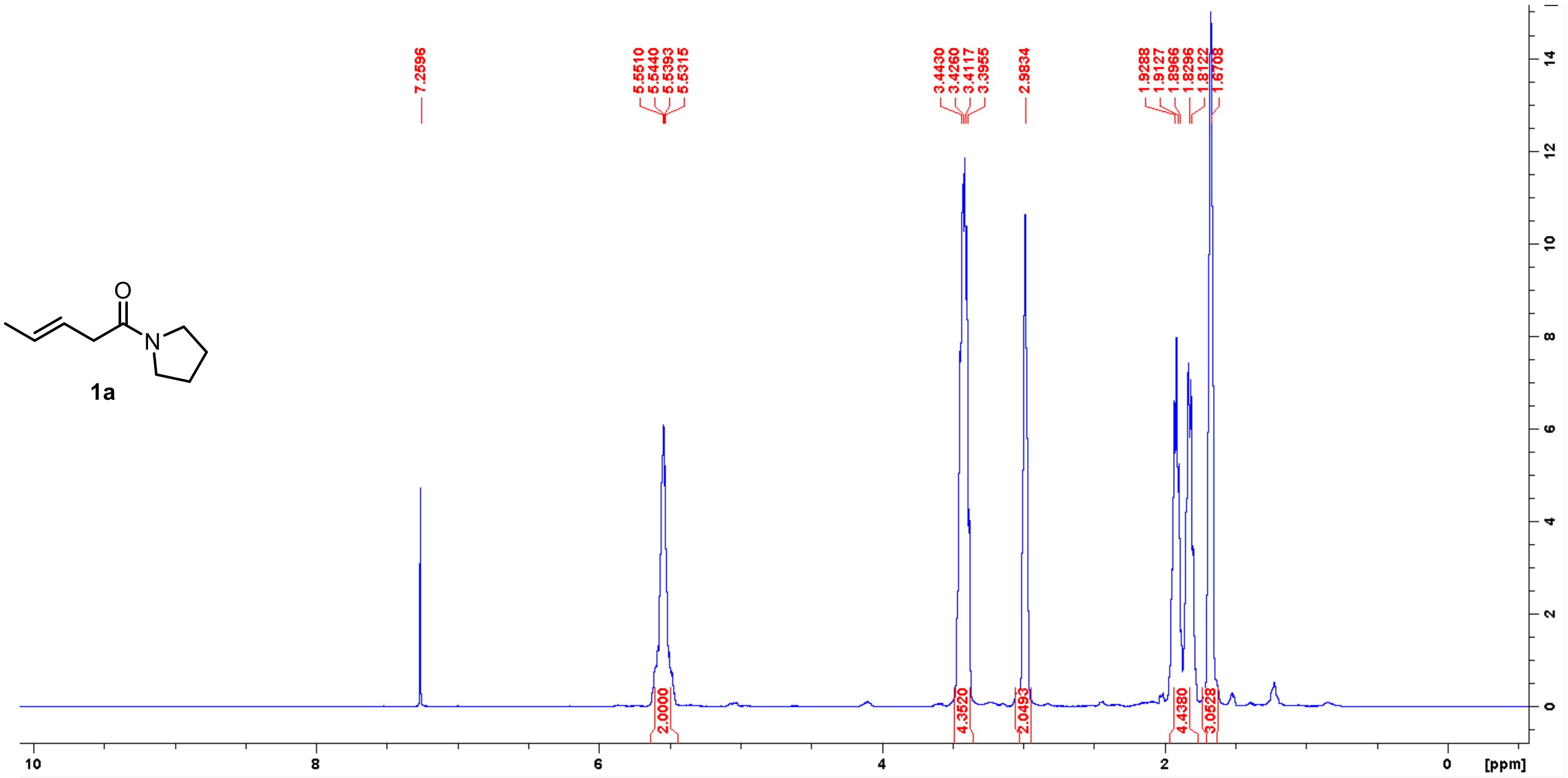

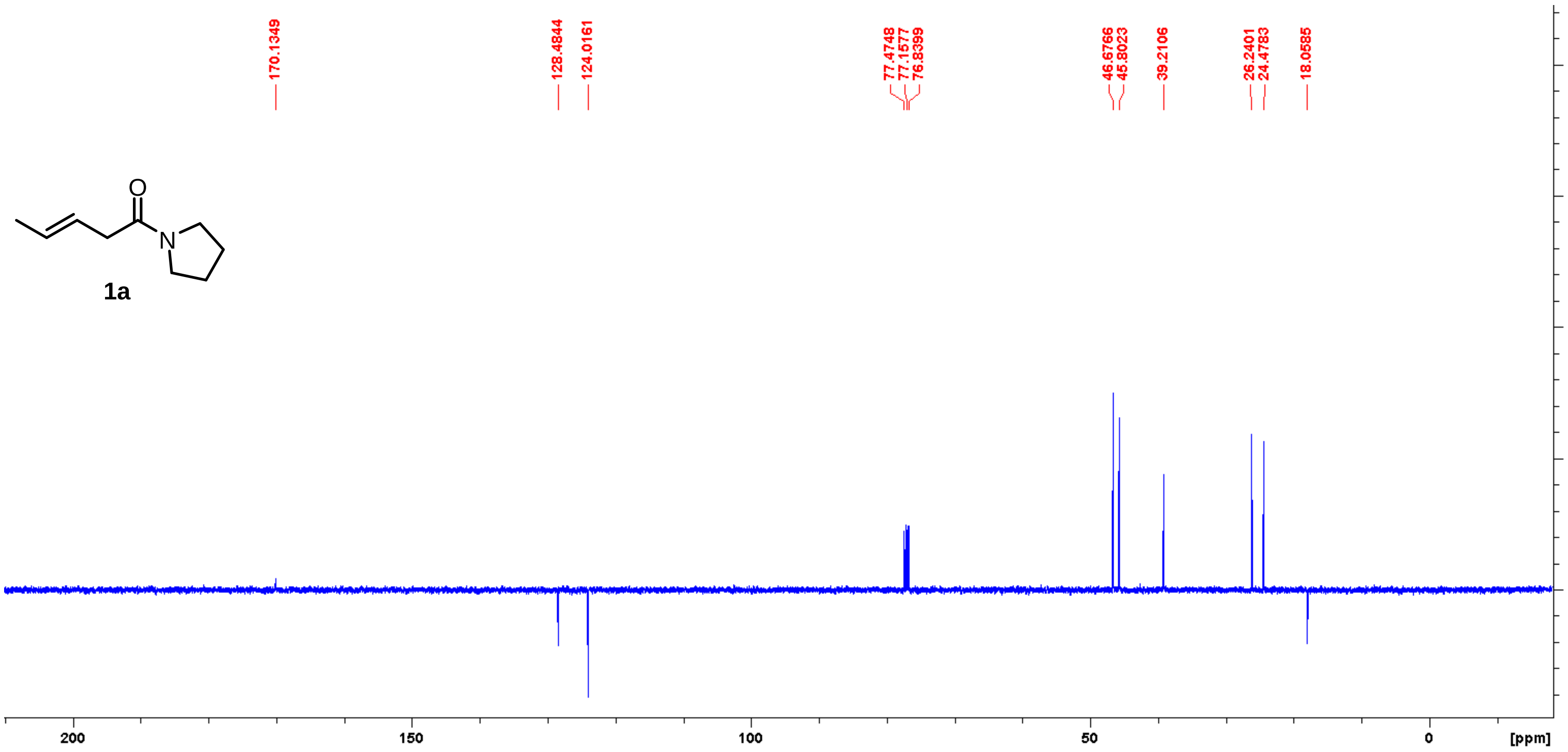

(*E*)-1-(Pyrrolidin-1-yl)-tridec-3-en-1-one (1b)

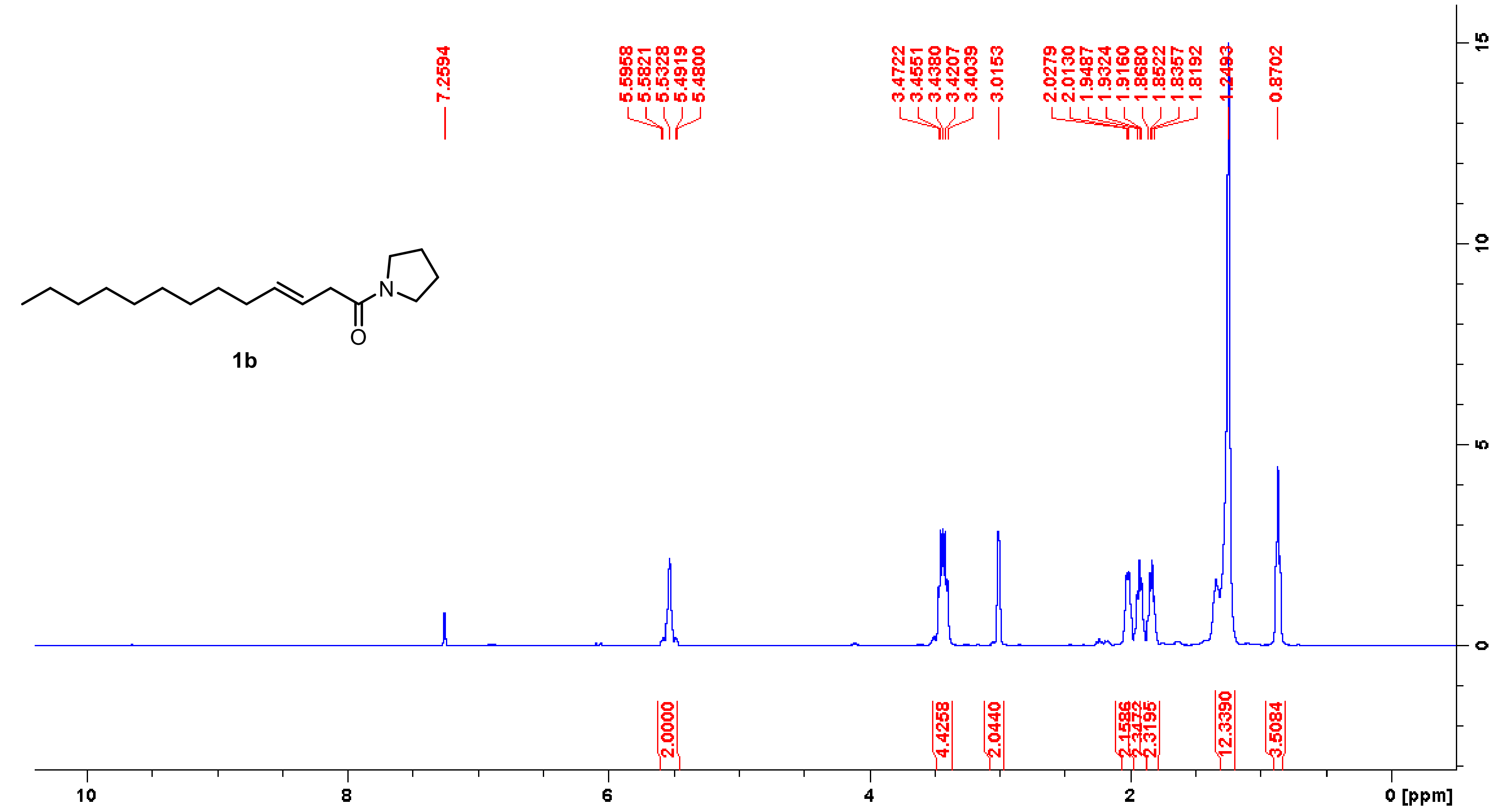

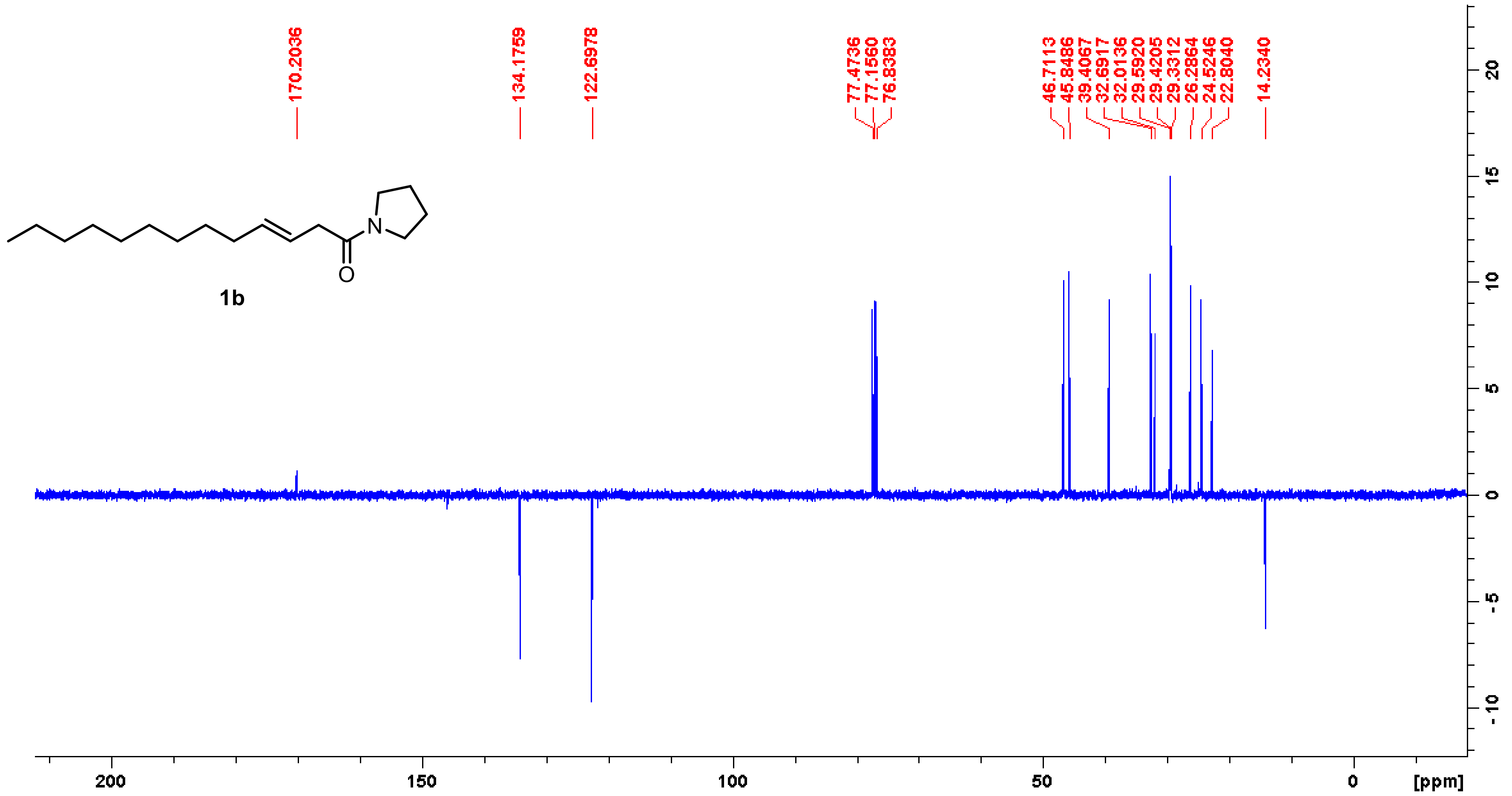

(*E*)-5-Phenyl-1-(pyrrolidin-1-yl)-pent-3-en-1-one (1c)

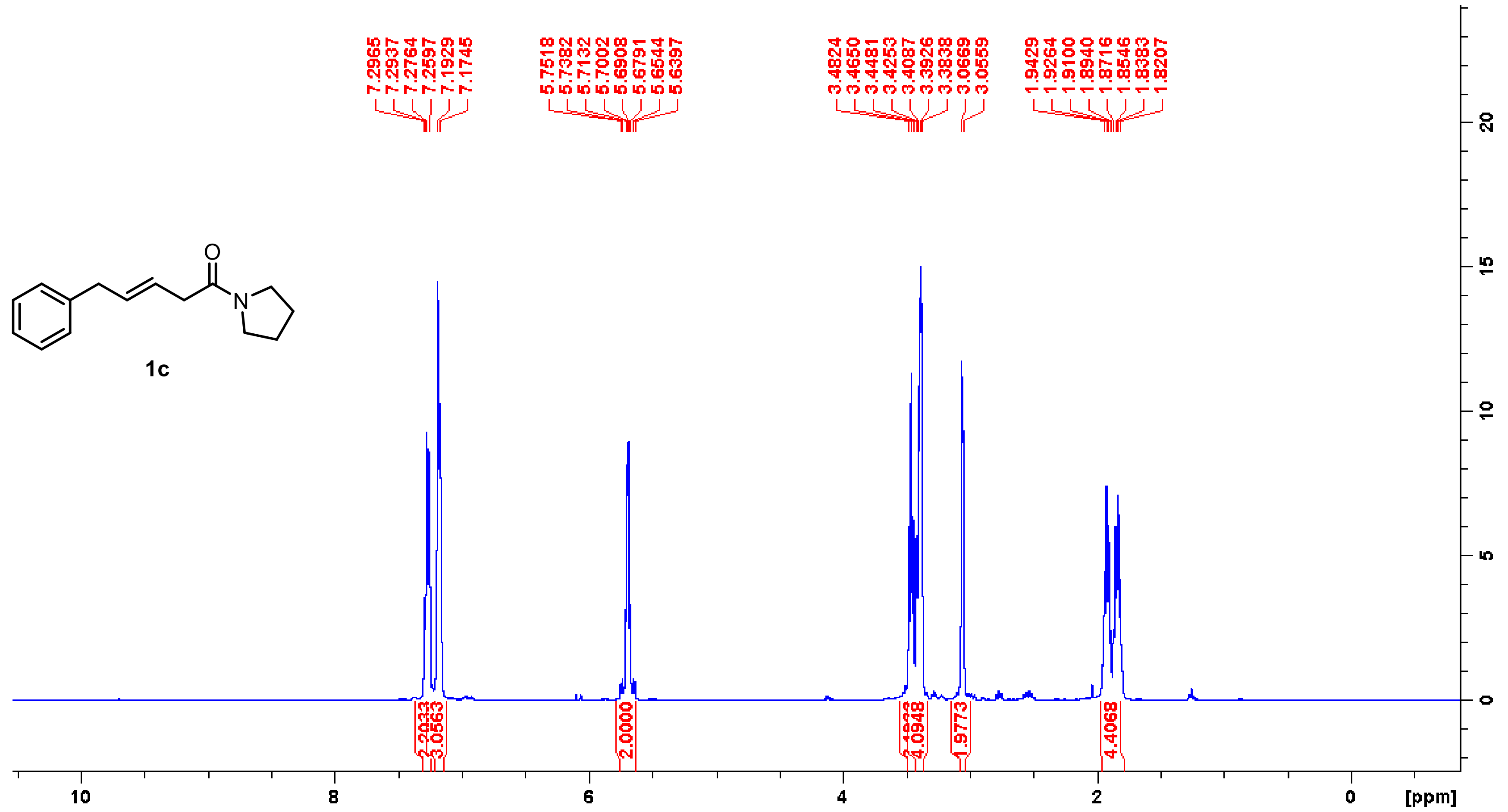

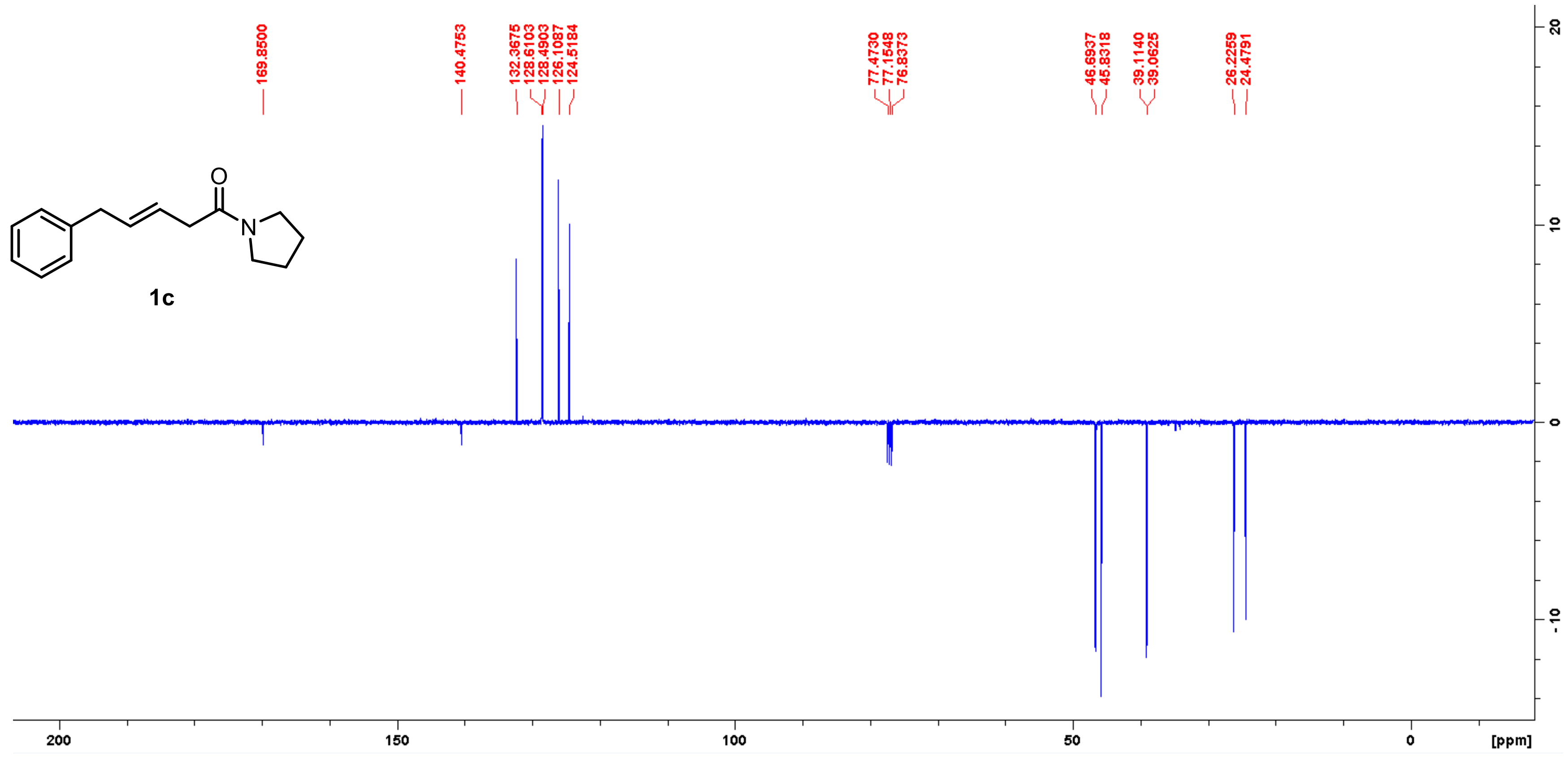

1-(Pyrrolidin-1-yl)but-3-en-1-one (1d)

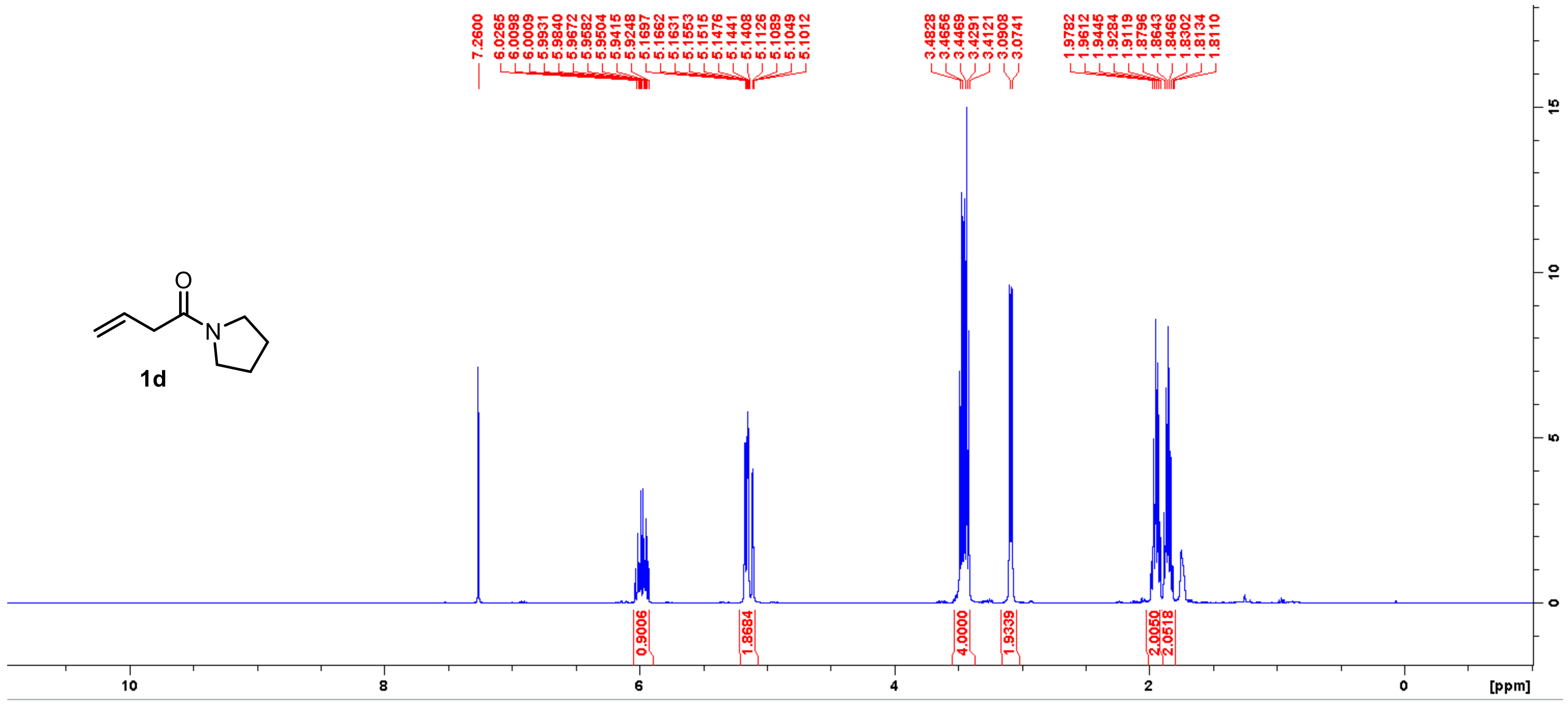

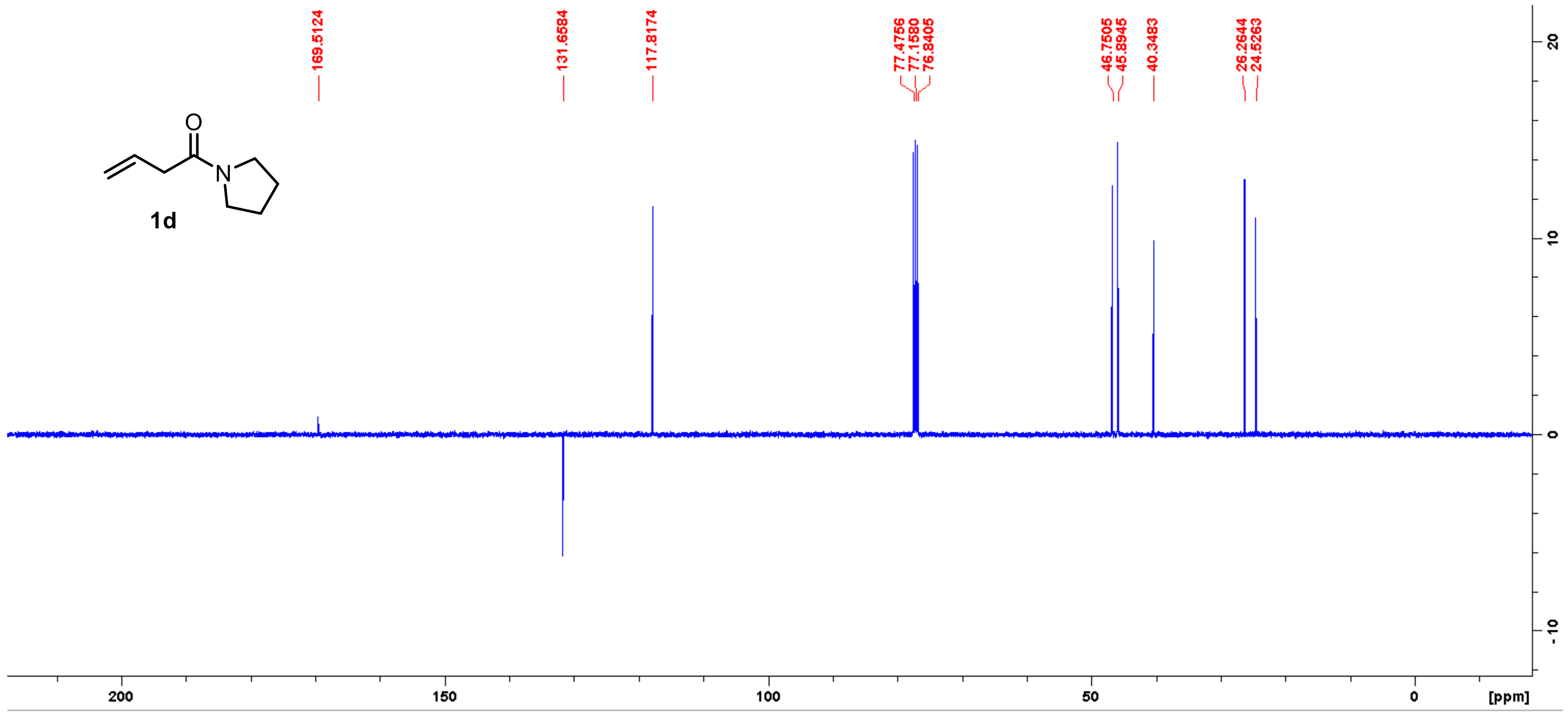

1-(Pyrrolidin-1-yl)-penta-3,4-dien-1-one (1e)

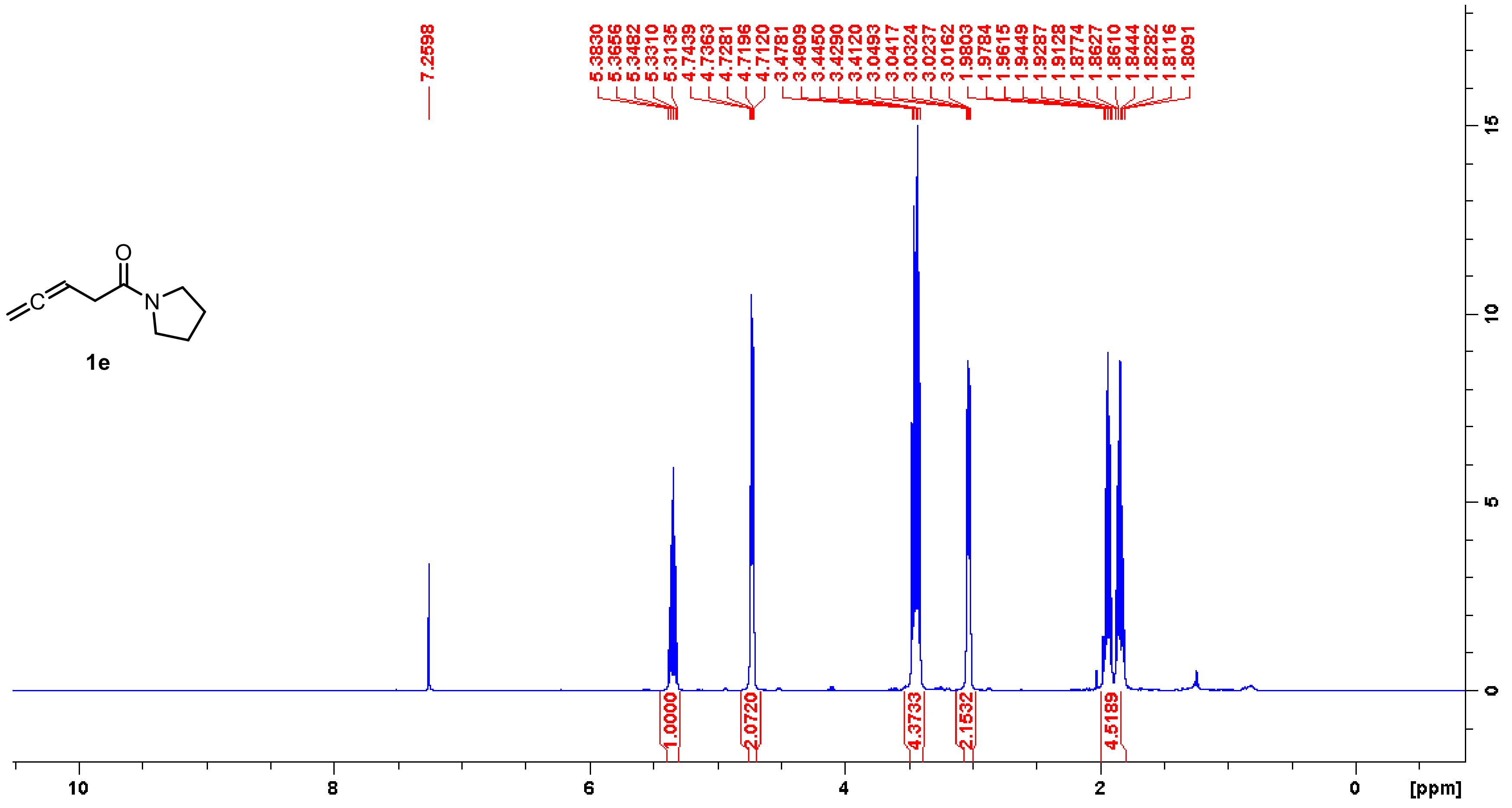

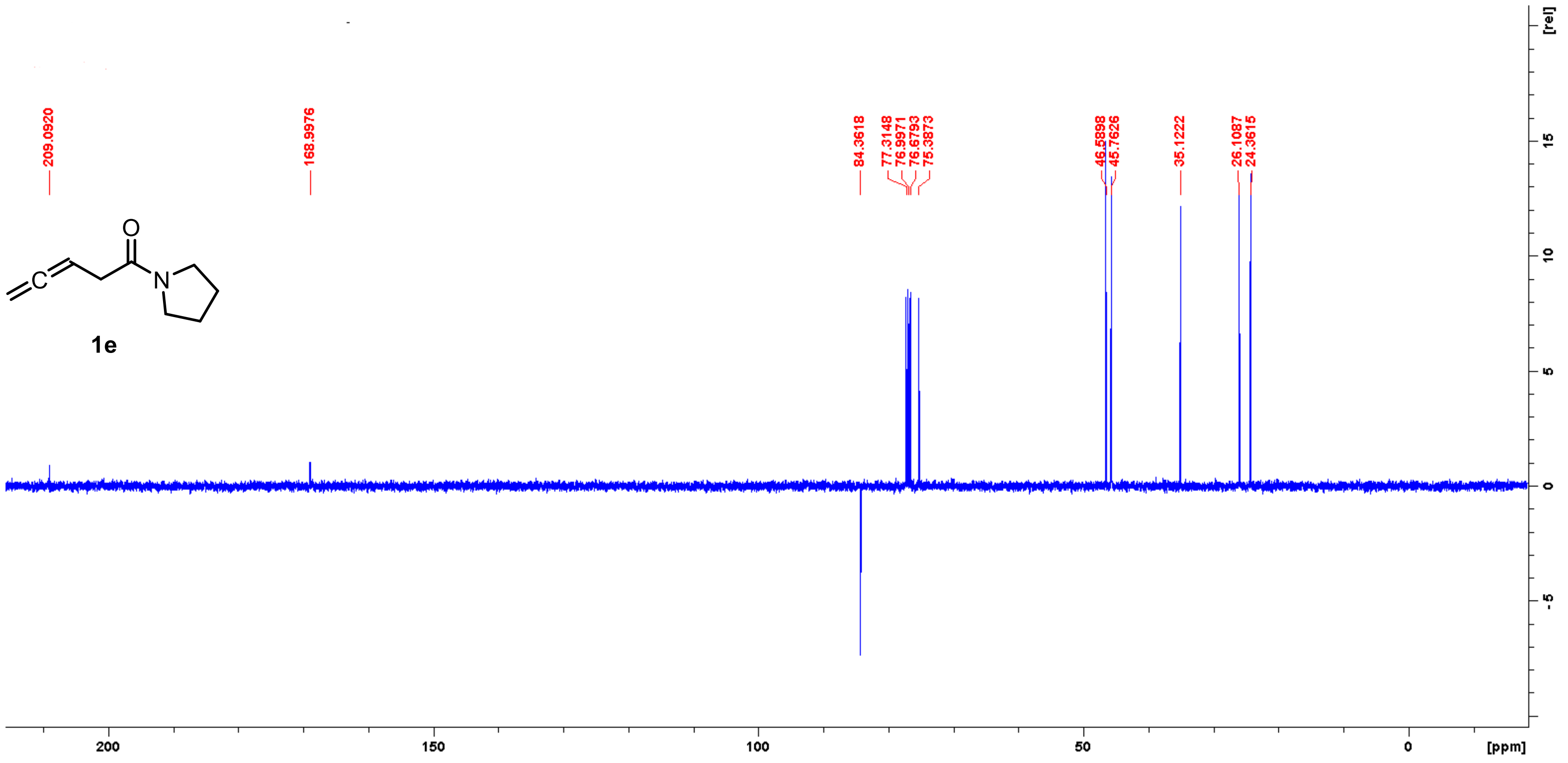

(*E*)-2-Methyl-1-(pyrrolidin-1-yl)-pent-3-en-1-one (1f)

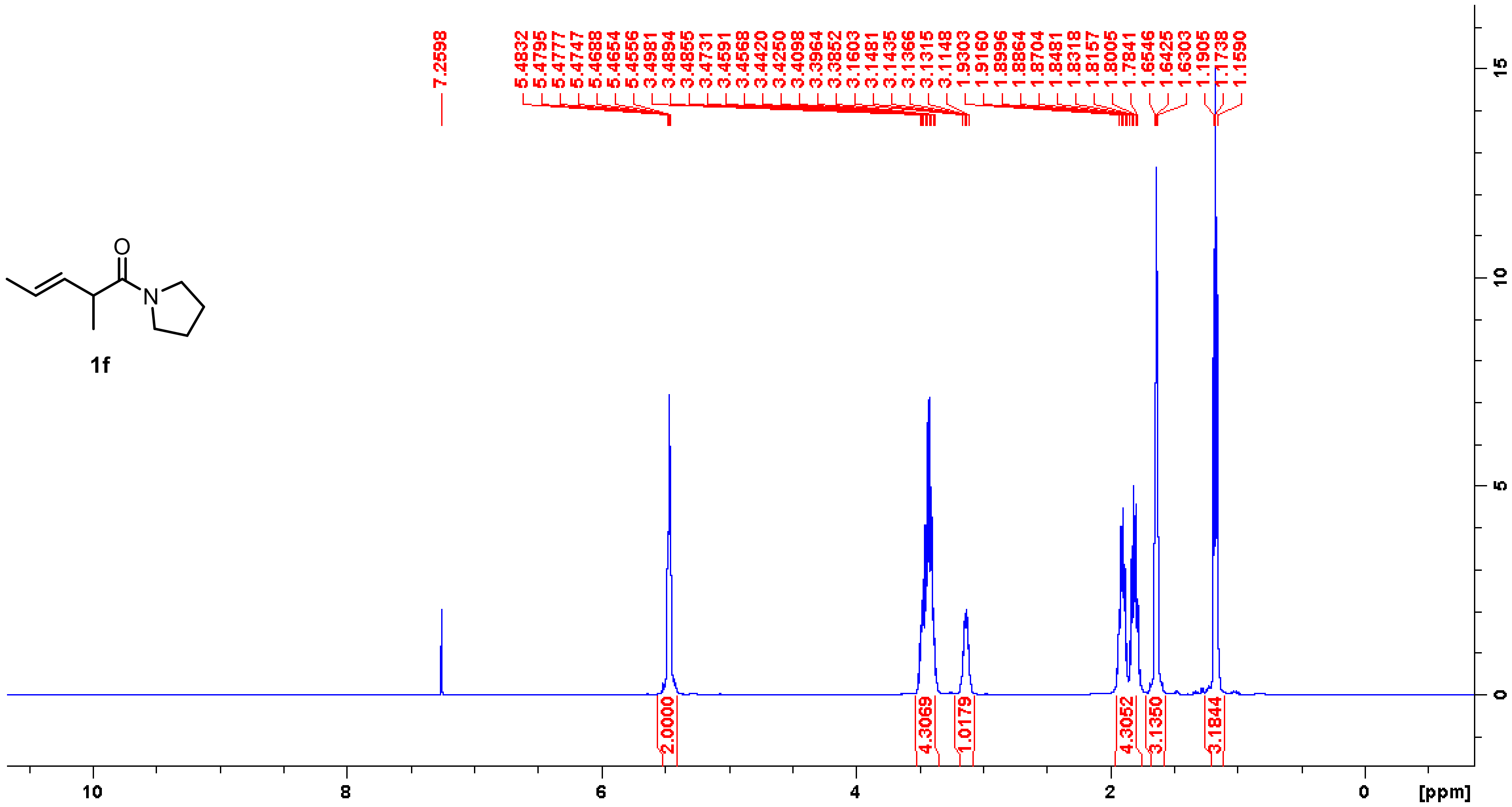

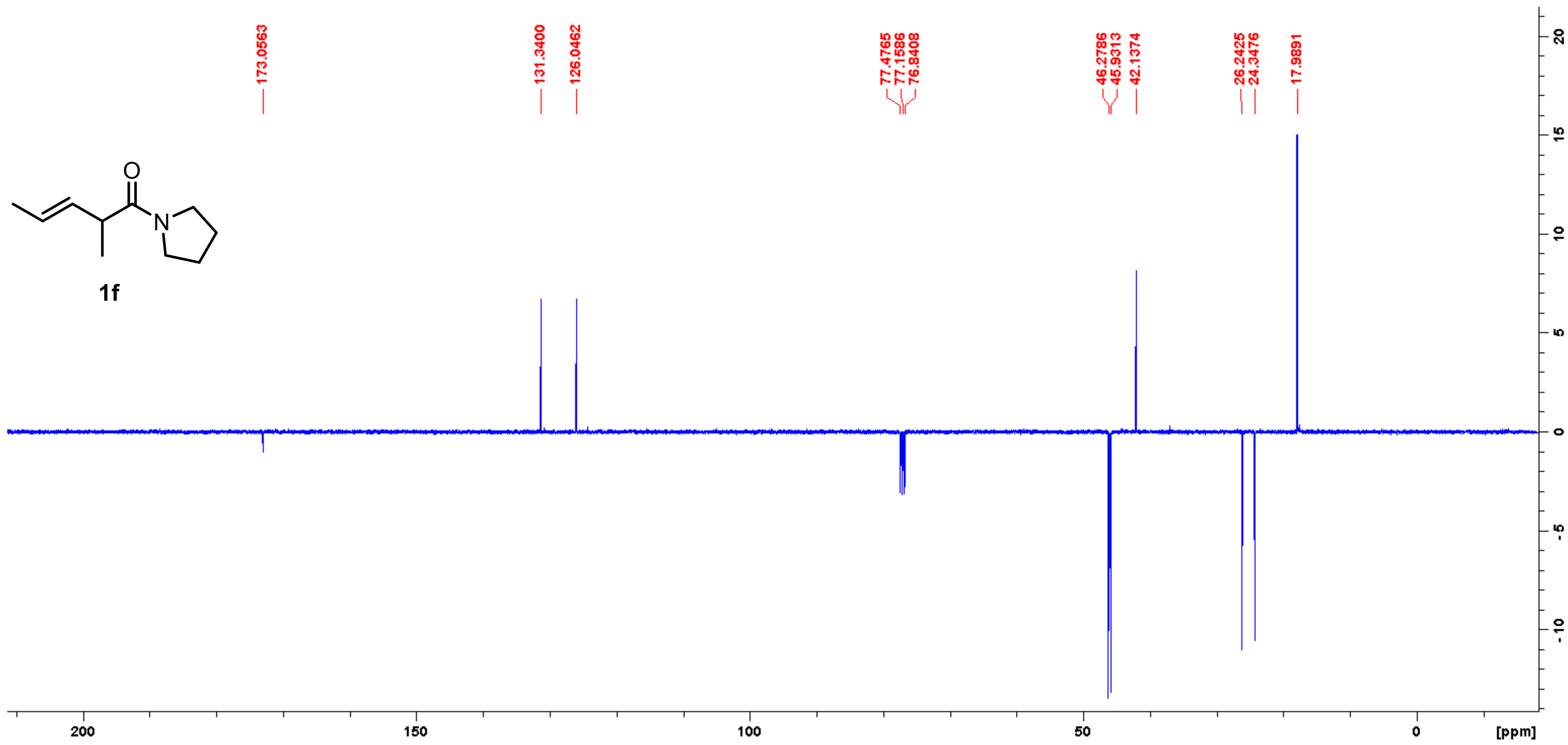

(*E*)-*N*-cyclopropyl-*N*,2-dimethylpent-3-enamide (1g)

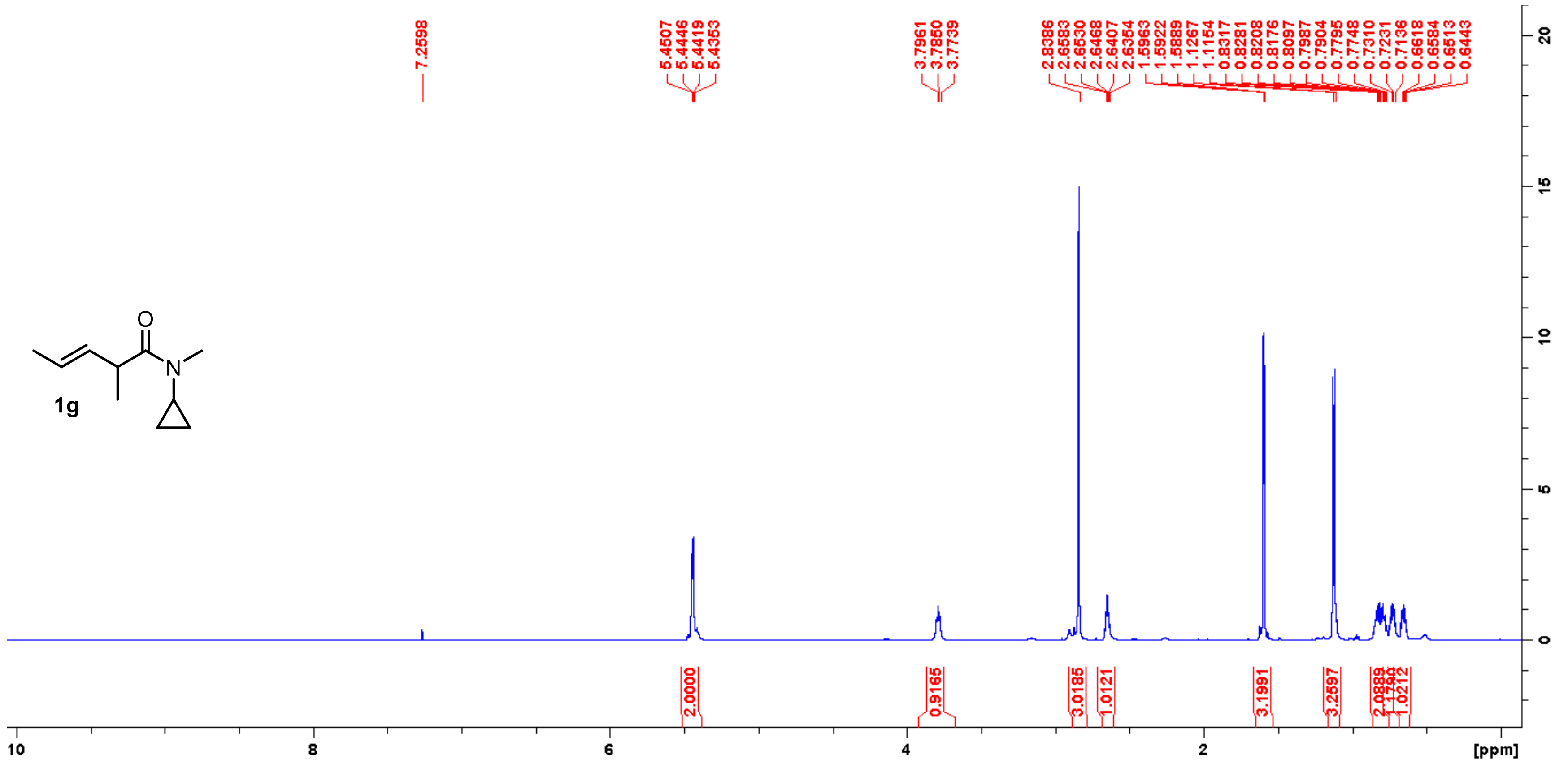

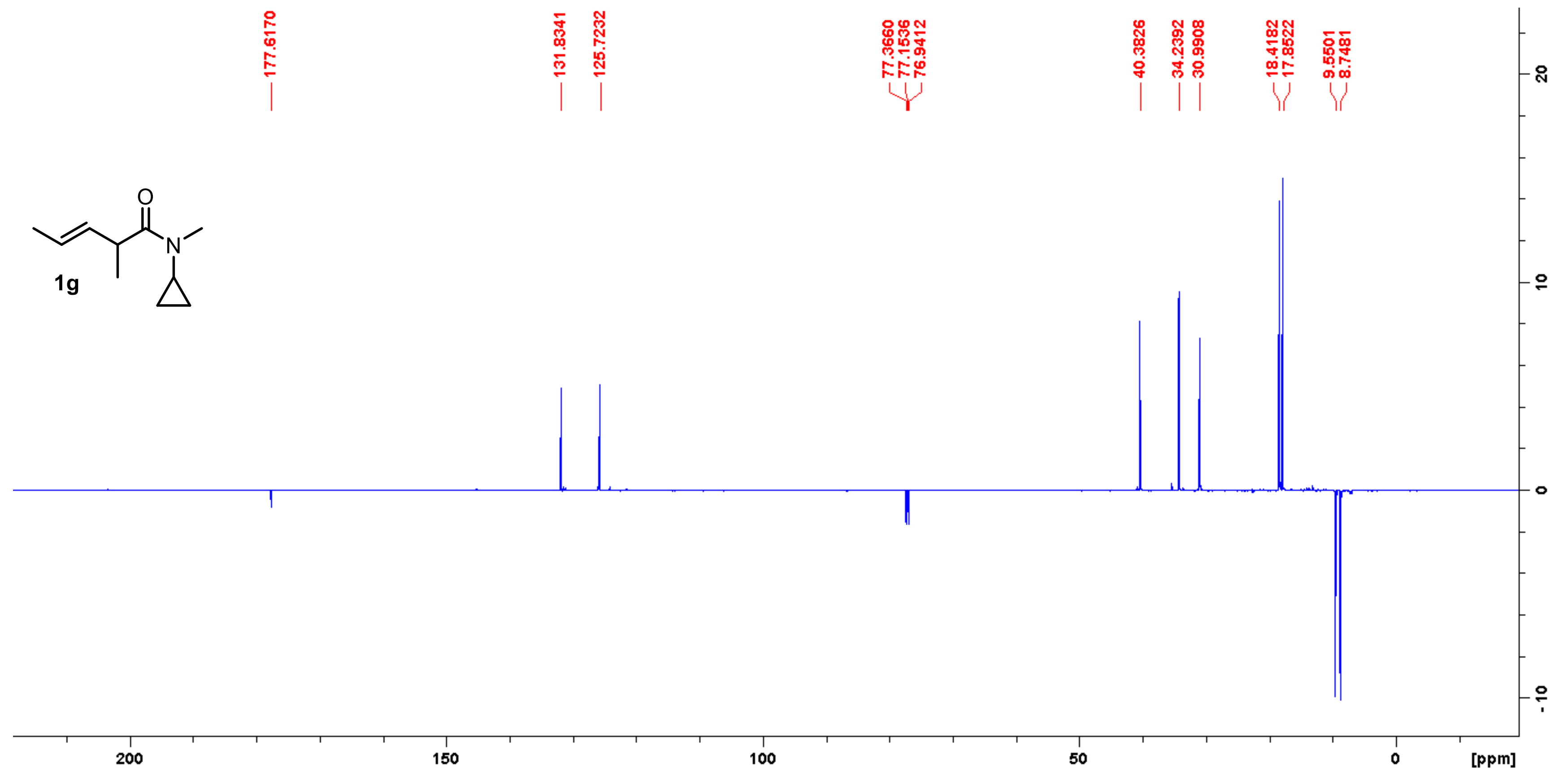

(Cyclohex-1-en-1-yl)-1-(pyrrolidin-1-yl)-ethan-1-one (1h)

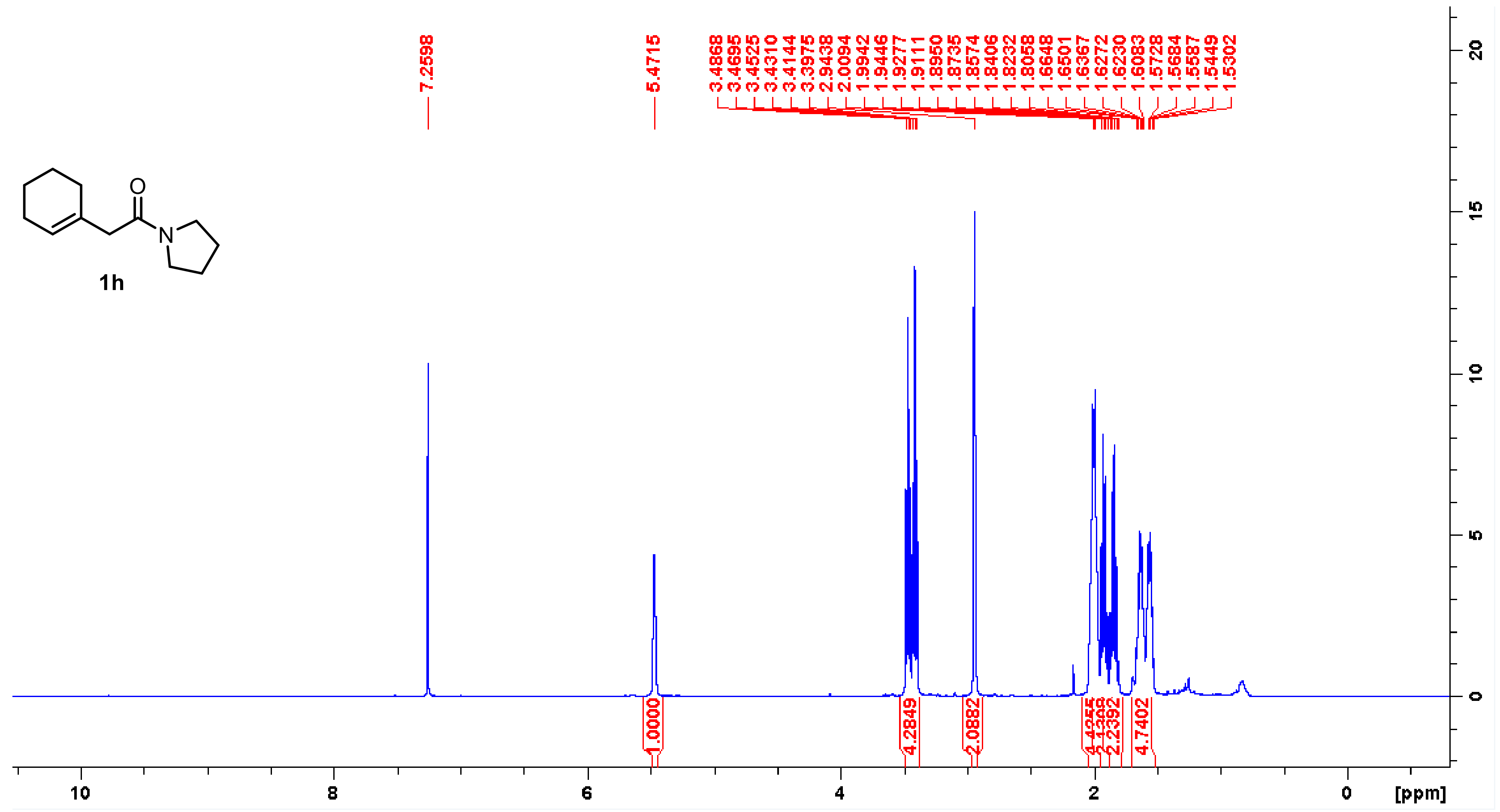

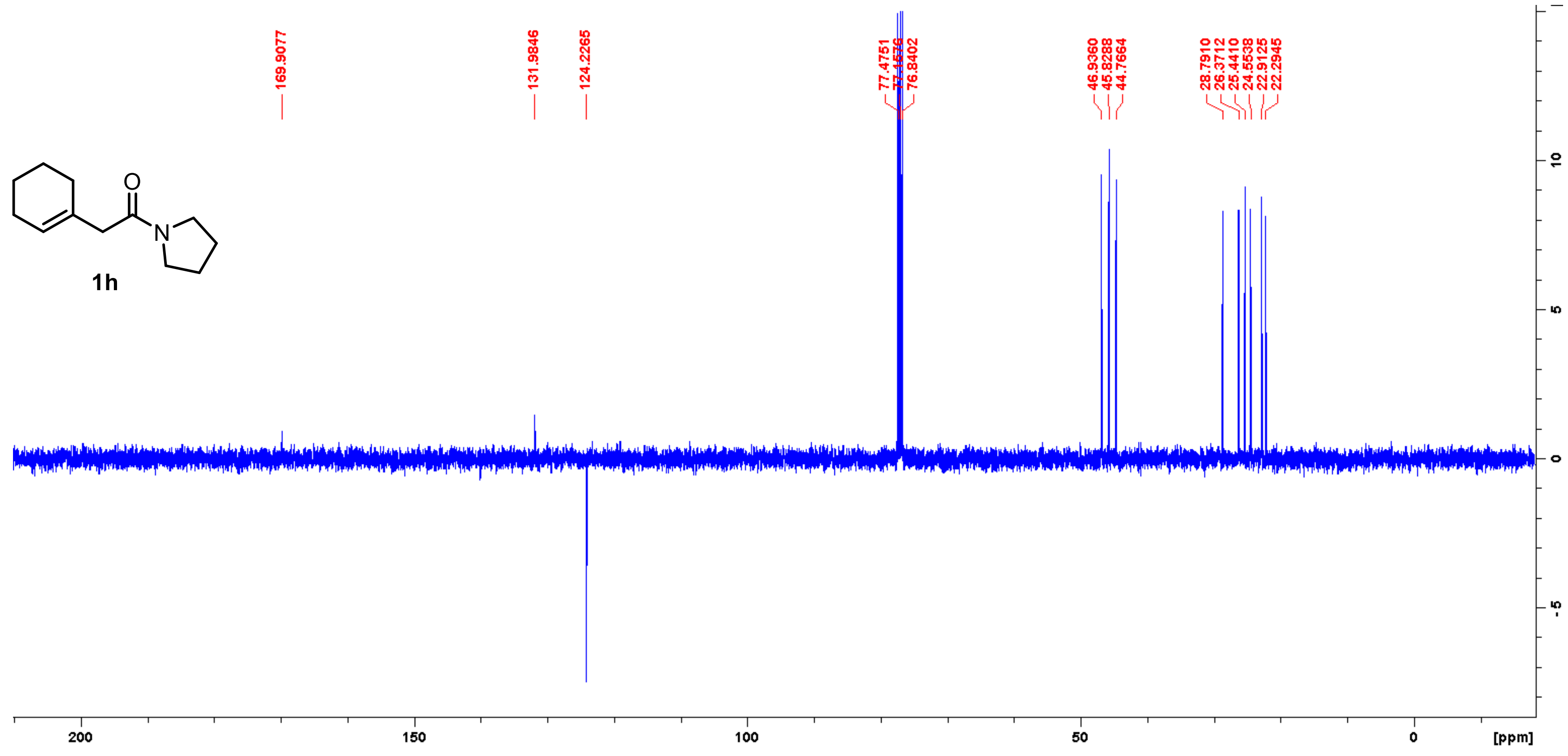

Methyl (*E*)-12-oxo-12-(pyrrolidin-1-yl)-dodec-9-enoate (1i)

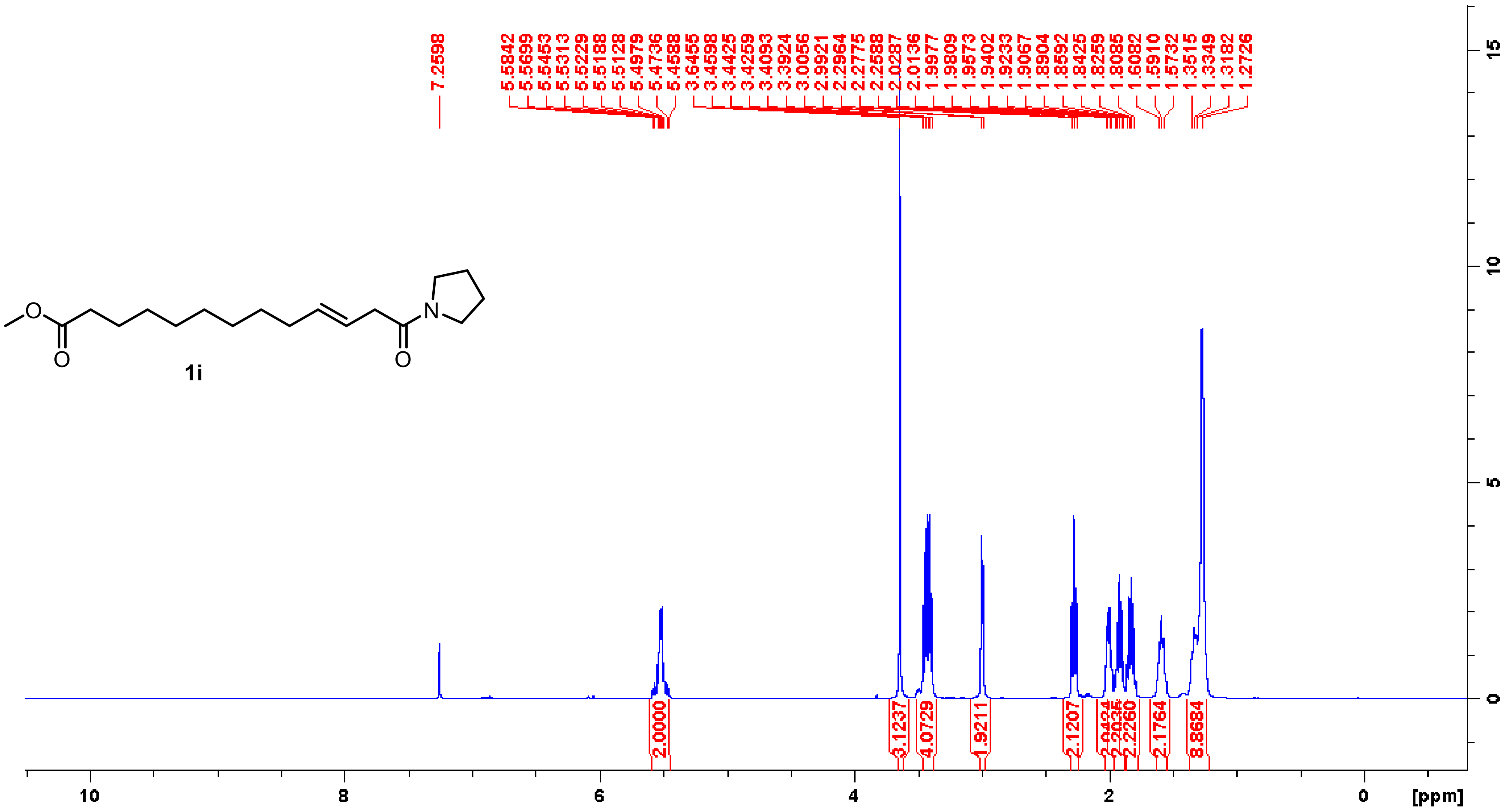

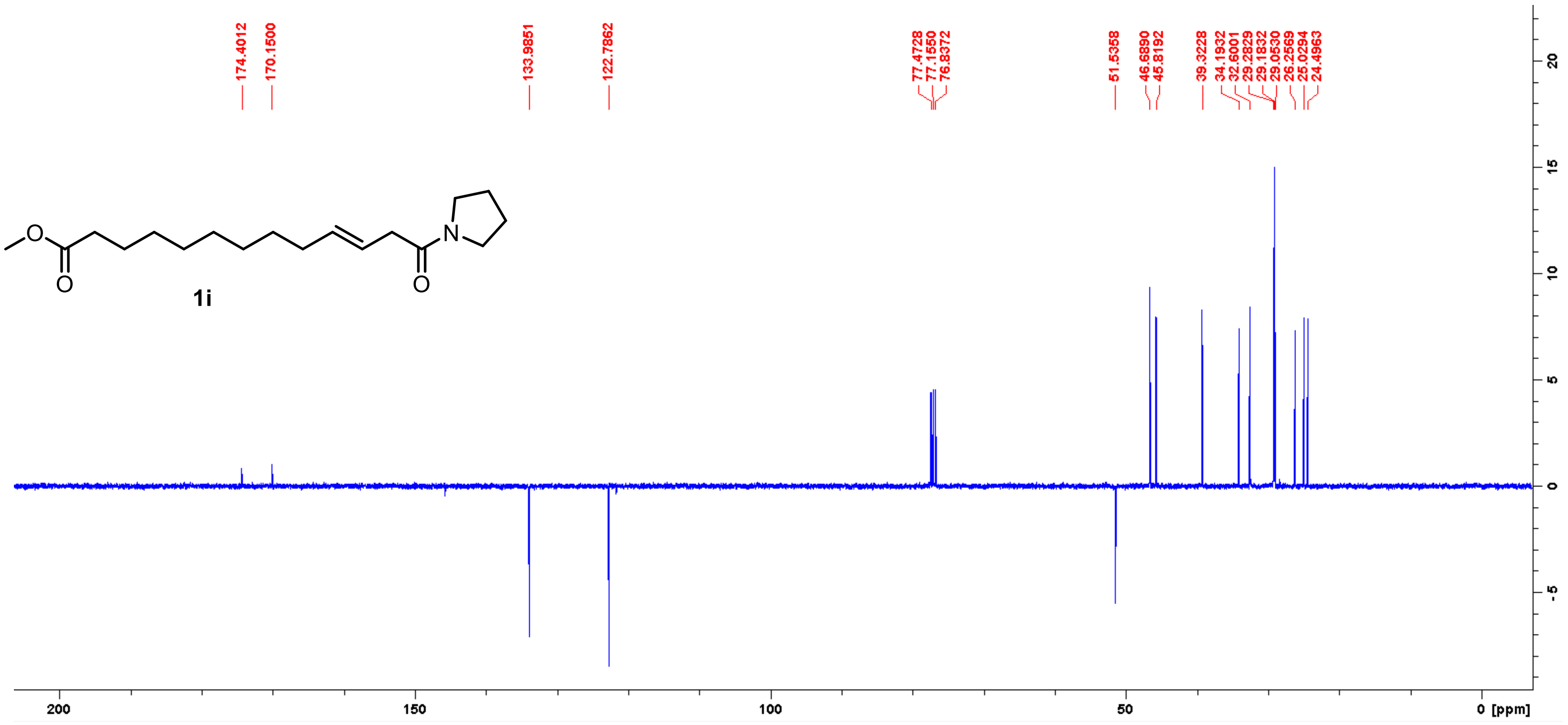

(*E*)-1-(Pyrrolidin-1-yl)dodec-3-ene-1,11-dione (1j)

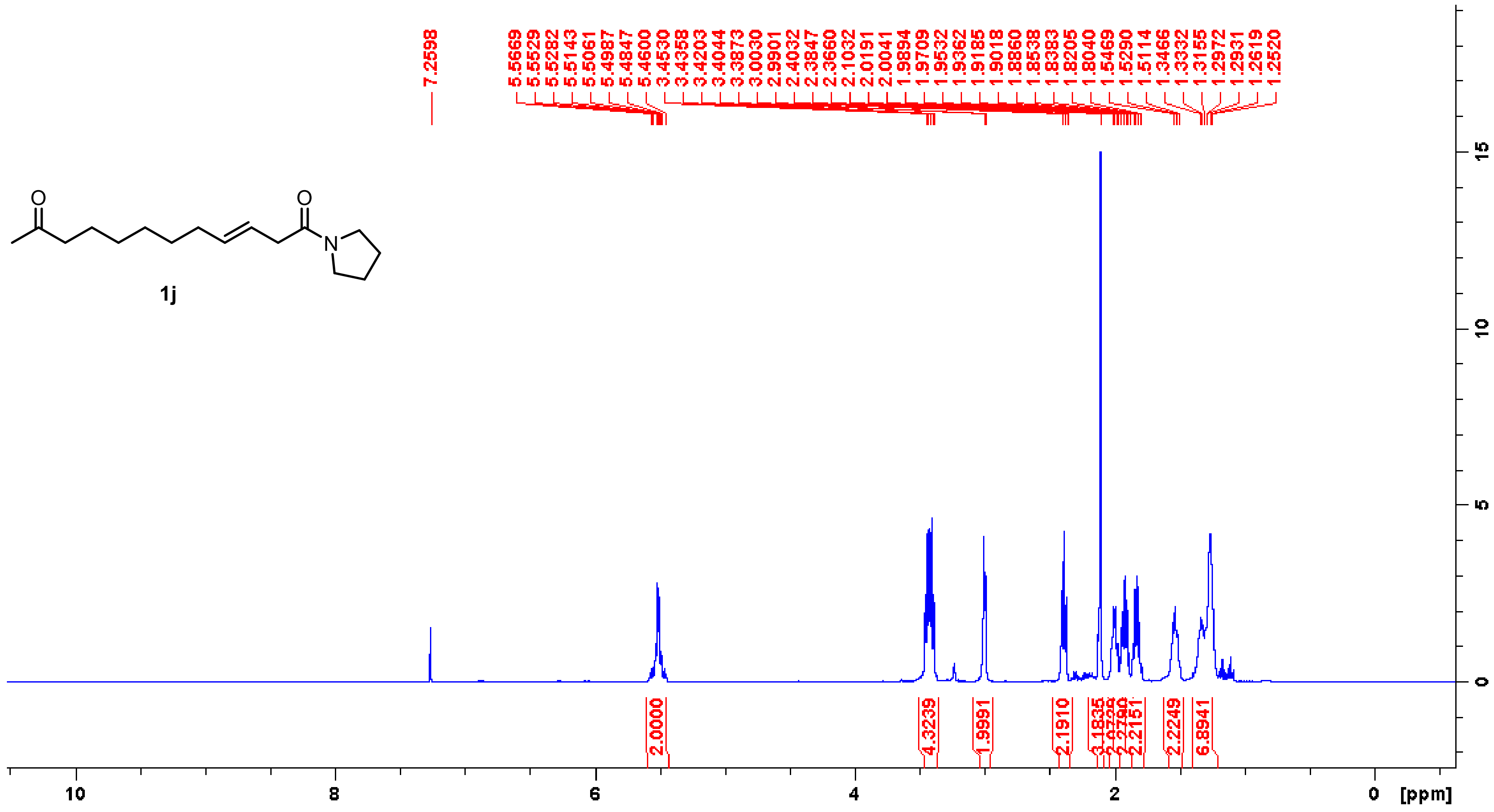

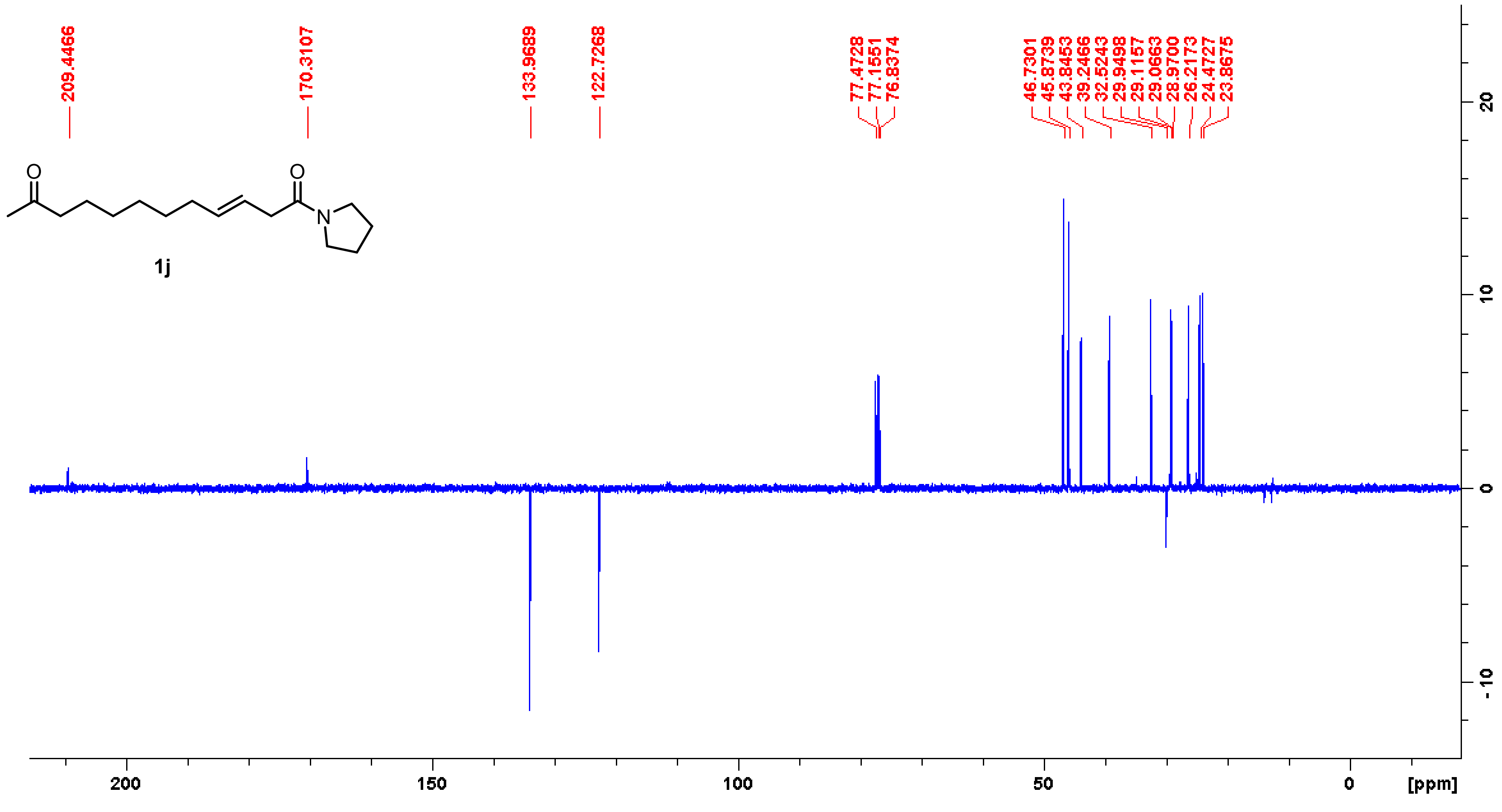

(*E*)-9-Oxo-9-(pyrrolidin-1-yl)-non-6-enenitrile (1k)

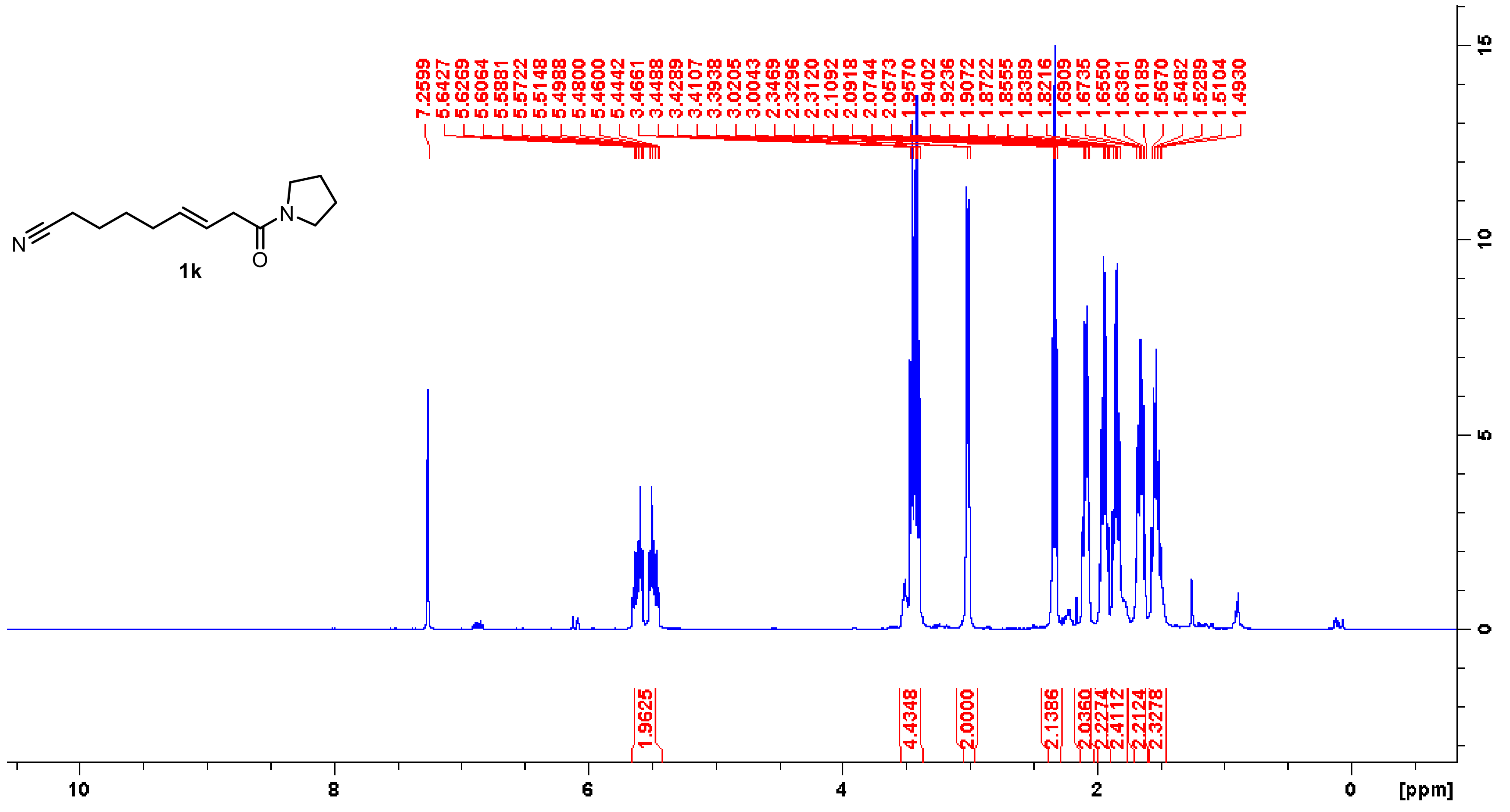

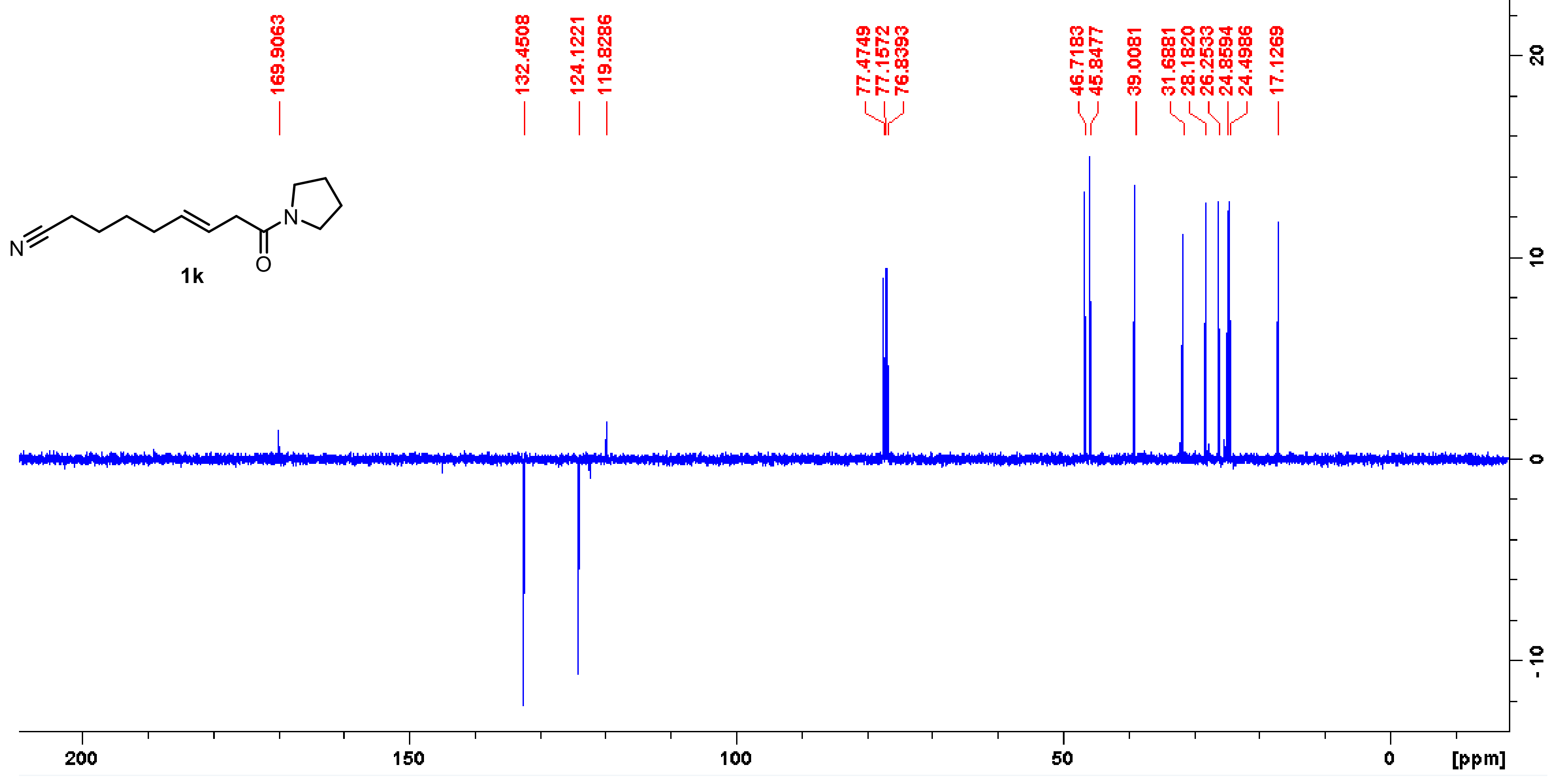

(*E*)-8-Chloro-1-(pyrrolidin-1-yl)-oct-3-en-1-one (1l)

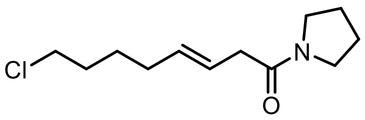

1l

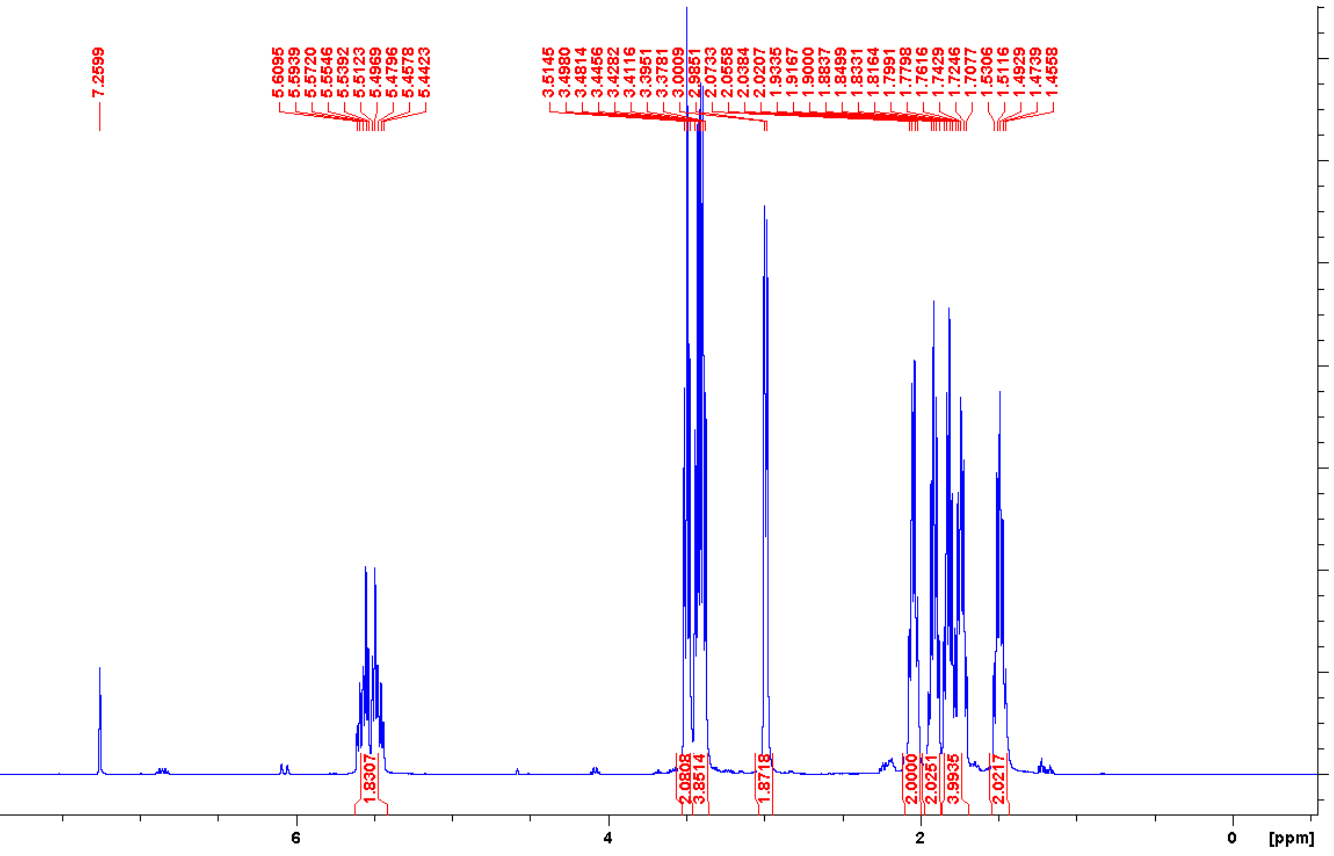

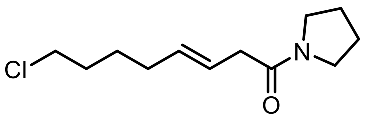

1l

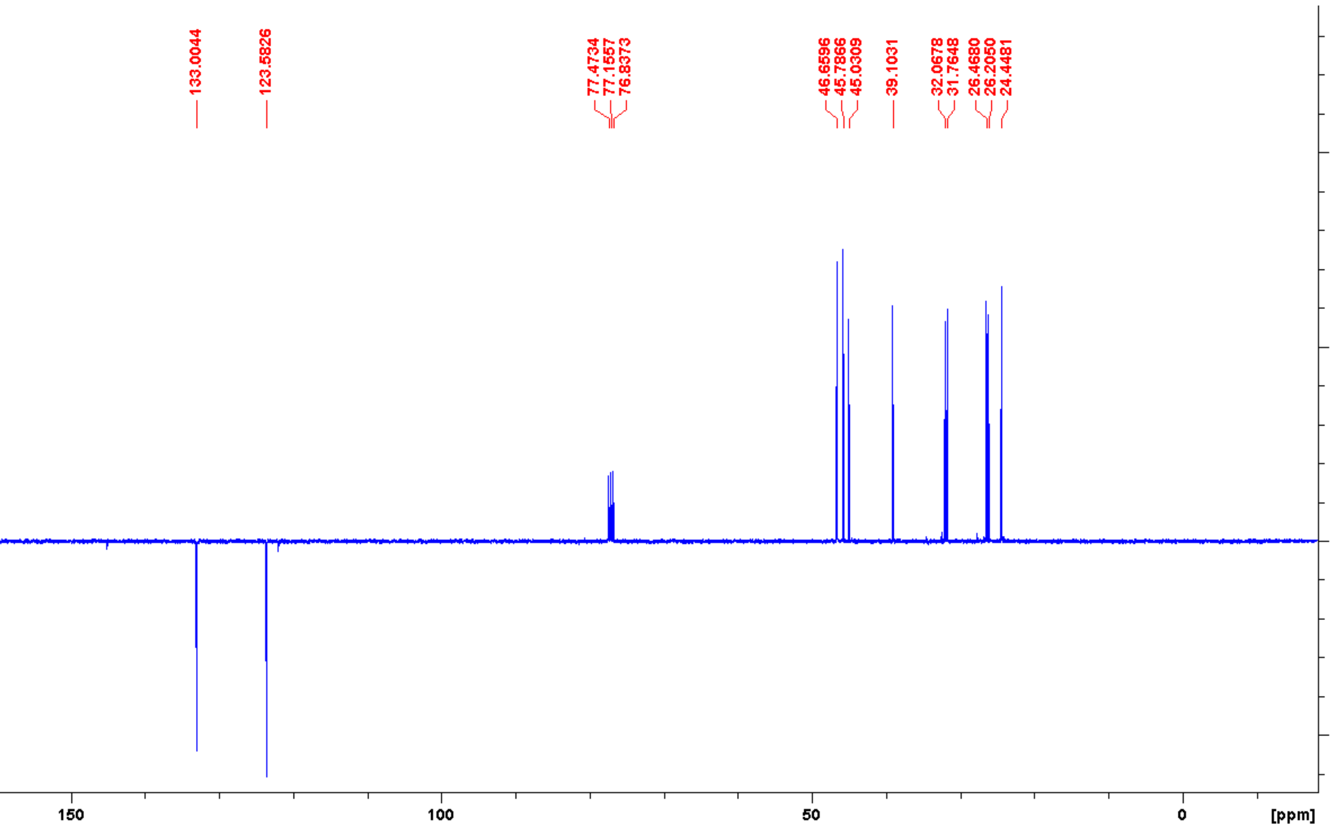

(*E*)-1-(Pyrrolidin-1-yl)-dodec-3-en-11-yn-1-one (1m)

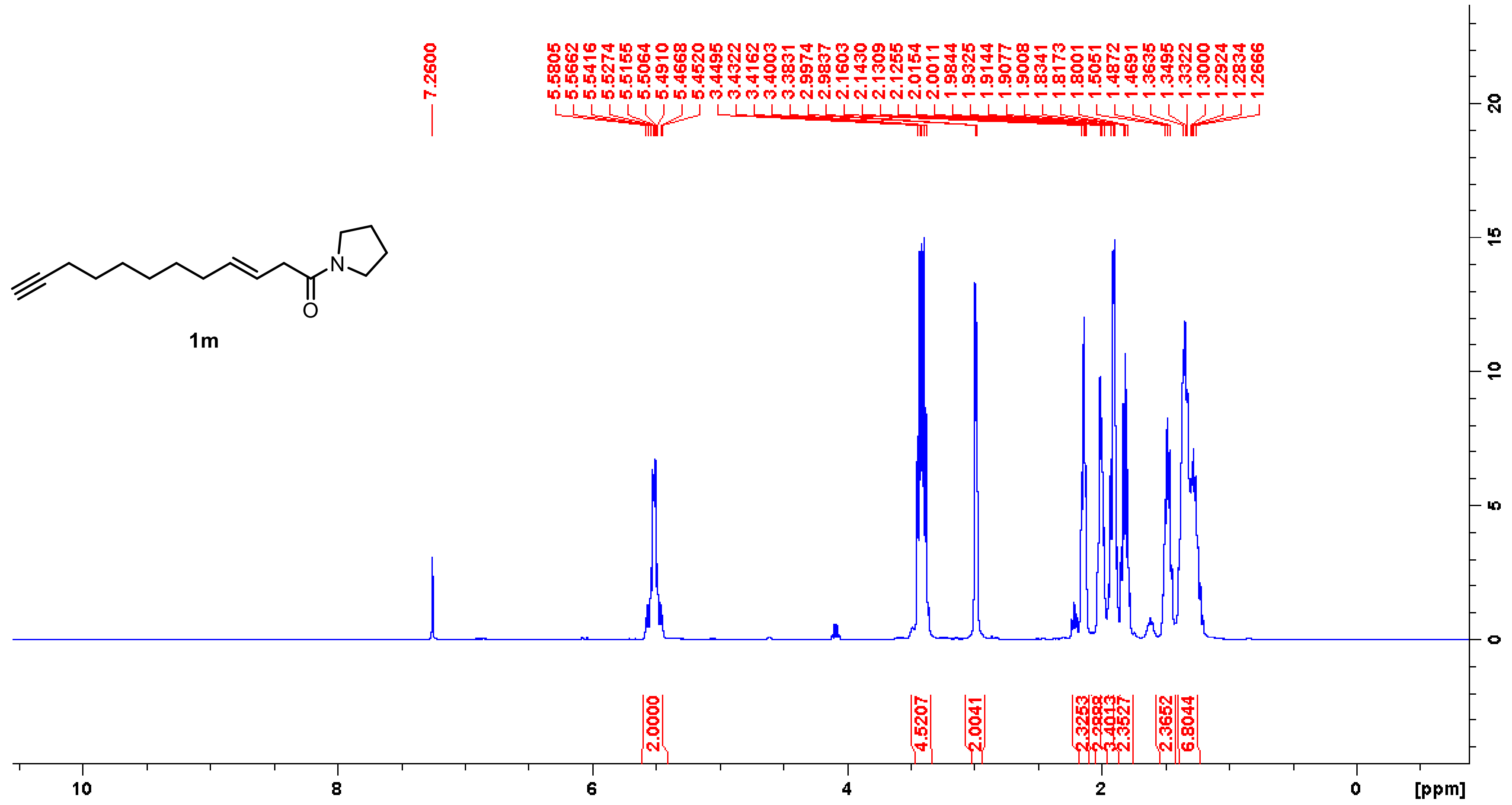

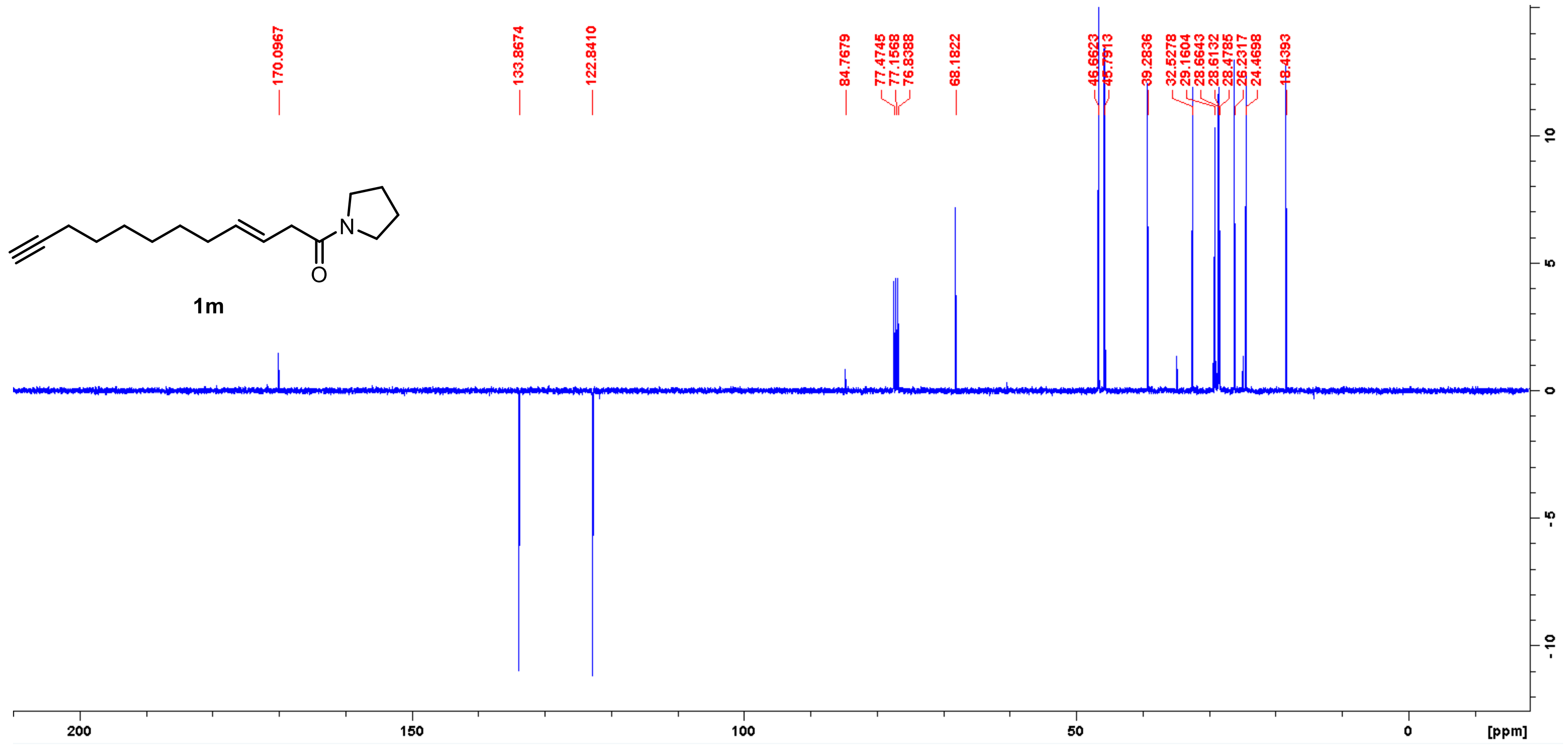

(*R*,*E*)-5,9-Dimethyl-1-(pyrrolidin-1-yl)-deca-3,8-dien-1-one (1n)

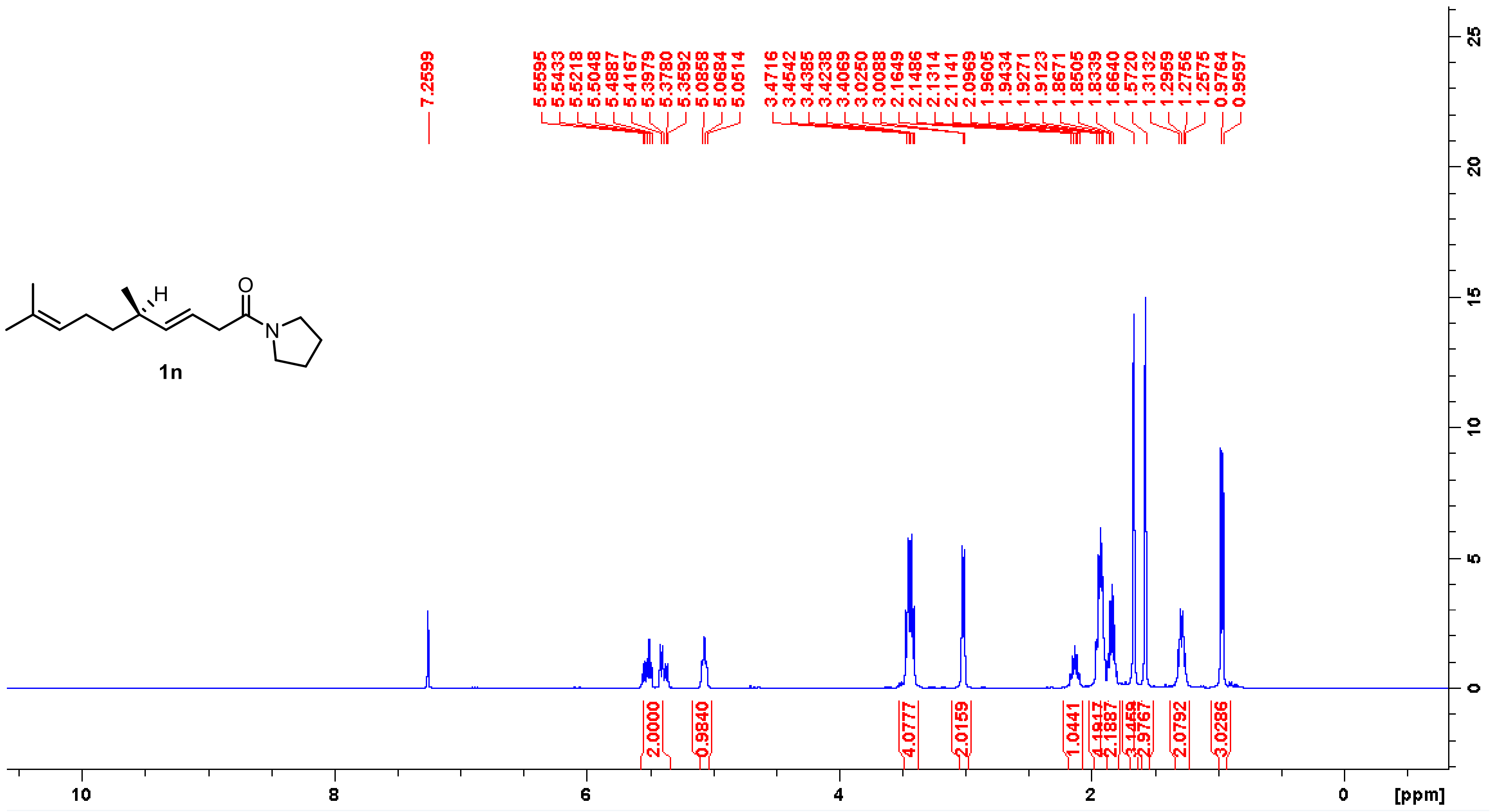

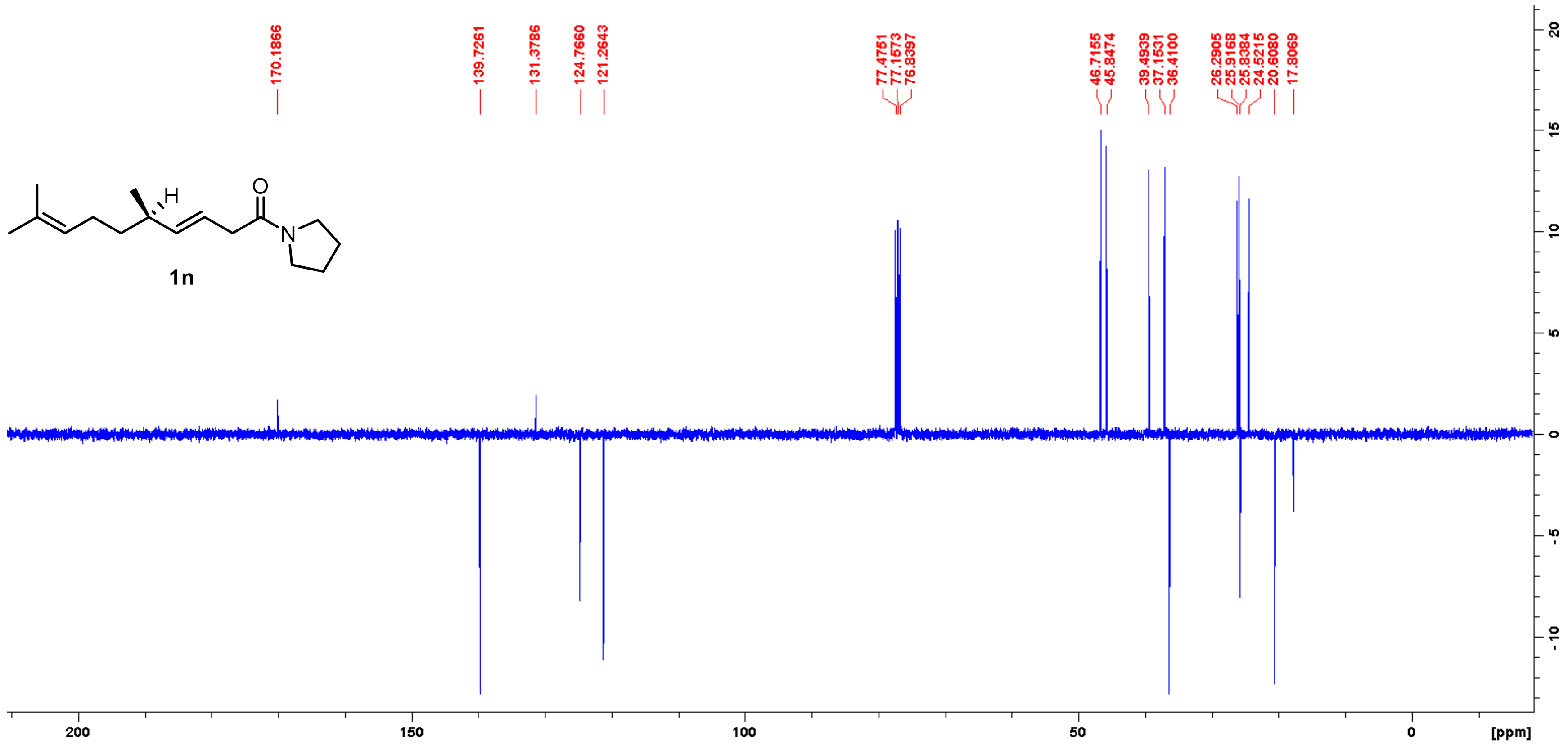

(*E*)-*N*-Butyldodec-3-enamide (1o)

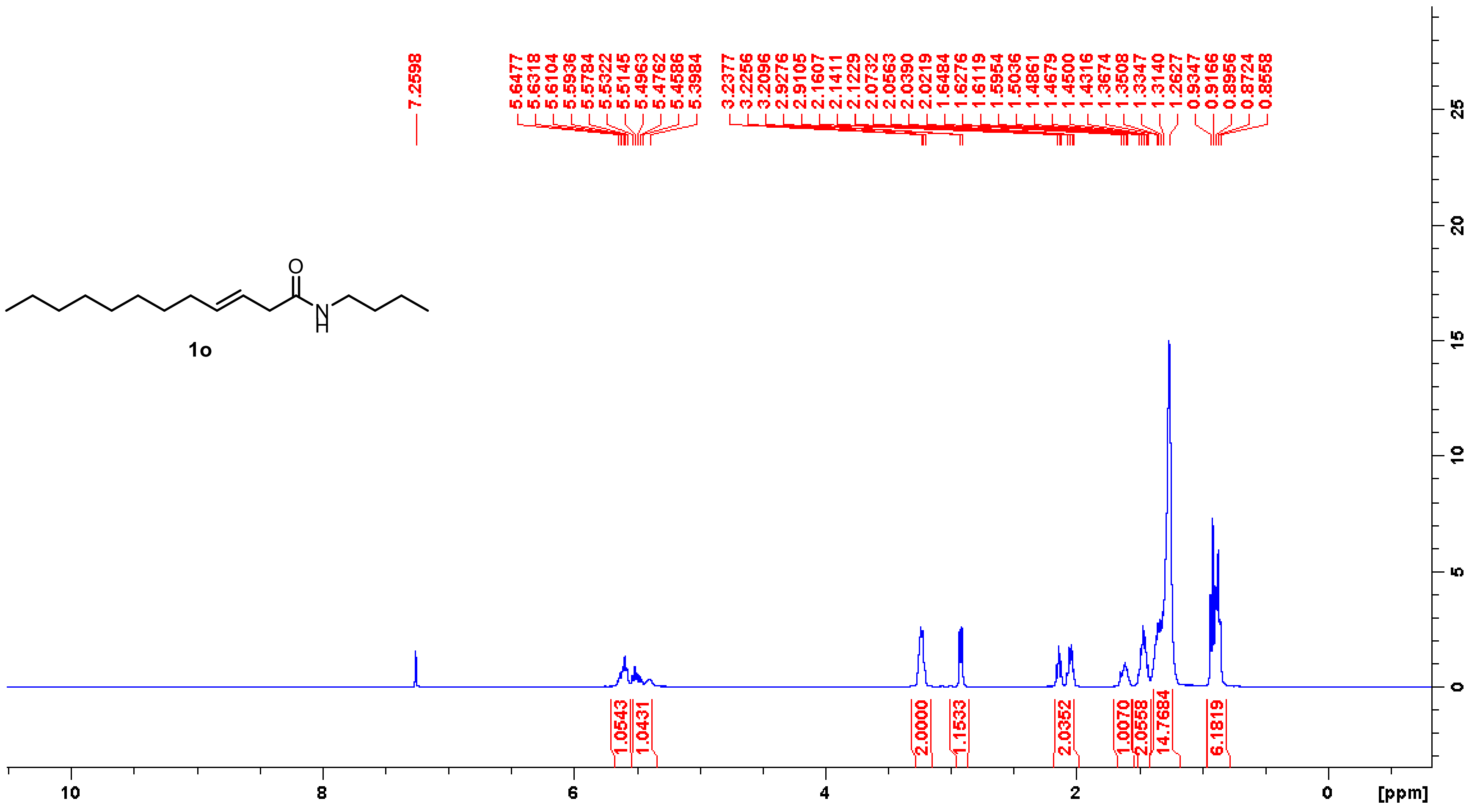

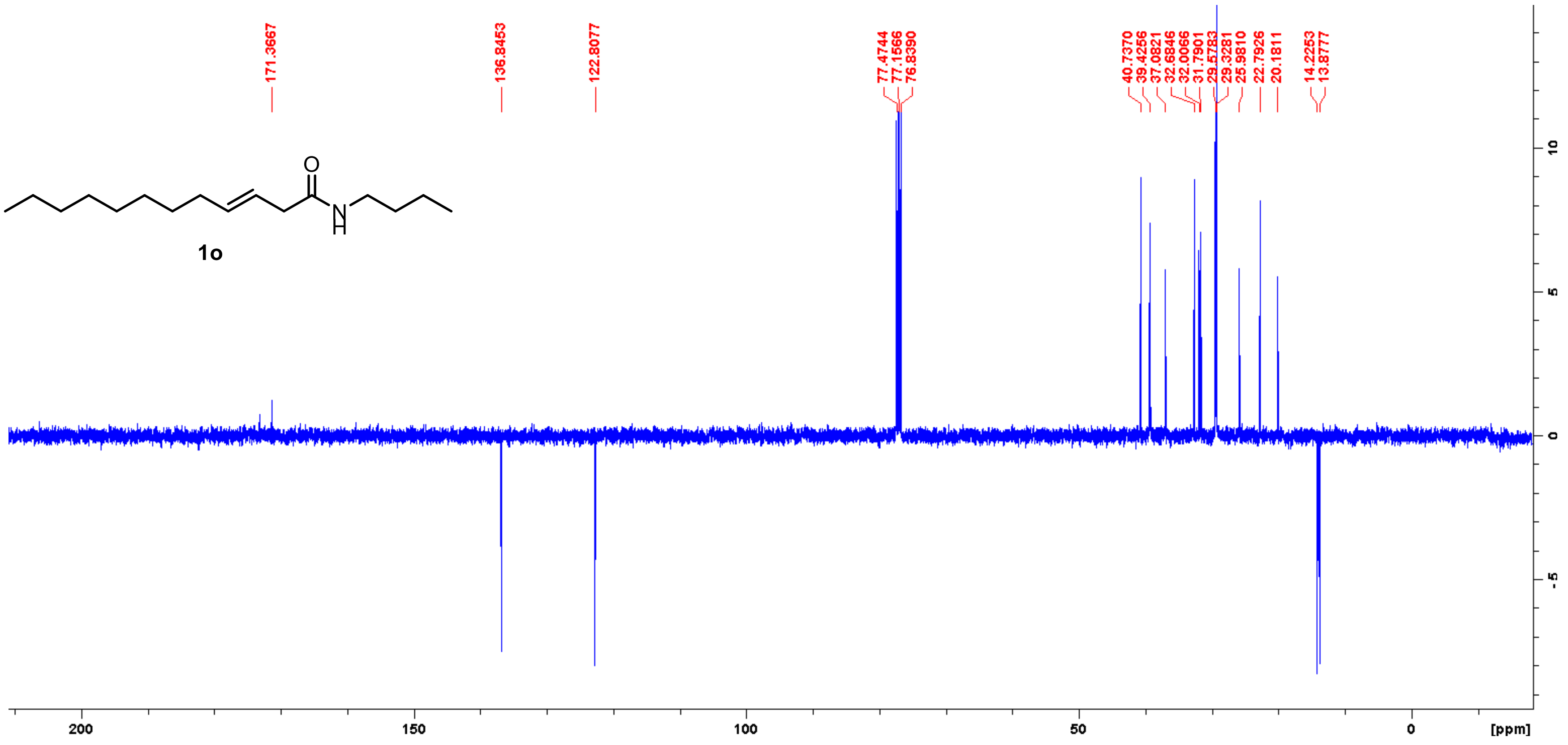

(*E*)-1-(Azepan-1-yl)-pent-3-en-1-one (1p)

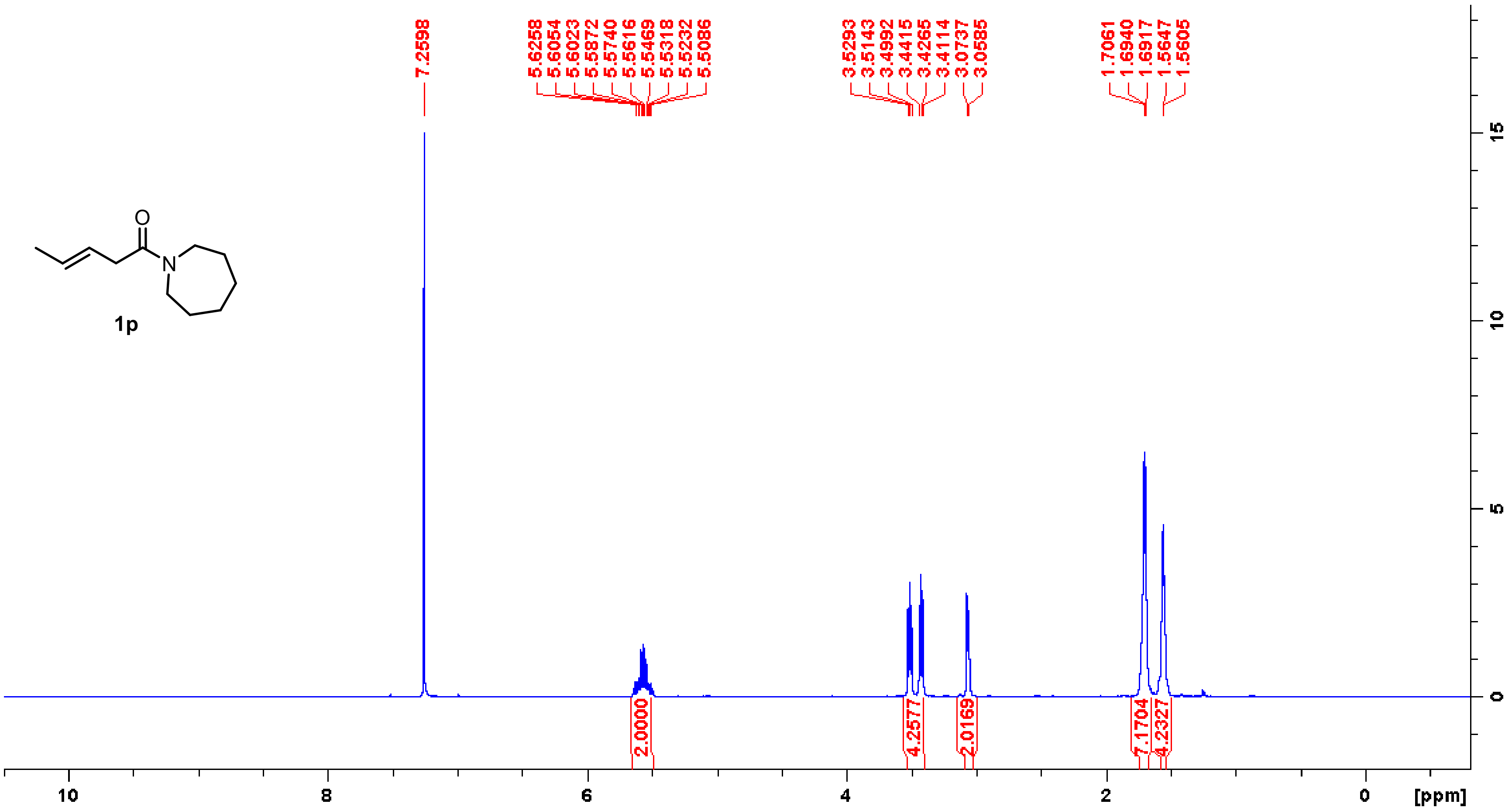

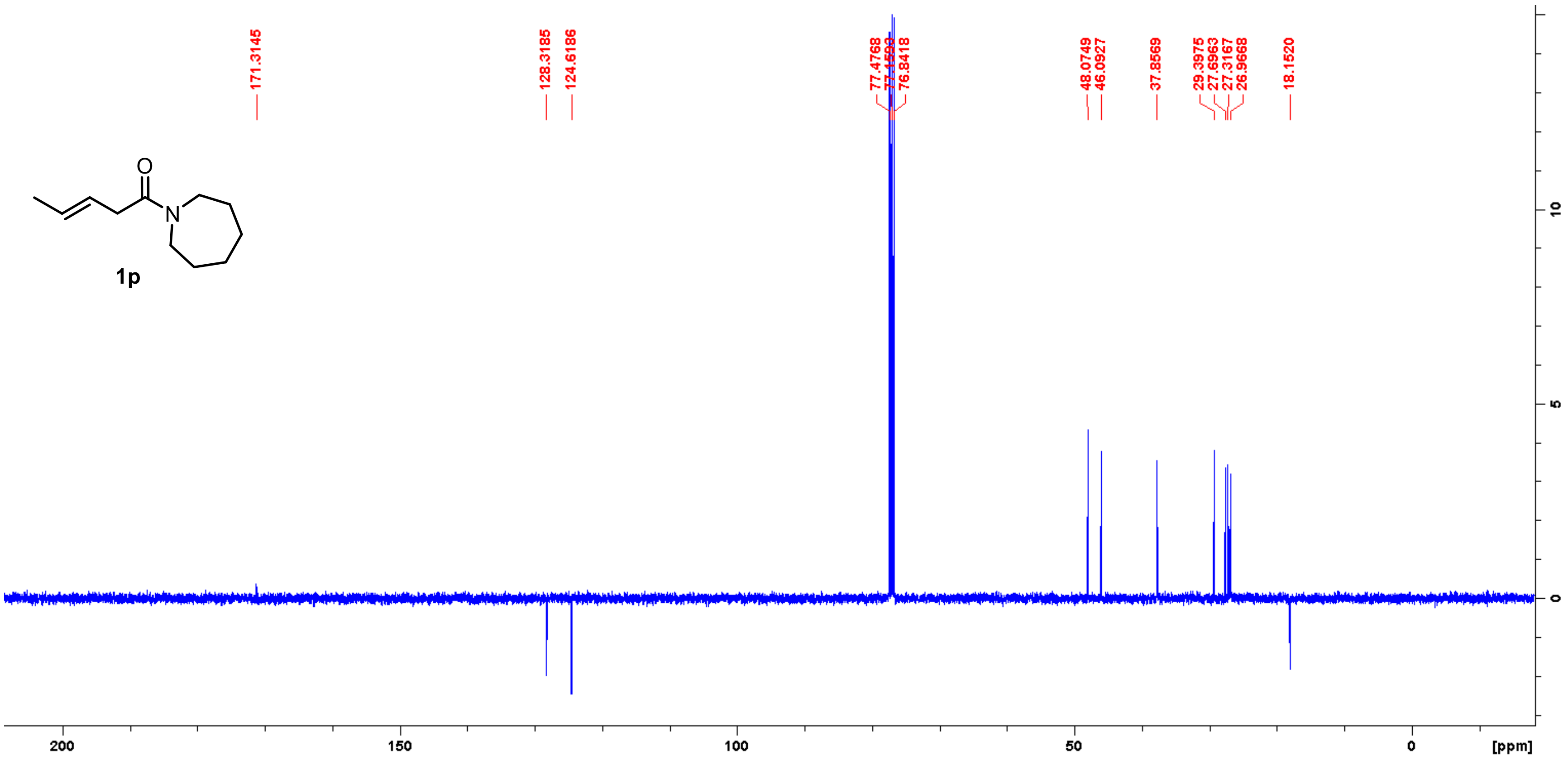

(*E*)-1-(Azetidin-1-yl)-pent-3-en-1-one (1q)

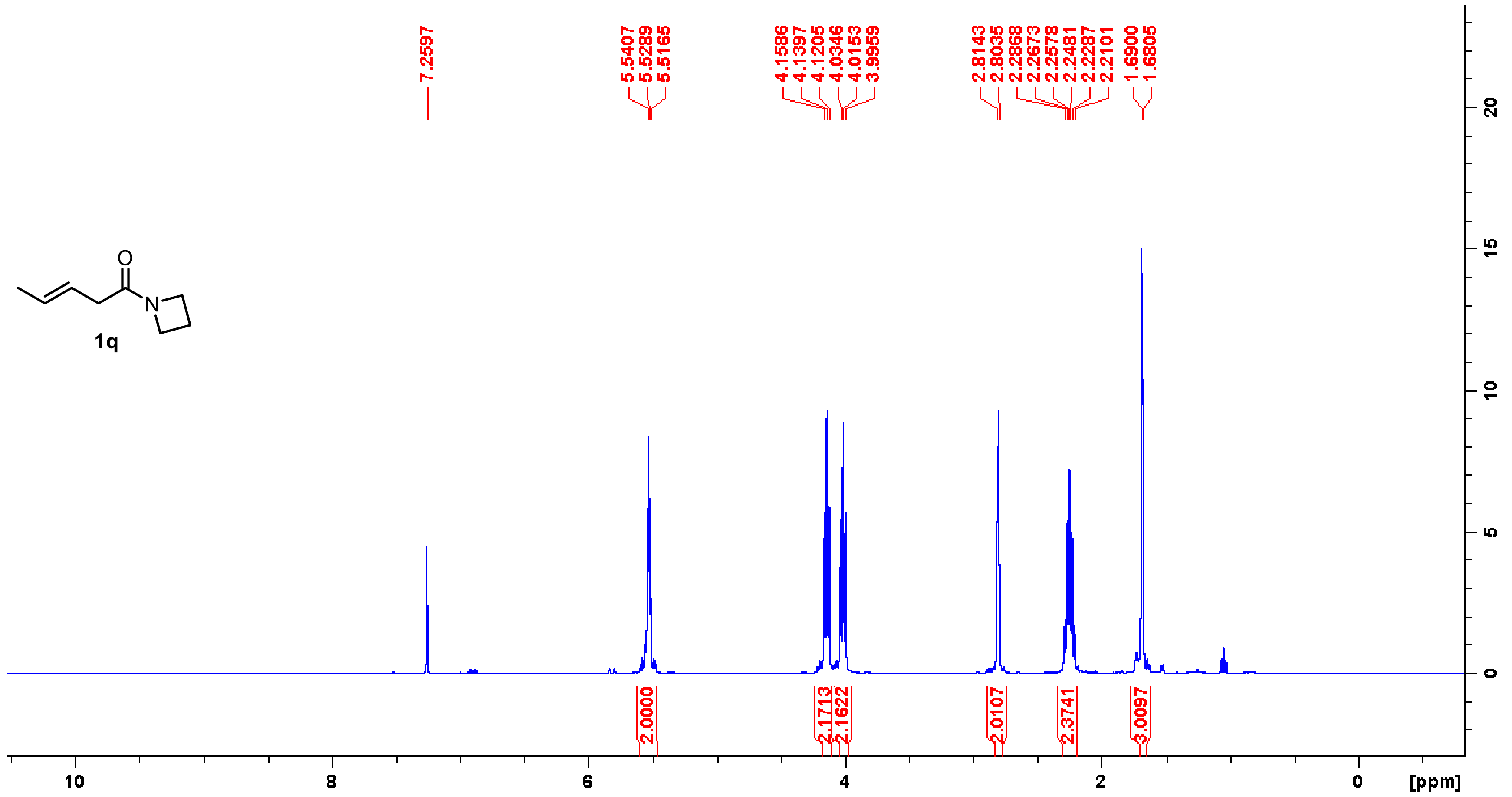

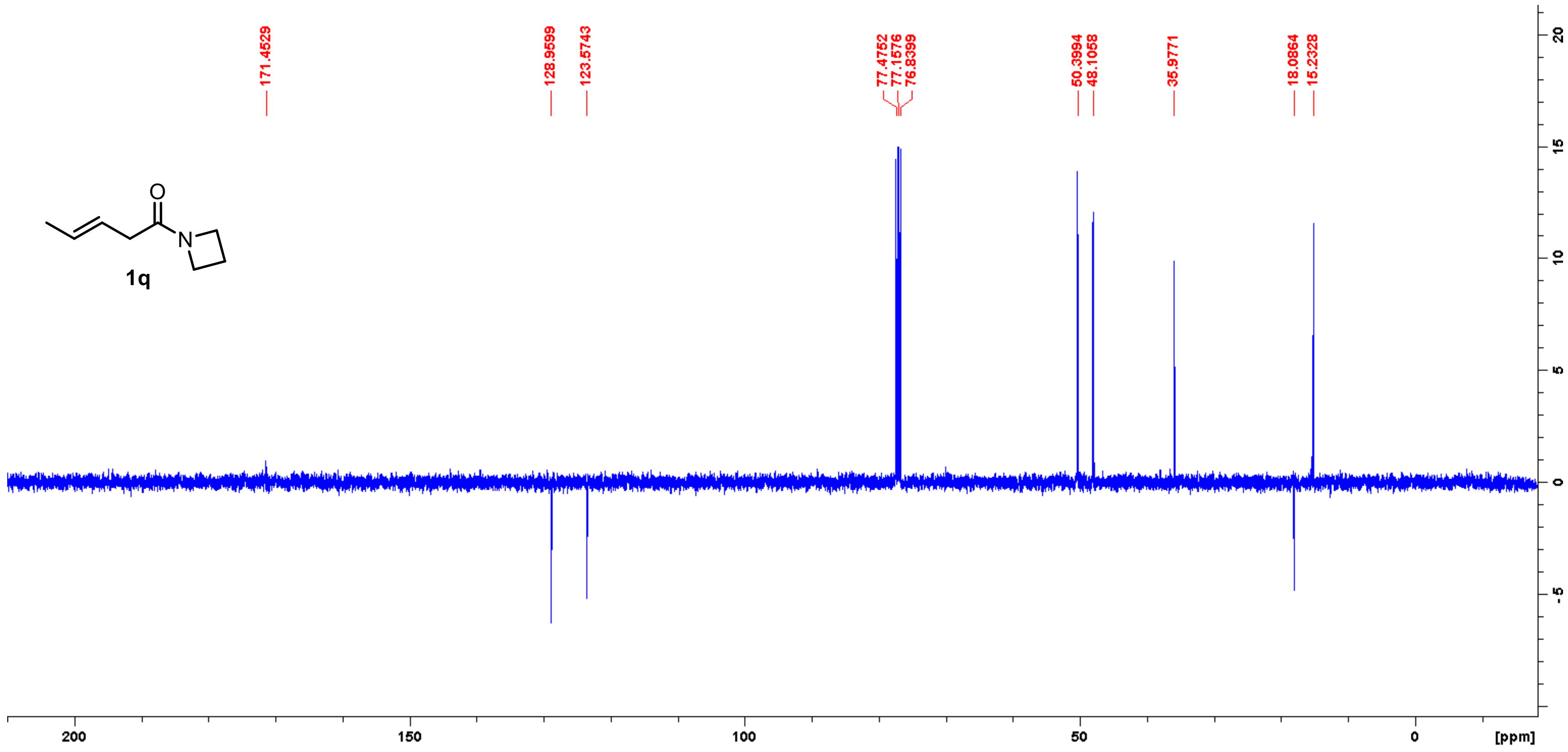

(*E*)-*N*,*N*-Dimethylpent-3-en-amide (1r)

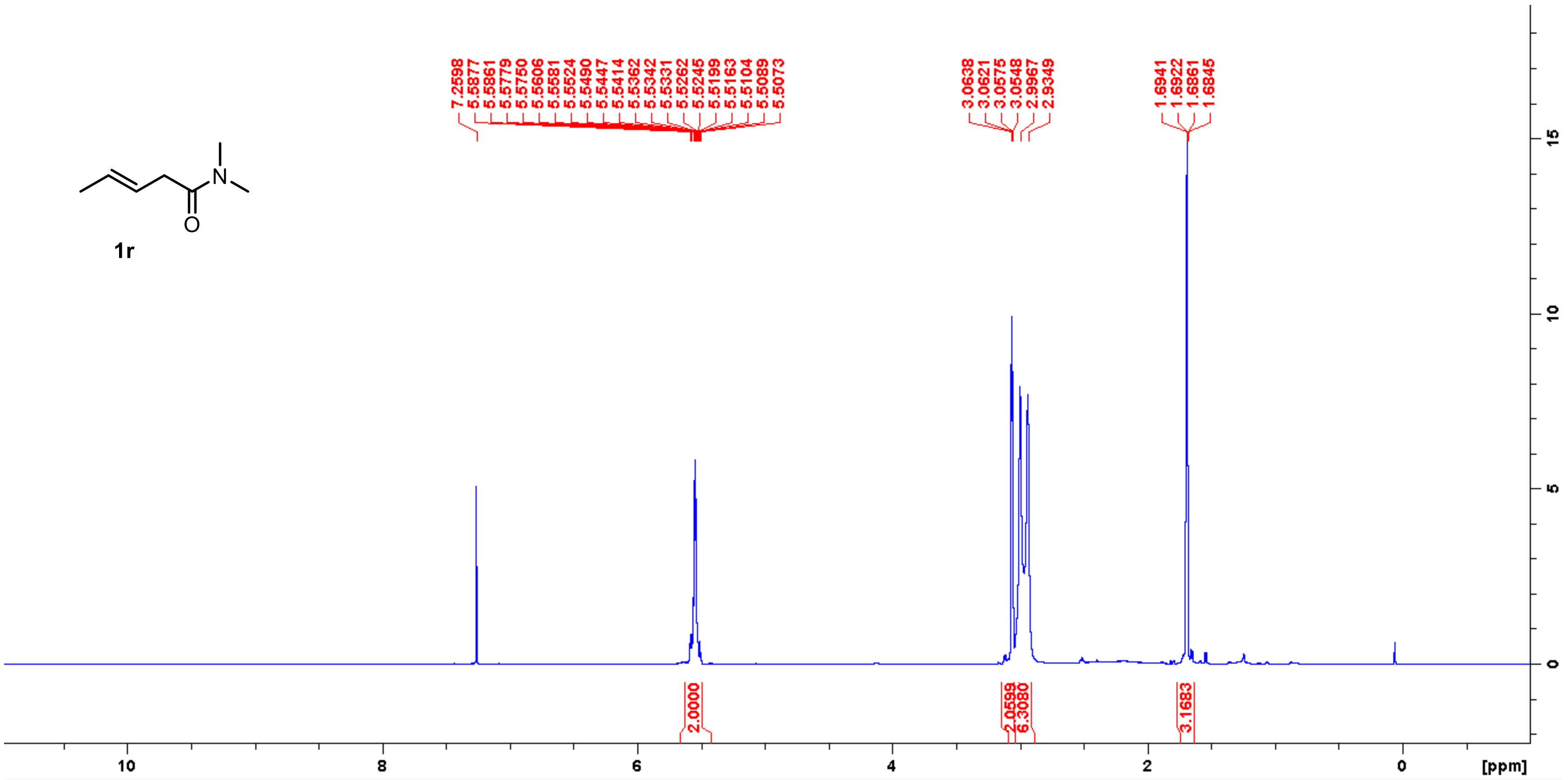

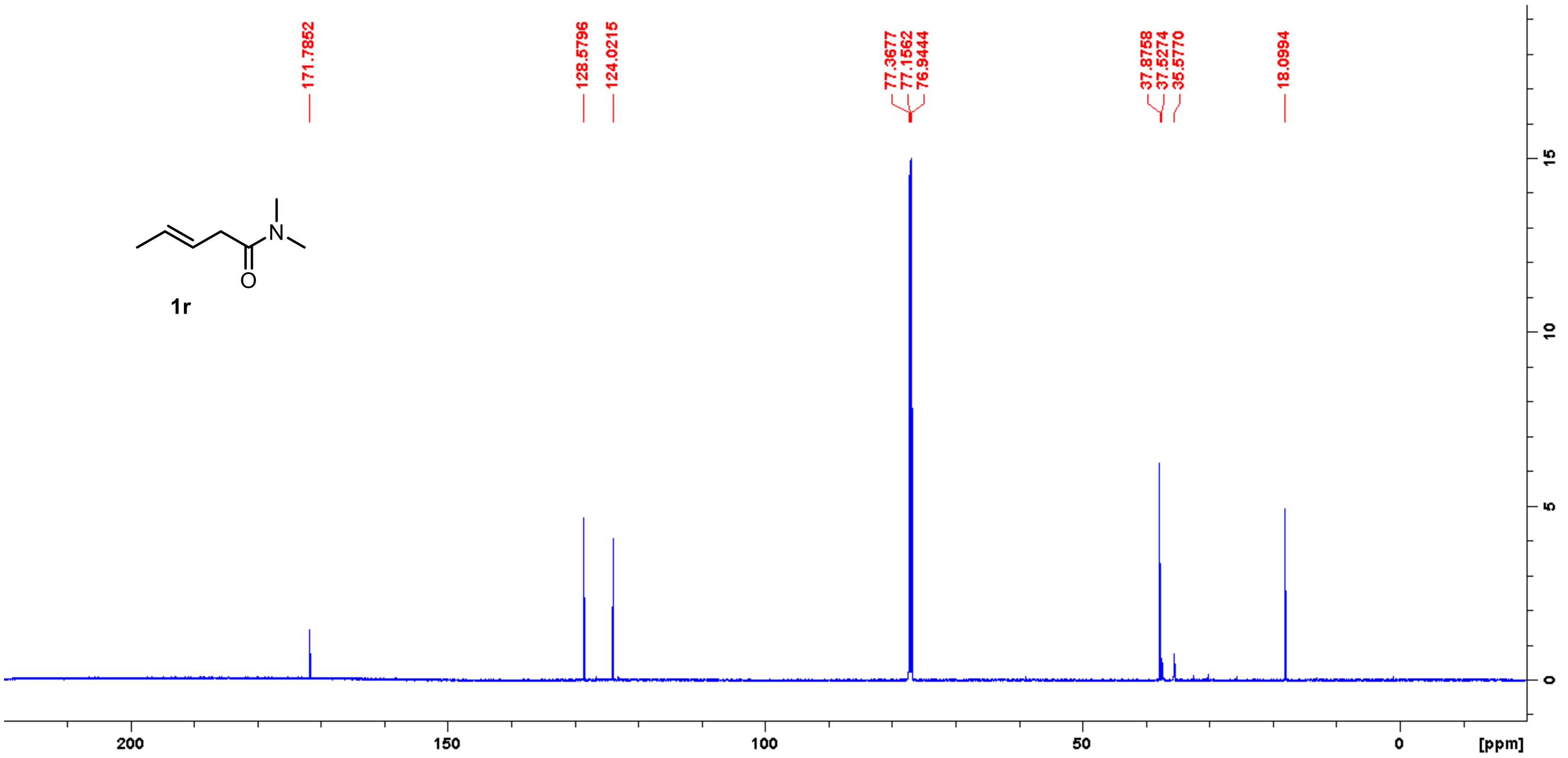

(*E*)-1-Morpholinopent-3-en-1-one (1s)

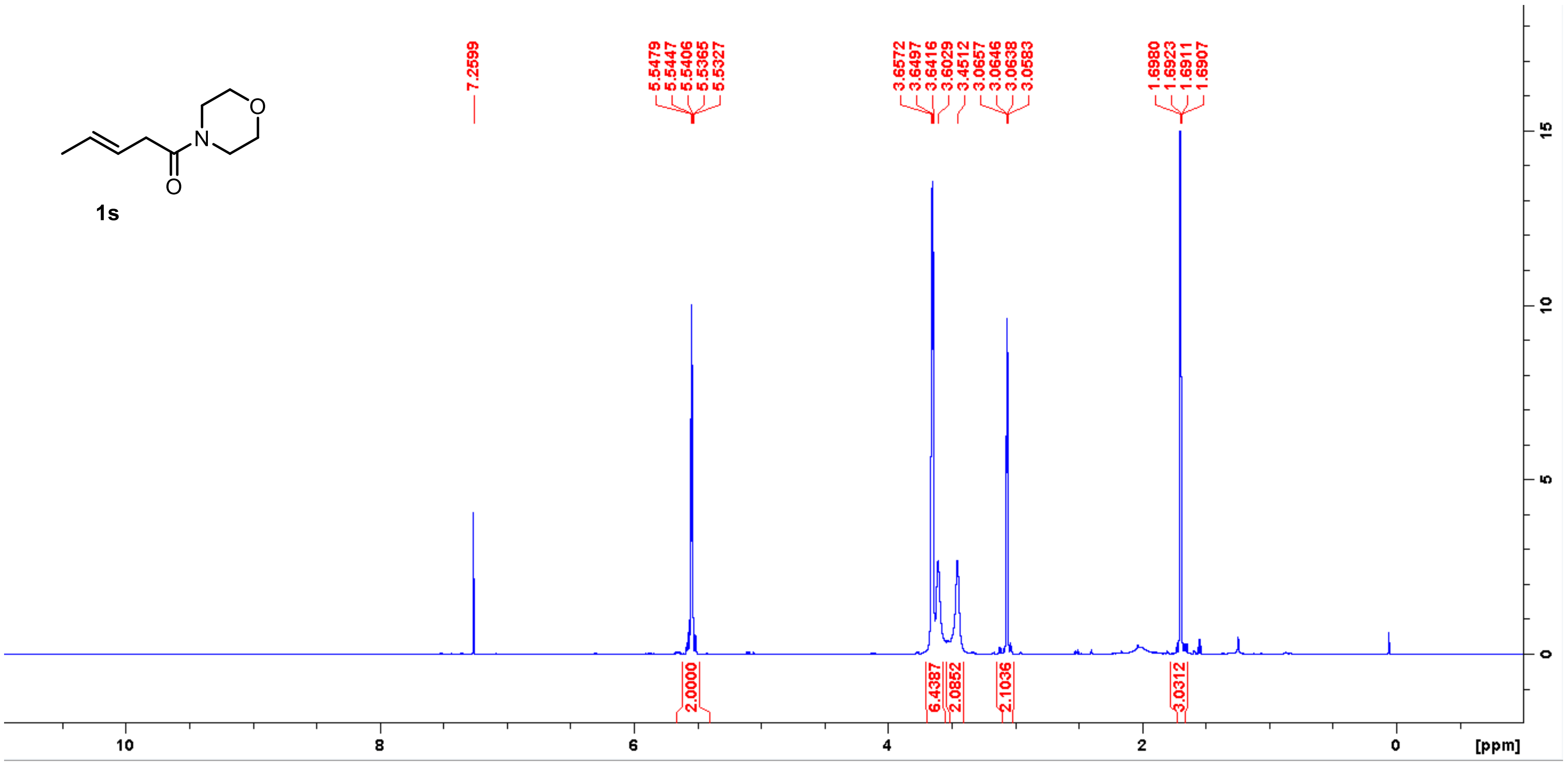

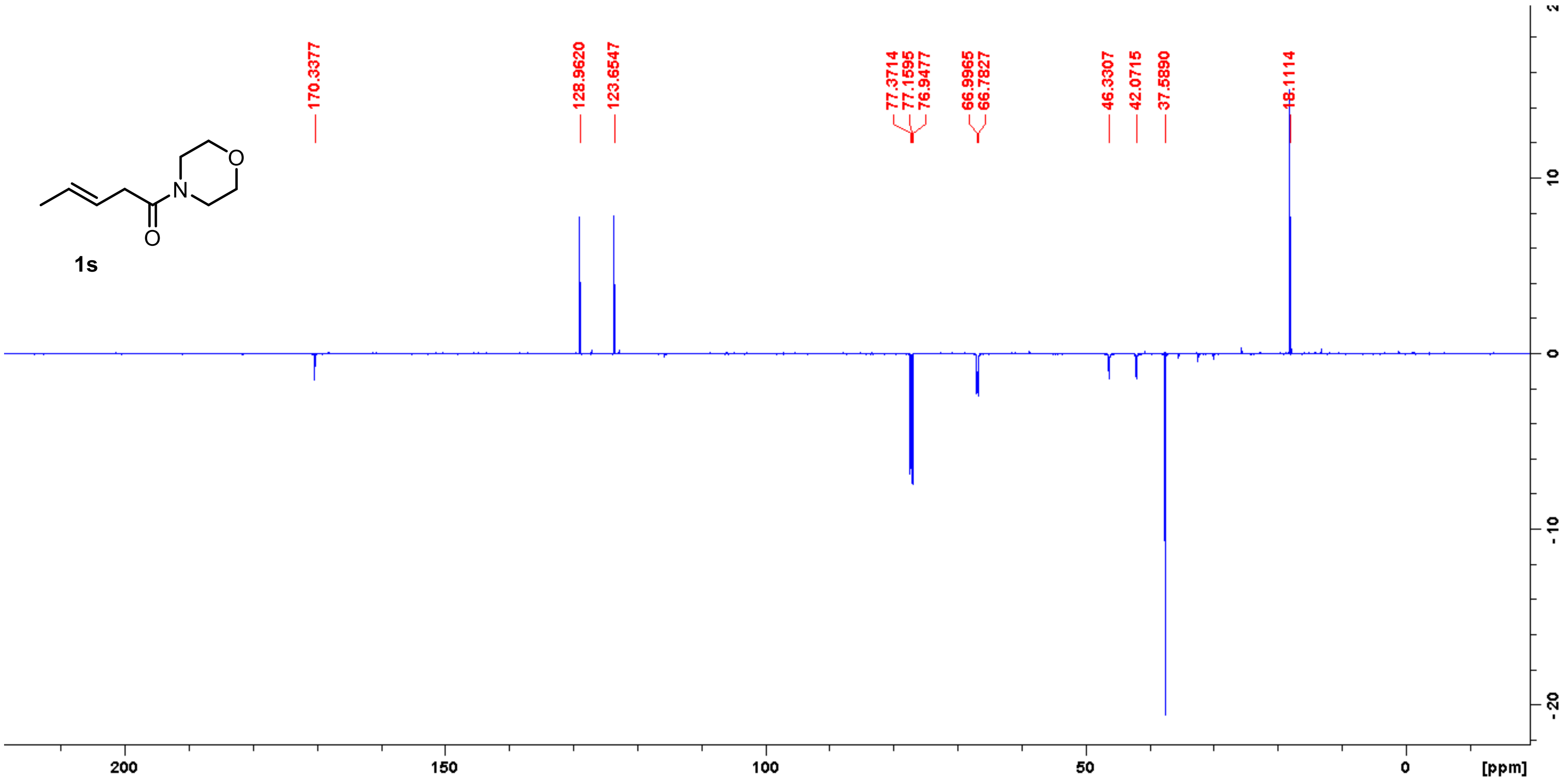

(*E*)-*N*,*N*-Dibenzylpent-3-en-amide (1t)

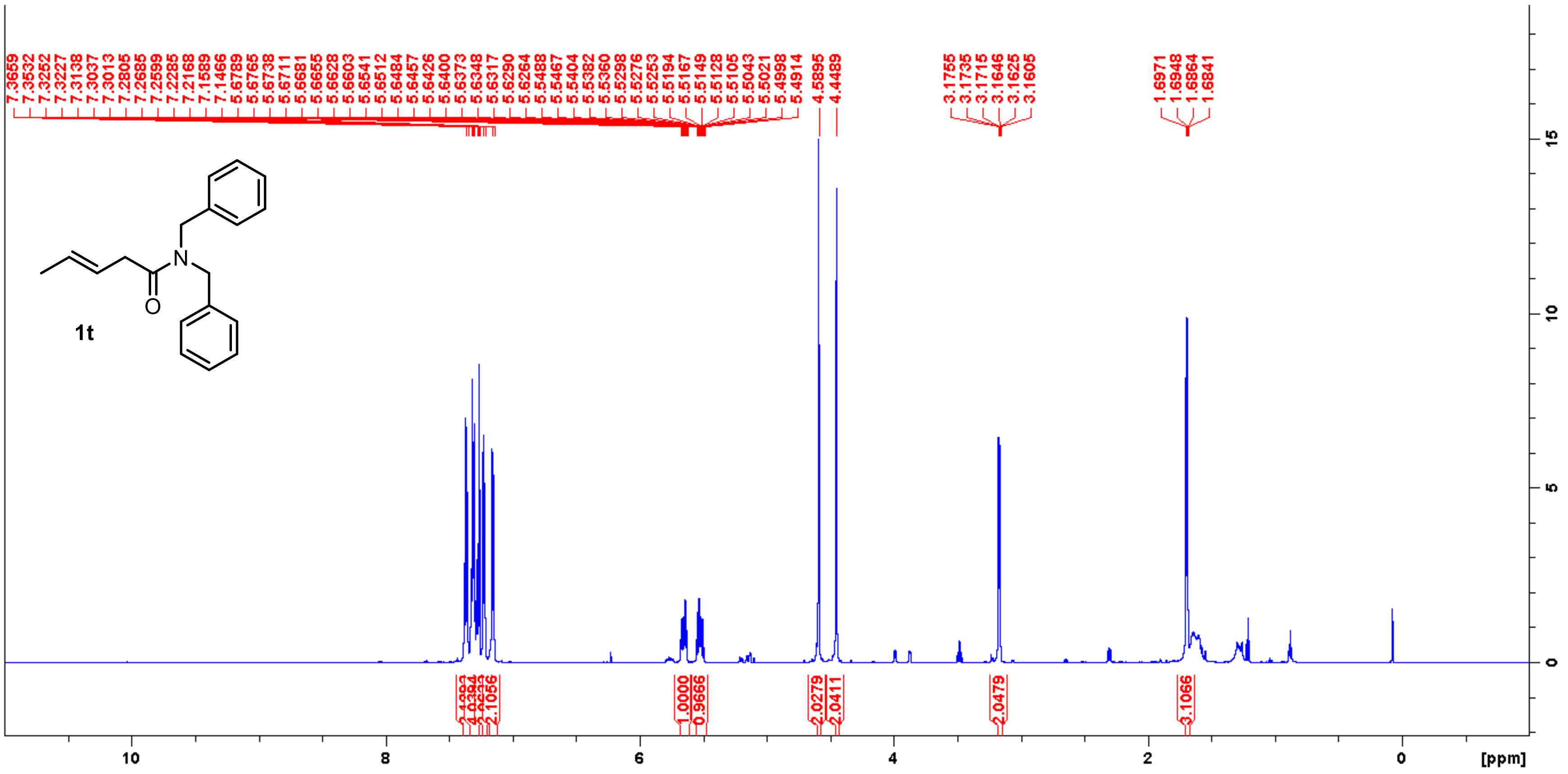

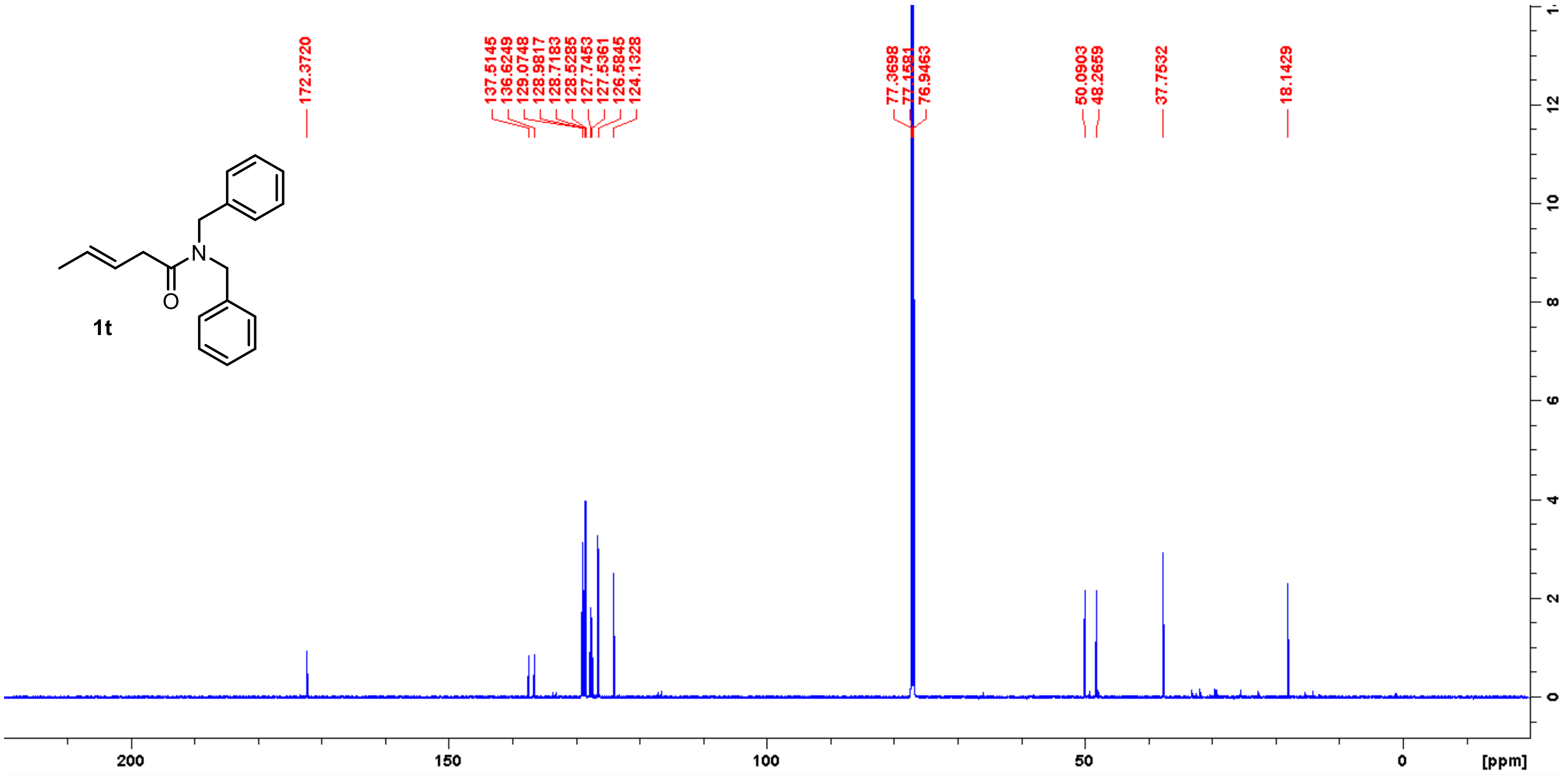

(*E*)-*N*-Butyl-*N*-(3-methylbut-2-en-1-yl)-pent-3-enamide (1u; rotamers present)

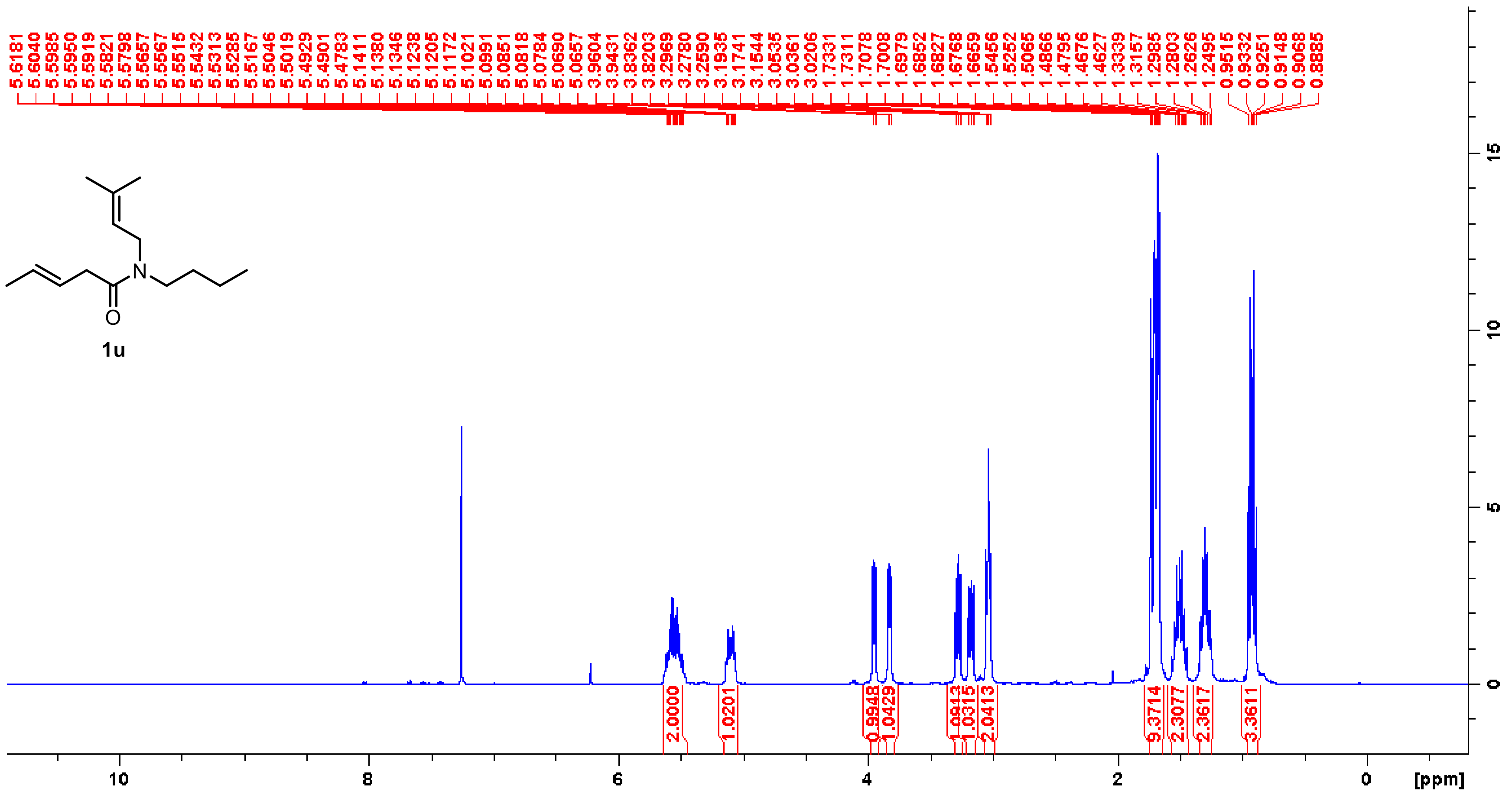

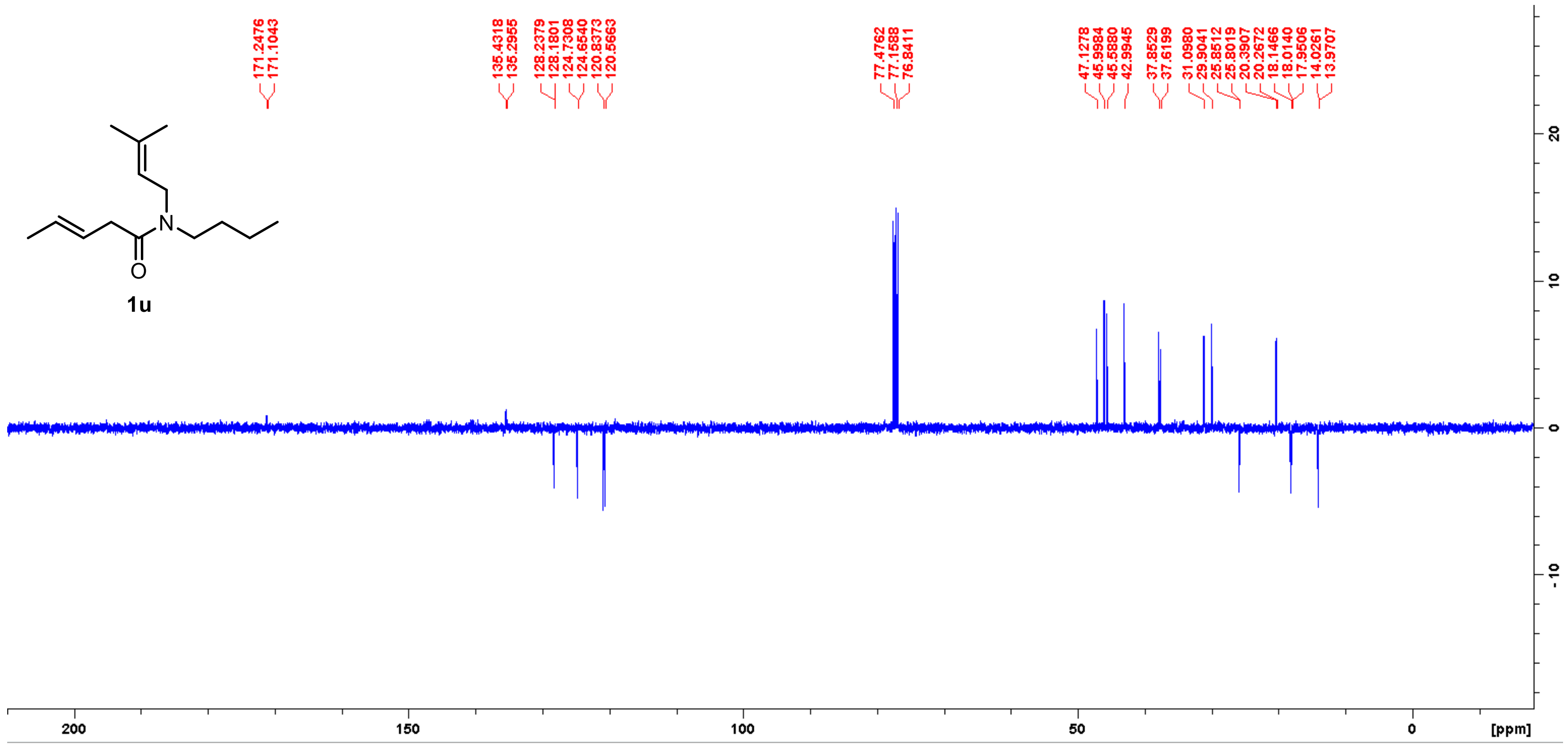

(*E*)-*N*-Allyl-*N*-methylpent-3-en-amide (1v, rotamers present)

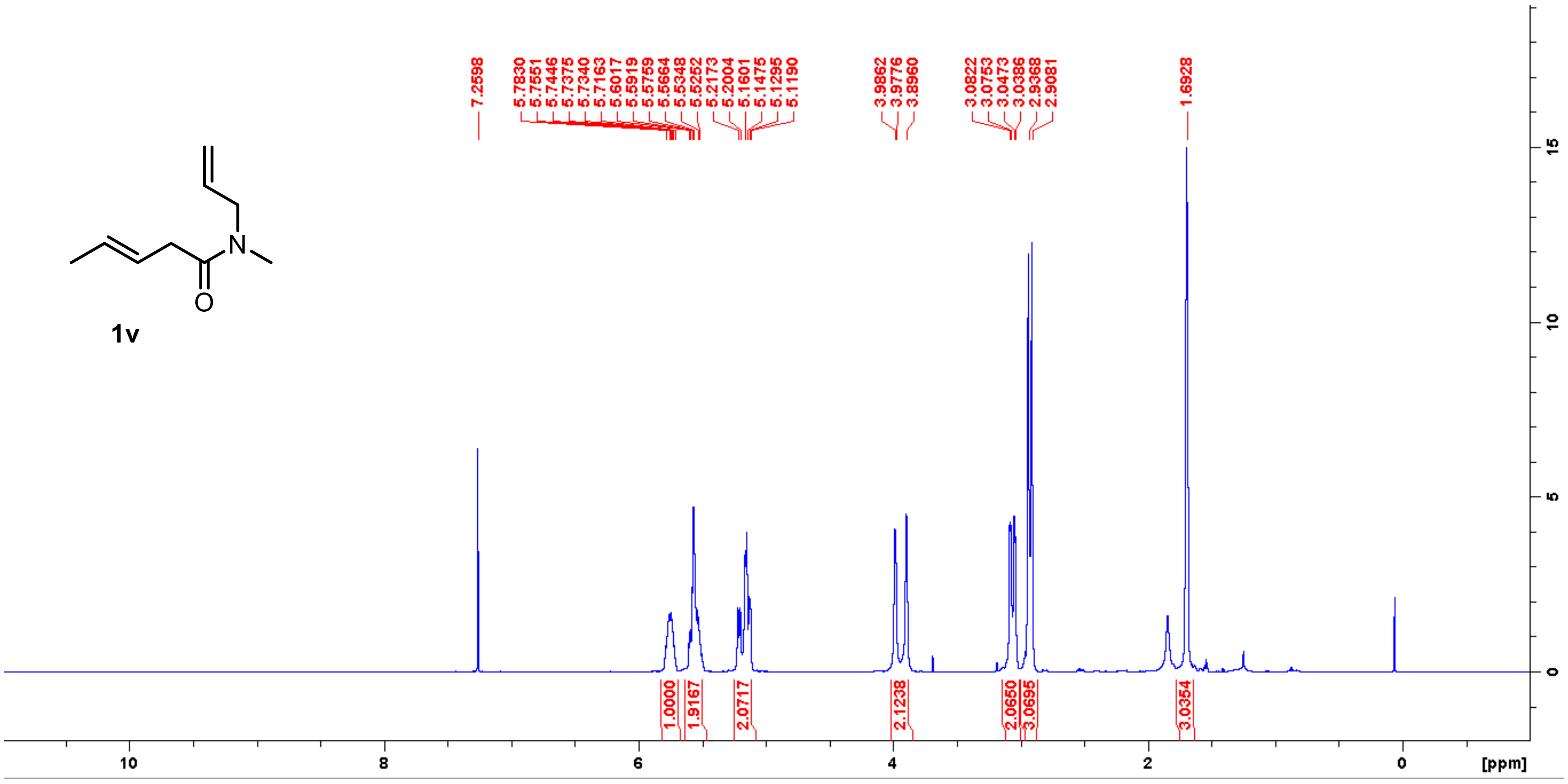

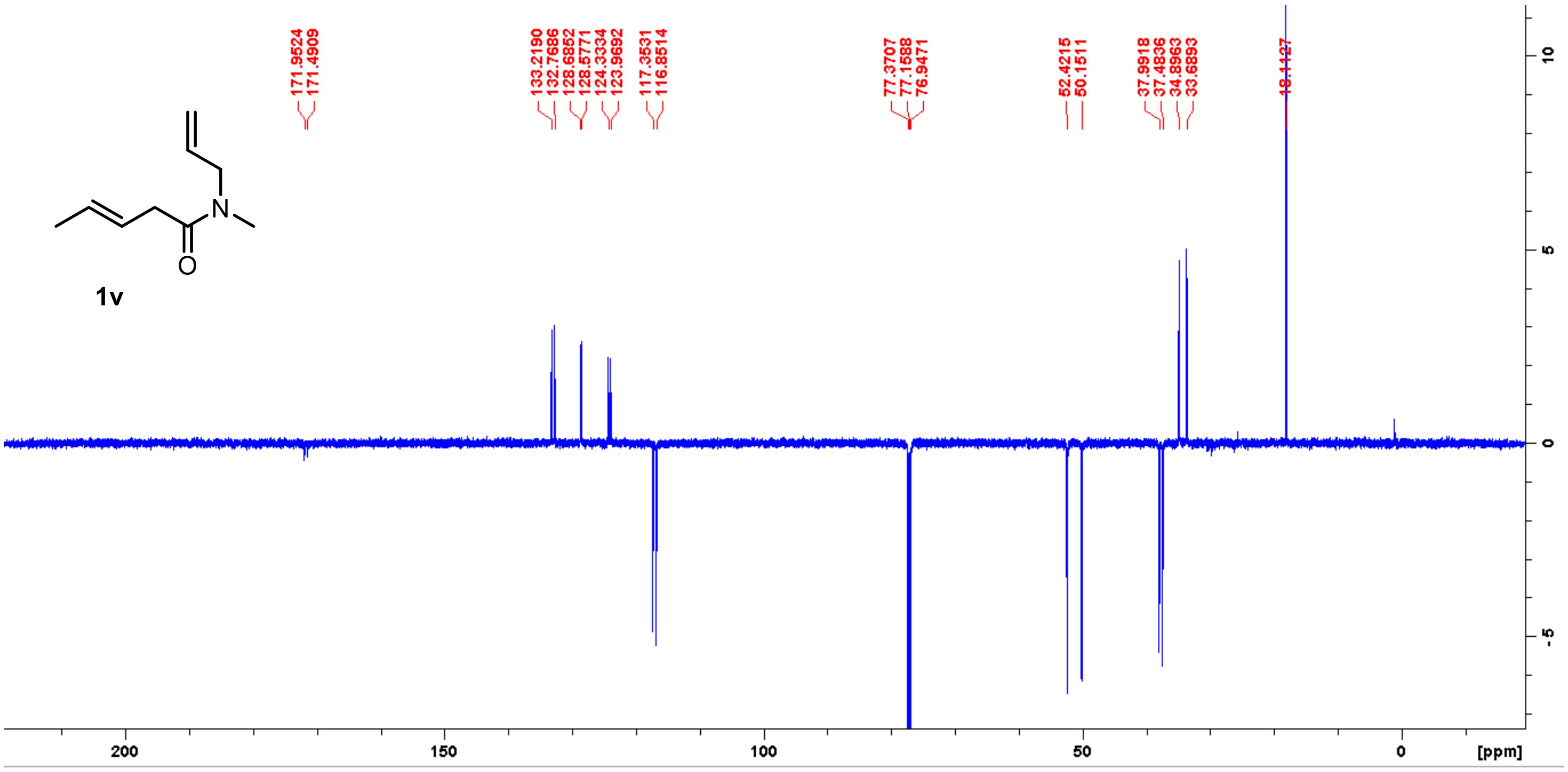

(*E*)-*N*-Cyclopropyl-*N*-methylpent-3-enamide (1w)

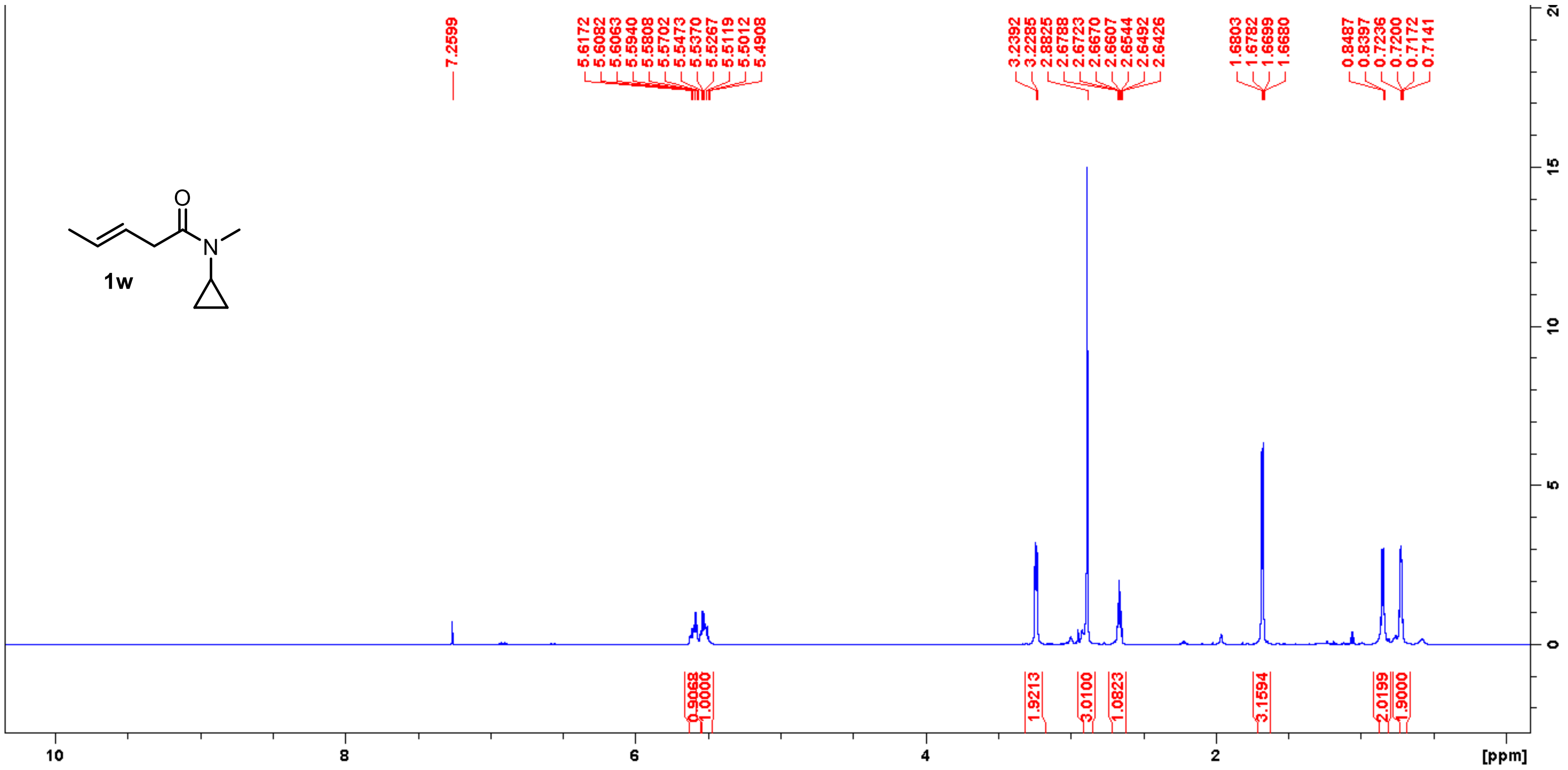

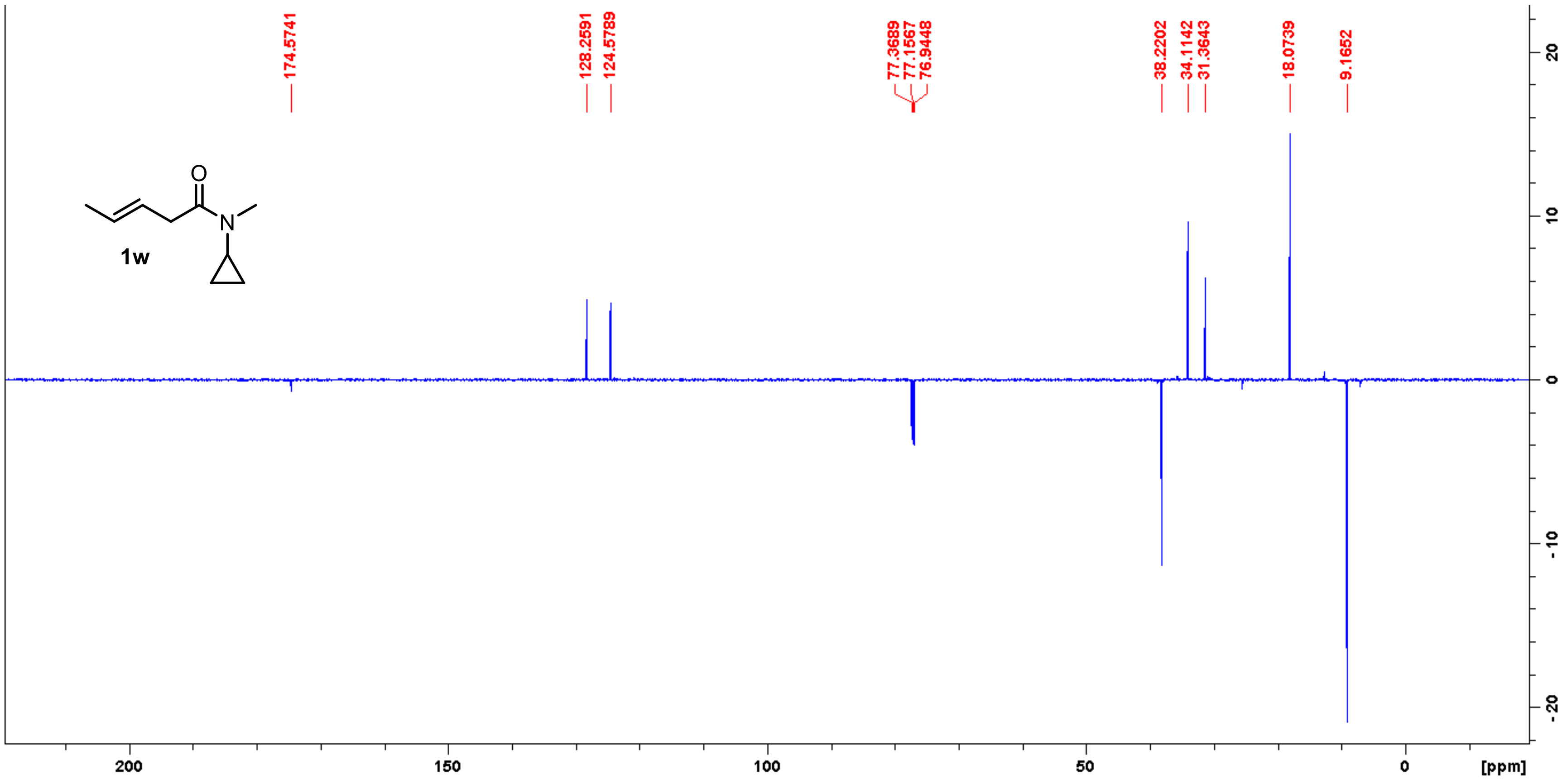

(*E*)-*N*-(Cyclopropylmethyl)-*N*-methylpent-3-enamide (1x; rotamers present)

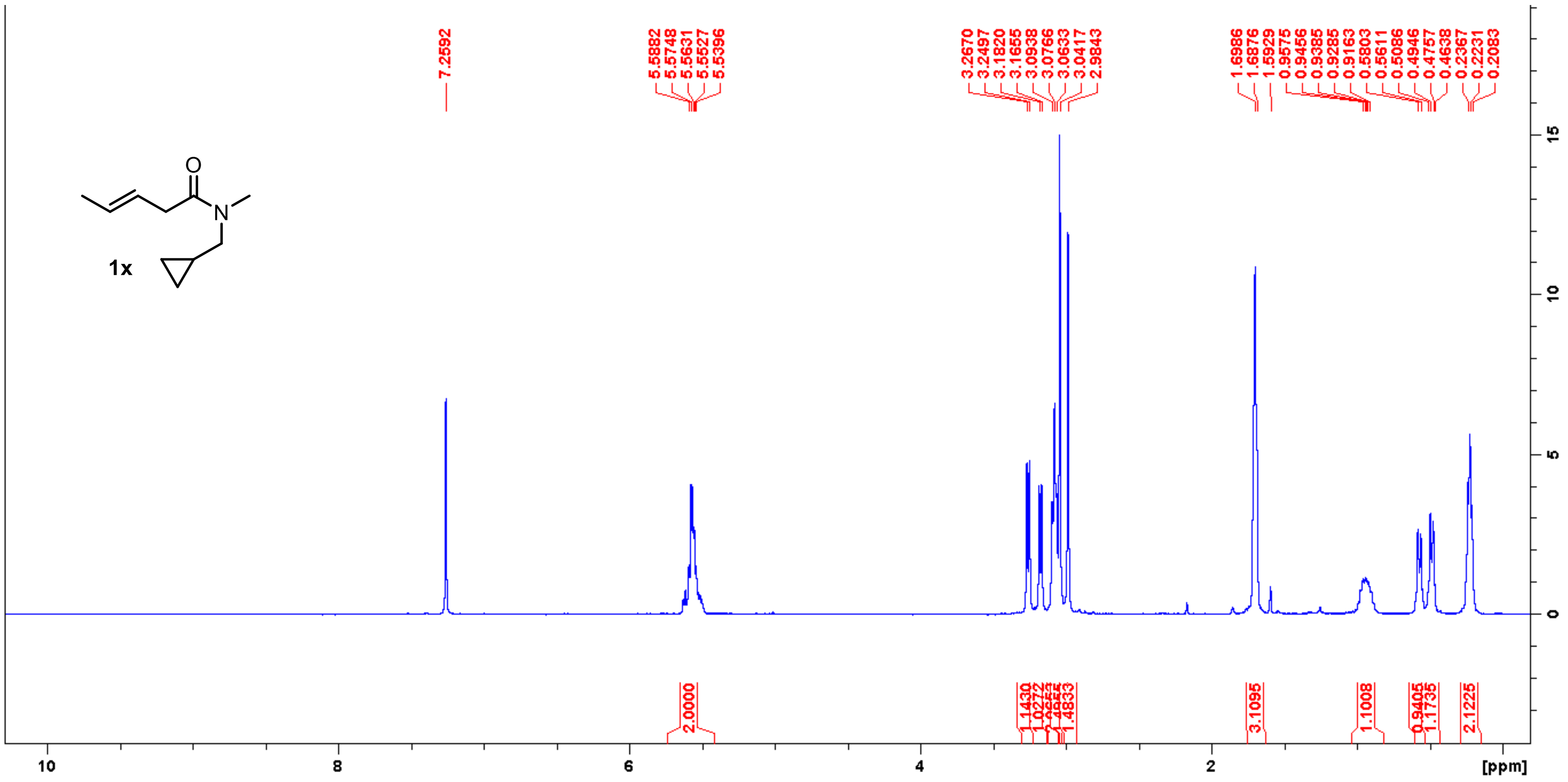

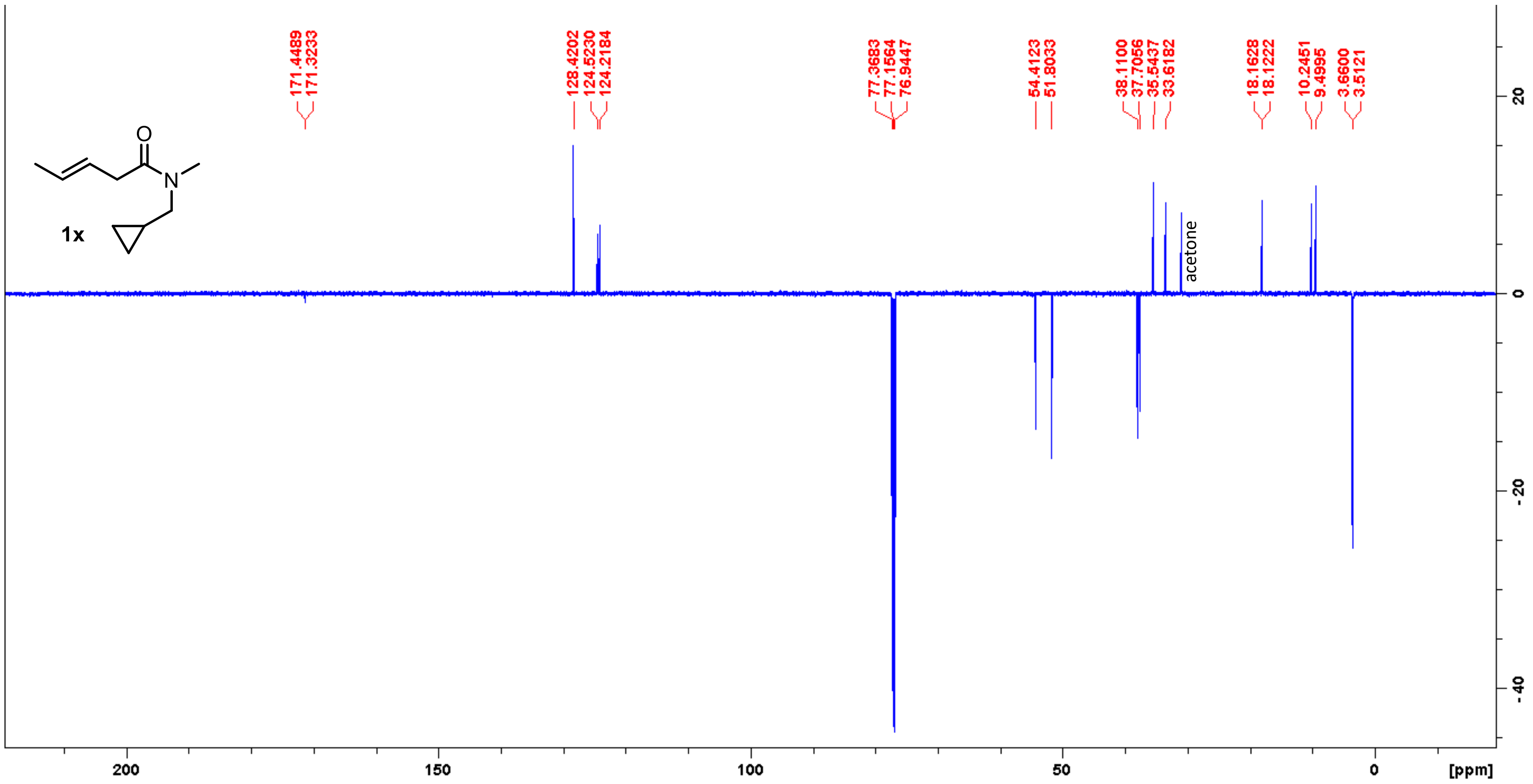

(*E*)-1-(2-Phenylpyrrolidin-1-yl)-dodec-3-en-1-one (1y; rotamers present)

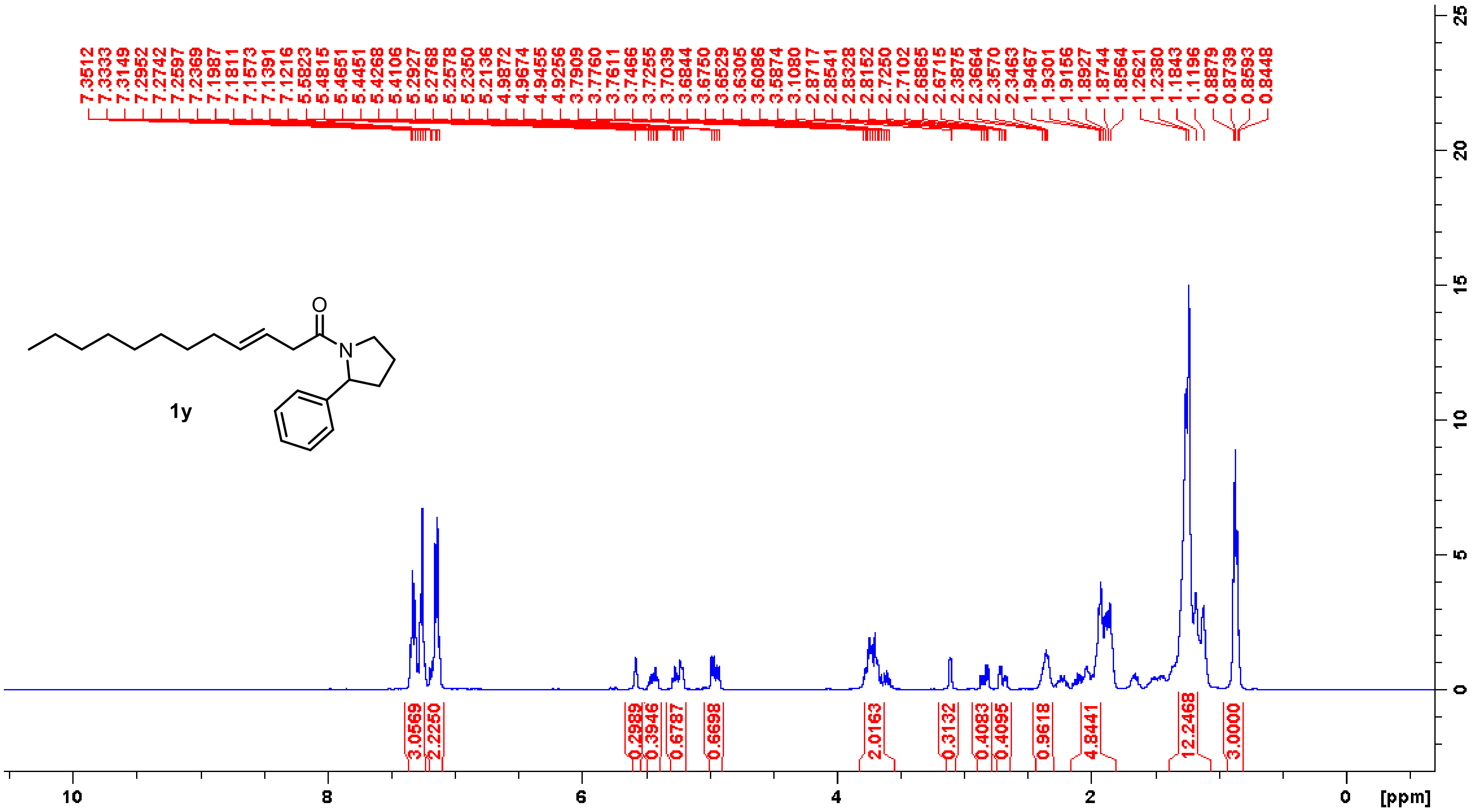

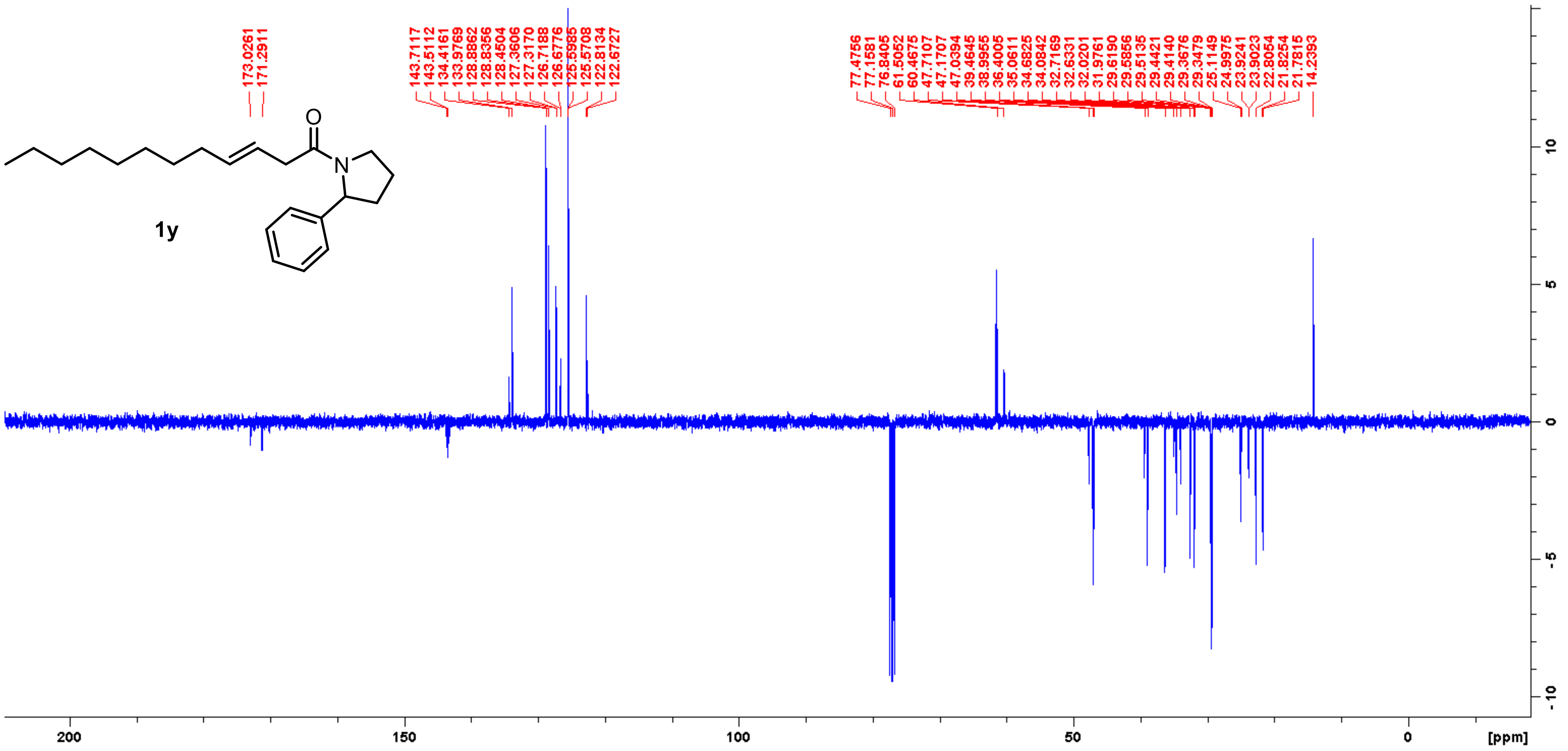

(*R*,*E*)-*N*-Butyl-5,9-dimethyl-N-(3-methylbut-2-en-1-yl)-deca-3,8-dienamide (1z; rotamers present)

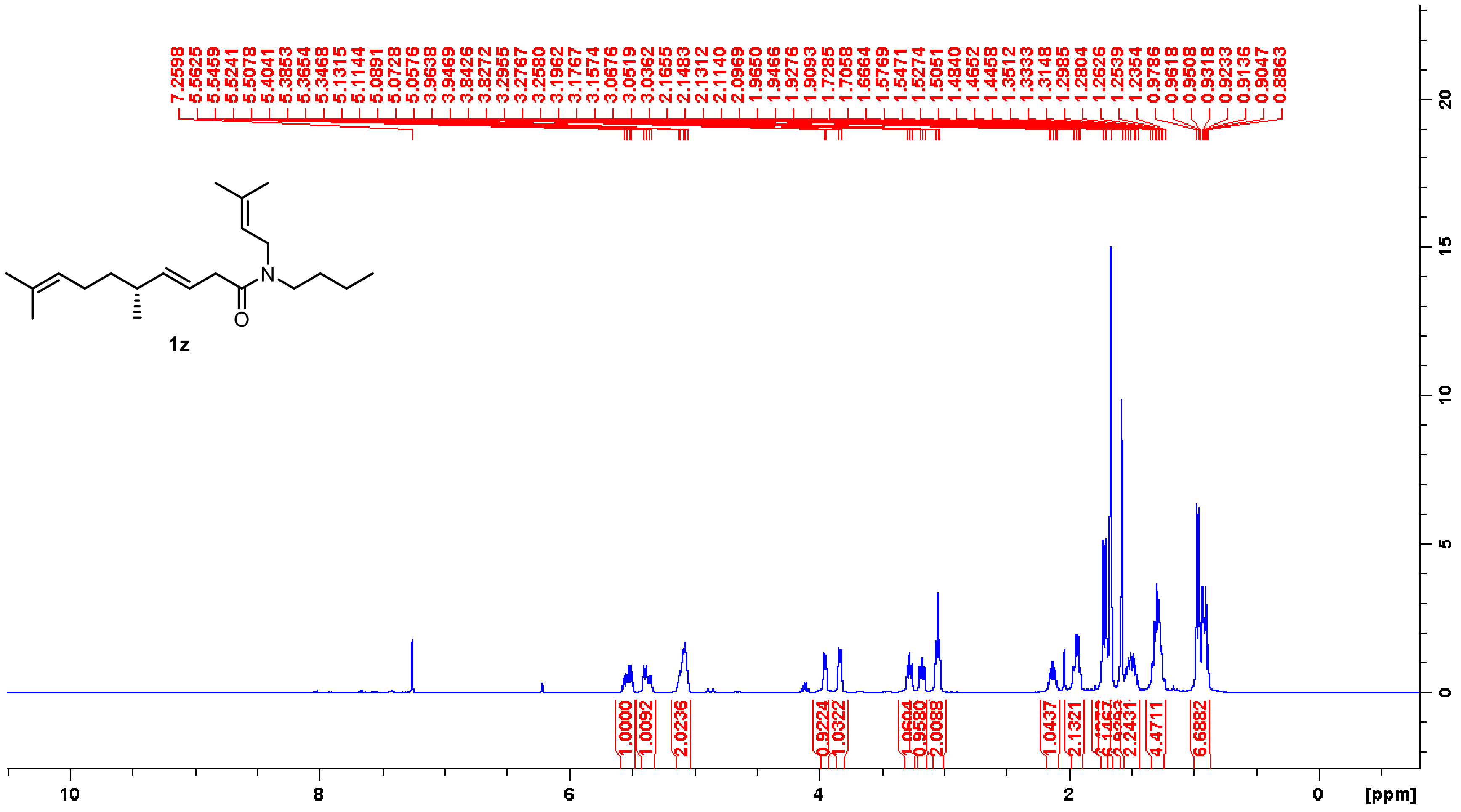

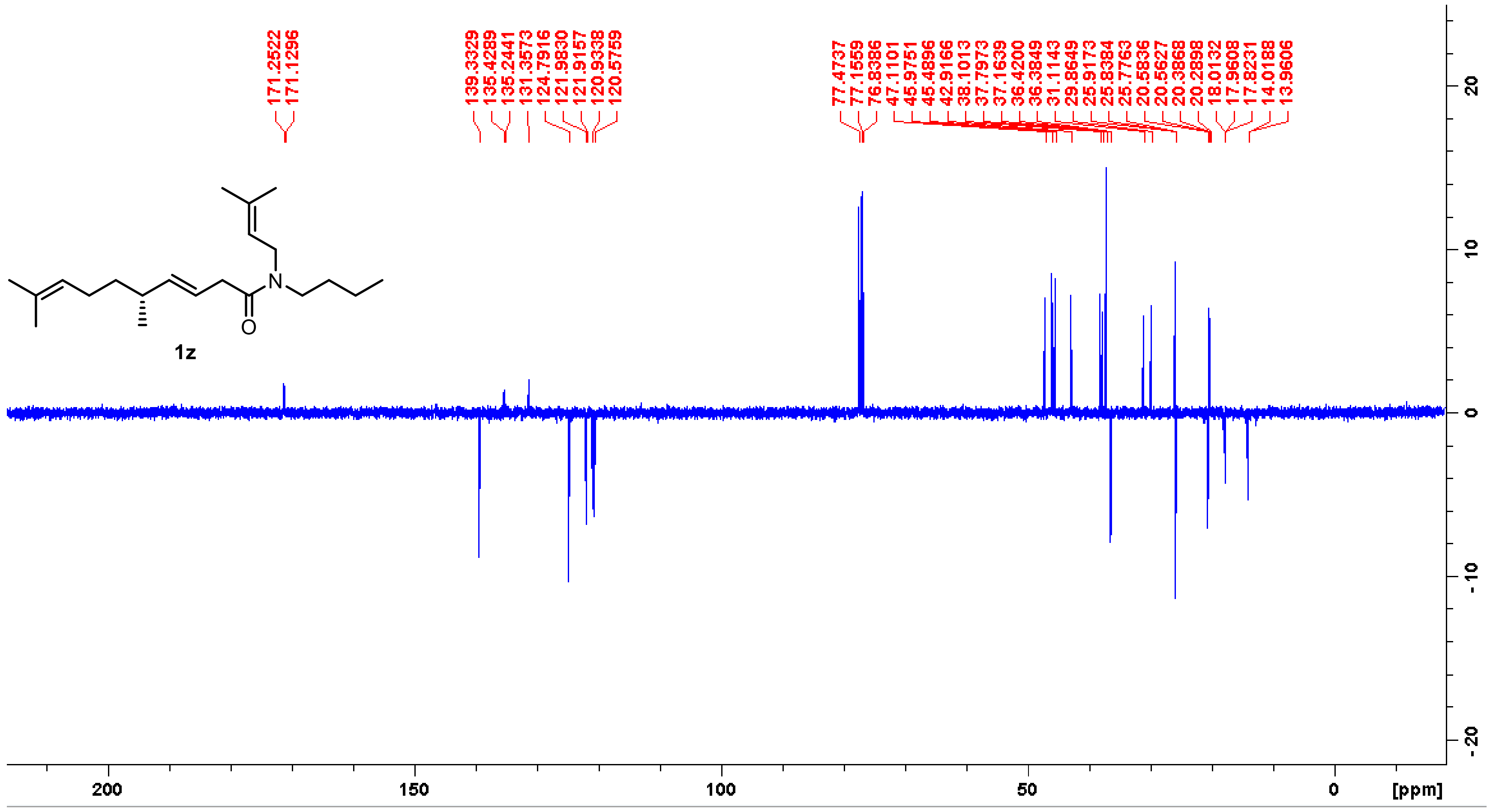

(*E*)-*N*-(3-(10,11-dihydro-5*H*-dibenzo[b,f]azepin-5-yl)propyl)-*N*-methylpent-3-enamide (1ab; rotamers present)

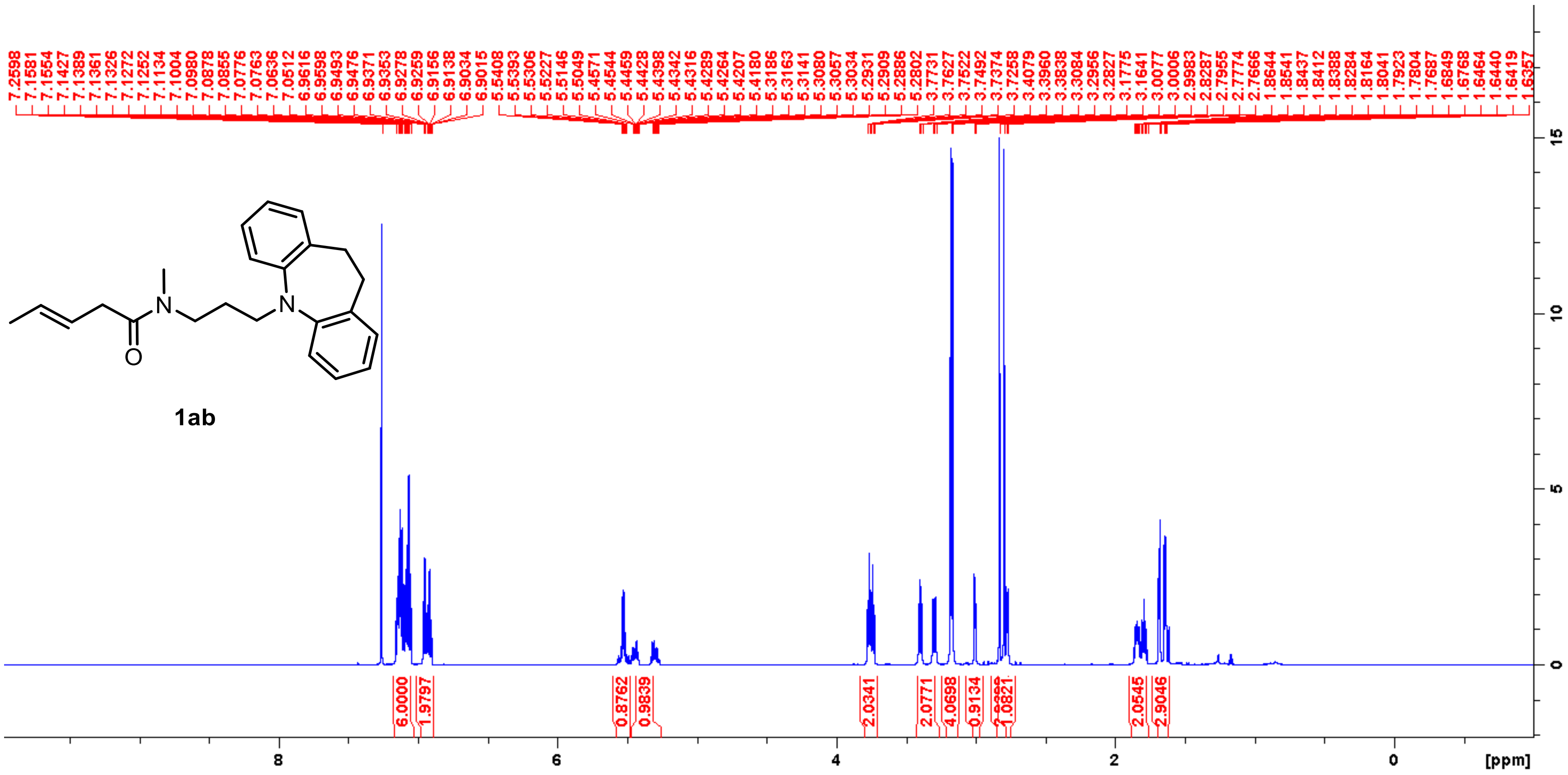

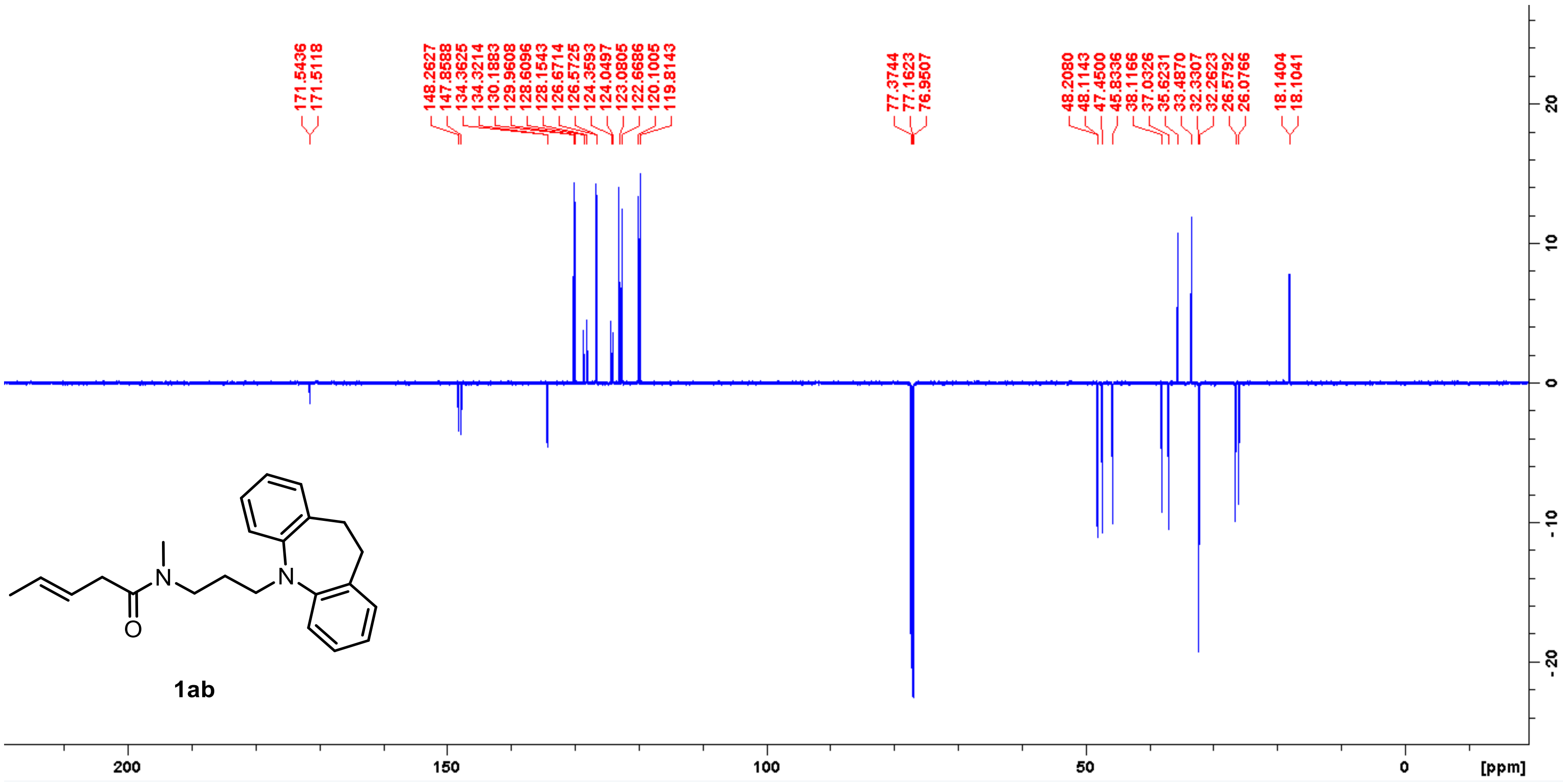

1-(Pyrrolidin-1-yl)-but-3-yn-1-one (1ac)

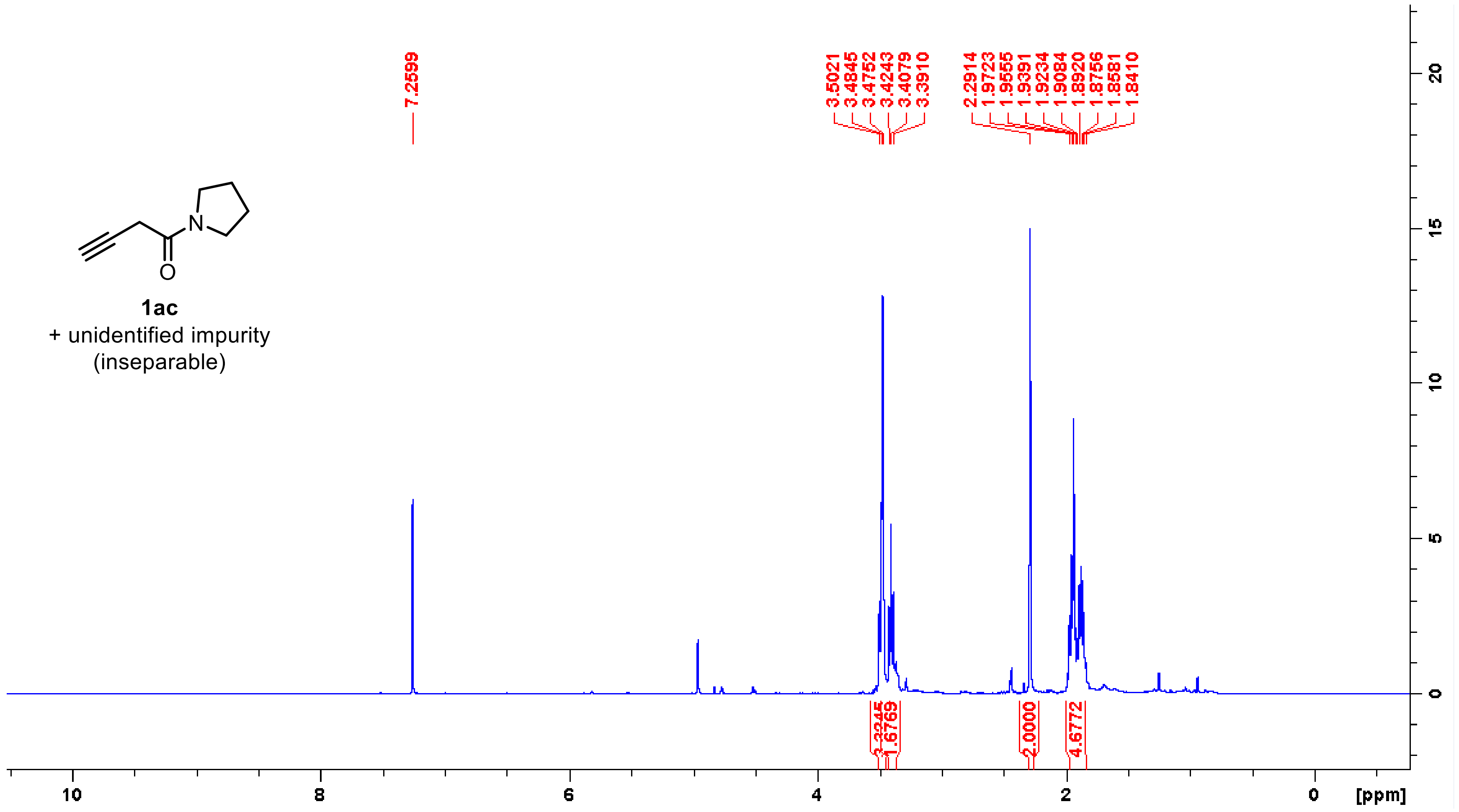

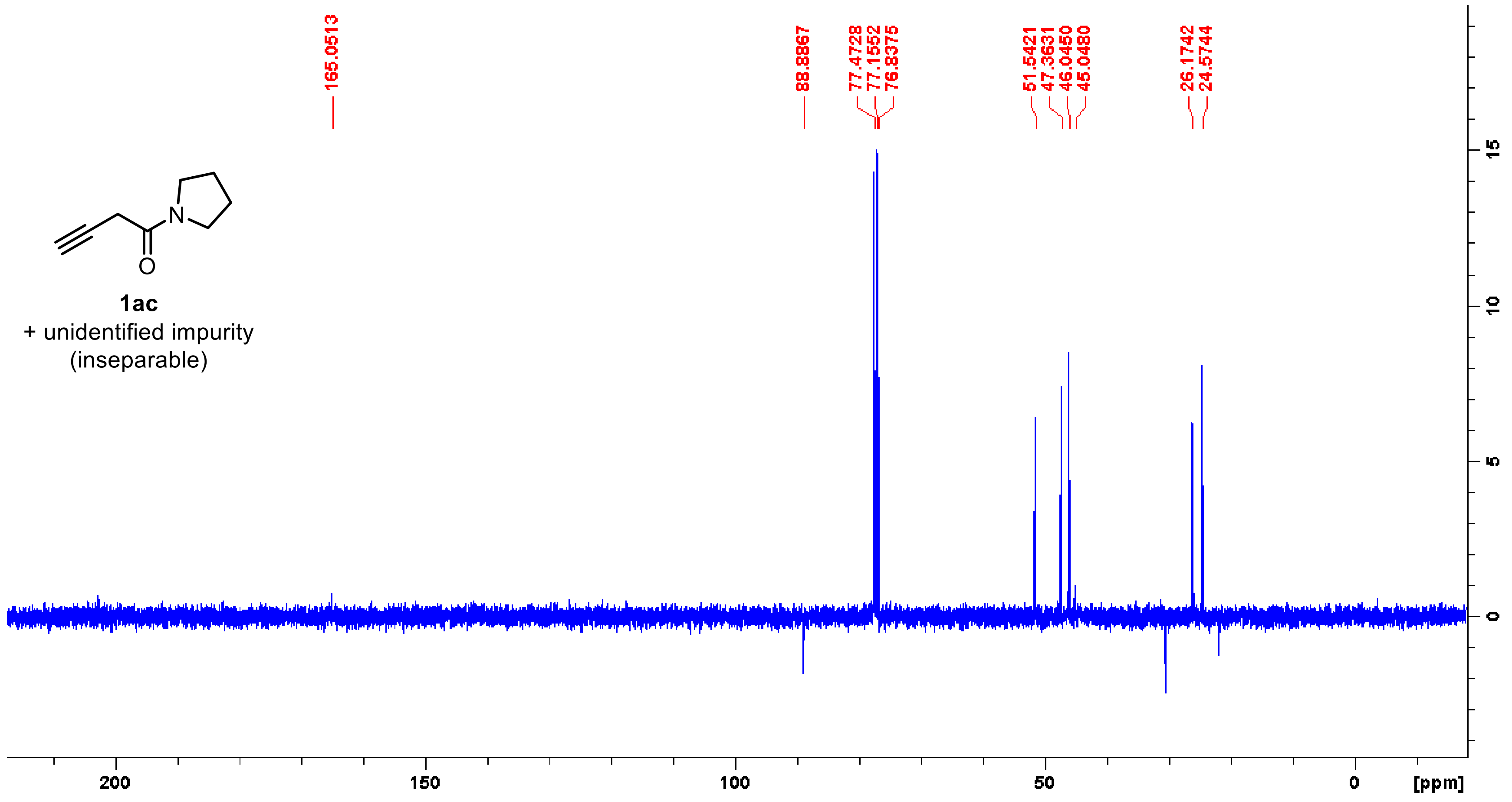

4-Methyl-1-(pyrrolidin-1-yl)-pent-3-en-1-one (1ad)

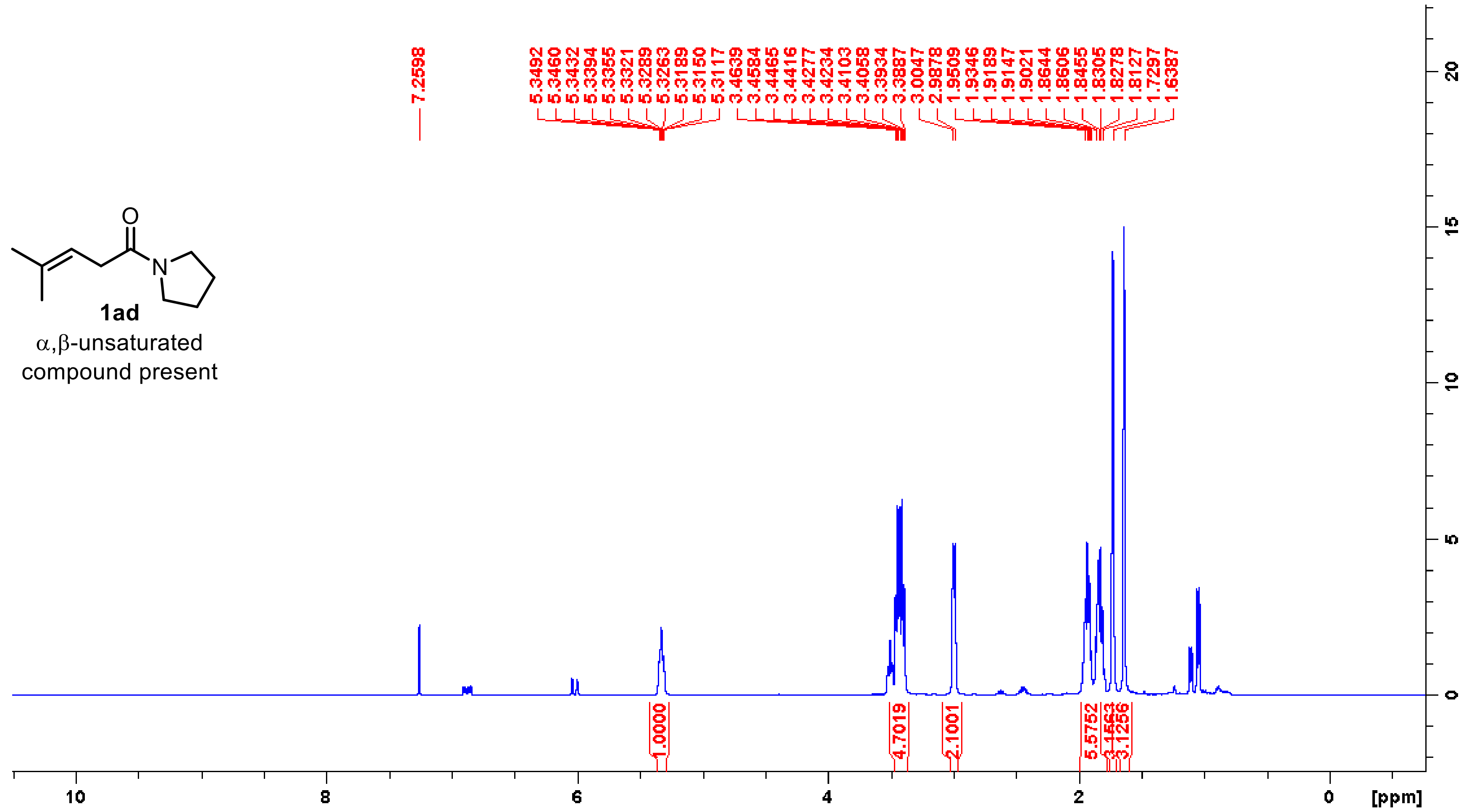

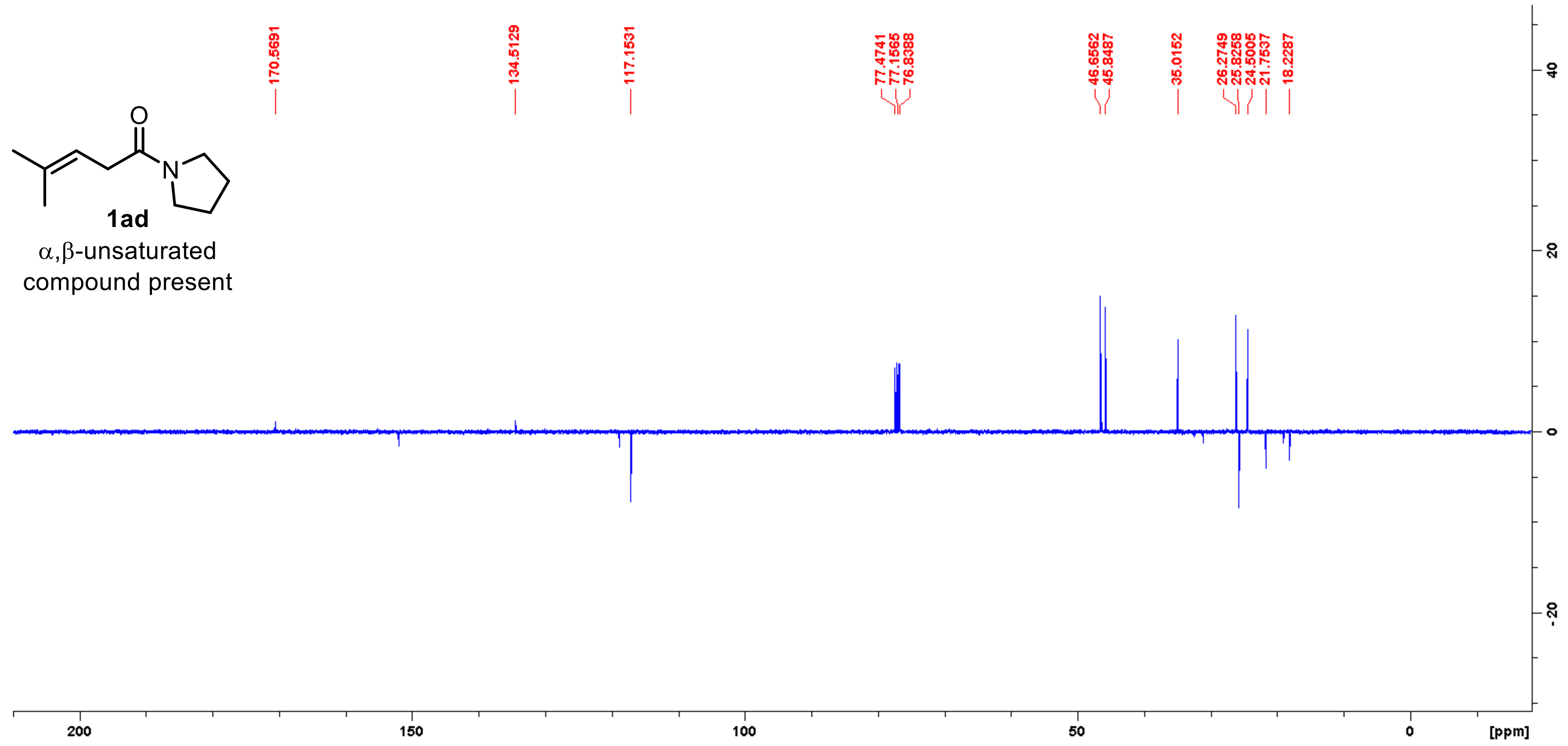

(*E*)-1-(Pyrrolidin-1-yl)pent-2-en-1-one (1ae)

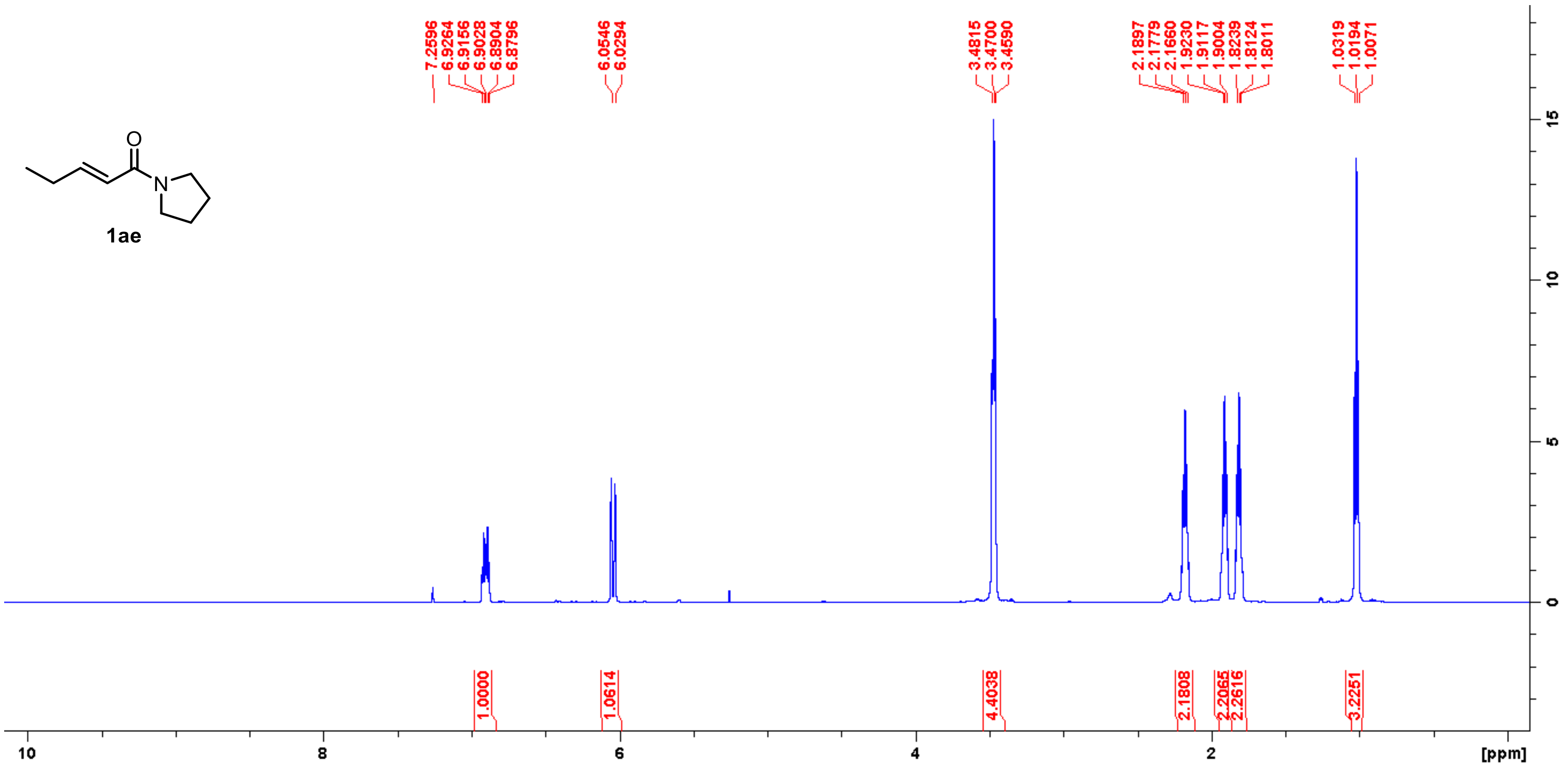

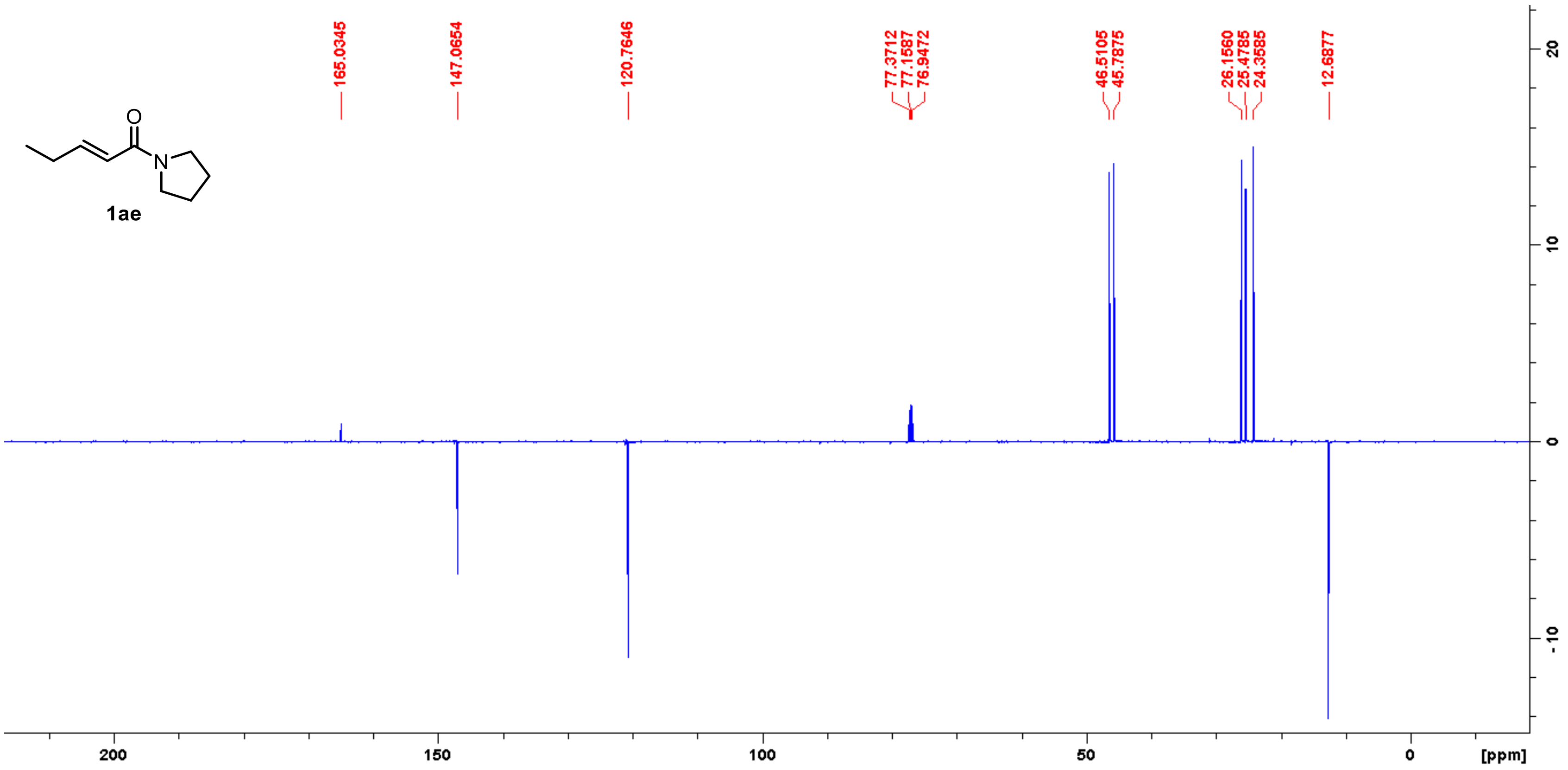

(*E*)-*N*,*N*-Dimethyldodec-3-enamide (1af)

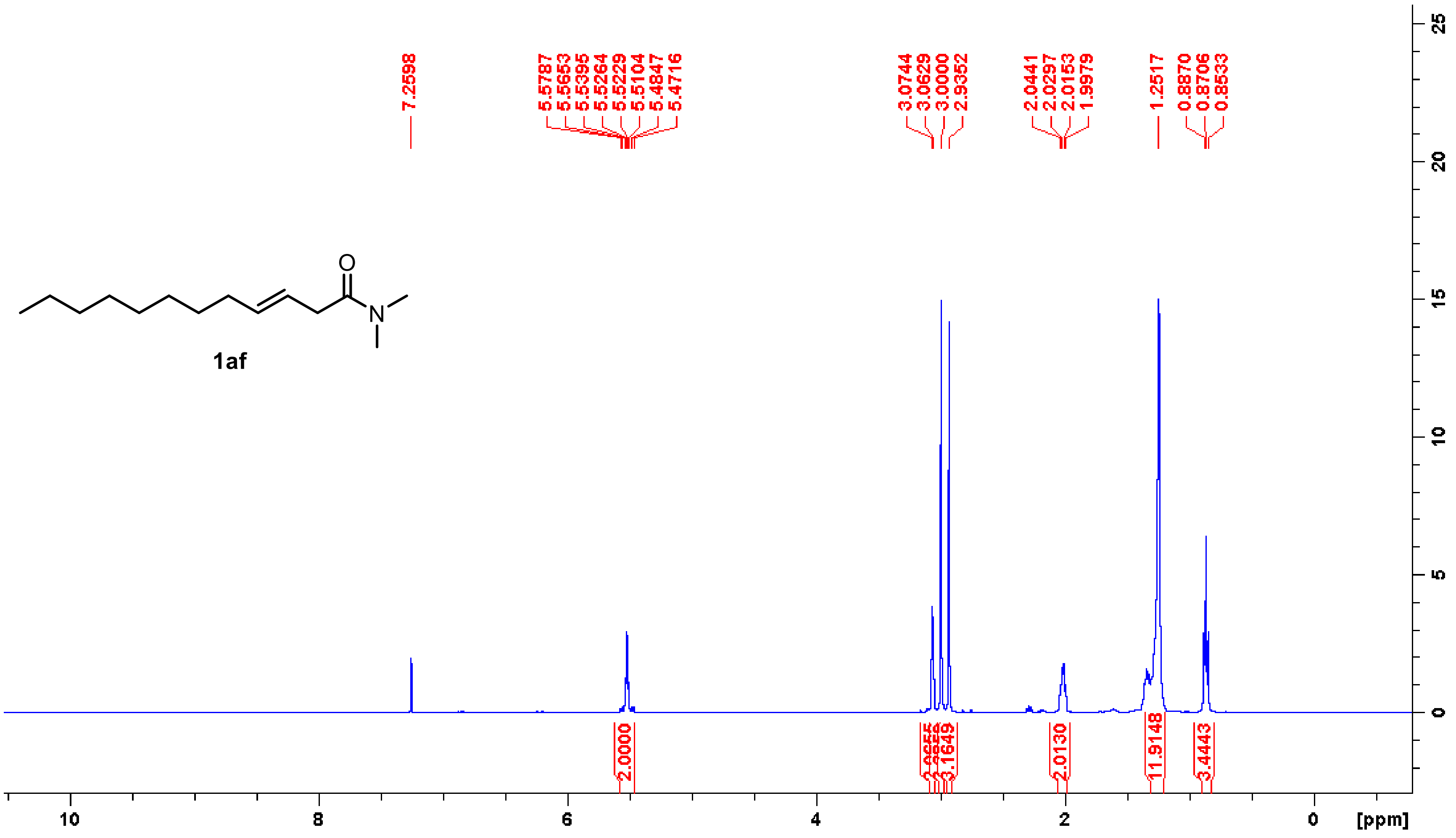

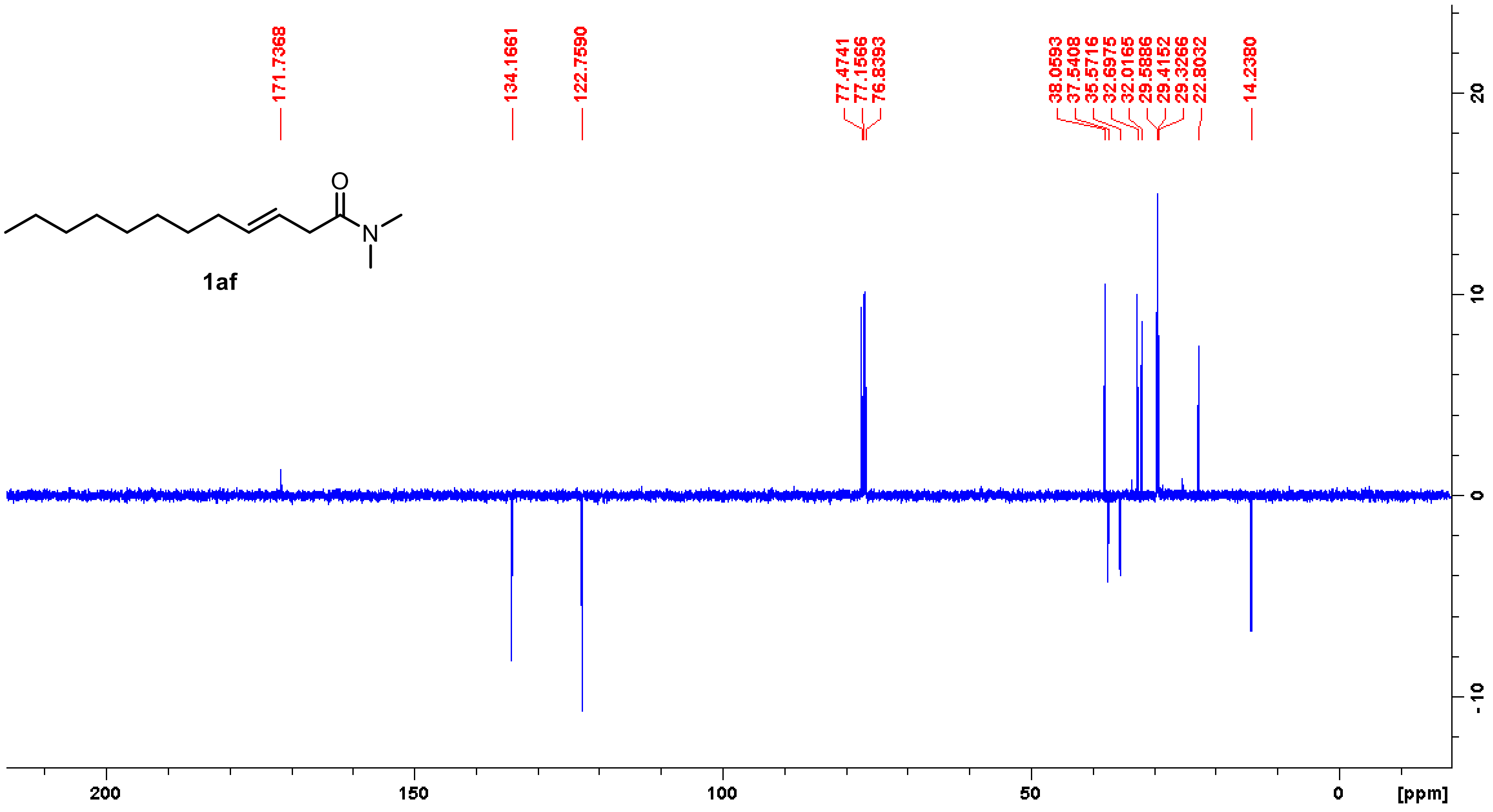

1-(Pyrrolidin-1-yl)hex-3-yn-1-one (1ag)

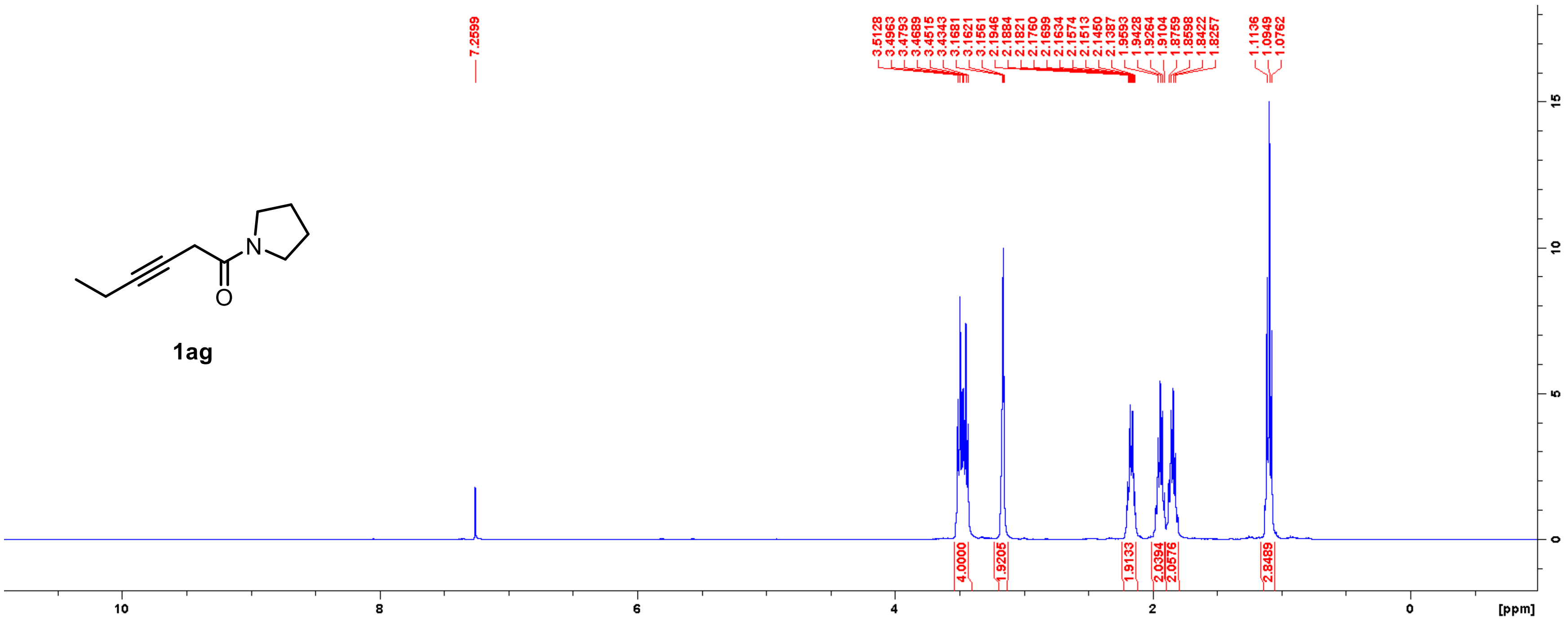

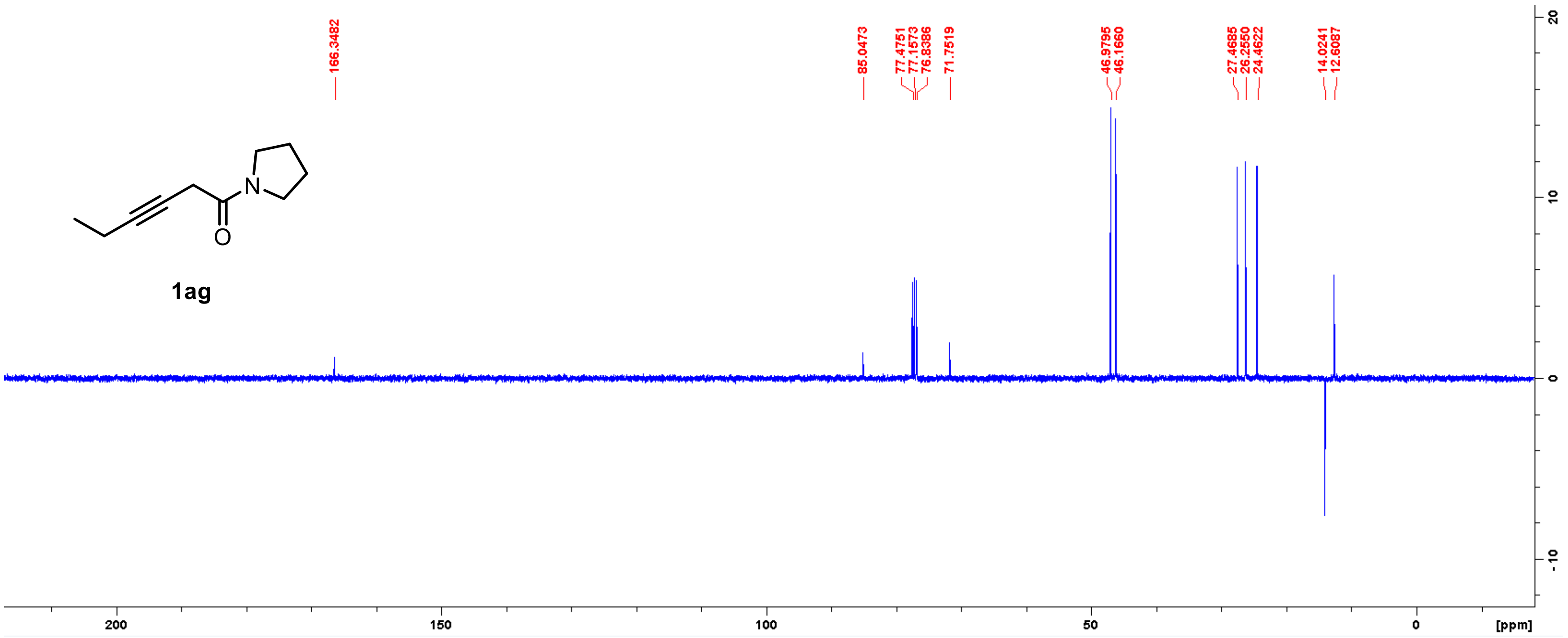

N-Butyl-3-methylbut-2-en-1-amine (8)

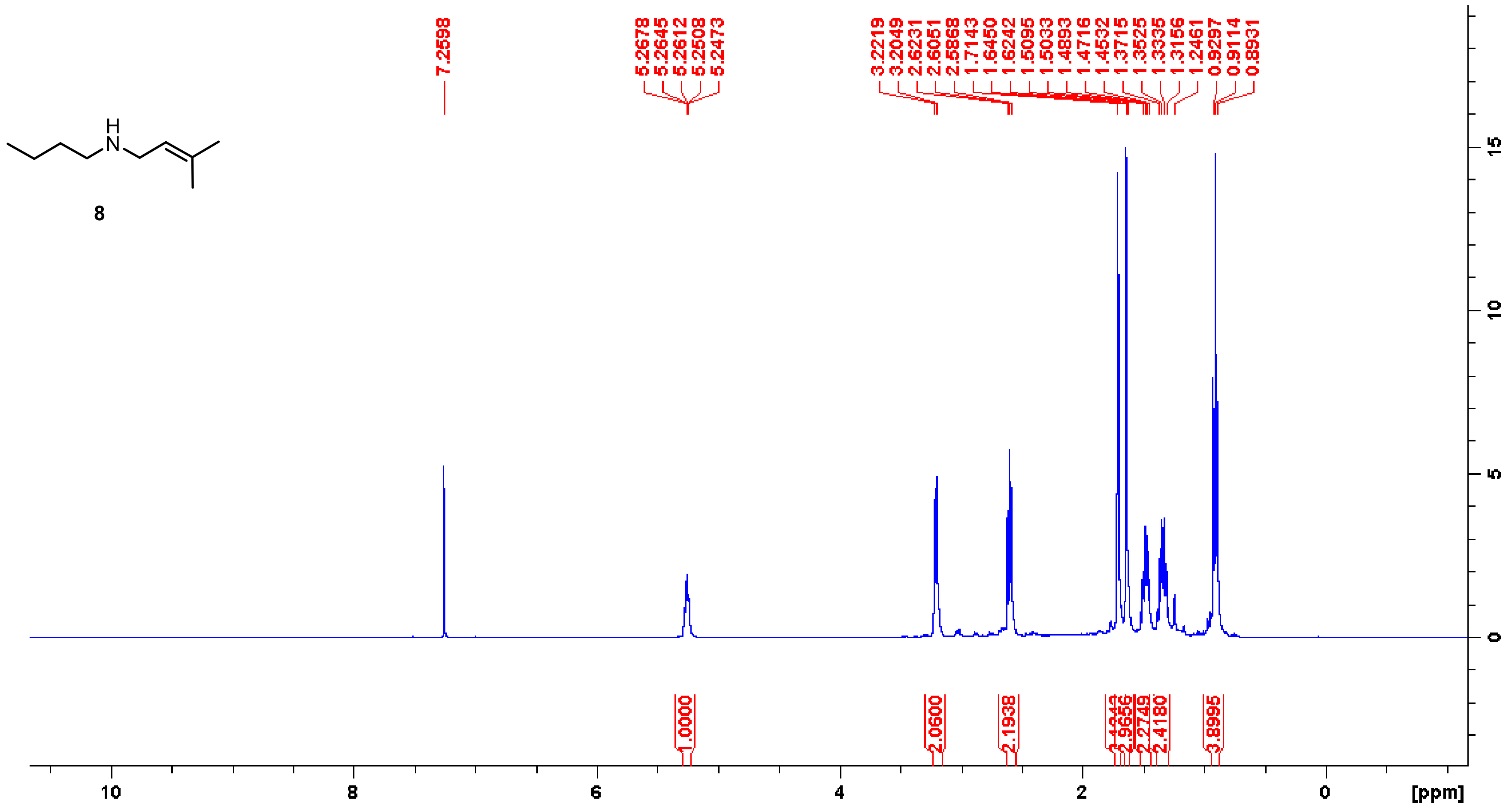

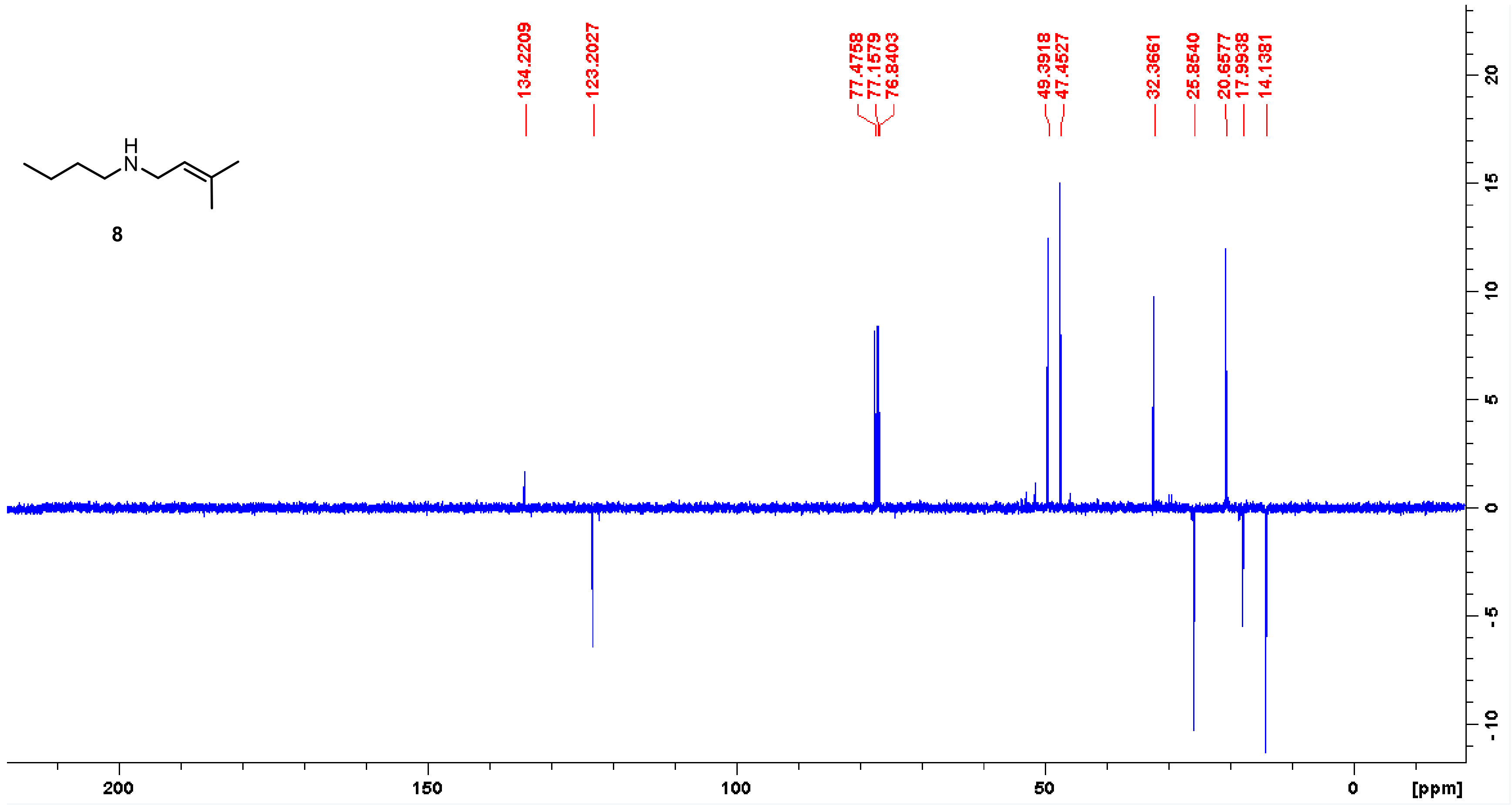

4.2 α,β-unsaturated-γ-OTMP amides

(*E*)-1-(Pyrrolidin-1-yl)-4-((2,2,6,6-tetramethylpiperidin-1-yl)-oxy)-pent-2-en-1-one (2a)

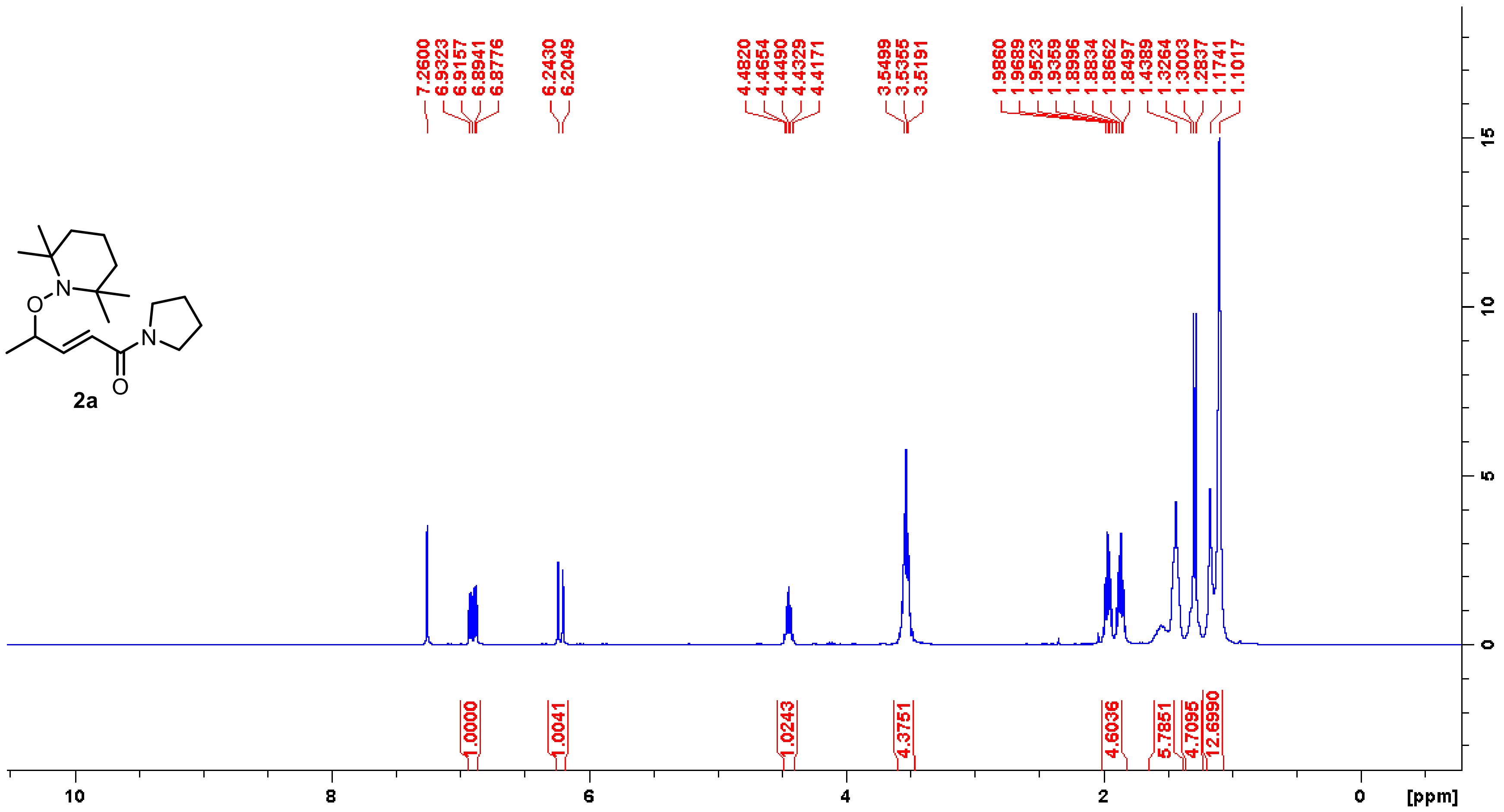

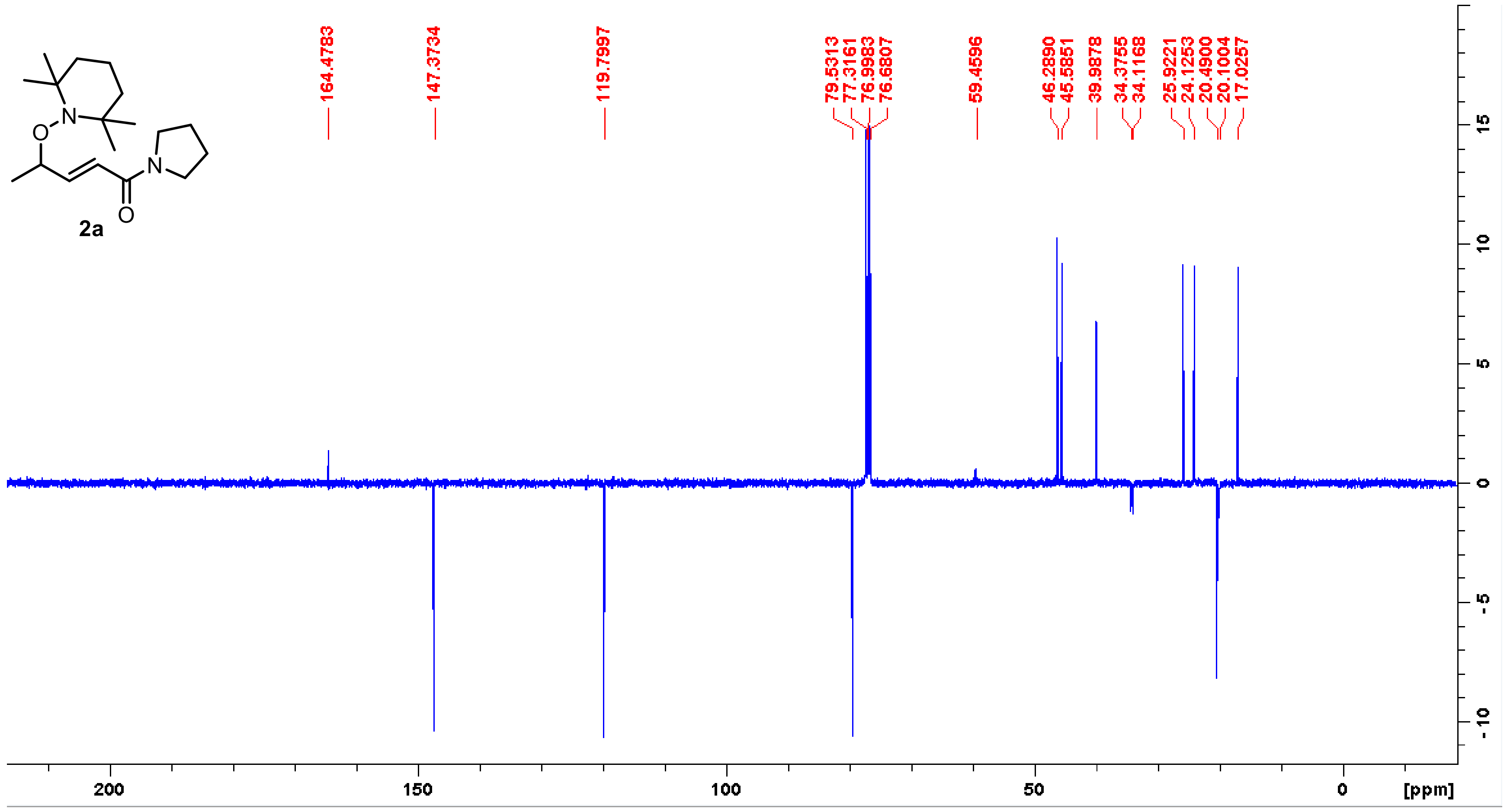

(*E*)-1-(Pyrrolidin-1-yl)-4-((2,2,6,6-tetramethylpiperidin-1-yl)-oxy)-dodec-2-en-1-one (2b)

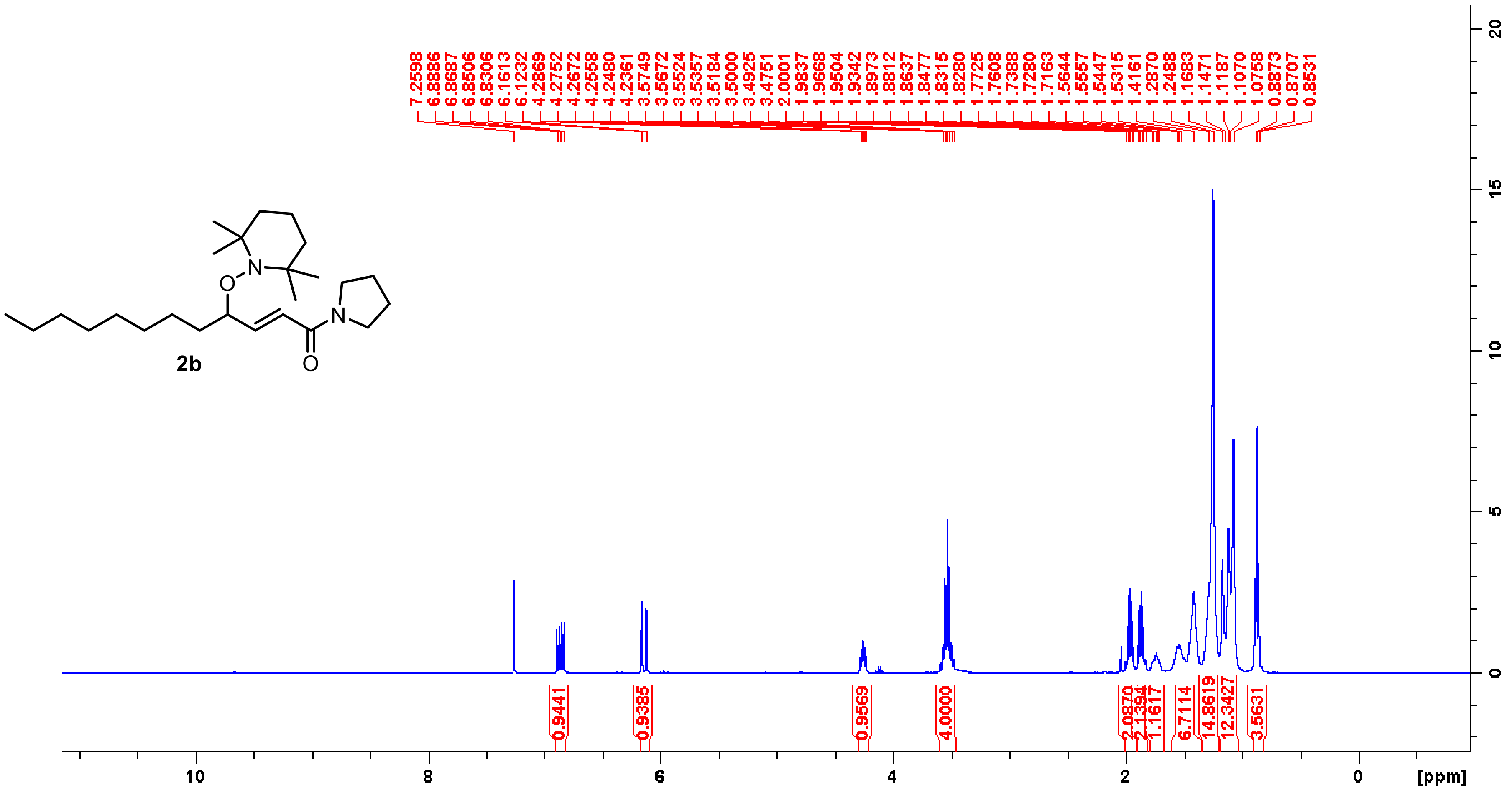

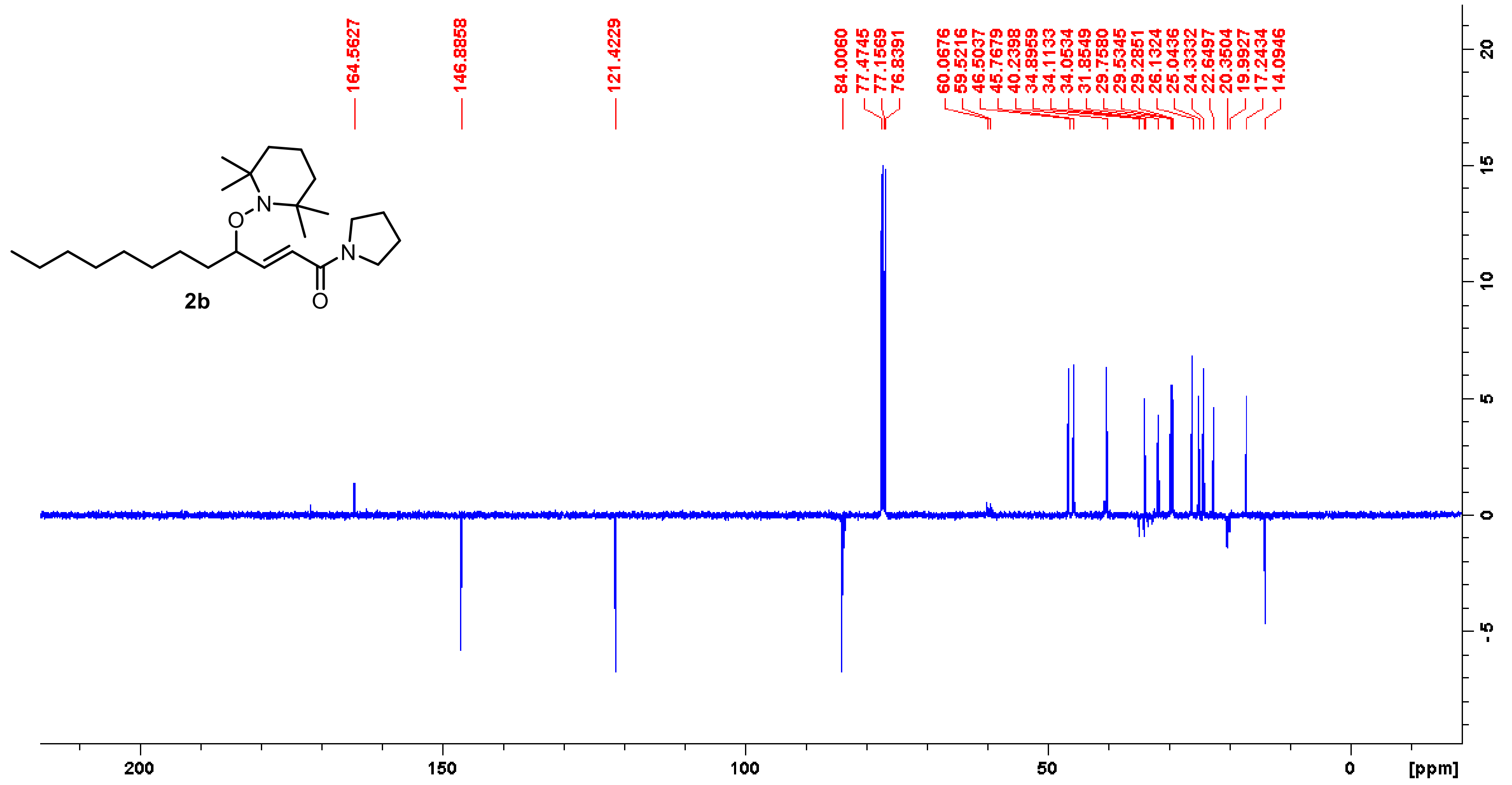

(*E*)-5-Phenyl-1-(pyrrolidin-1-yl)-4-((2,2,6,6-tetramethylpiperidin-1-yl)-oxy)-pent-2-en-1-one (2c)

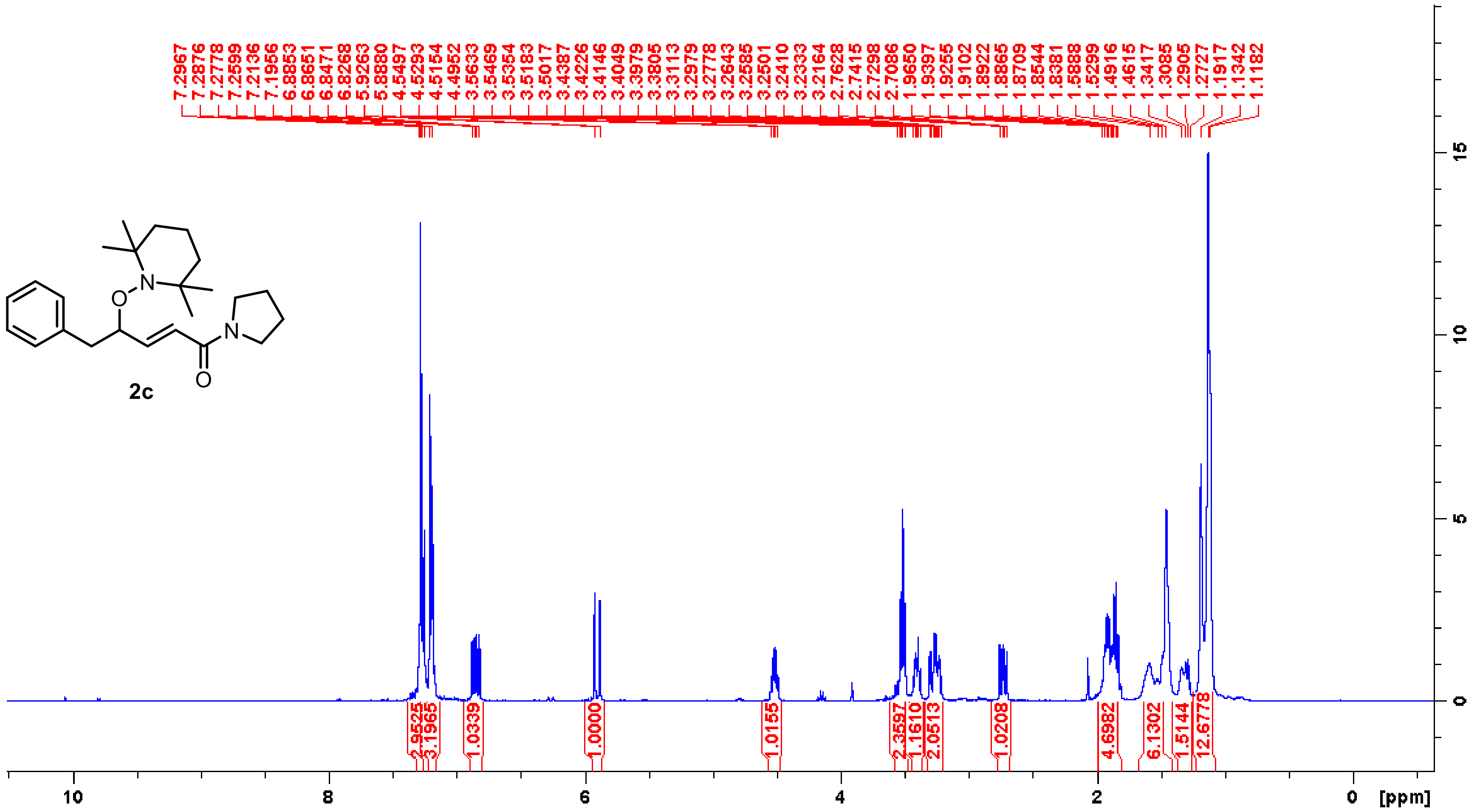

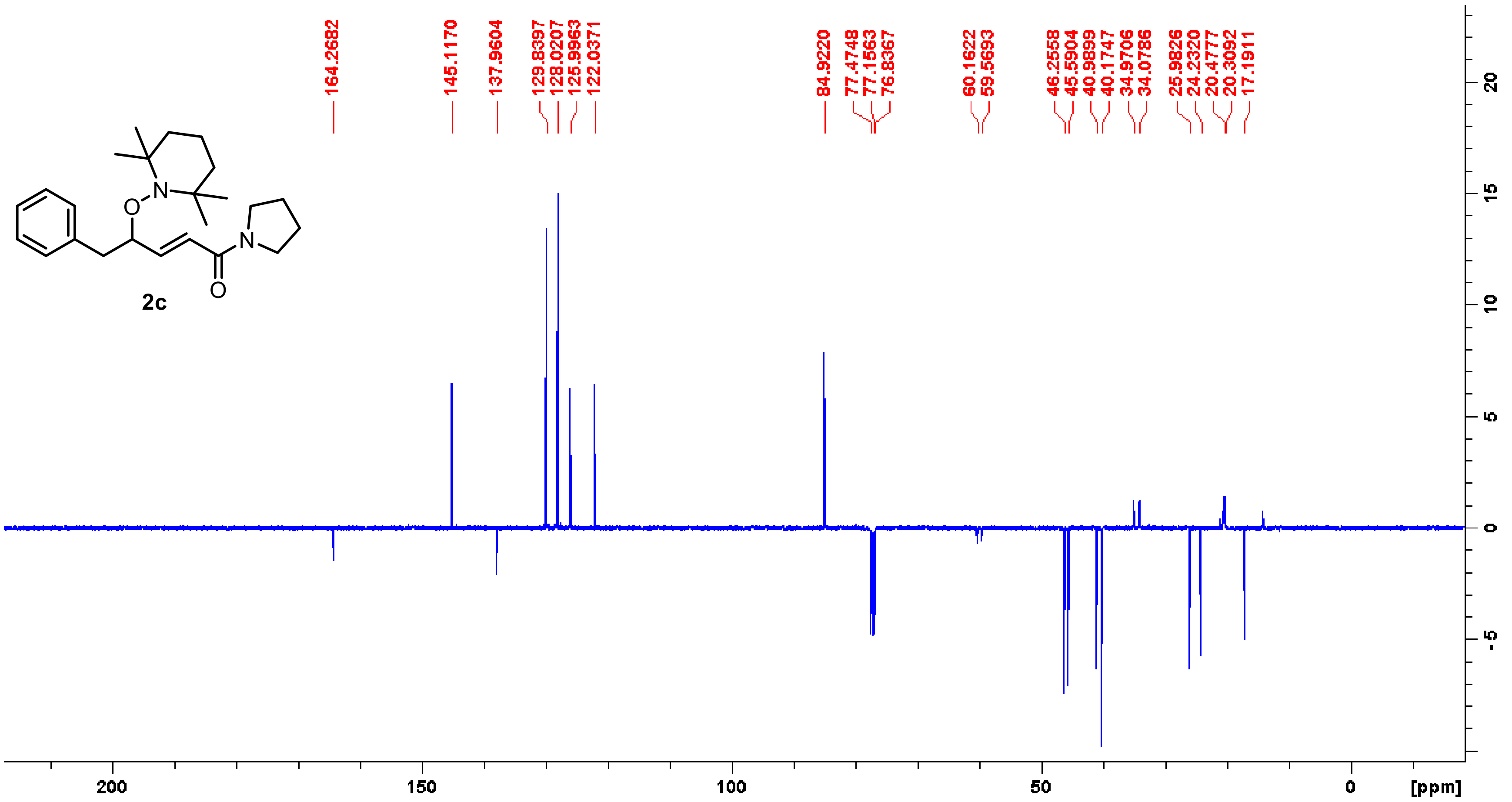

(*E*)-1-(Pyrrolidin-1-yl)-4-((2,2,6,6-tetramethylpiperidin-1-yl)oxy)but-2-en-1-one (2d)

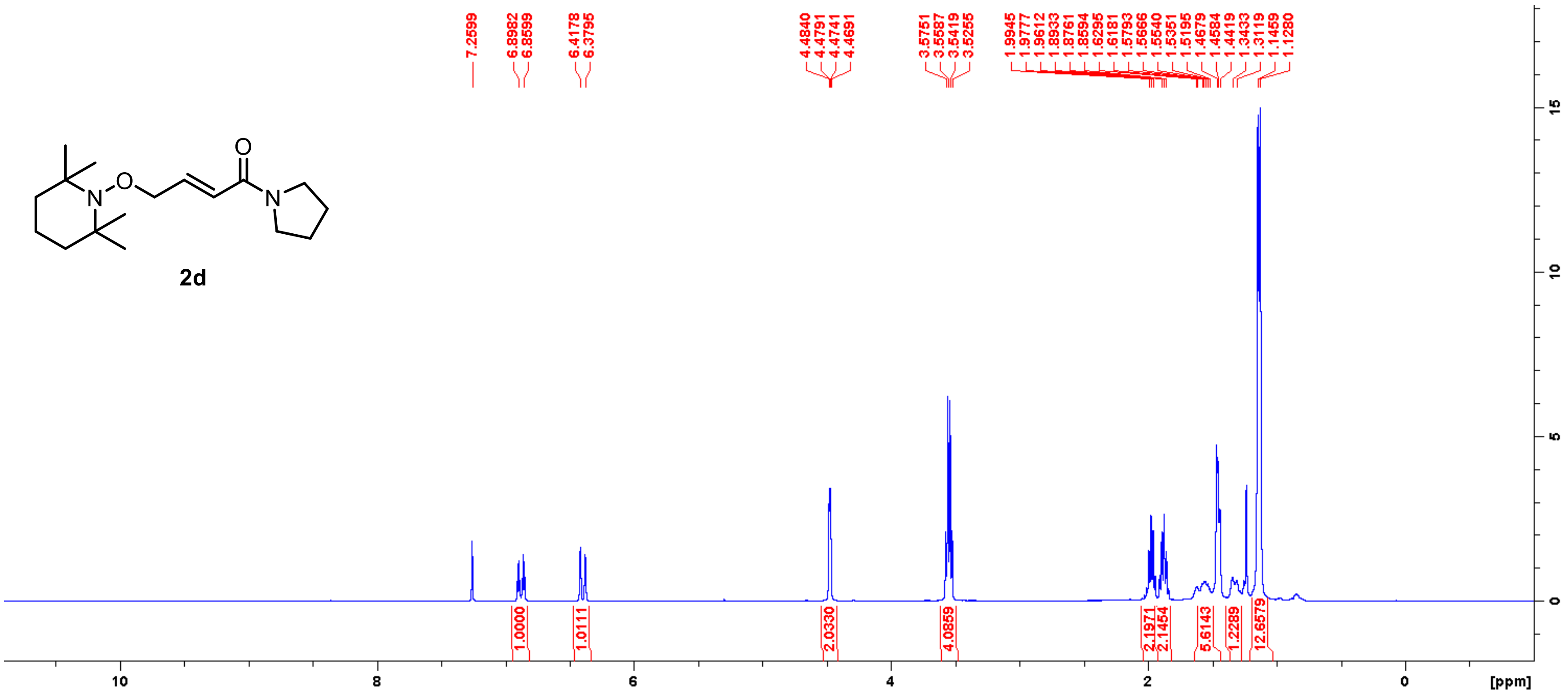

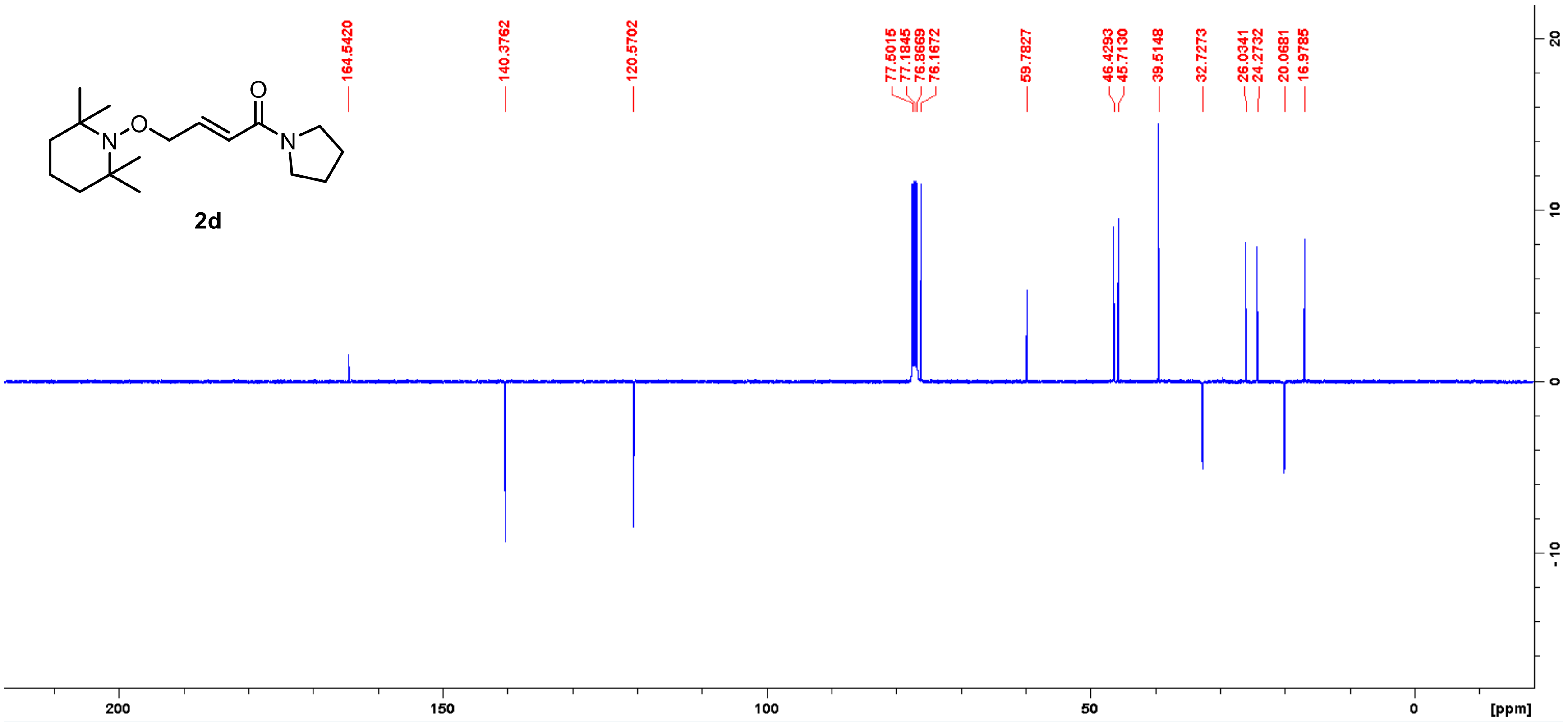

(*E*)-1-(Pyrrolidin-1-yl)-4-((2,2,6,6-tetramethylpiperidin-1-yl)-oxy)-penta-2,4-dien-1-one (2e)

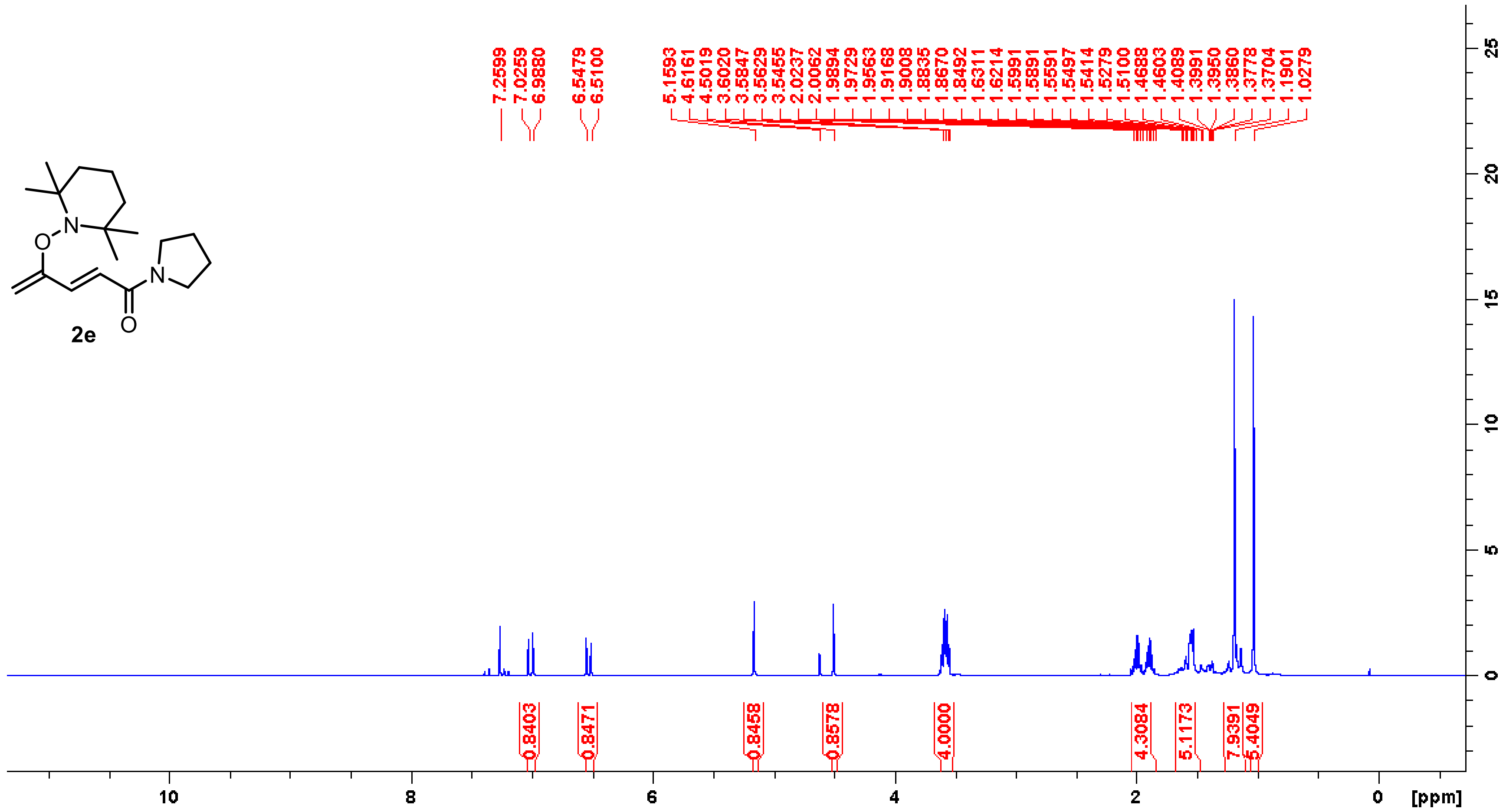

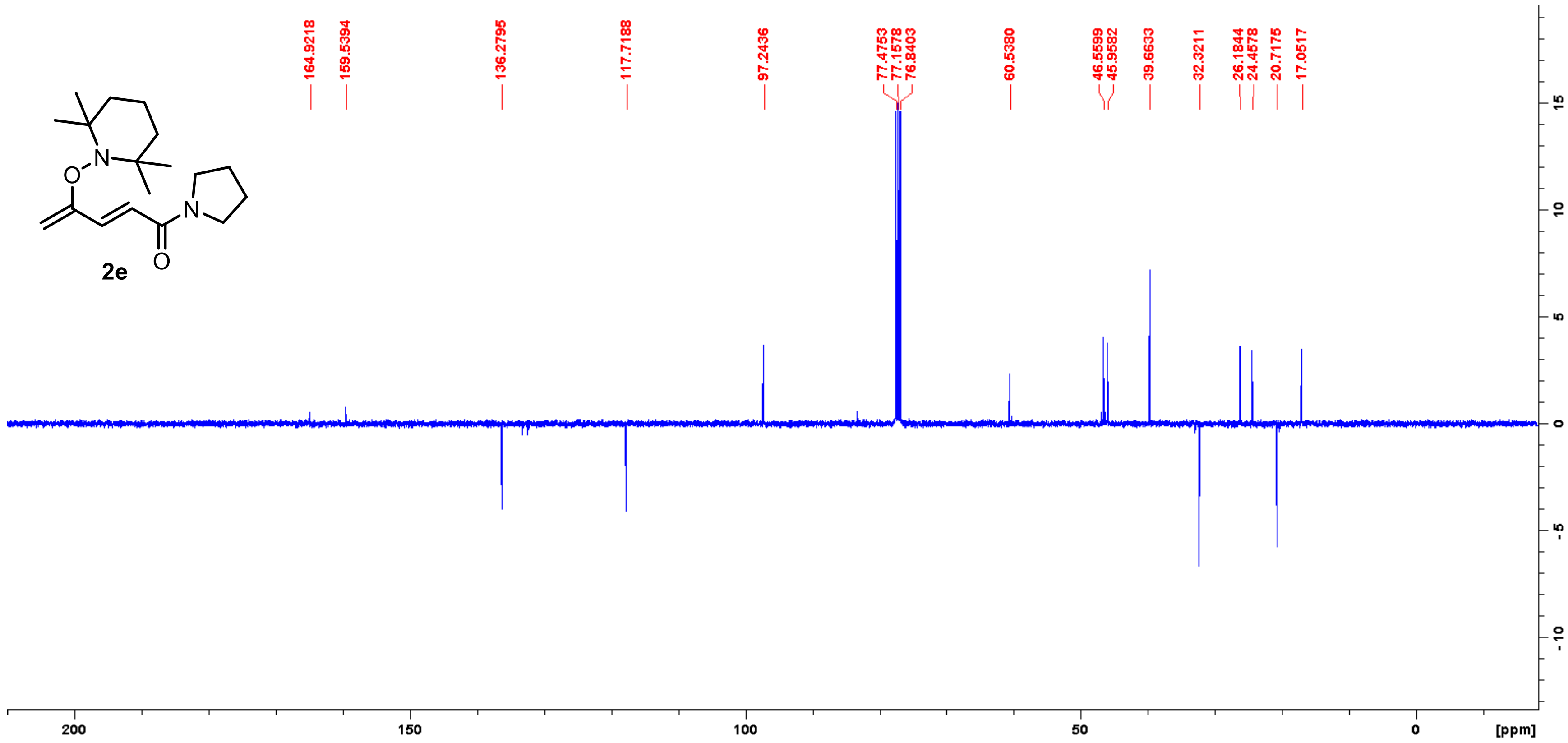

(*E*)-2-Methyl-1-(pyrrolidin-1-yl)-4-((2,2,6,6-tetramethylpiperidin-1-yl)-oxy)-pent-2-en-1-one (2f)

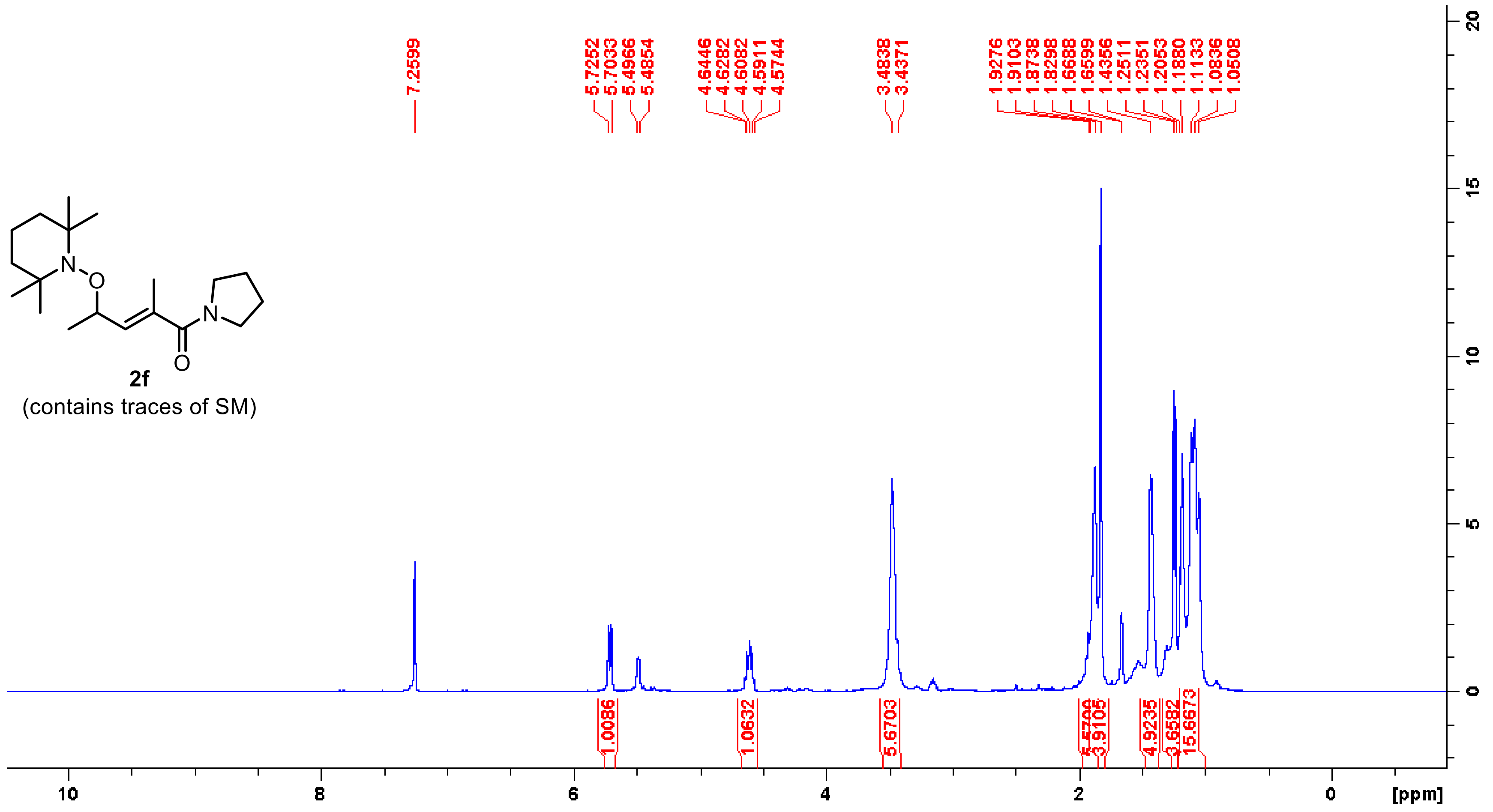

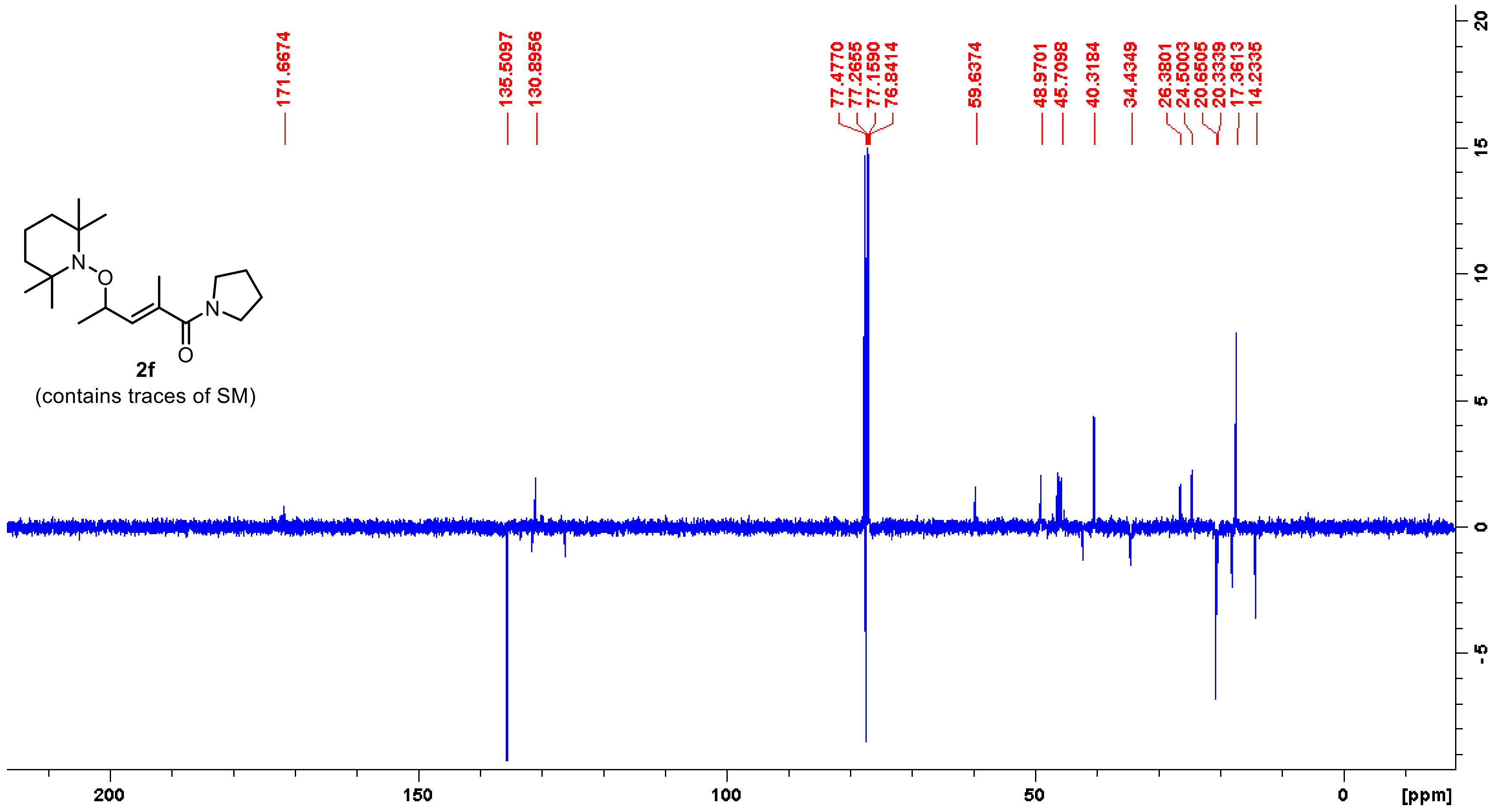

(*E*)-*N*-Cyclopropyl-*N*,2-dimethyl-4-((2,2,6,6-tetramethylpiperidin-1-yl)oxy)pent-2-enamide (2g)

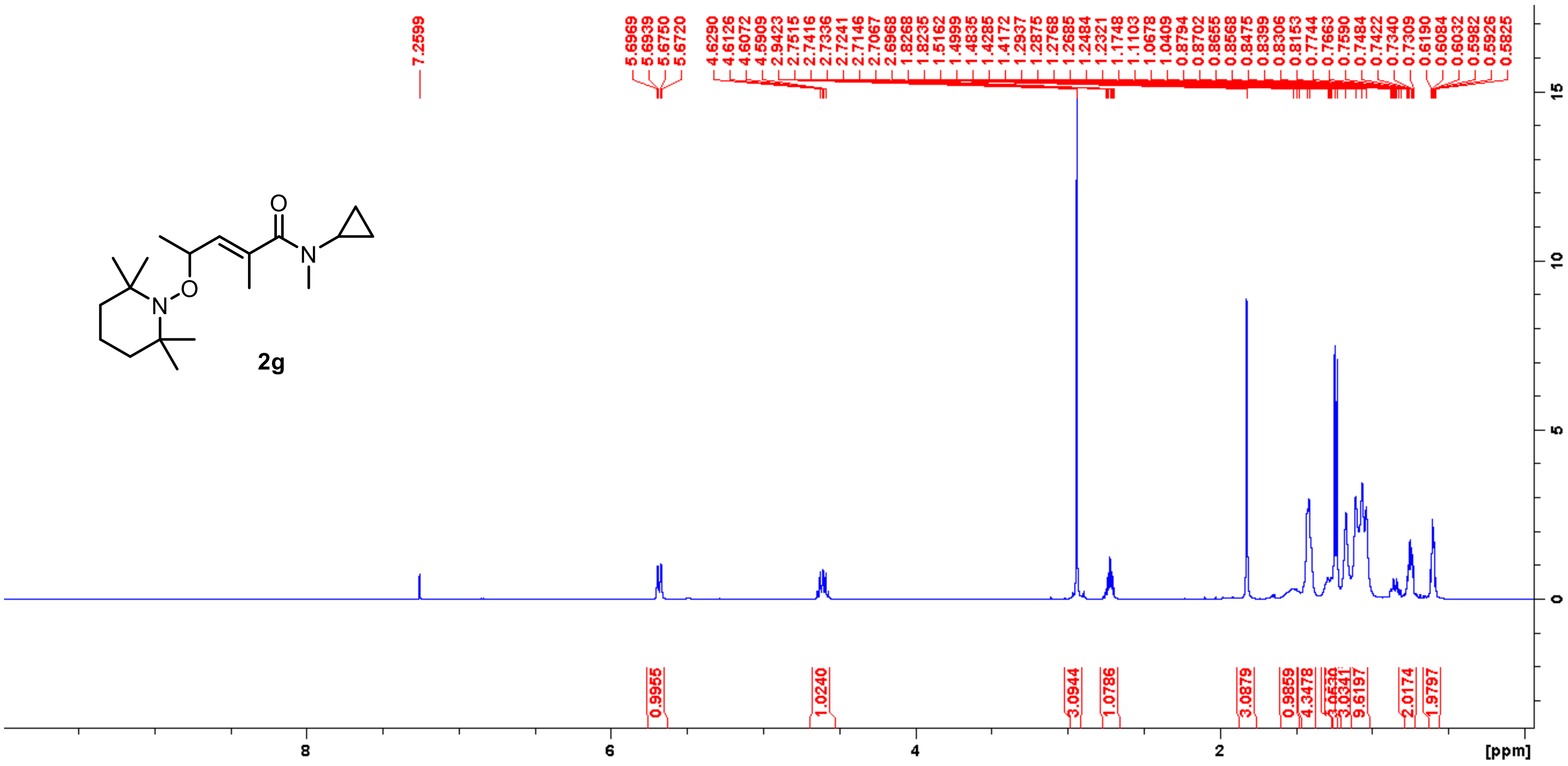

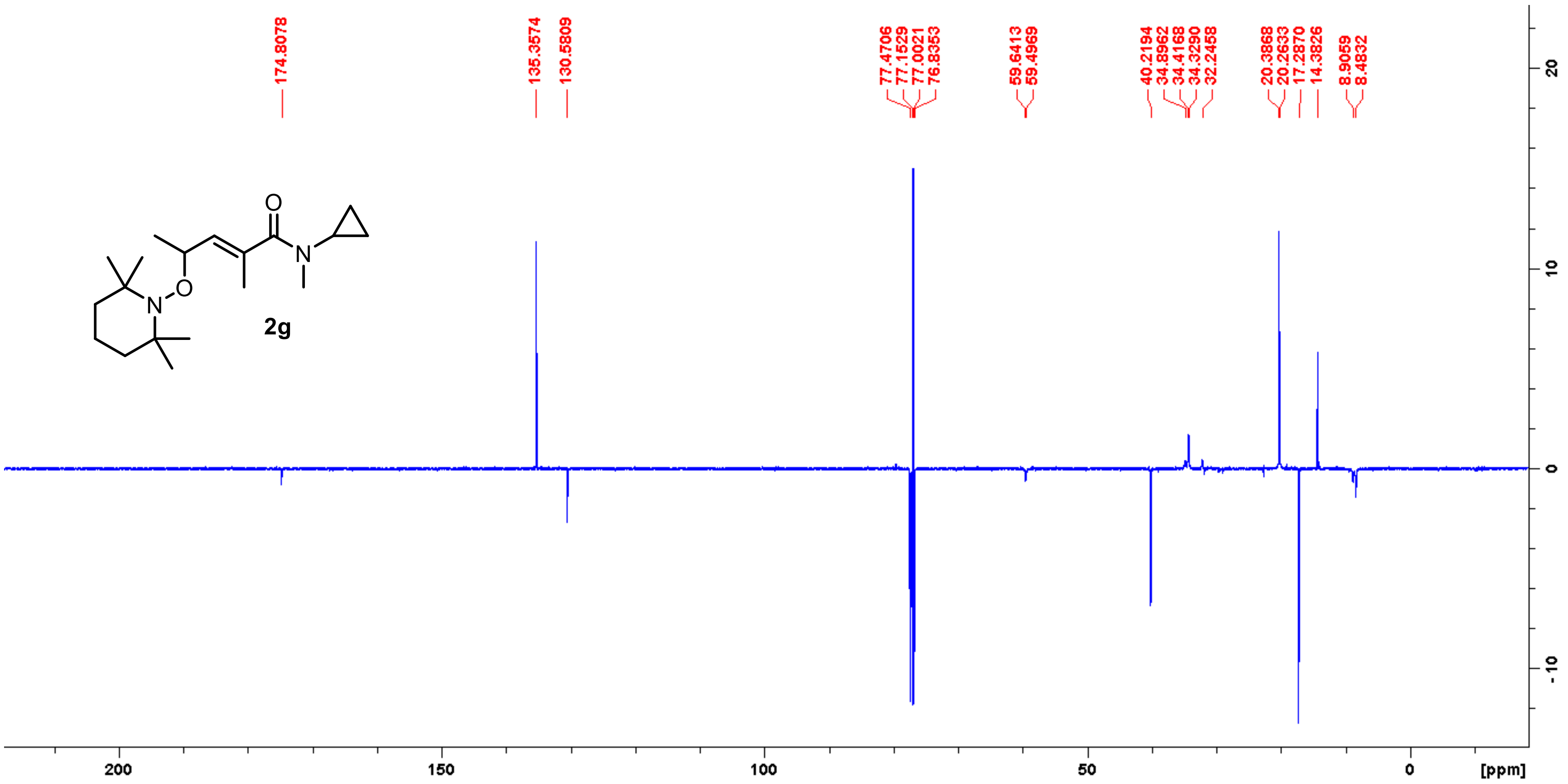

(*E*)-1-(Pyrrolidin-1-yl)-2-(2-((2,2,6,6-tetramethylpiperidin-1-yl)-oxy)-cyclohexylidene)-ethan-1-one (2h)

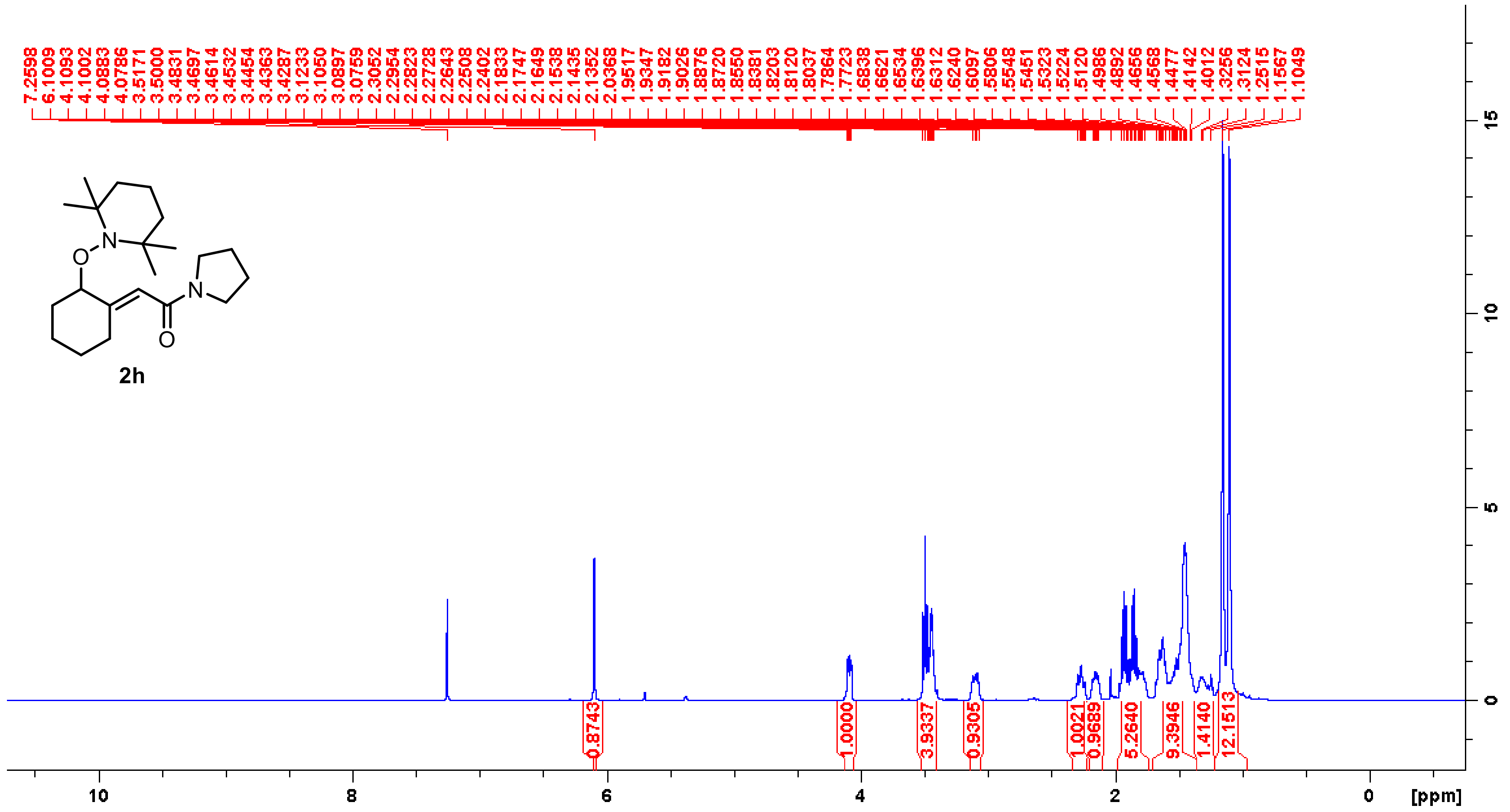

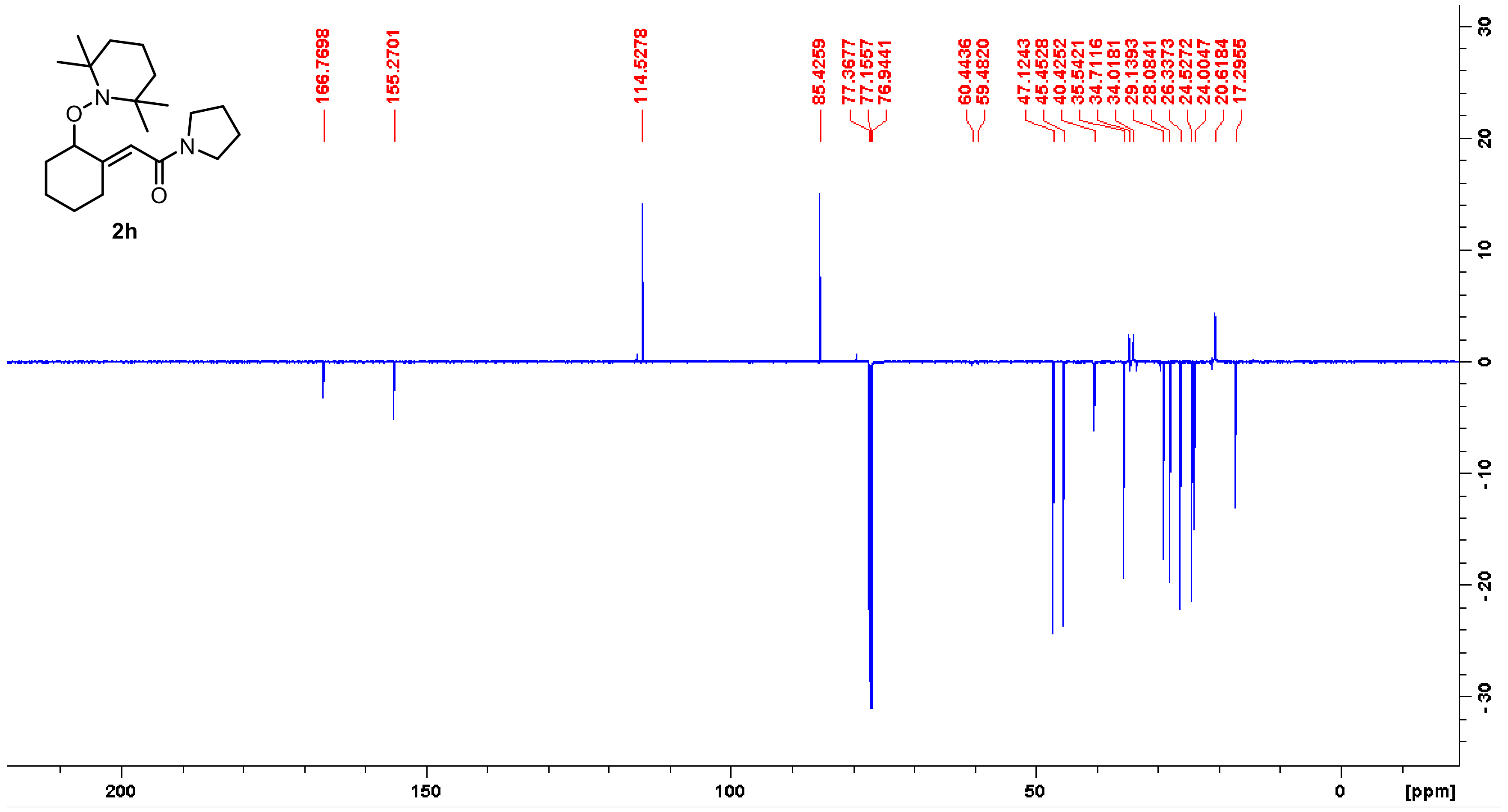

Methyl-(*E*)-12-oxo-12-(pyrrolidin-1-yl)-9-((2,2,6,6-tetramethylpiperidin-1-yl)-oxy)-dodec-10-enoate (2i)

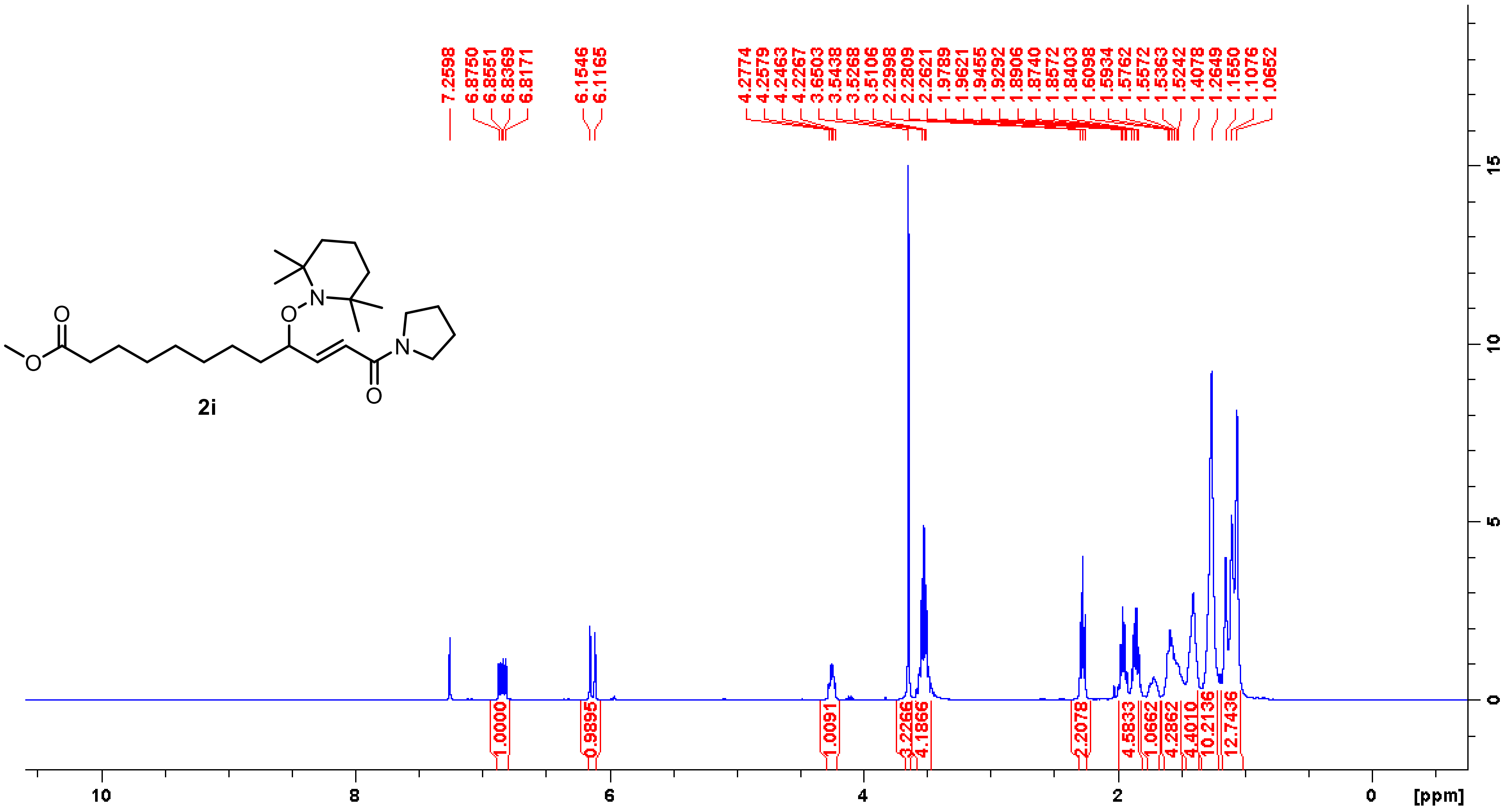

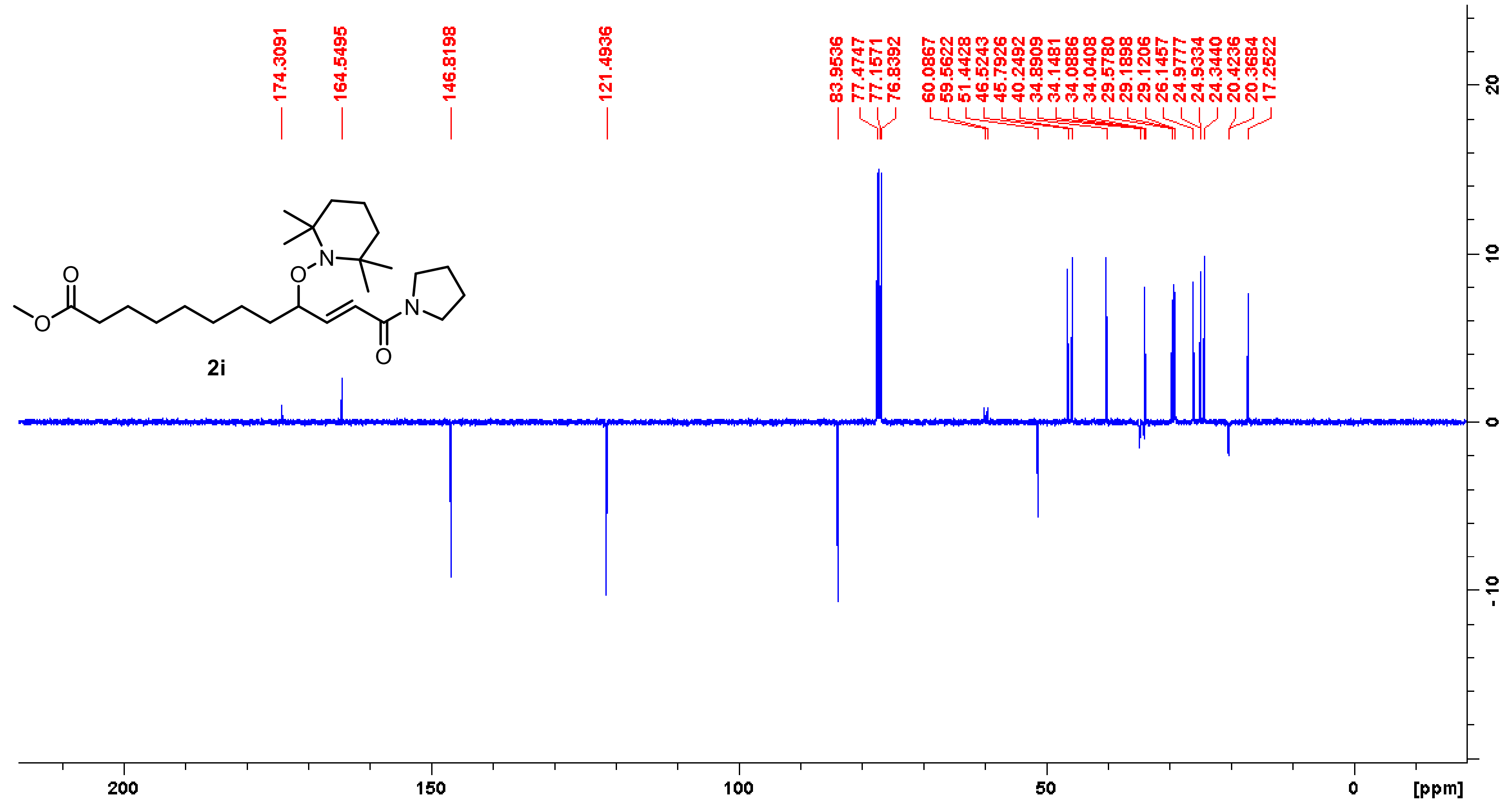

(*E*)-1-(Pyrrolidin-1-yl)-4-((2,2,6,6-tetramethylpiperidin-1-yl)oxy)dodec-2-ene-1,11-dione (2j)

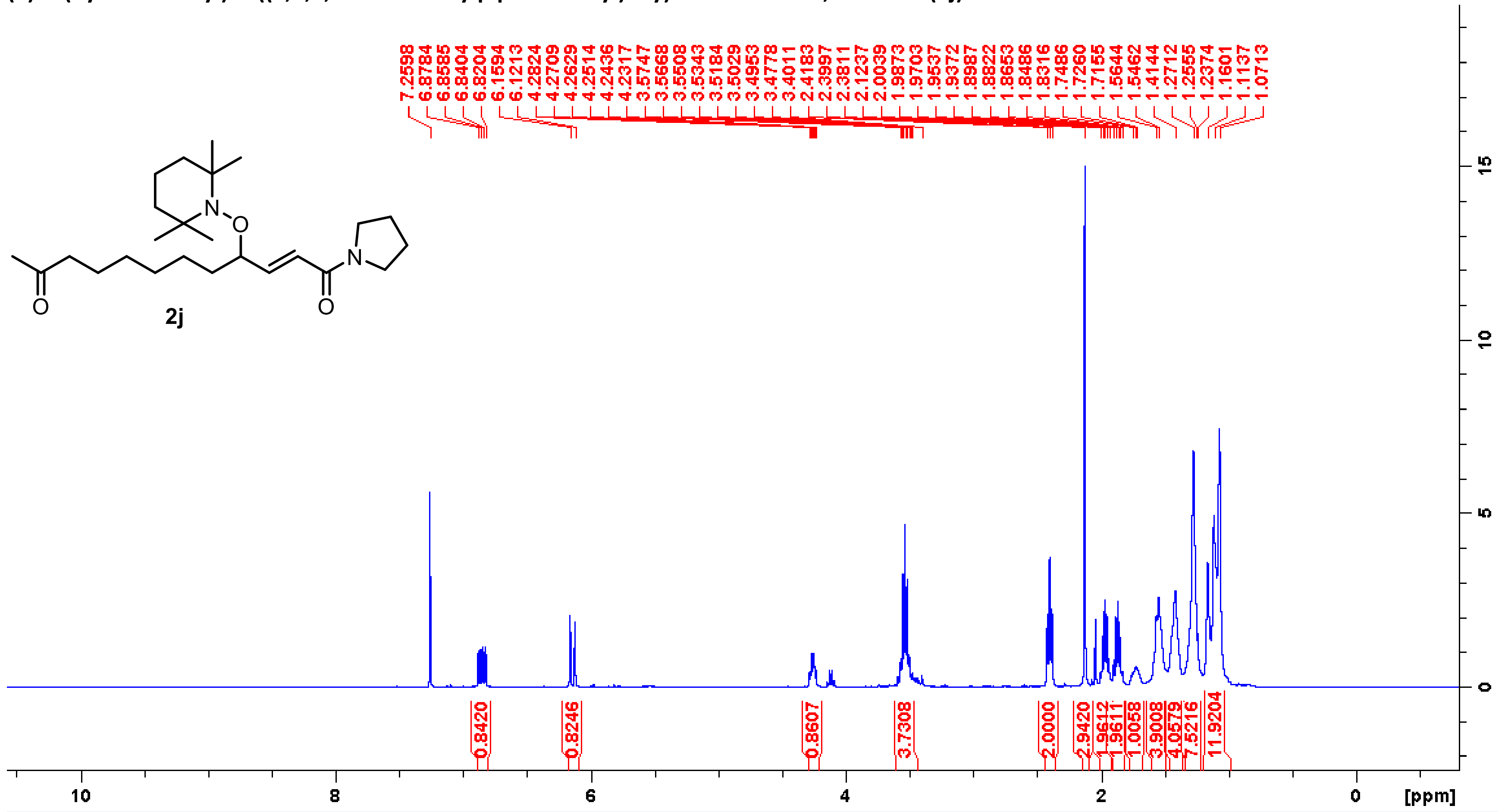

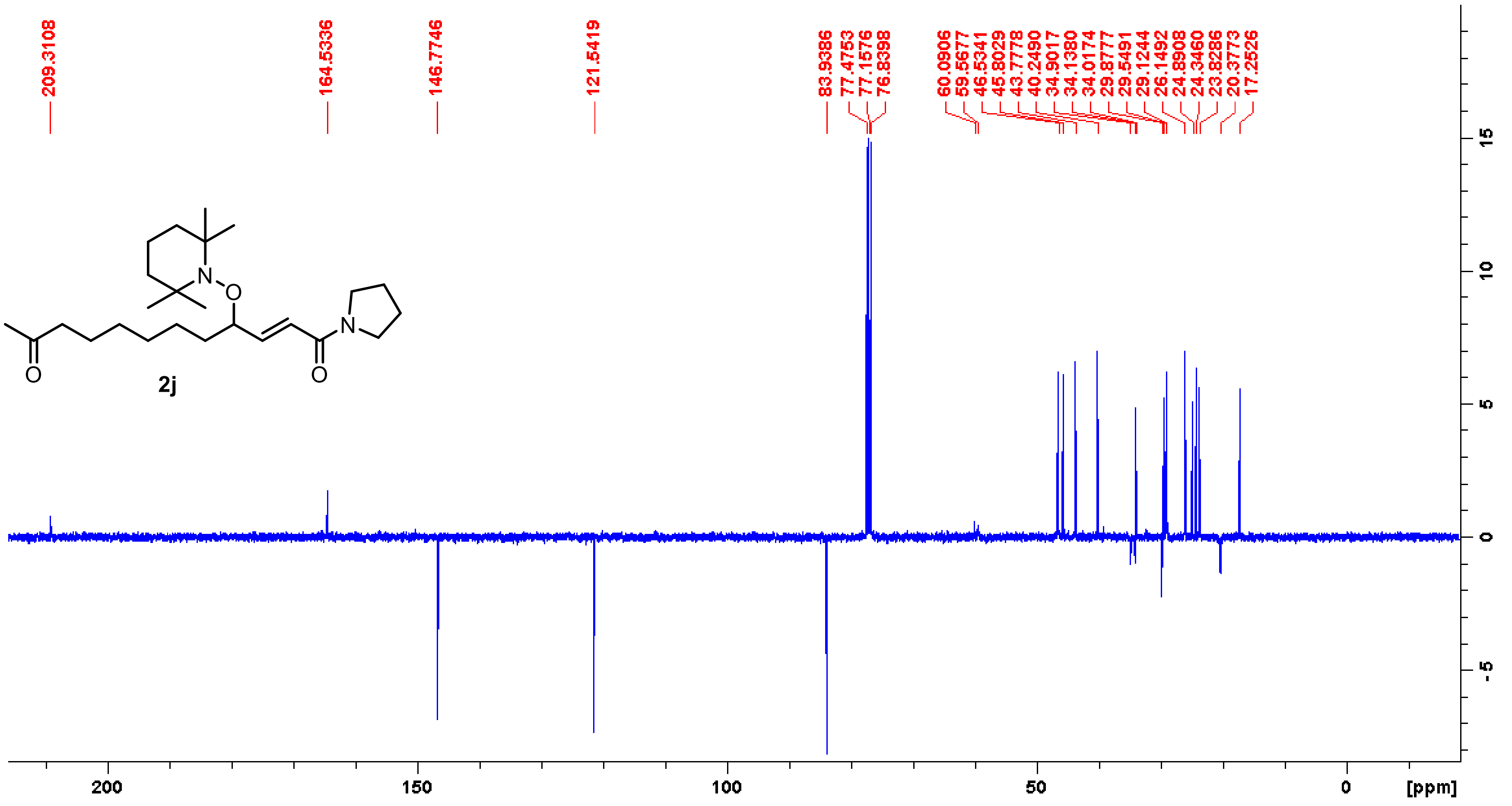

(*E*)-9-Oxo-9-(pyrrolidin-1-yl)-6-((2,2,6,6-tetramethylpiperidin-1-yl)-oxy)-non-7-enenitrile (2k)

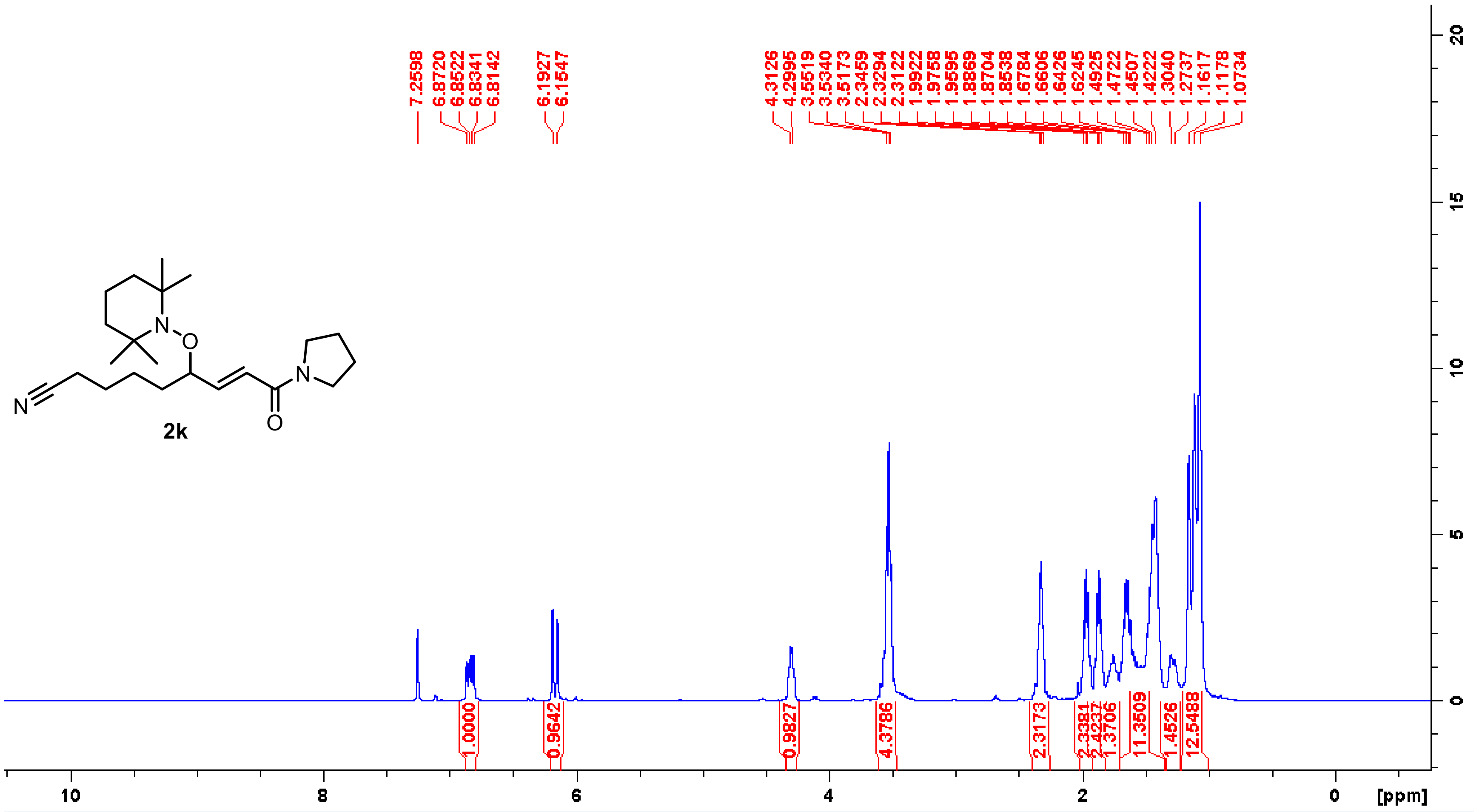

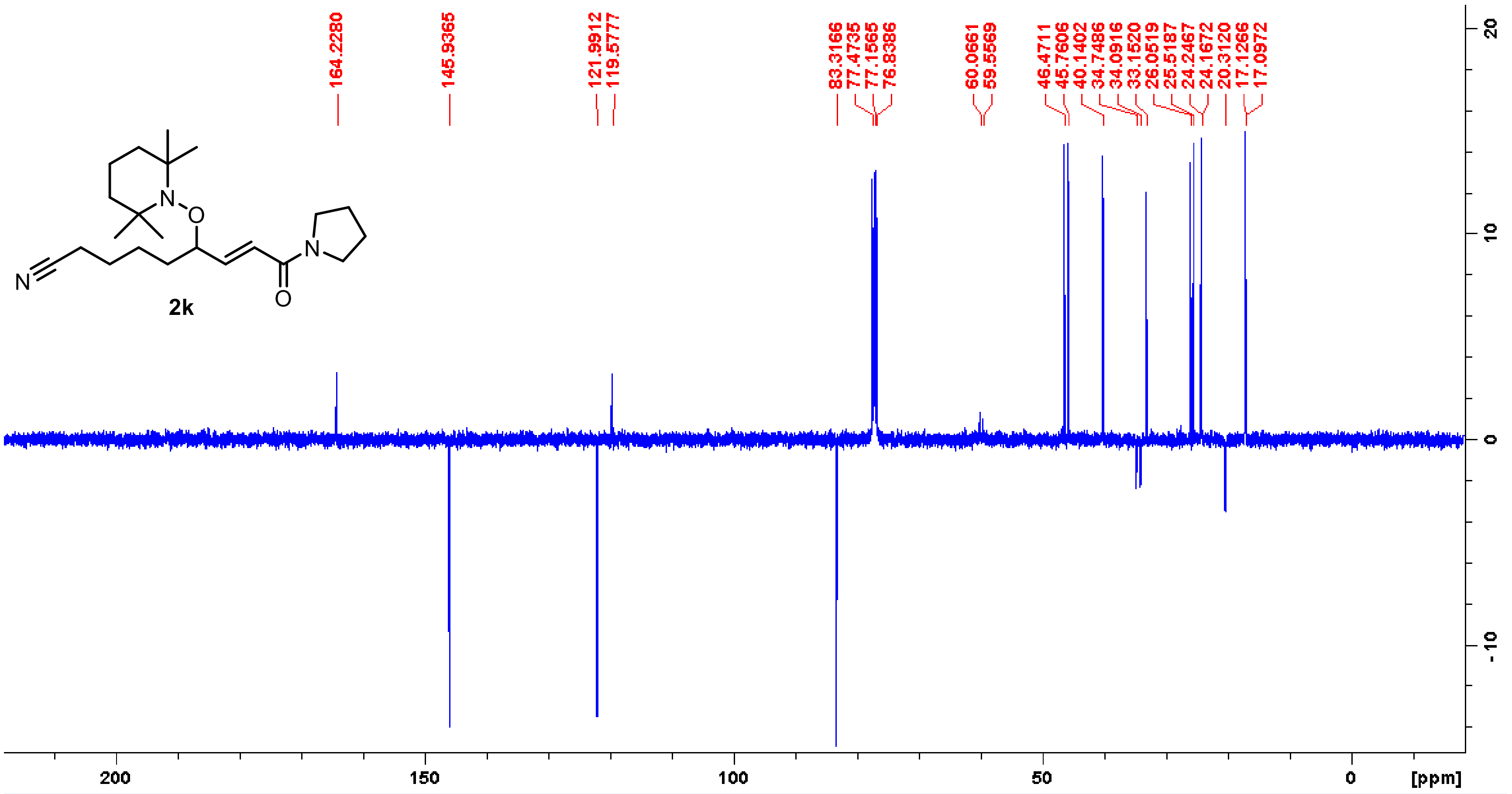

(*E*)-8-Chloro-1-(pyrrolidin-1-yl)-4-((2,2,6,6-tetramethylpiperidin-1-yl)-oxy)-oct-2-en-1-one (2l)

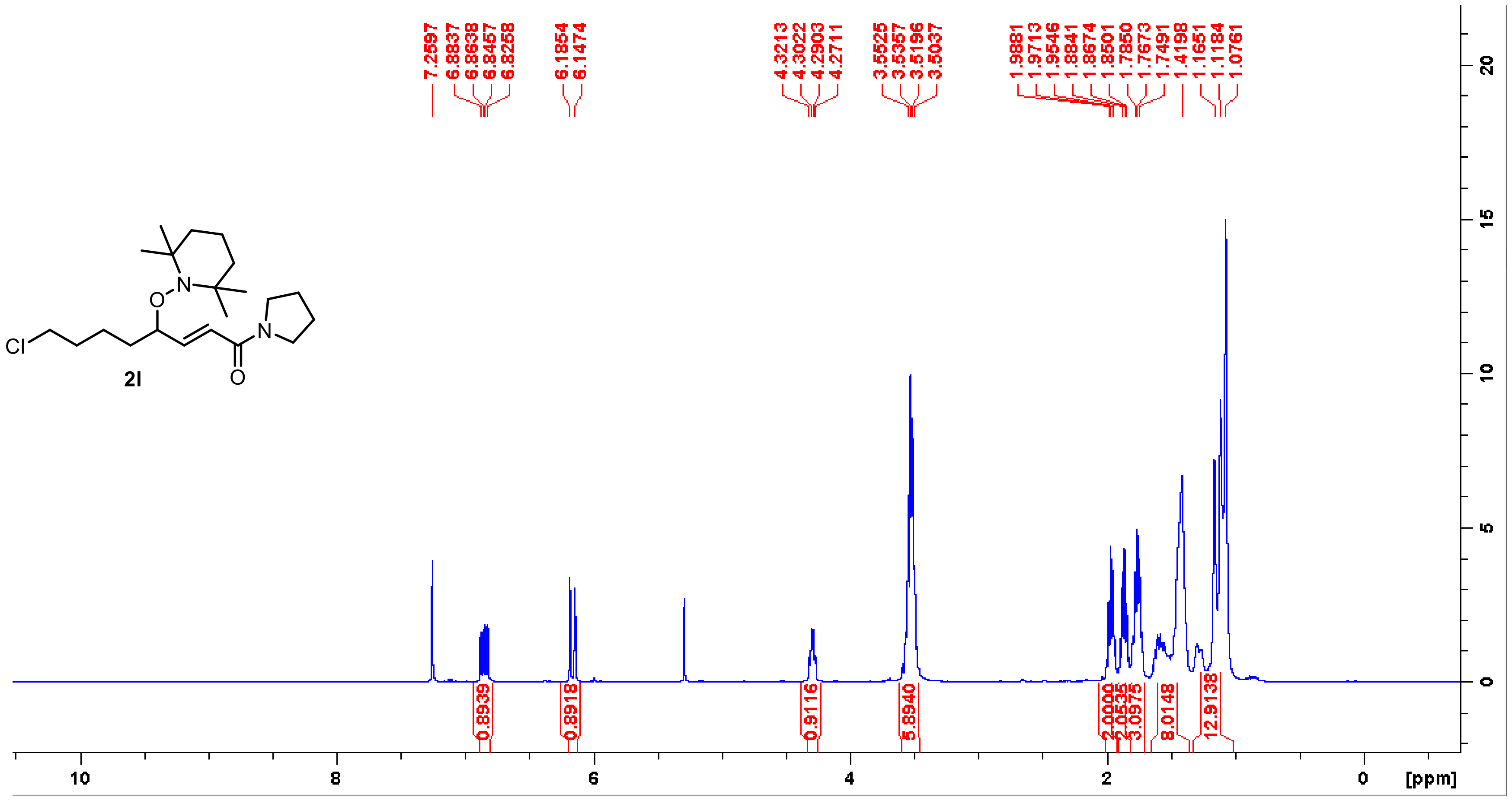

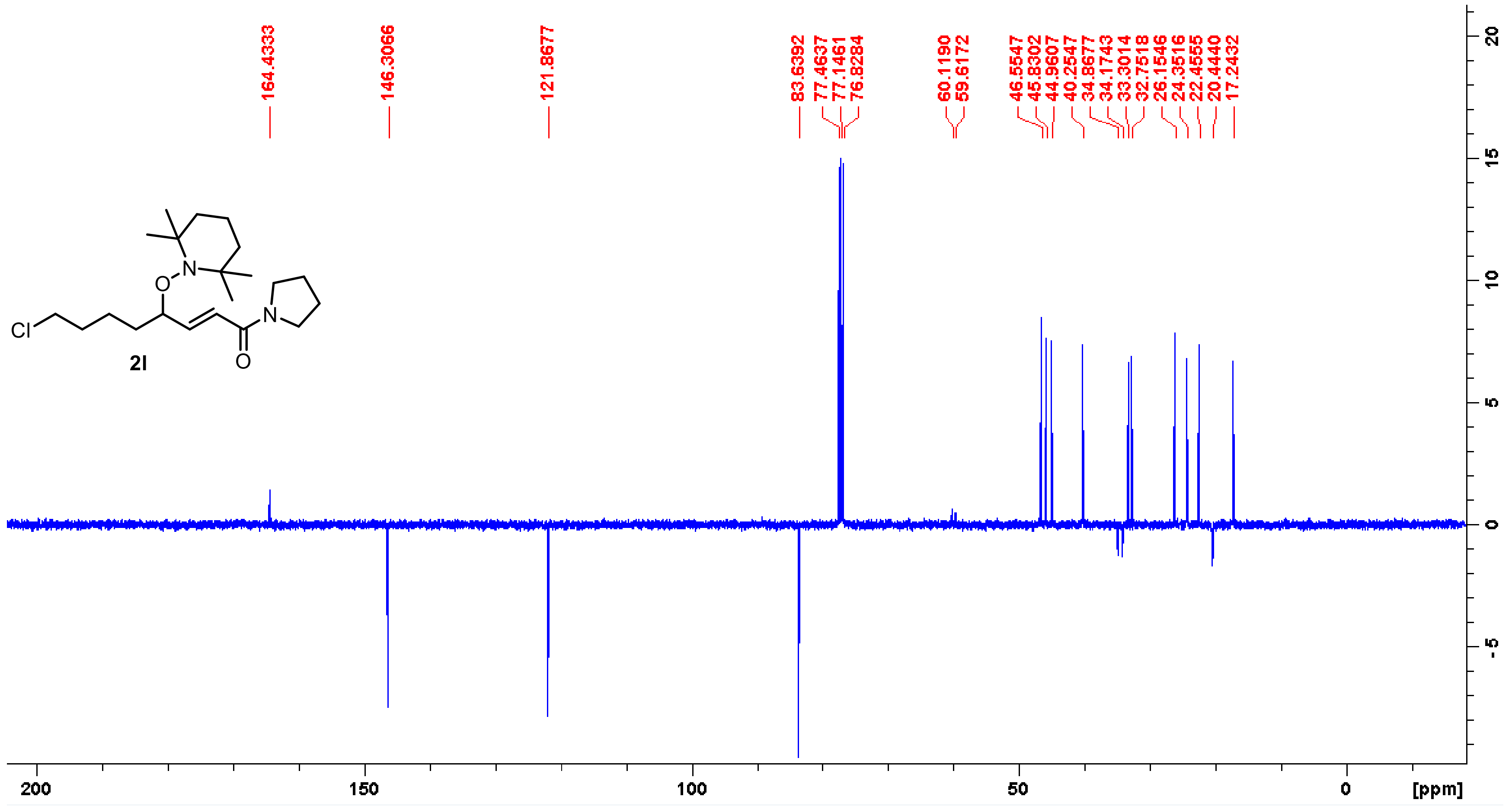

(*E*)-1-(Pyrrolidin-1-yl)-4-((2,2,6,6-tetramethylpiperidin-1-yl)-oxy)-dodec-2-en-11-yn-1-one (2m)

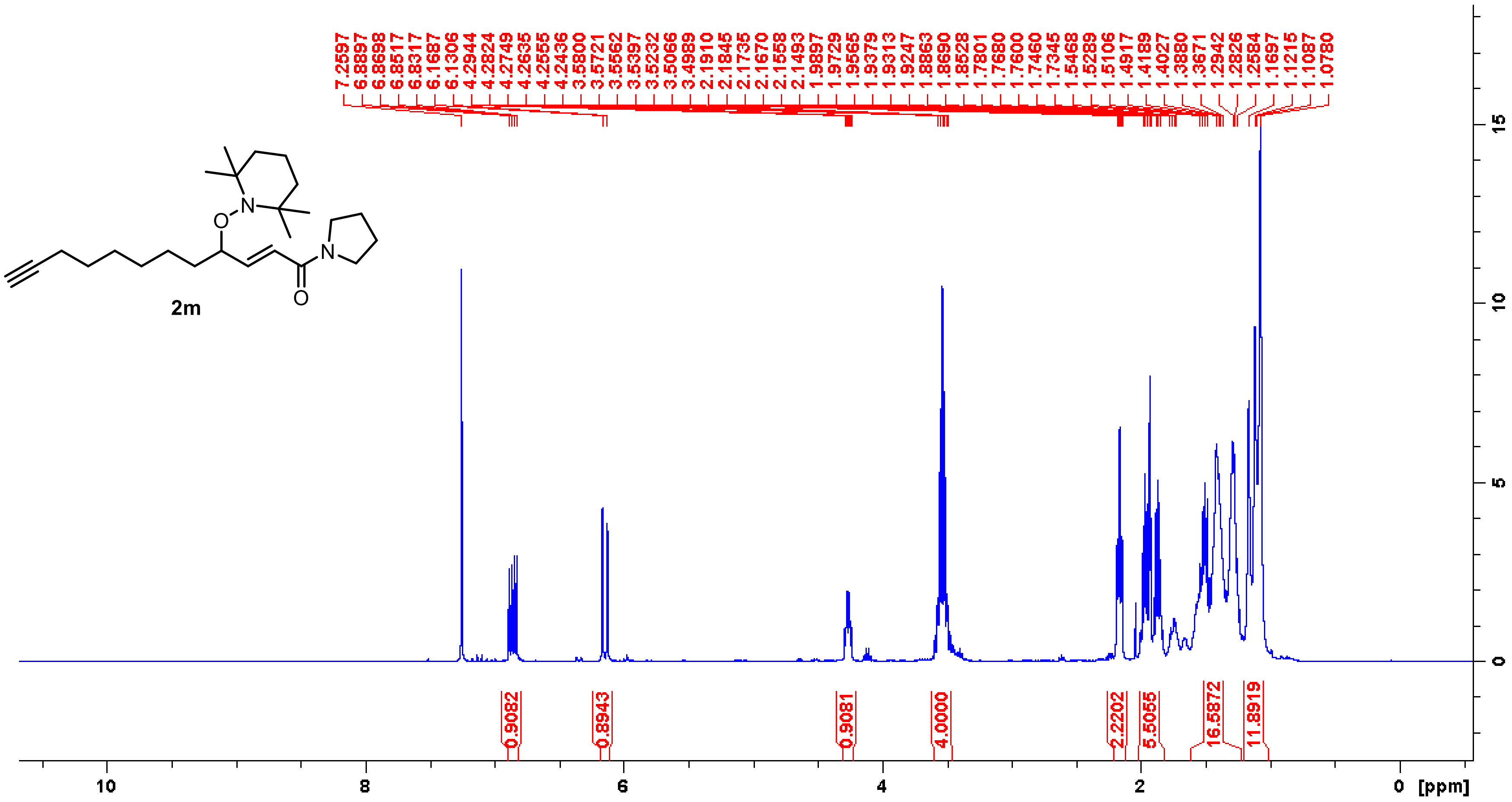

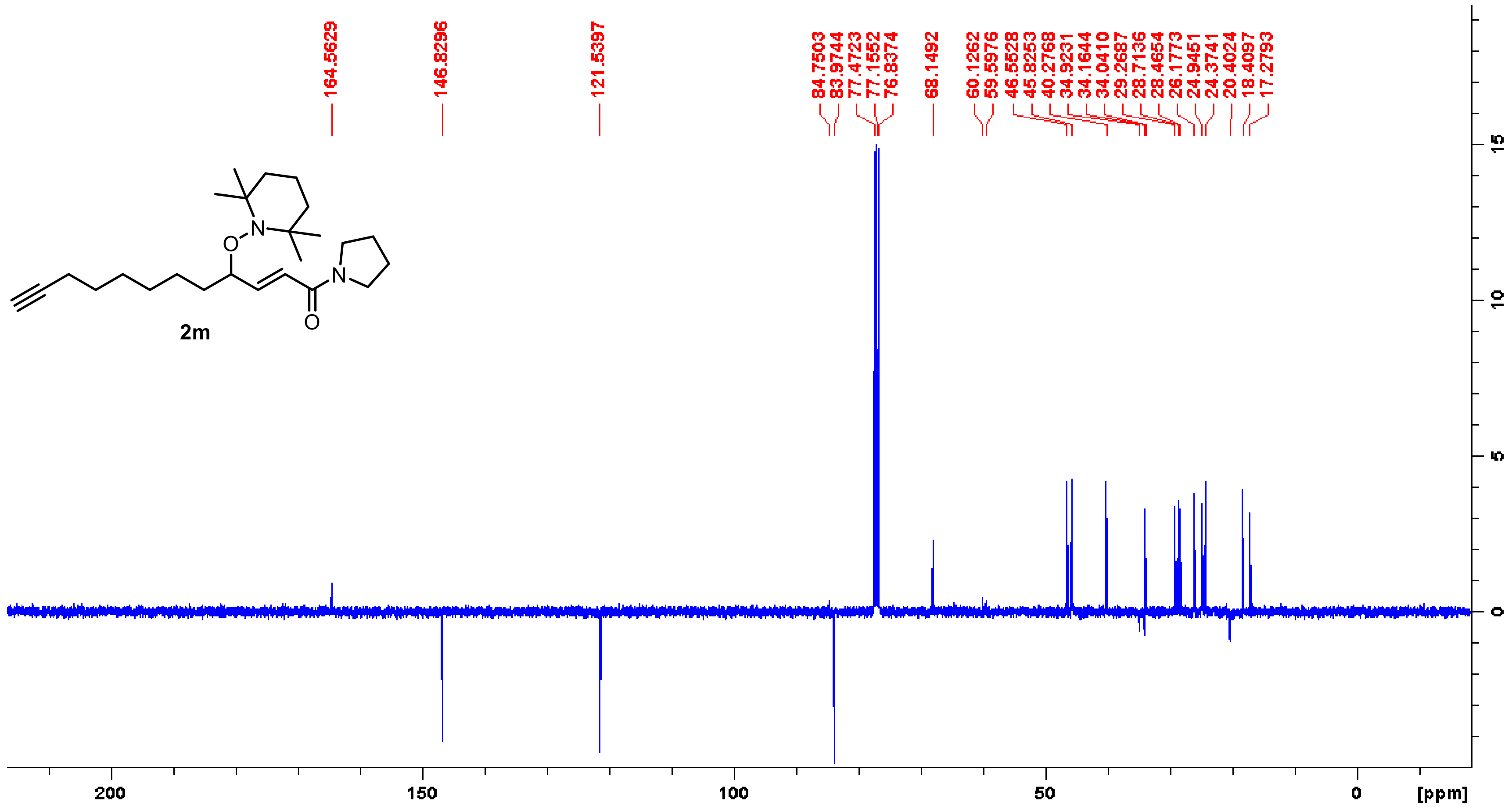

(5*R*,*E*)-5,9-Dimethyl-1-(pyrrolidin-1-yl)-4-((2,2,6,6-tetramethylpiperidin-1-yl)-oxy)-deca-2,8-dien-1-one (2n; diastereomers present)

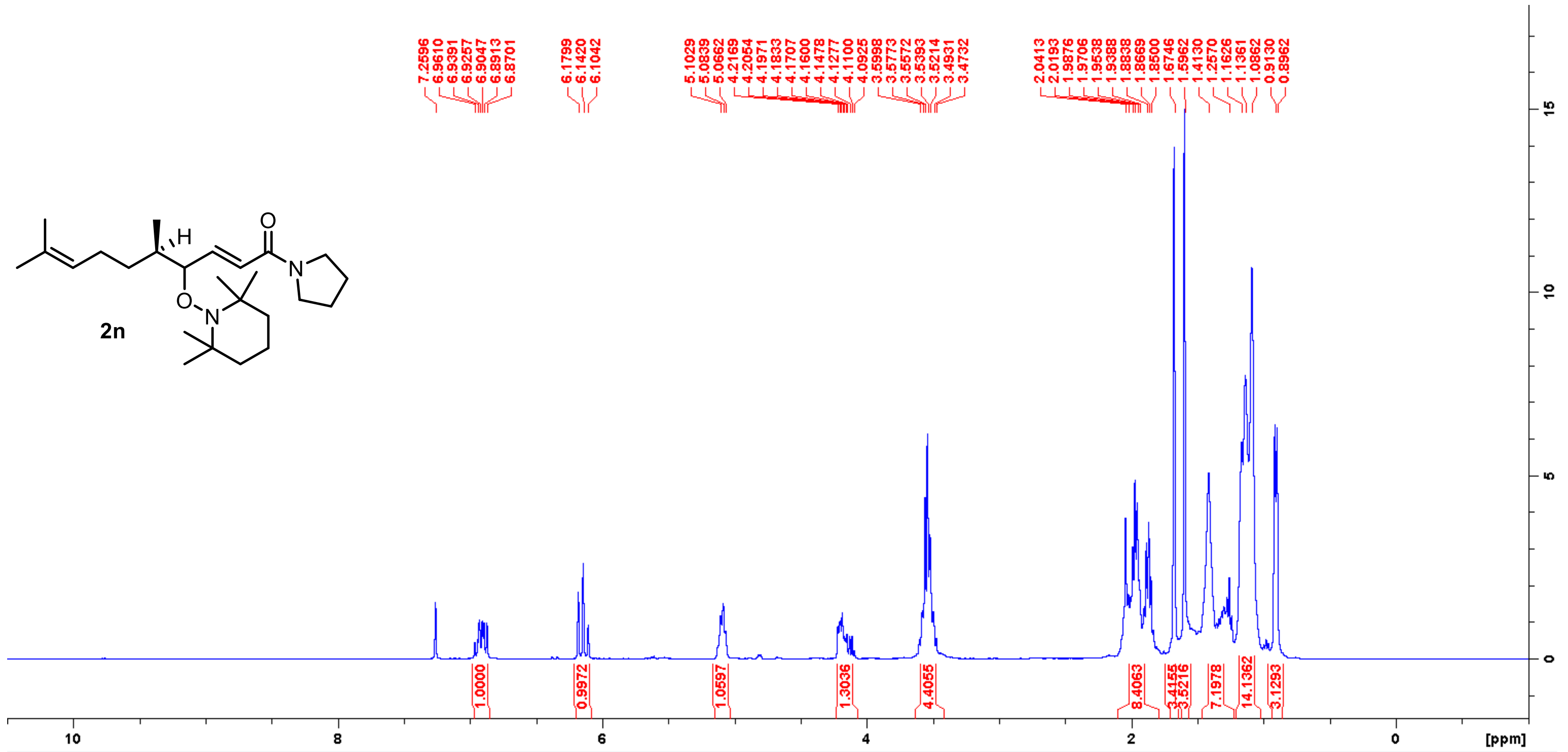

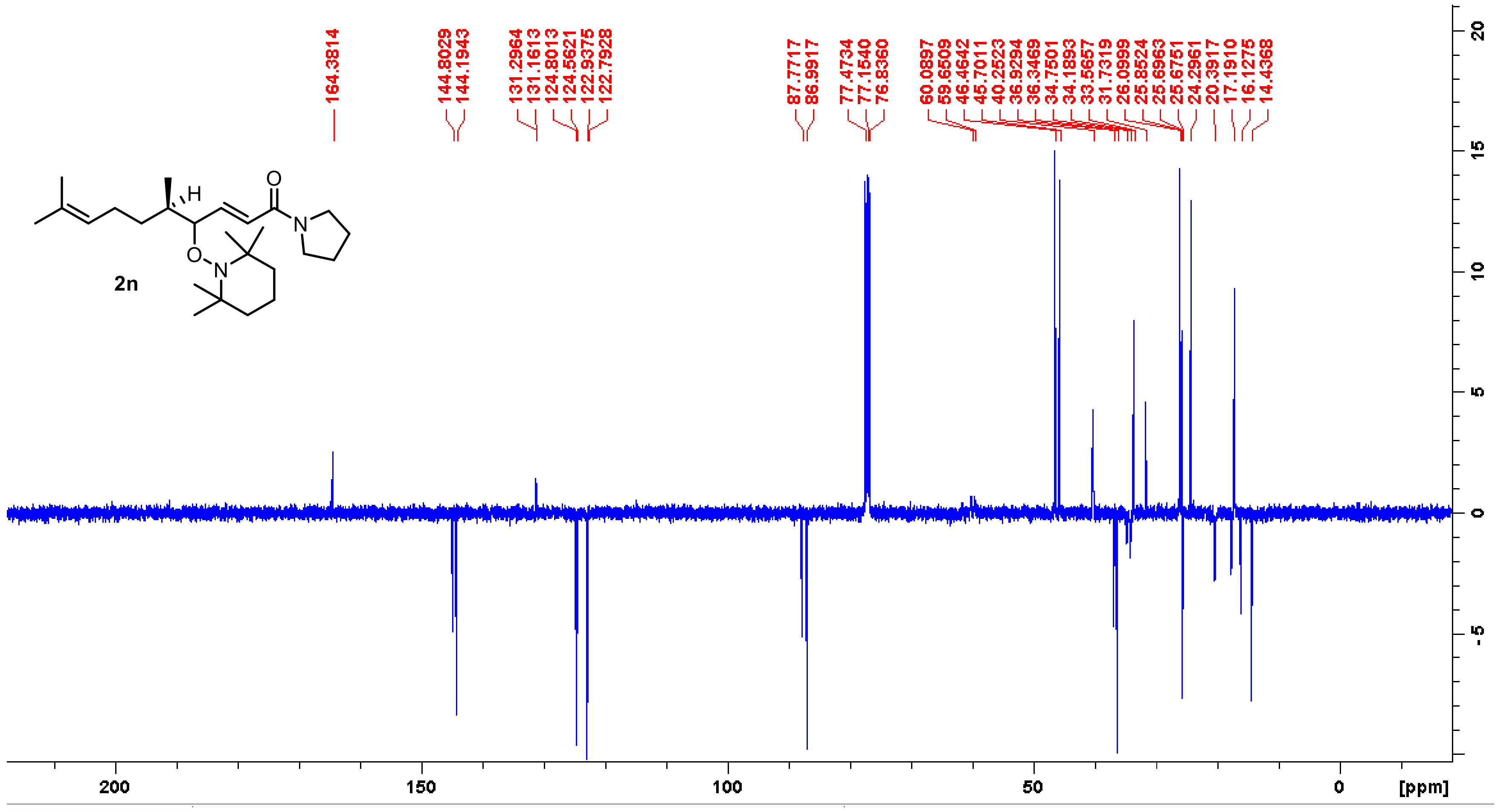

(*E*)-1-(Azepan-1-yl)-4-((2,2,6,6-tetramethylpiperidin-1-yl)-oxy)-pent-2-en-1-one (2p)

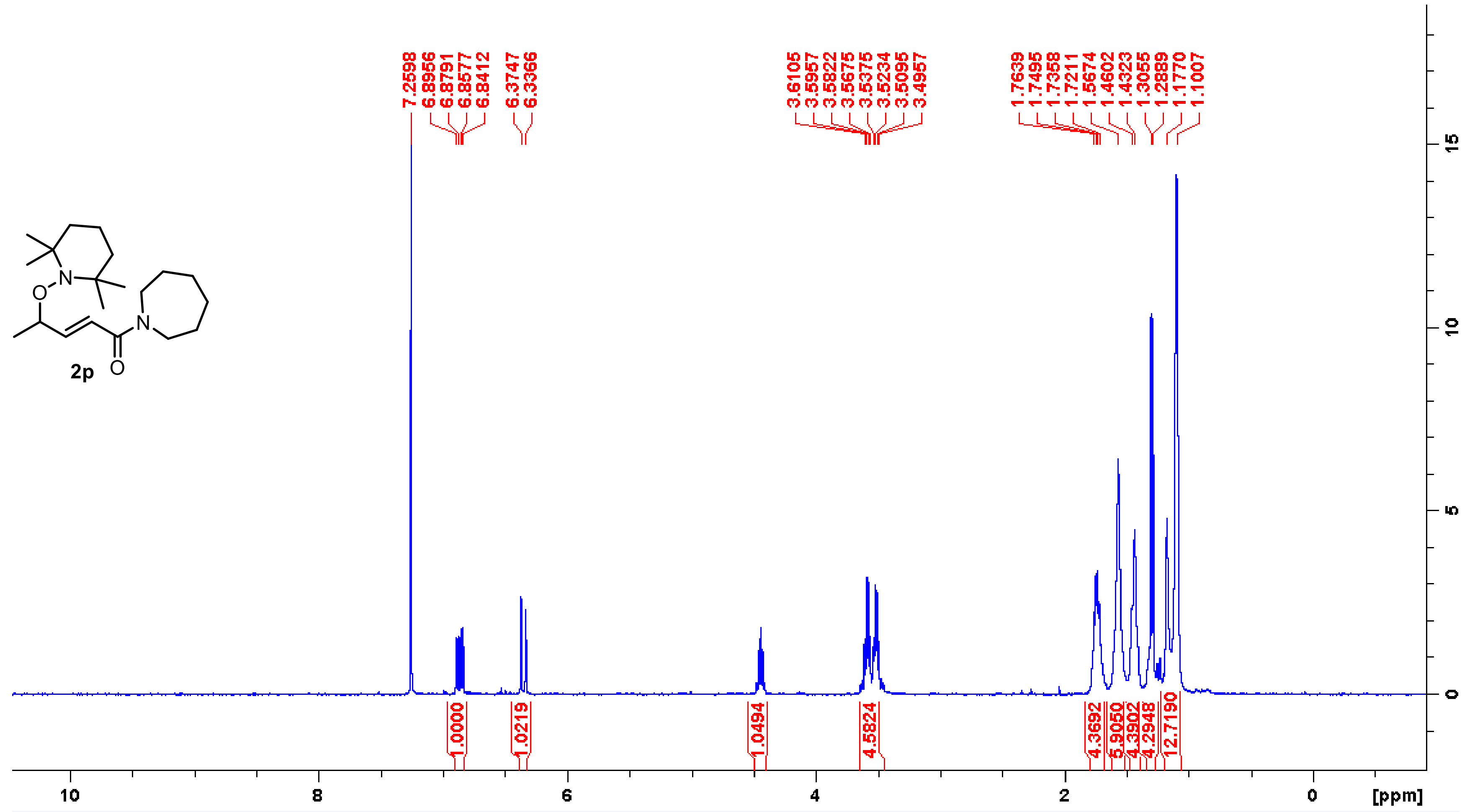

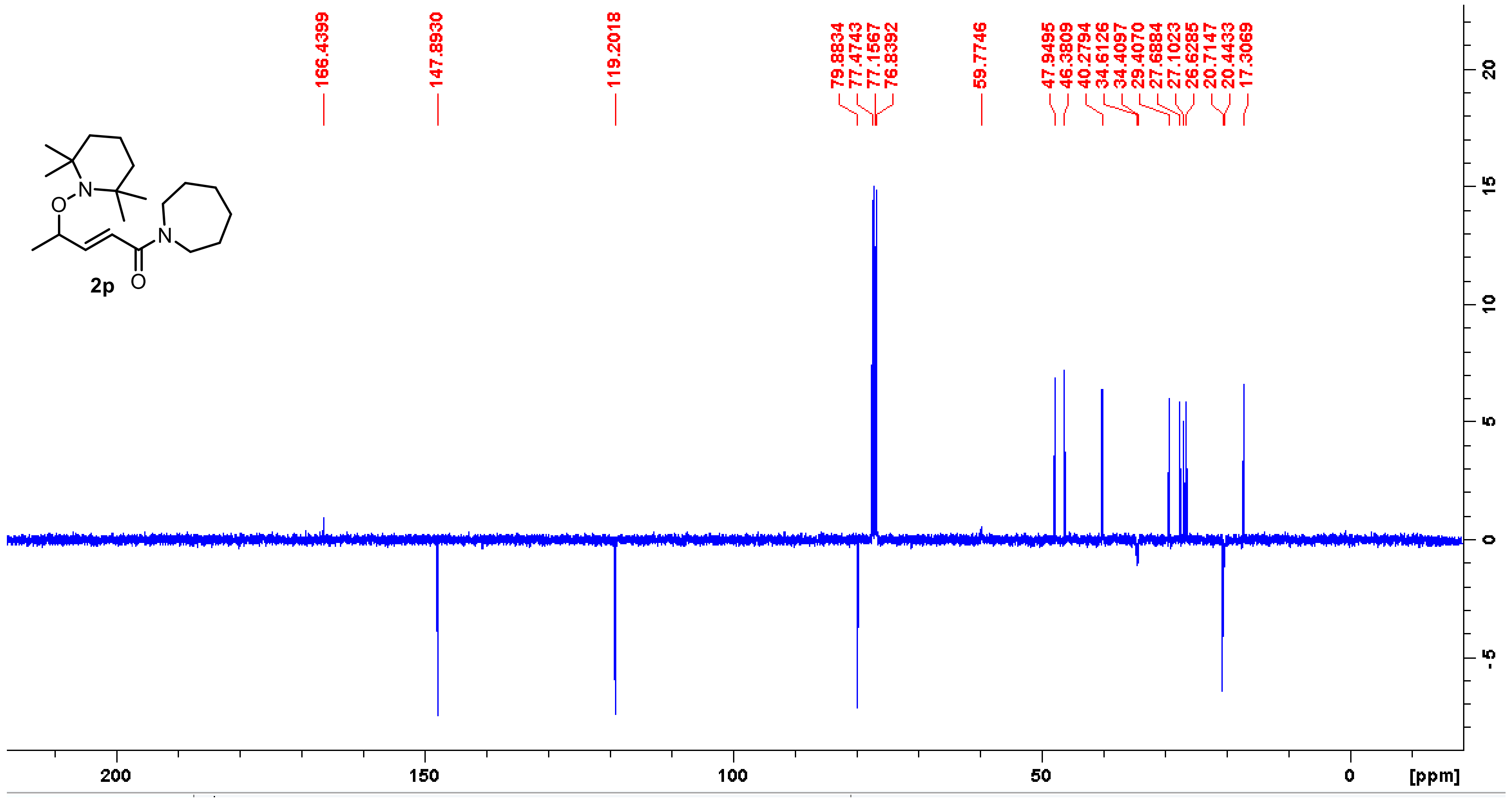

(*E*)-1-(Azetidin-1-yl)-4-((2,2,6,6-tetramethylpiperidin-1-yl)-oxy)-pent-2-en-1-one (2q)

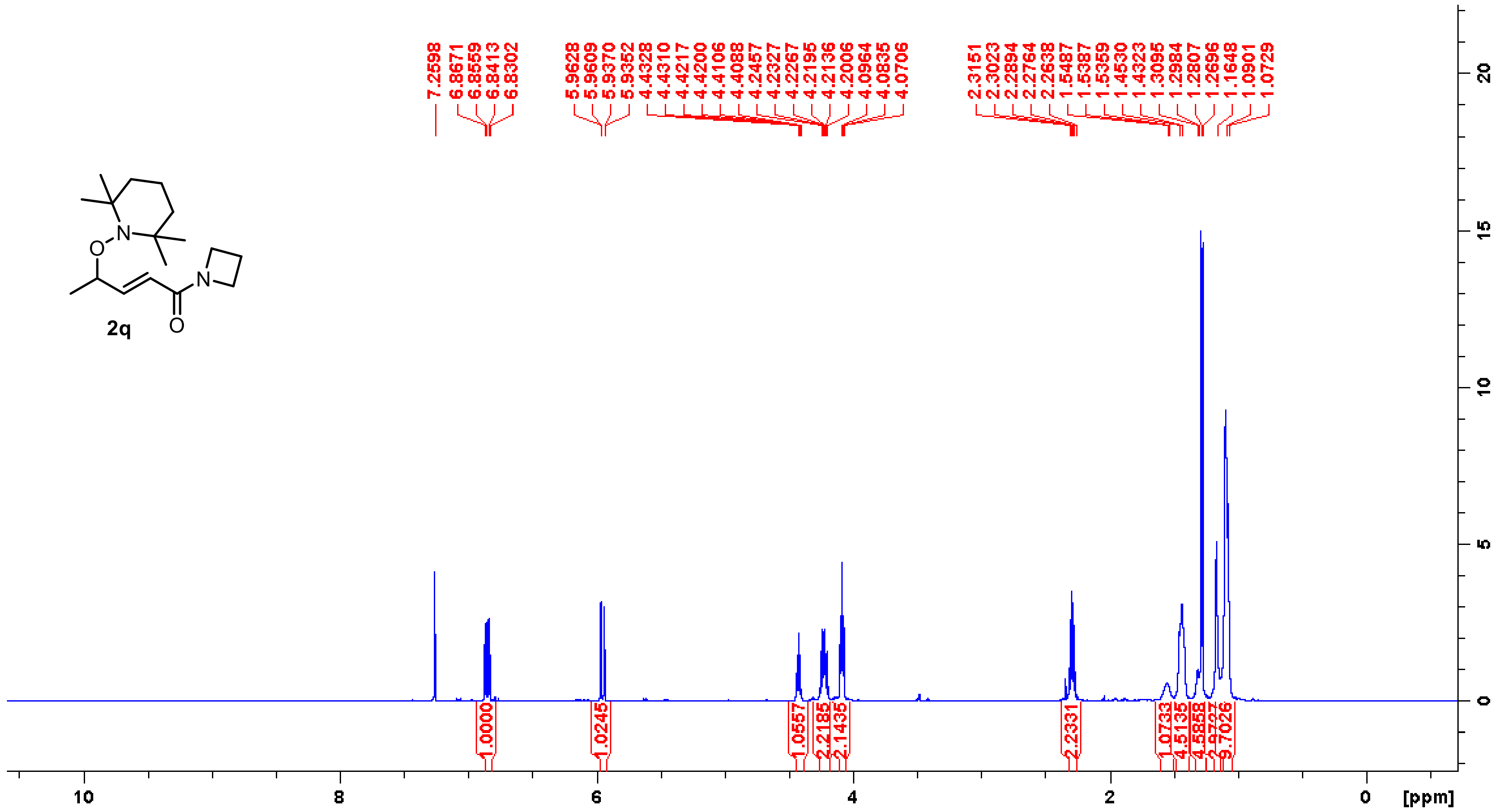

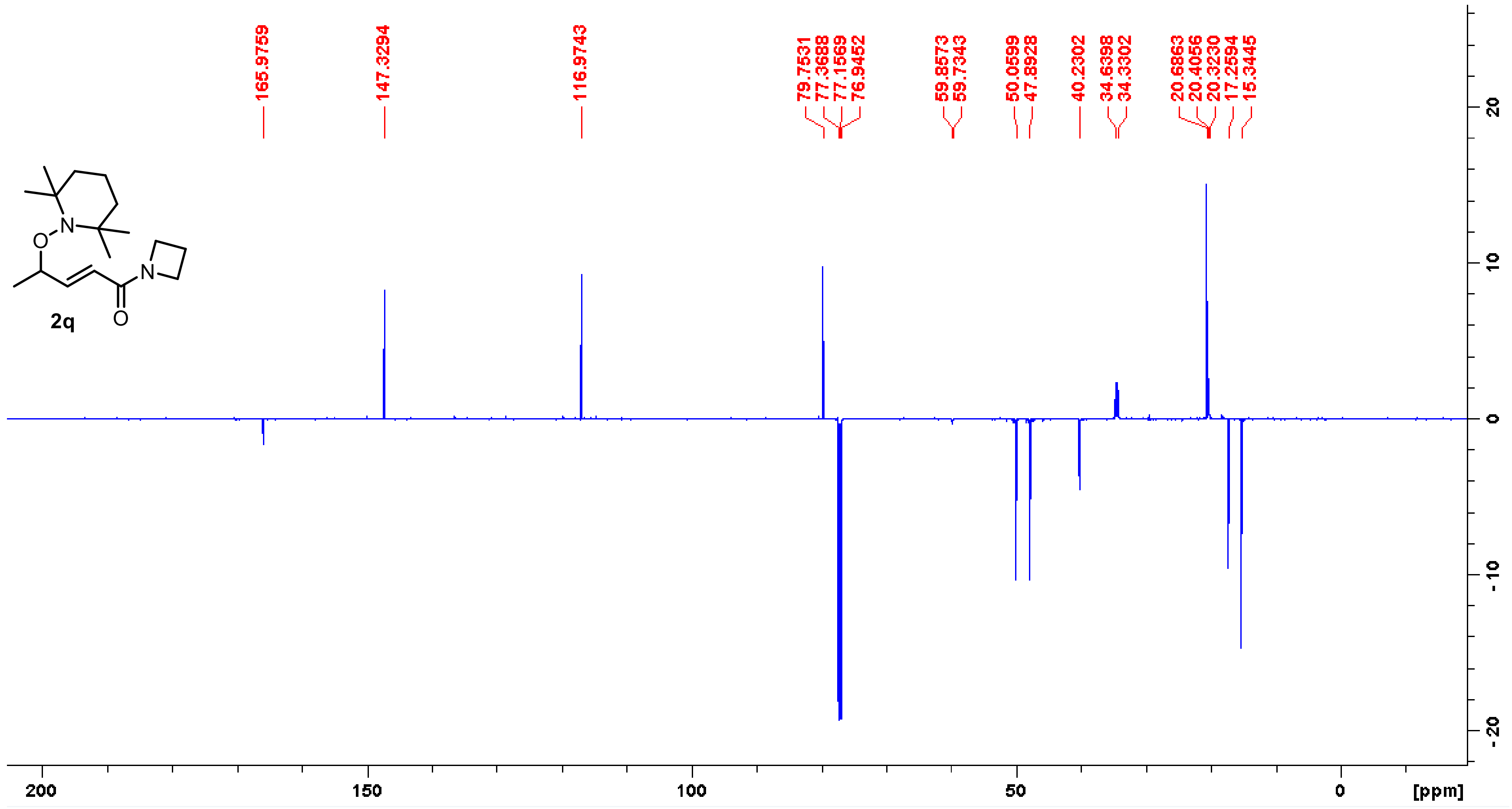

(*E*)-*N*,*N*-Dimethyl-4-((2,2,6,6-tetramethylpiperidin-1-yl)oxy)pent-2-enamide (2r)

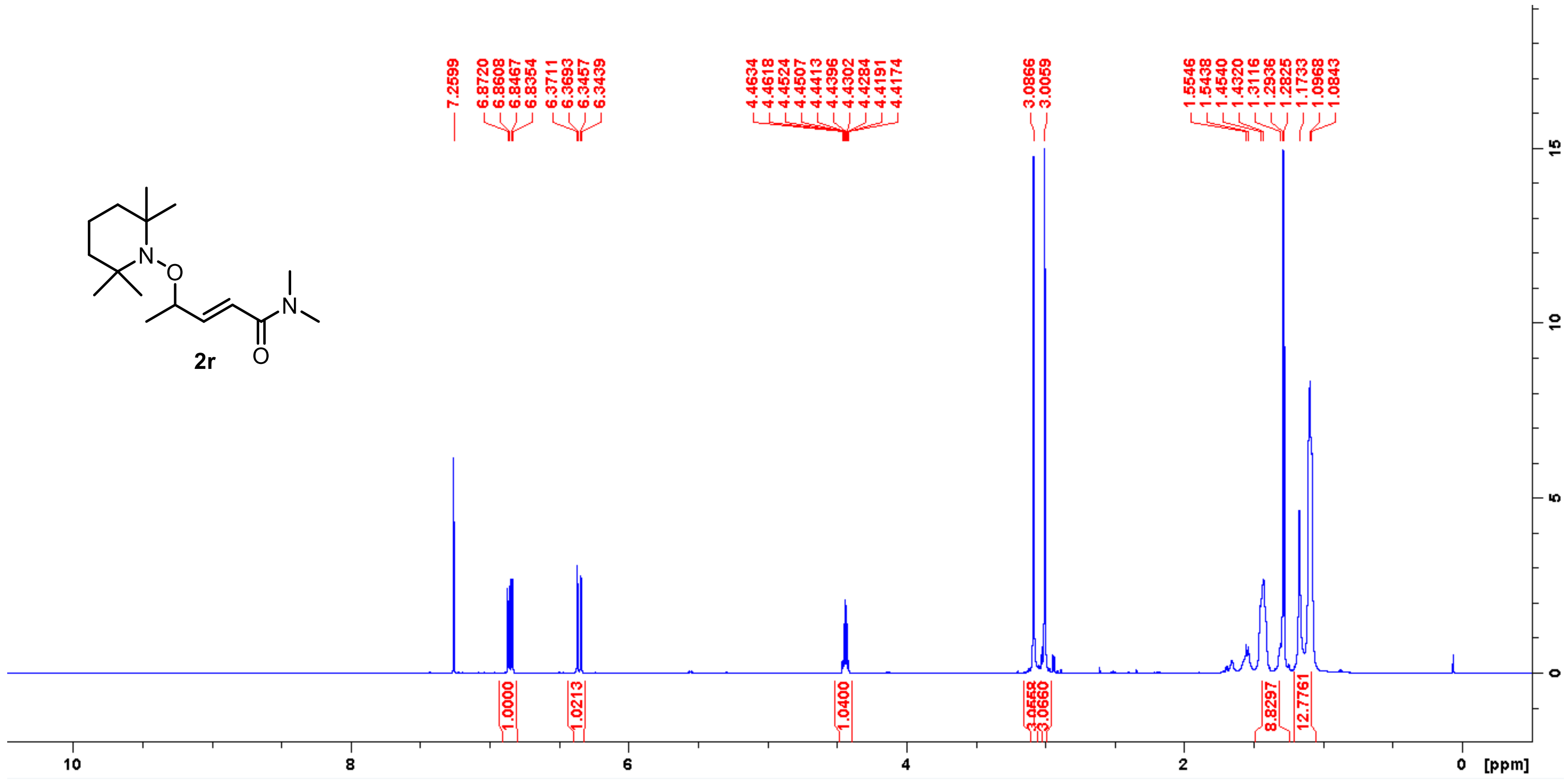

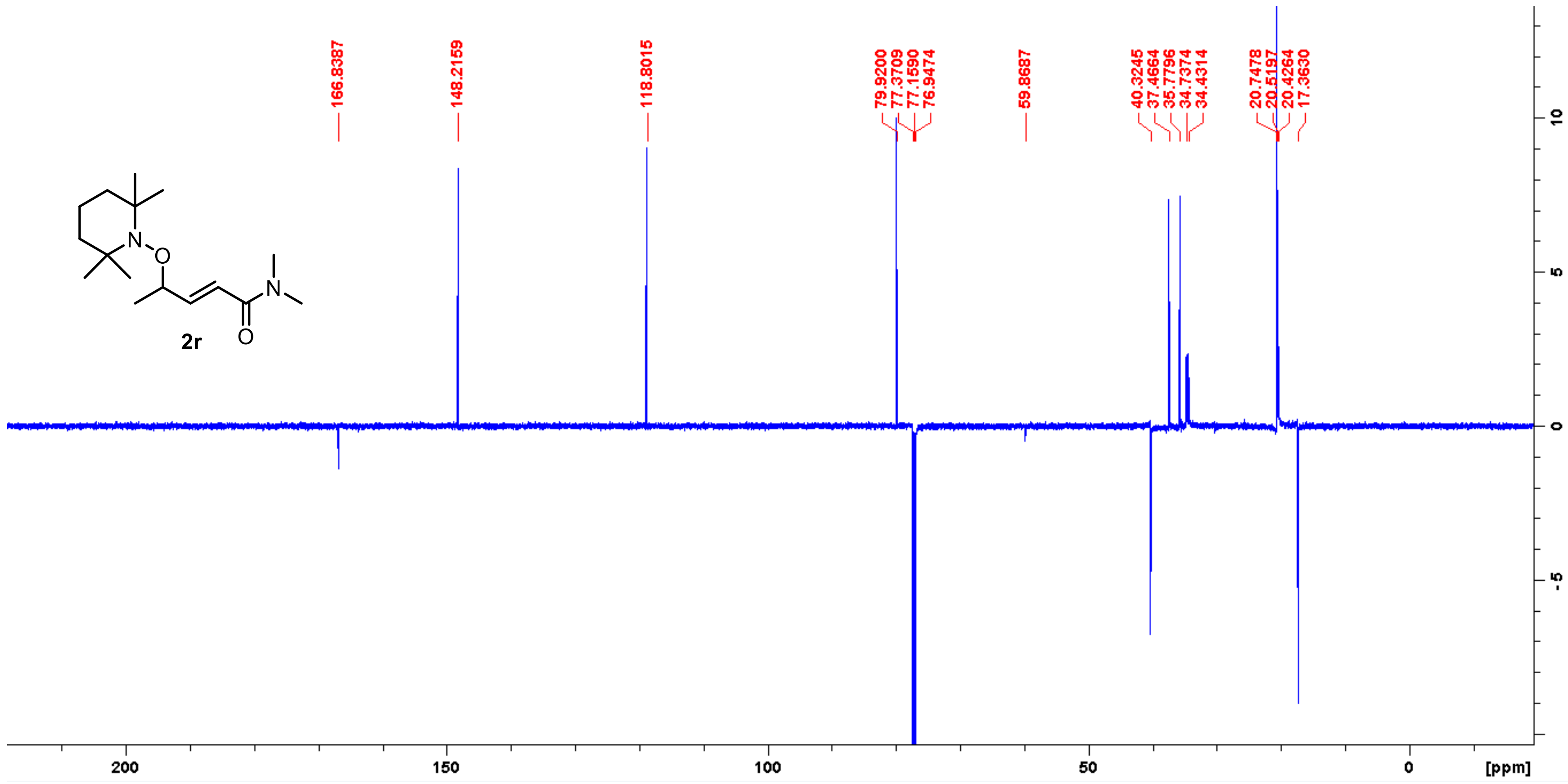

(*E*)-1-Morpholino-4-((2,2,6,6-tetramethylpiperidin-1-yl)oxy)pent-2-en-1-one (2s)

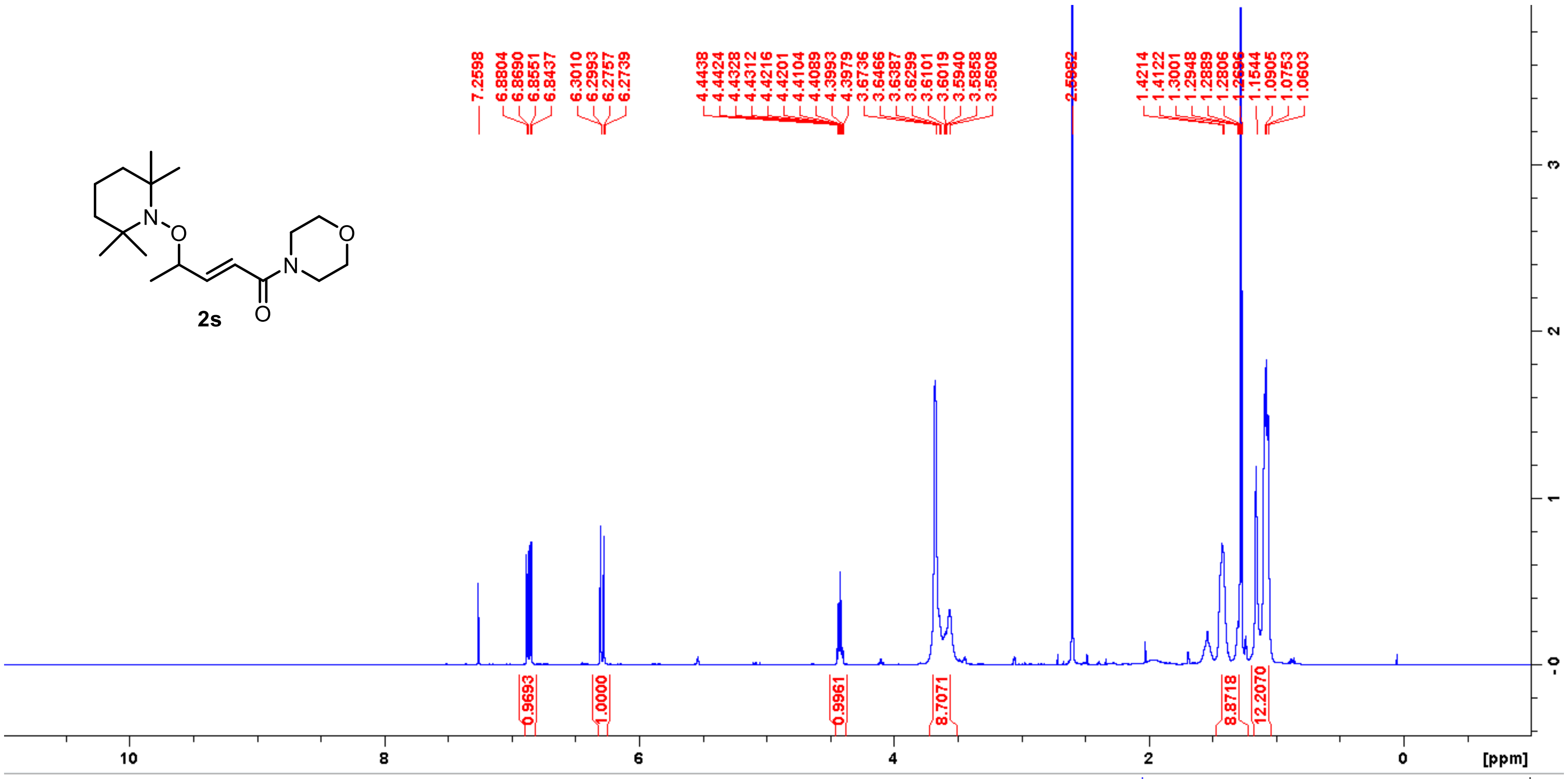

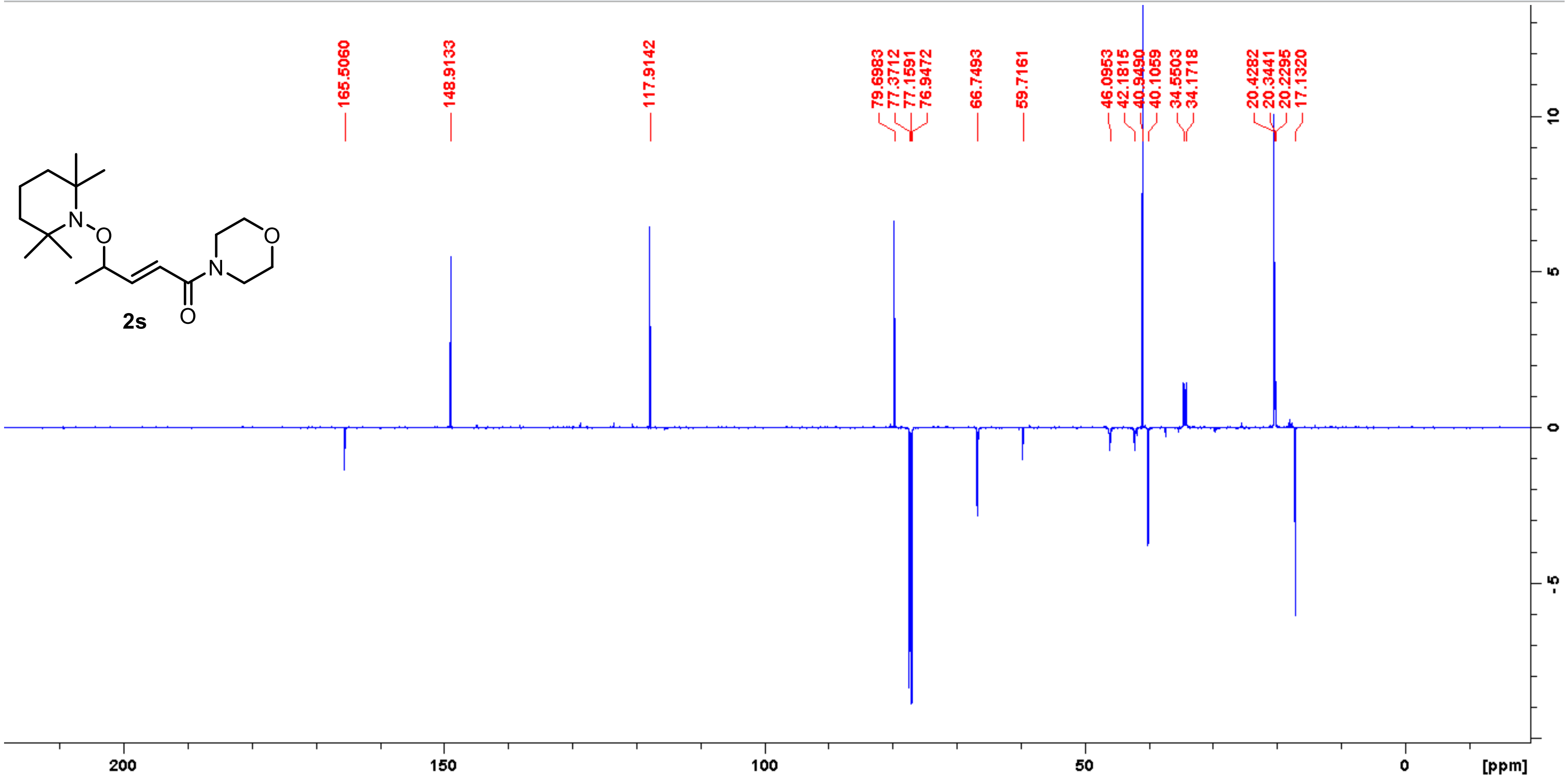

(*E*)-*N*,*N*-Dibenzyl-4-((2,2,6,6-tetramethylpiperidin-1-yl)oxy)pent-2-en-amide (2t)

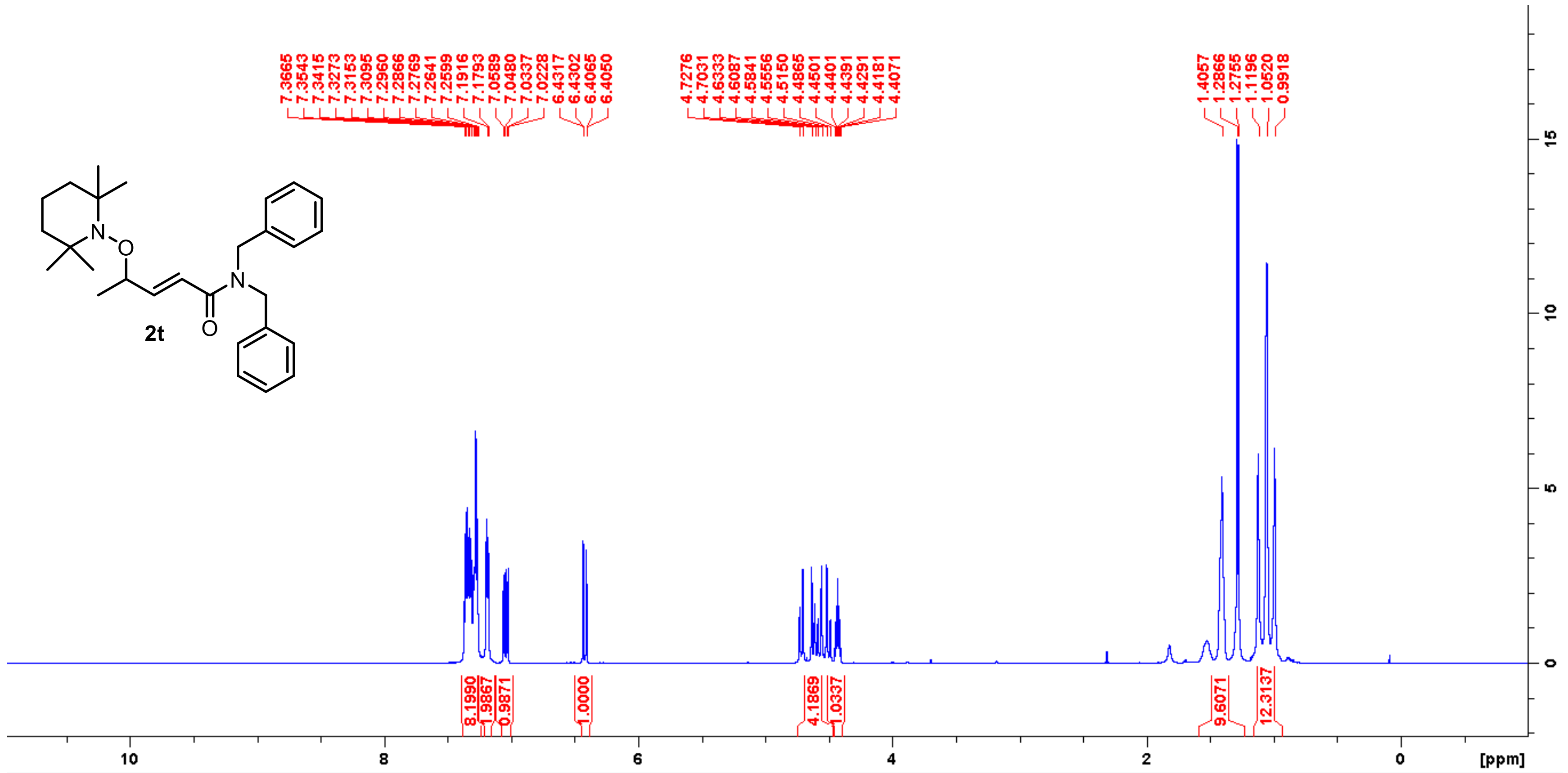

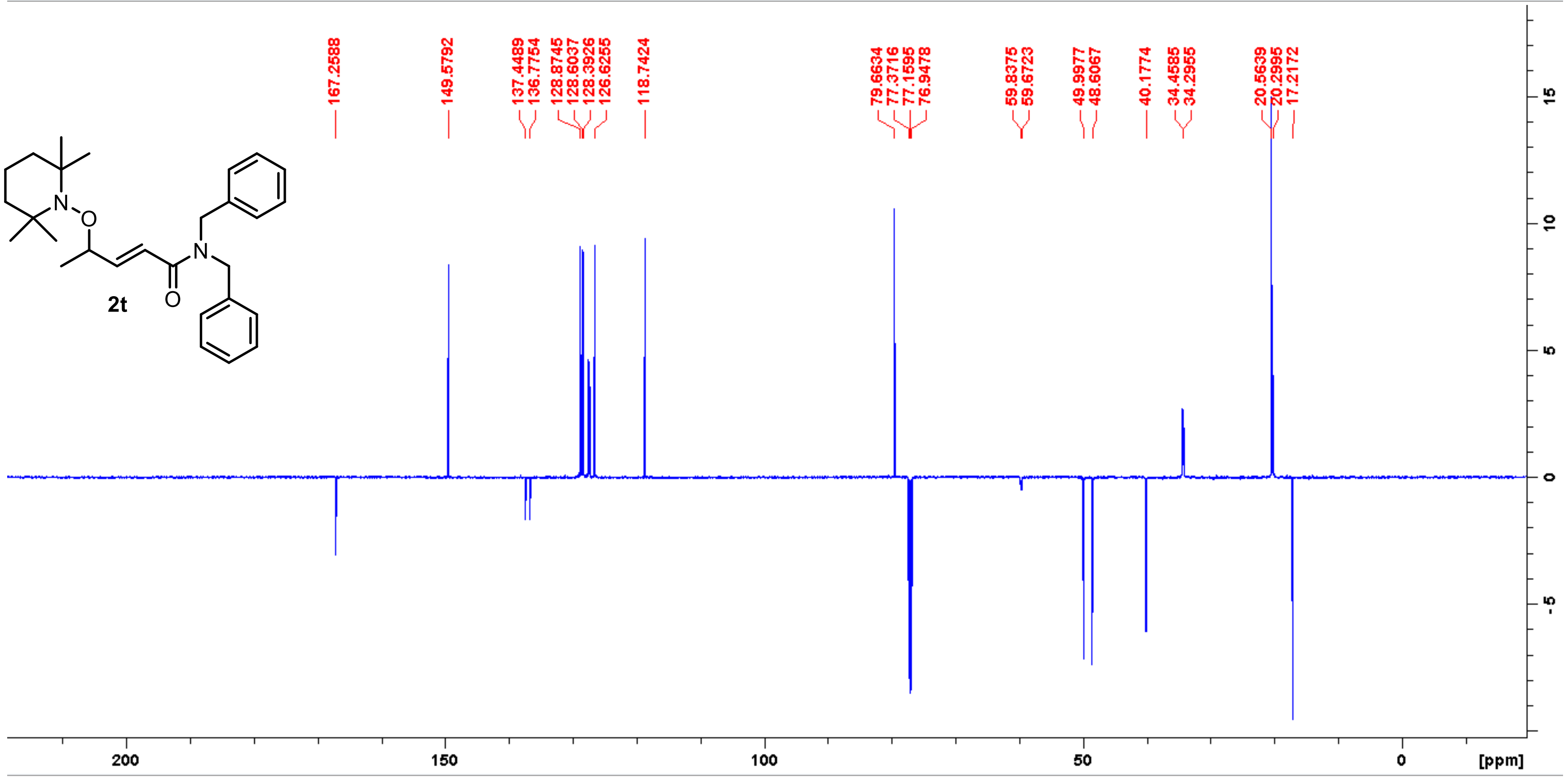

(*E*)-*N*-Butyl-*N*-(3-methylbut-2-en-1-yl)-4-((2,2,6,6-tetramethylpiperidin-1-yl)-oxy)-pent-2-enamide (2u; rotamers present)

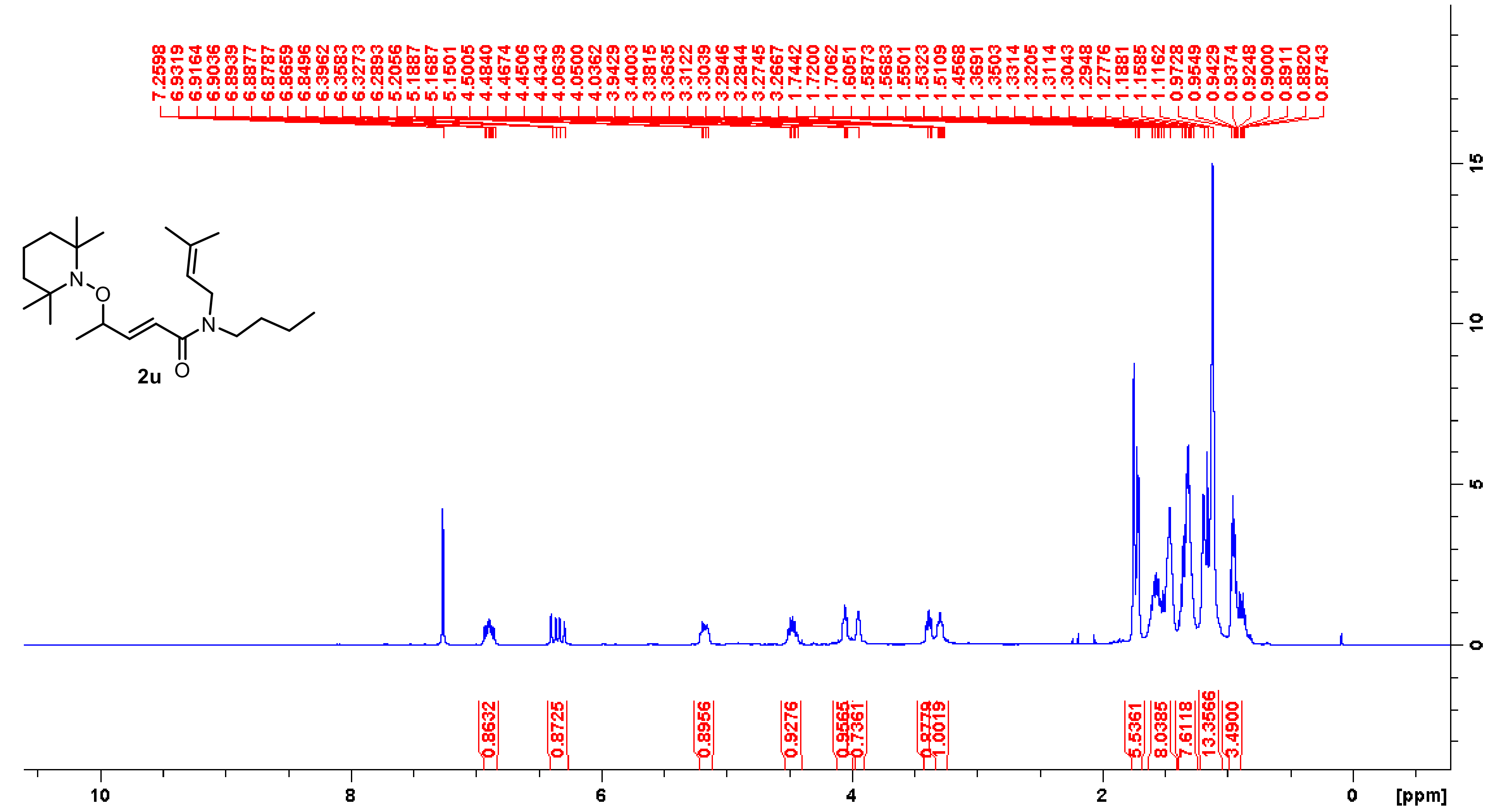

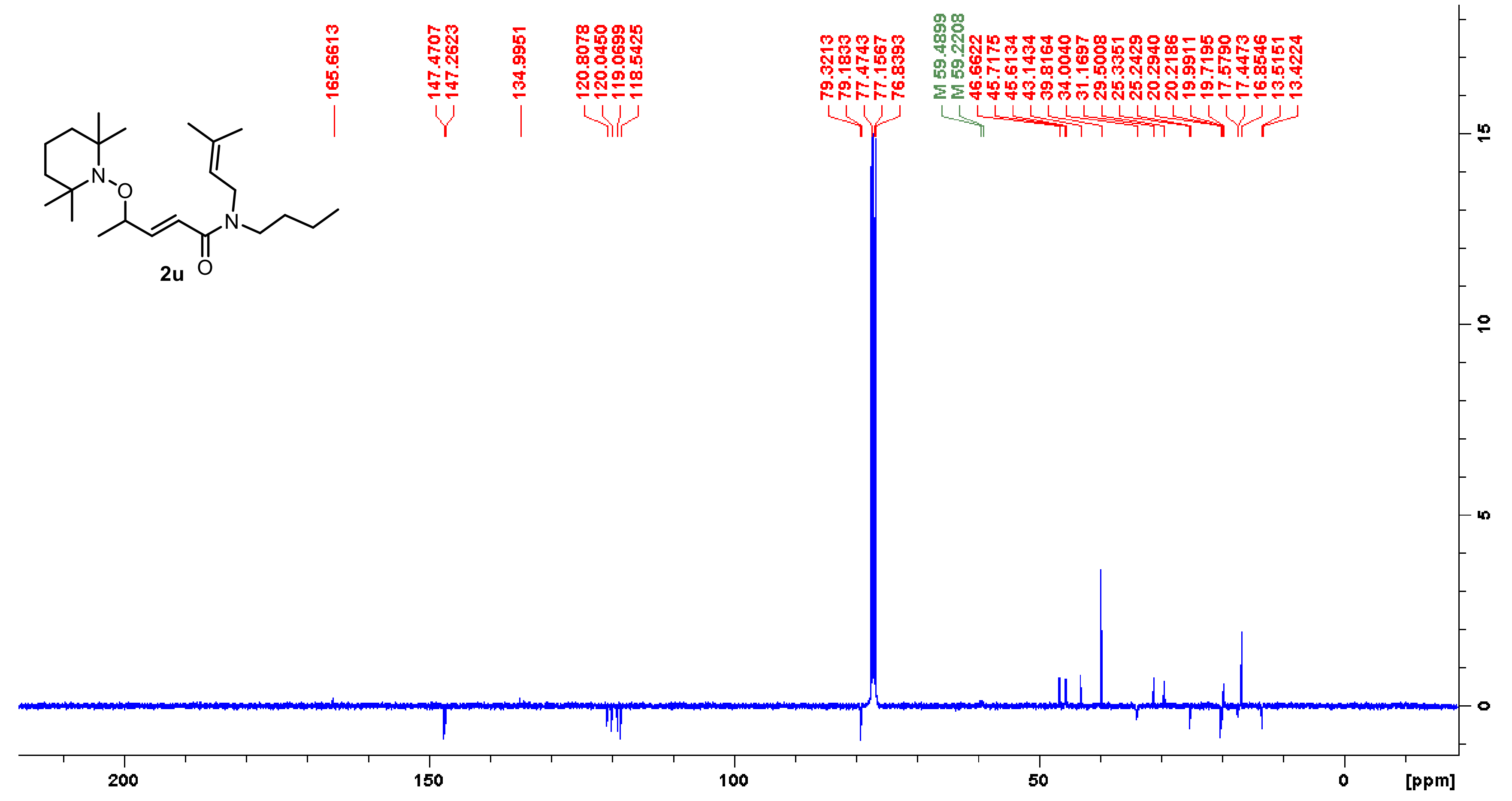

(*E*)-*N*-Allyl-*N*-methyl-4-((2,2,6,6-tetramethylpiperidin-1-yl)oxy)pent-2-en-amide (2v; rotamers present)

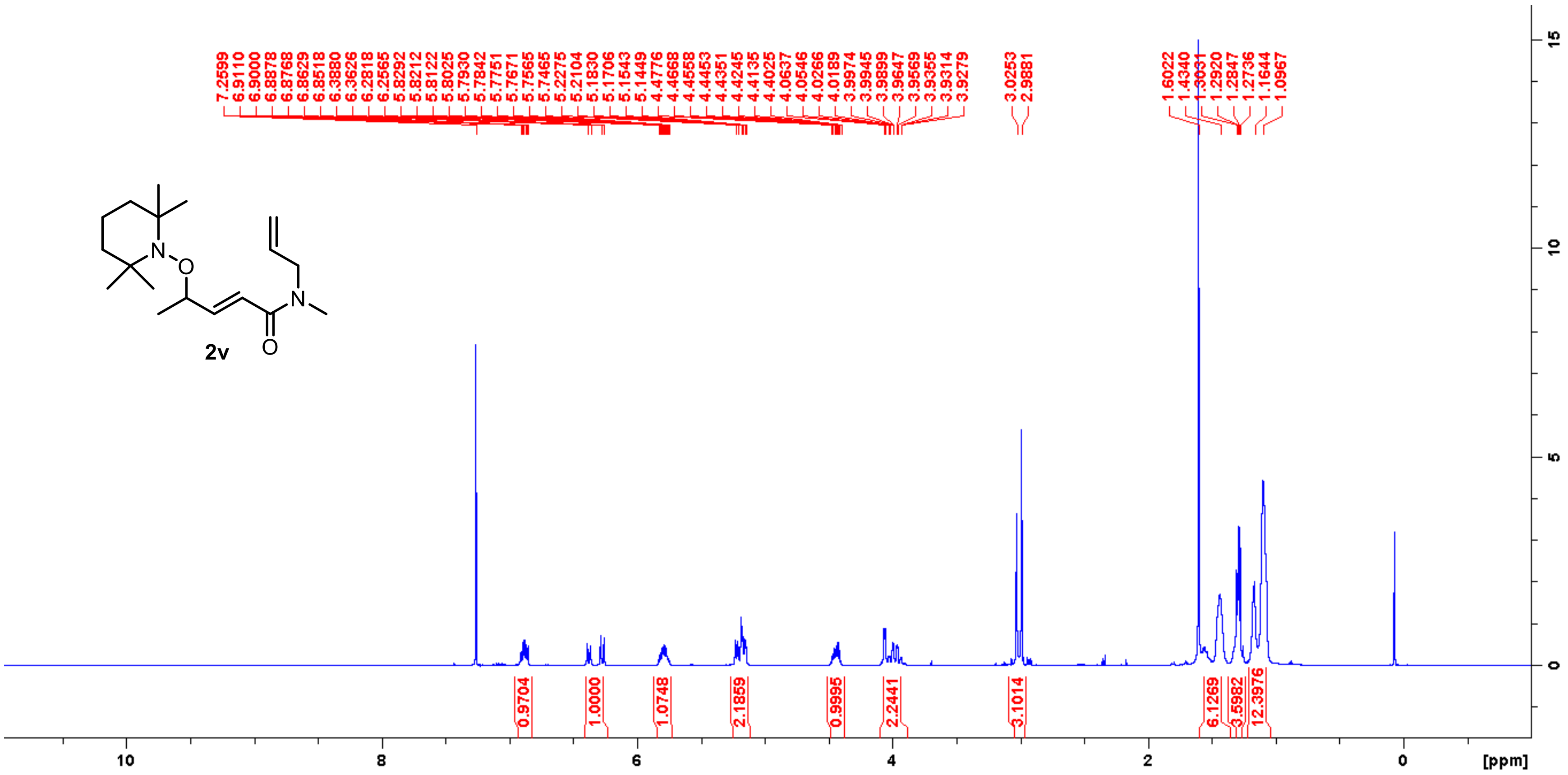

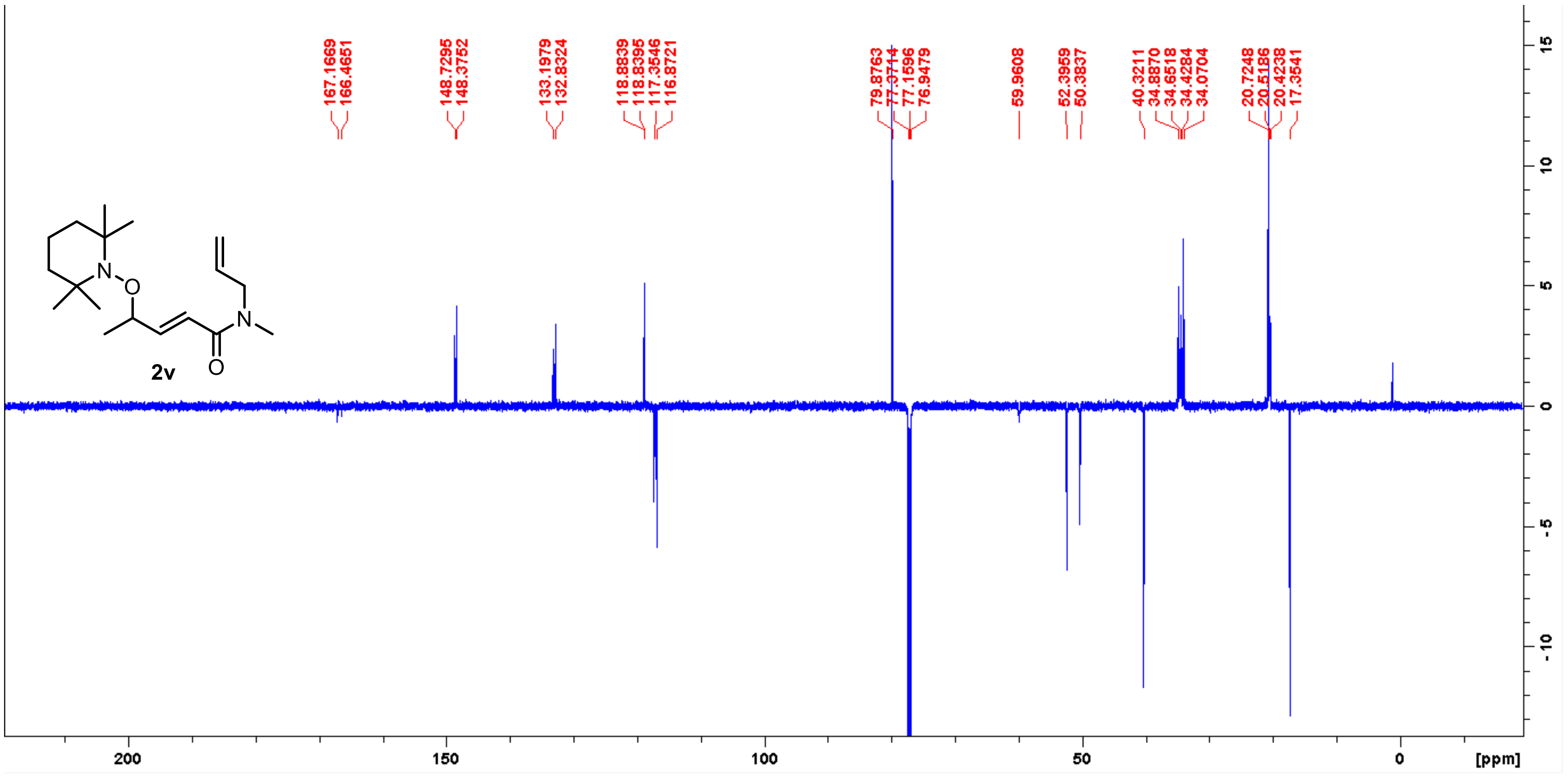

(*E*)-*N*-Cyclopropyl-*N*-methyl-4-((2,2,6,6-tetramethylpiperidin-1-yl)oxy)pent-2-enamide (2w)

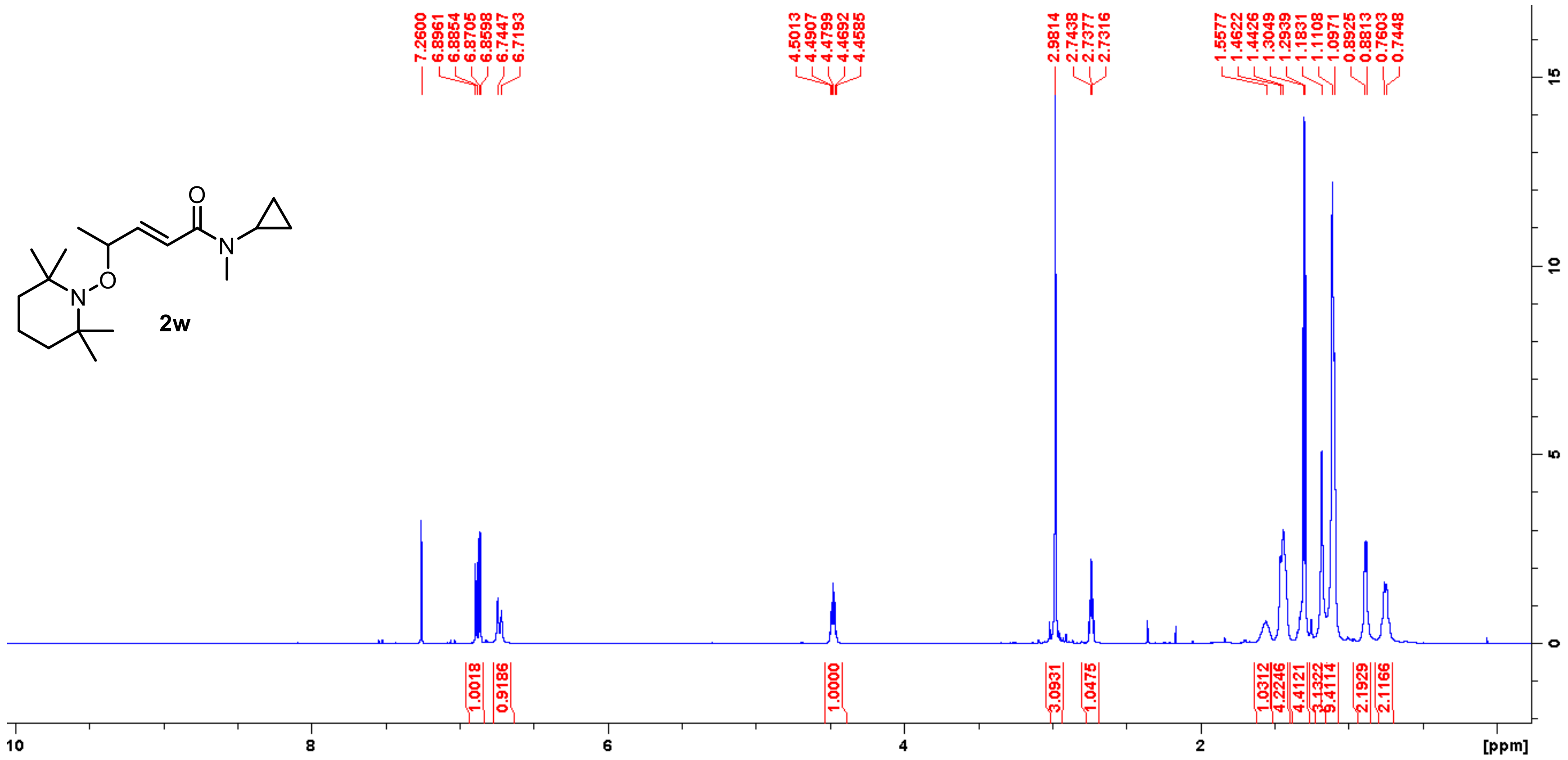

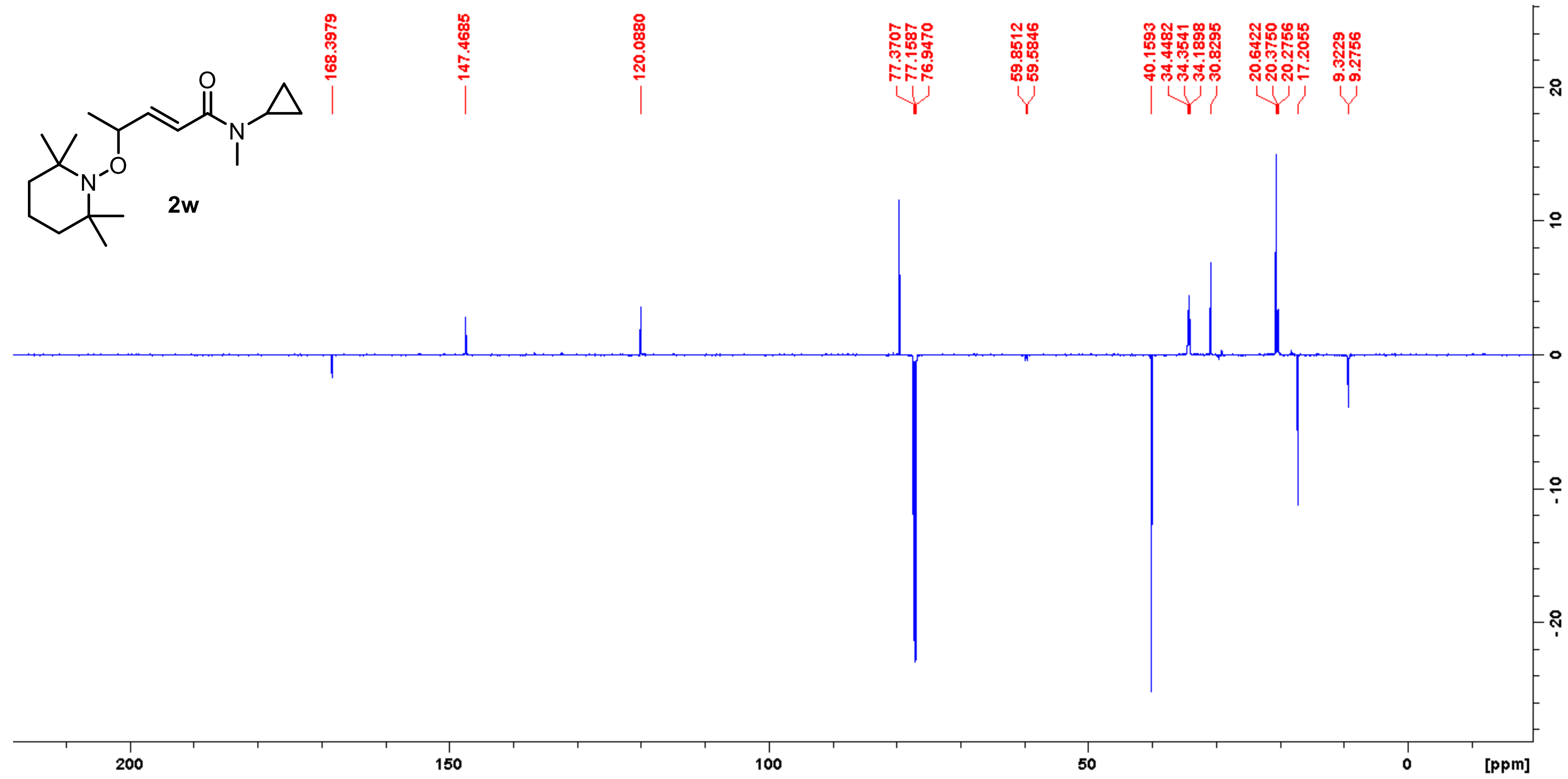

(*E*)-*N*-(Cyclopropylmethyl)-*N*-methyl-4-((2,2,6,6-tetramethylpiperidin-1-yl)oxy)pent-2-enamide (2x; rotamers present)

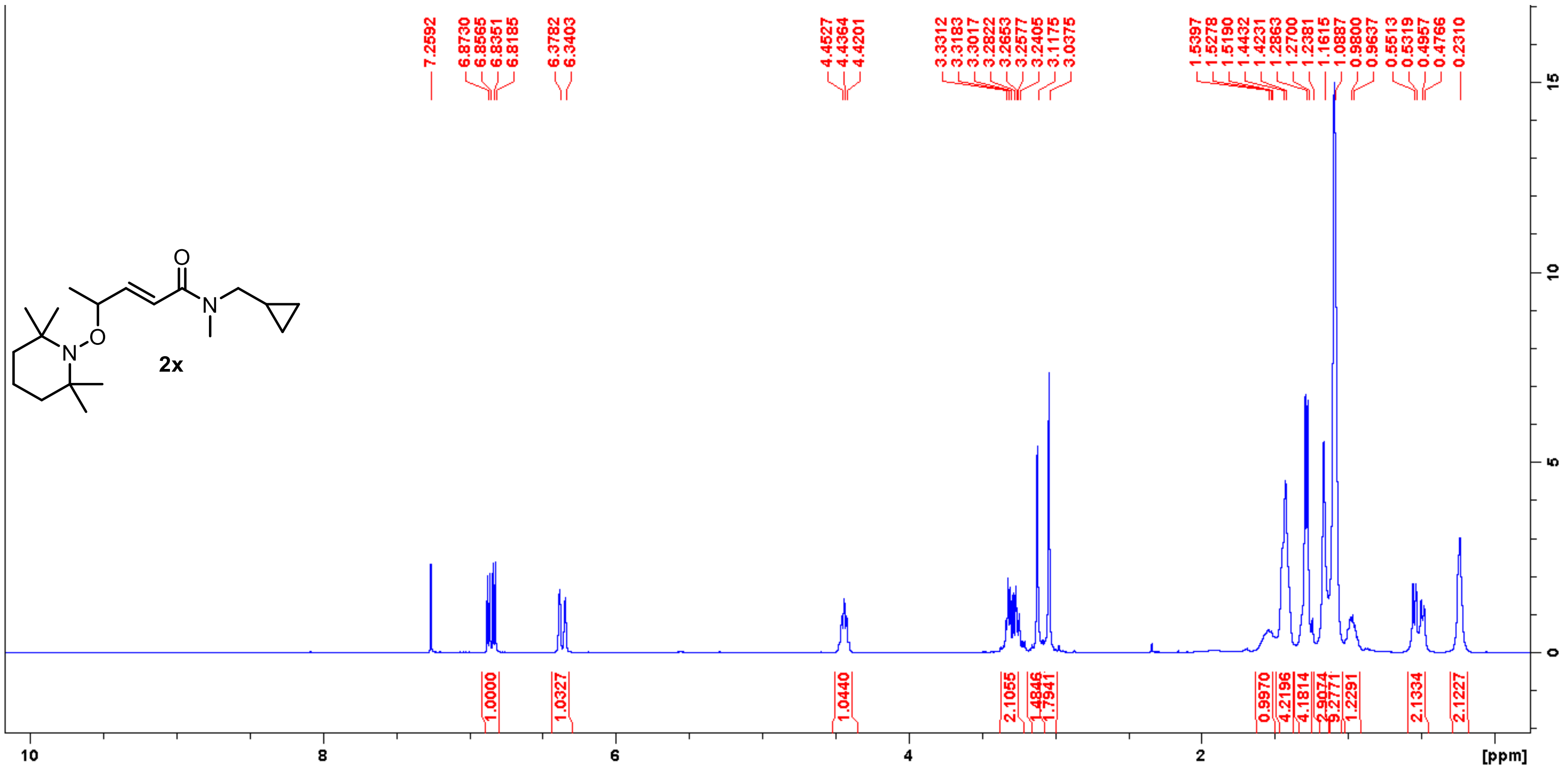

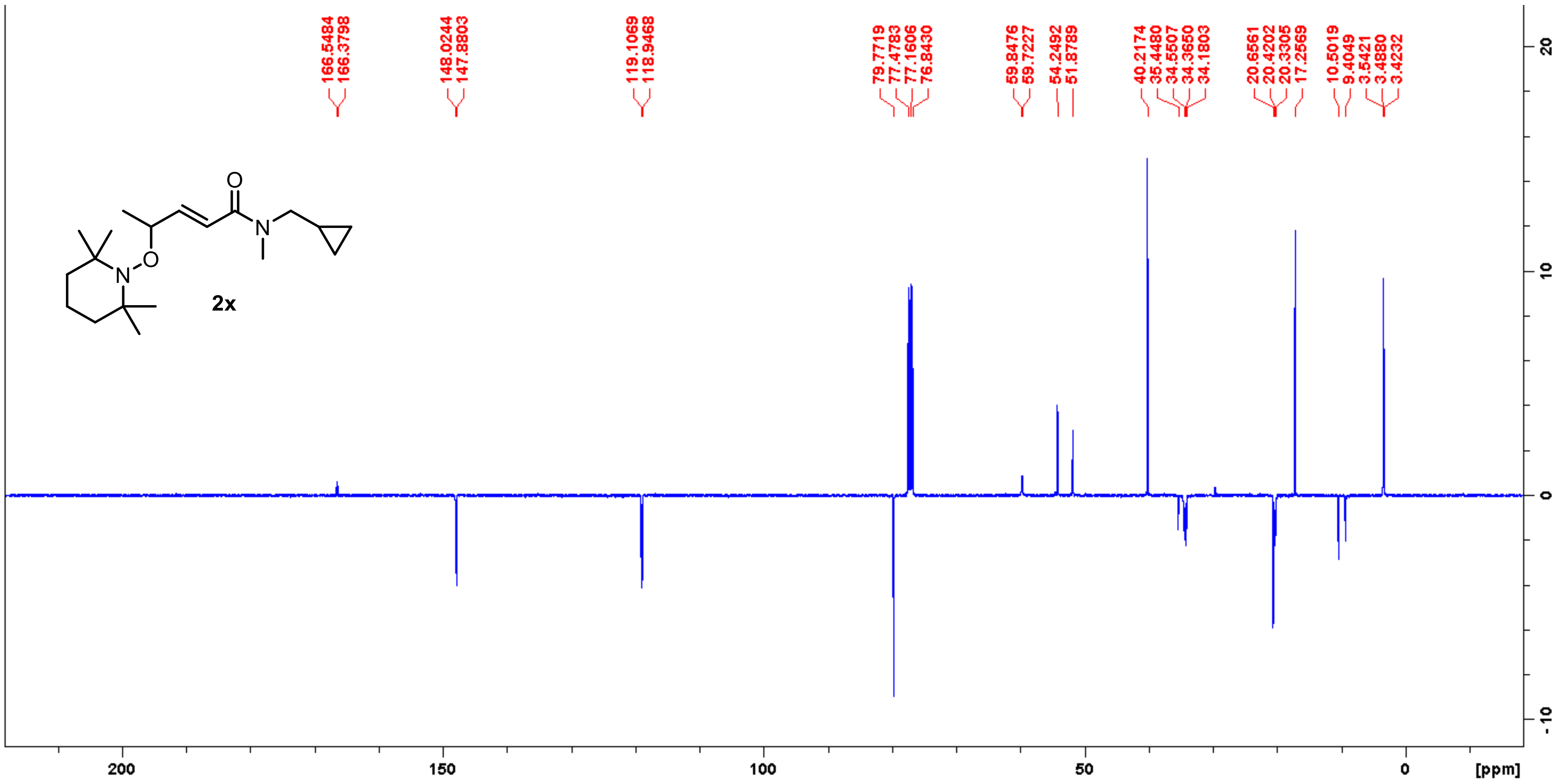

(*E*)-1-(2-Phenylpyrrolidin-1-yl)-4-((2,2,6,6-tetramethylpiperidin-1-yl)-oxy)-dodec-2-en-1-one (2y; rotamers and diastereomers present)

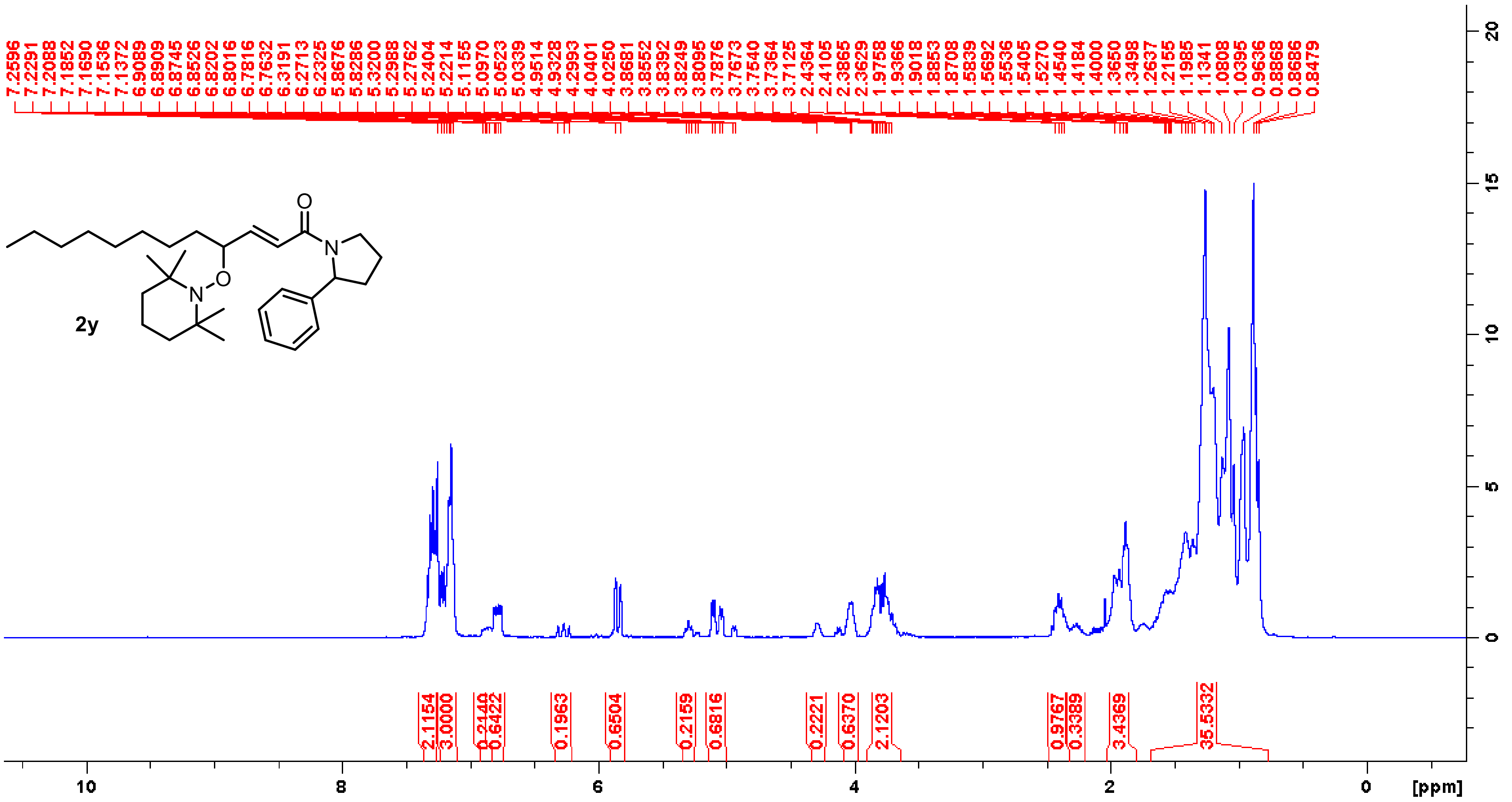

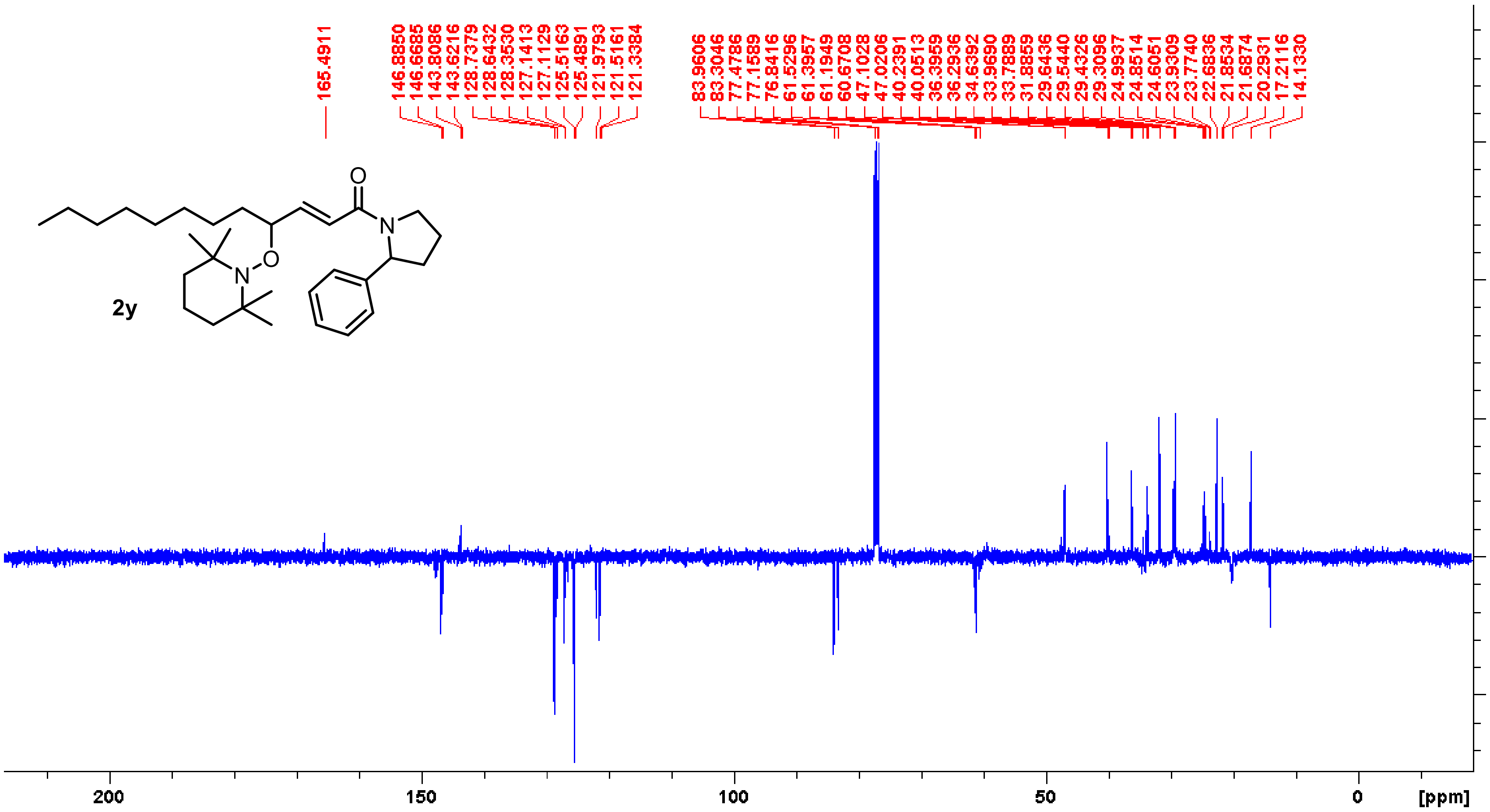

(5*R*,*E*)-*N*-Butyl-5,9-dimethyl-N-(3-methylbut-2-en-1-yl)-4-((2,2,6,6-tetramethylpiperidin-1-yl)-oxy)-deca-2,8-dienamide (2z; rotamers and diastereomers present)

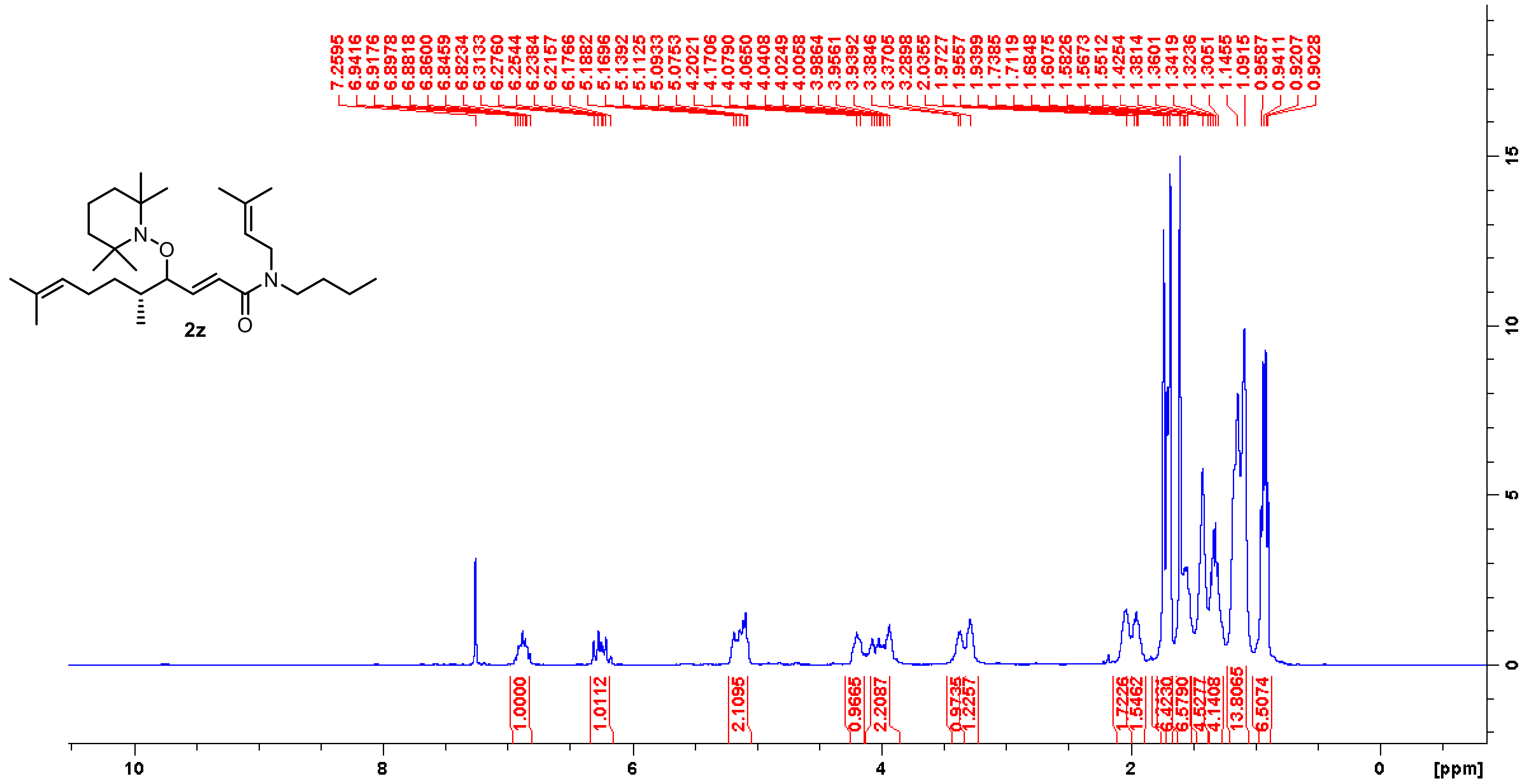

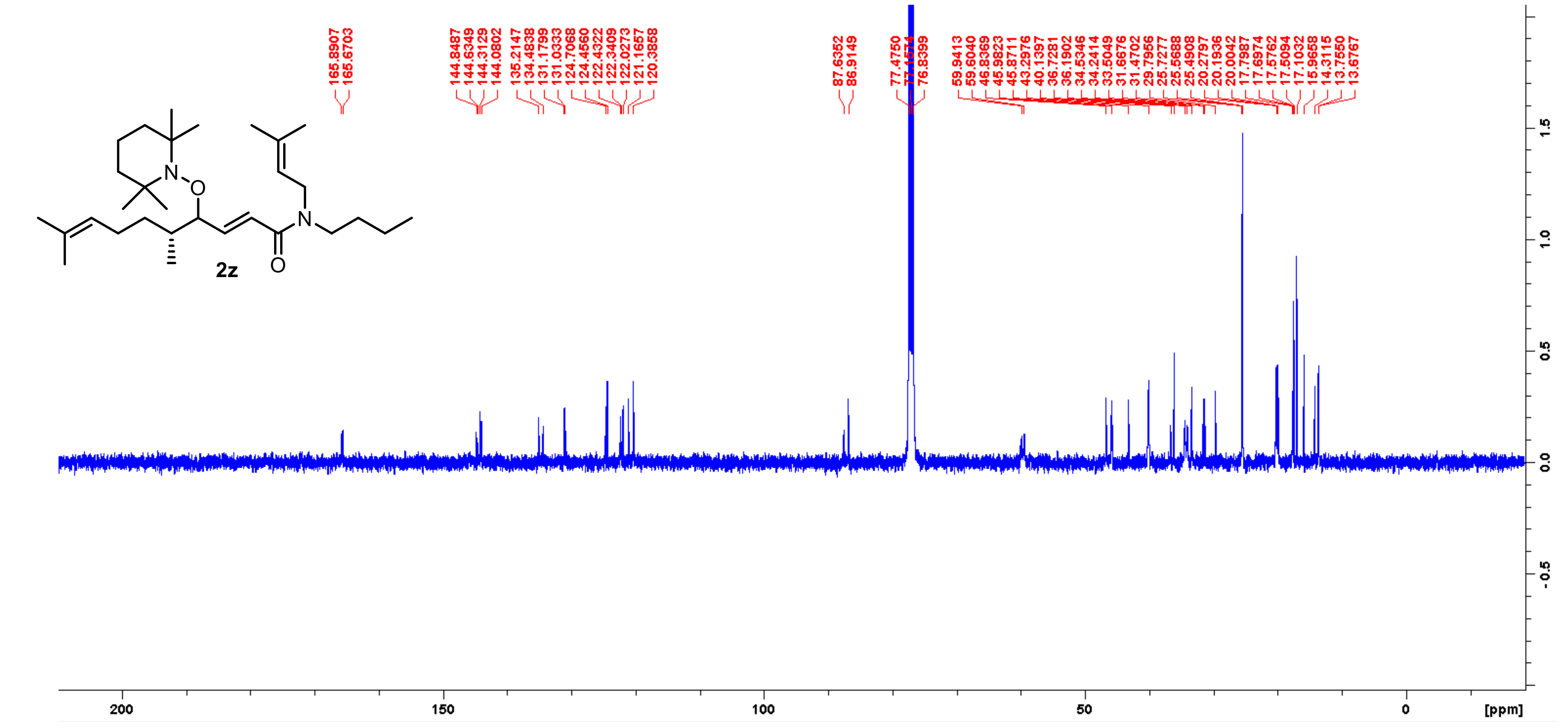

(*E*)-5-(benzo[d][1,3]dioxol-5-yl)-1-(piperidin-1-yl)-4-((2,2,6,6-tetramethylpiperidin-1-yl)oxy)pent-2-en-1-one (2aa)

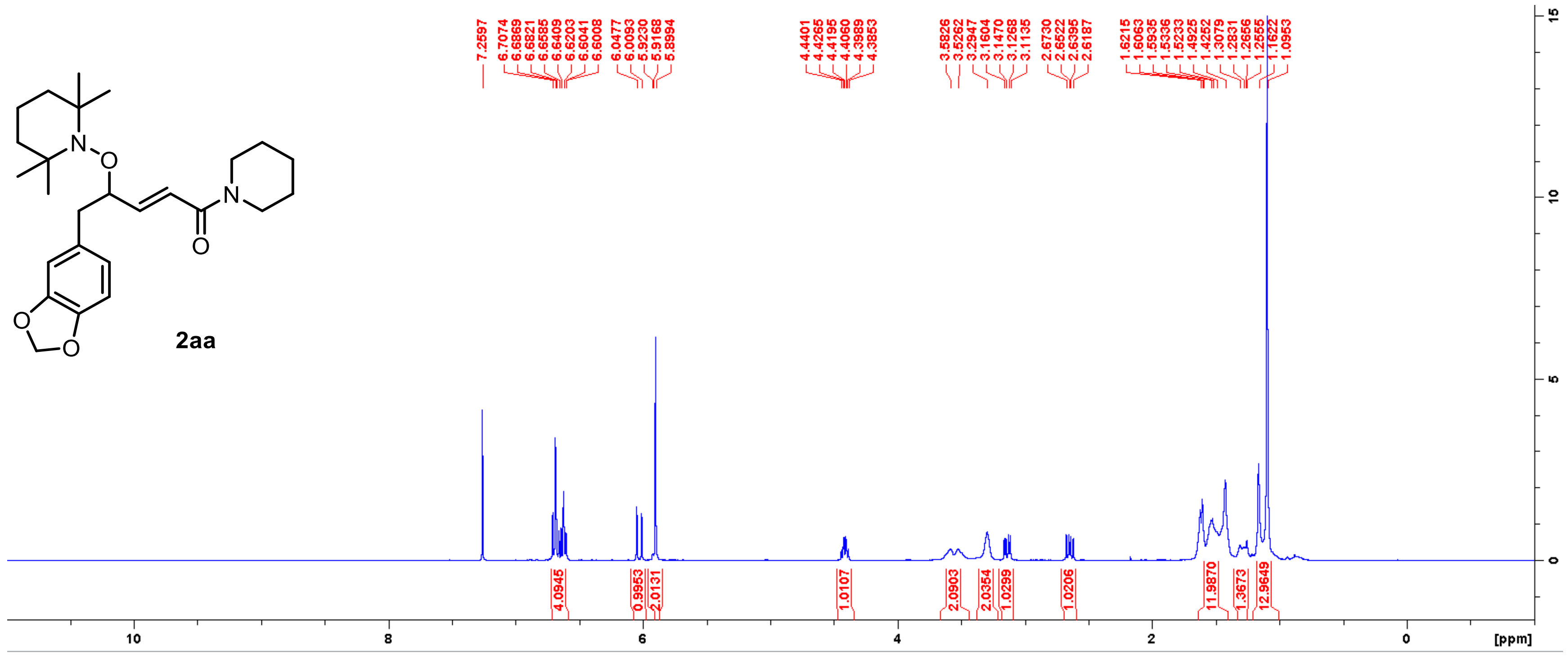

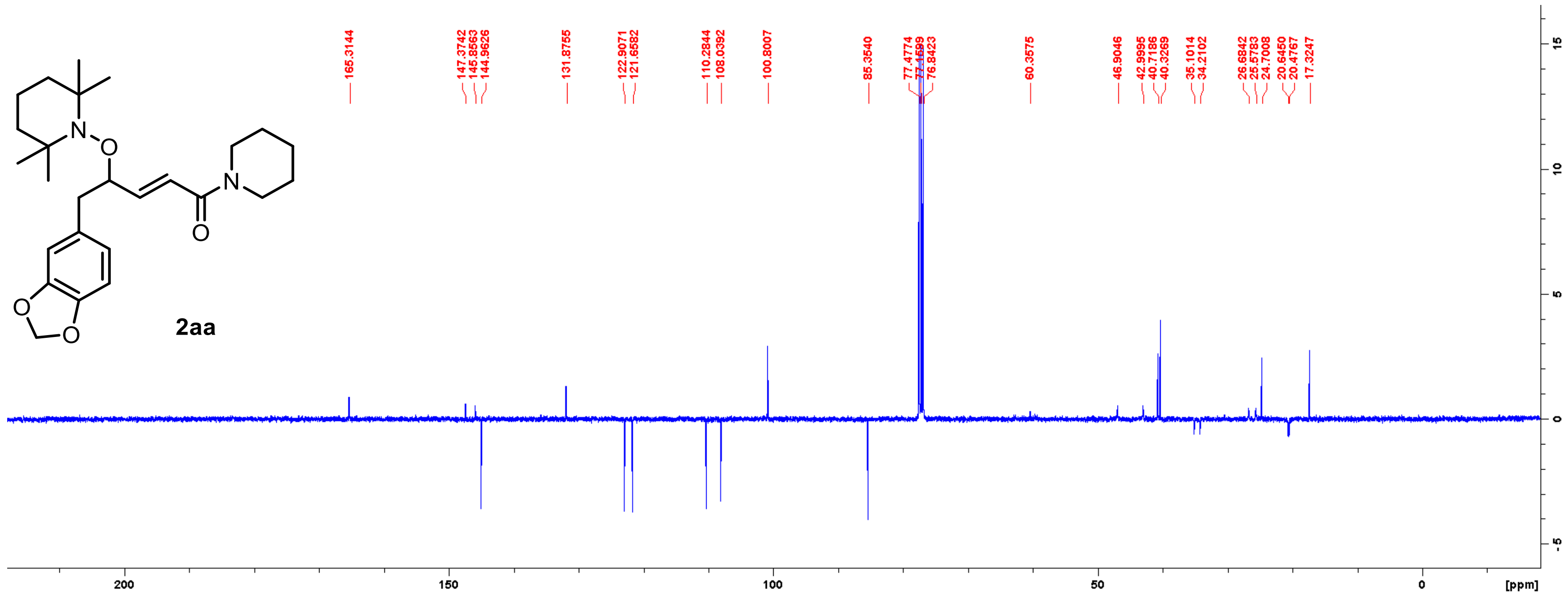

(*E*)-*N*-(3-(10,11-dihydro-5*H*-dibenzo[b,f]azepin-5-yl)propyl)-*N*-methyl-4-((2,2,6,6-tetramethylpiperidin-1-yl)oxy)pent-2-enamide (2ab, rotamers present)

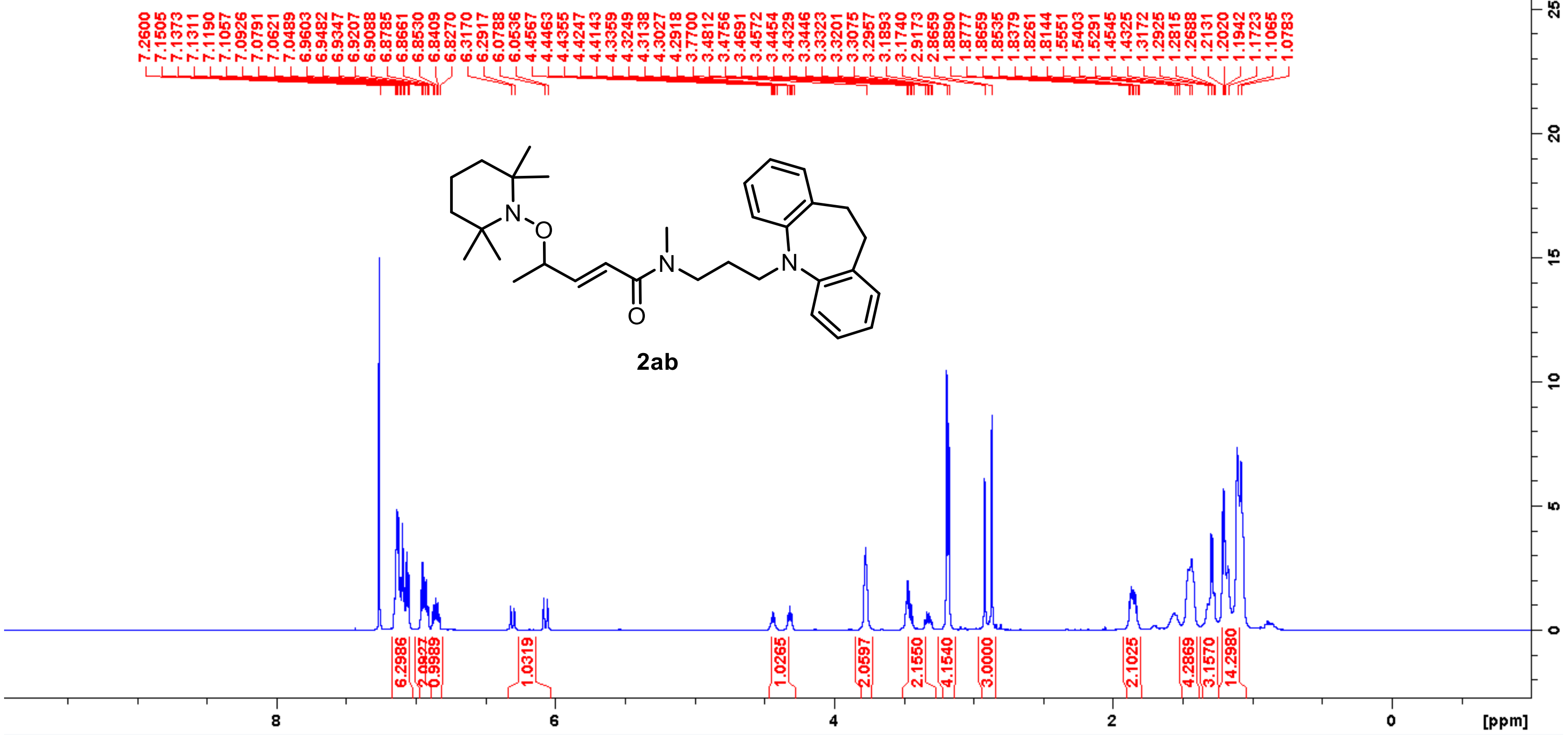

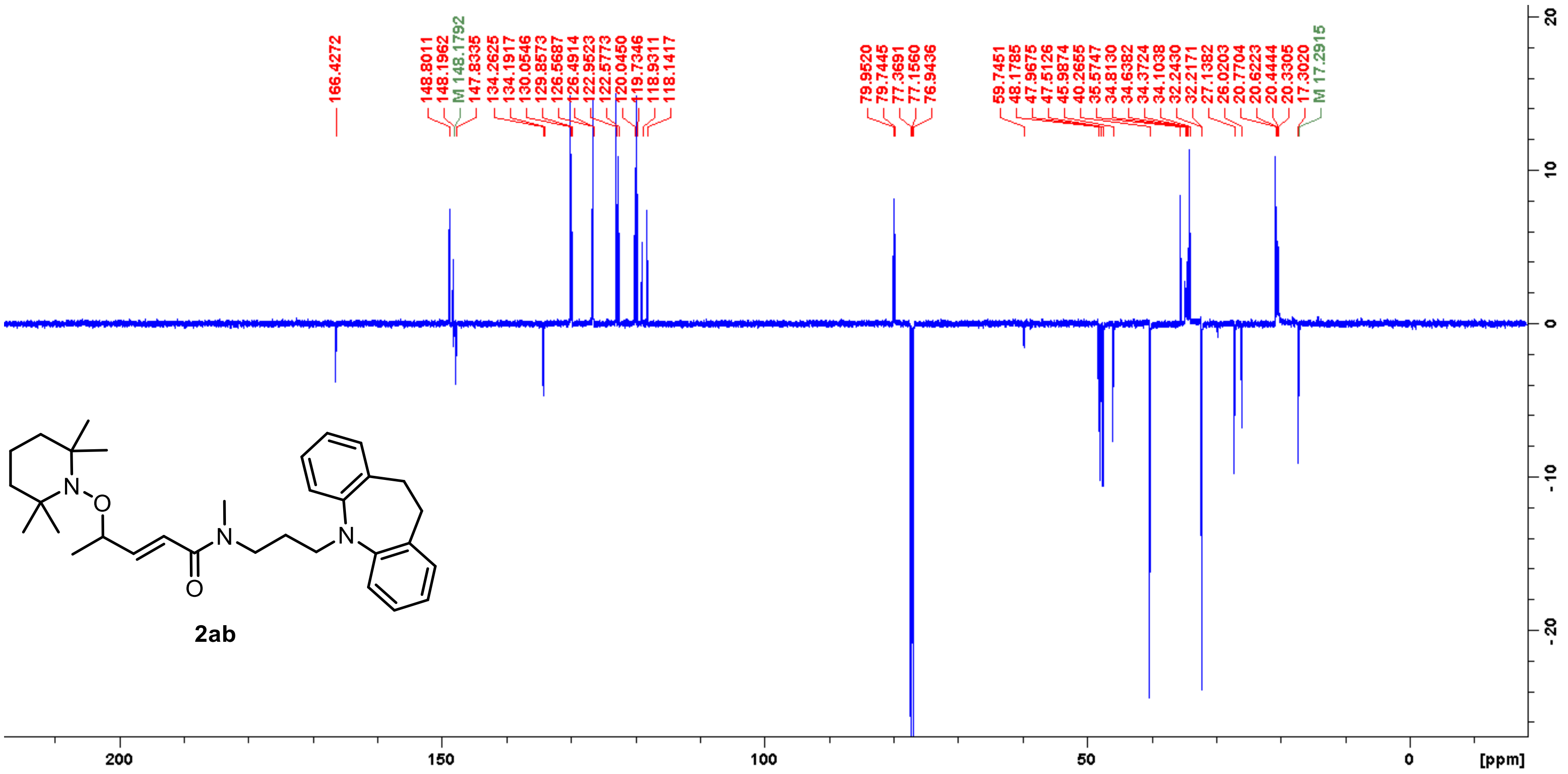

4.3. Mechanistic investigations

1-Isopropyl-2,2-dimethylpyrrolidine (VI)

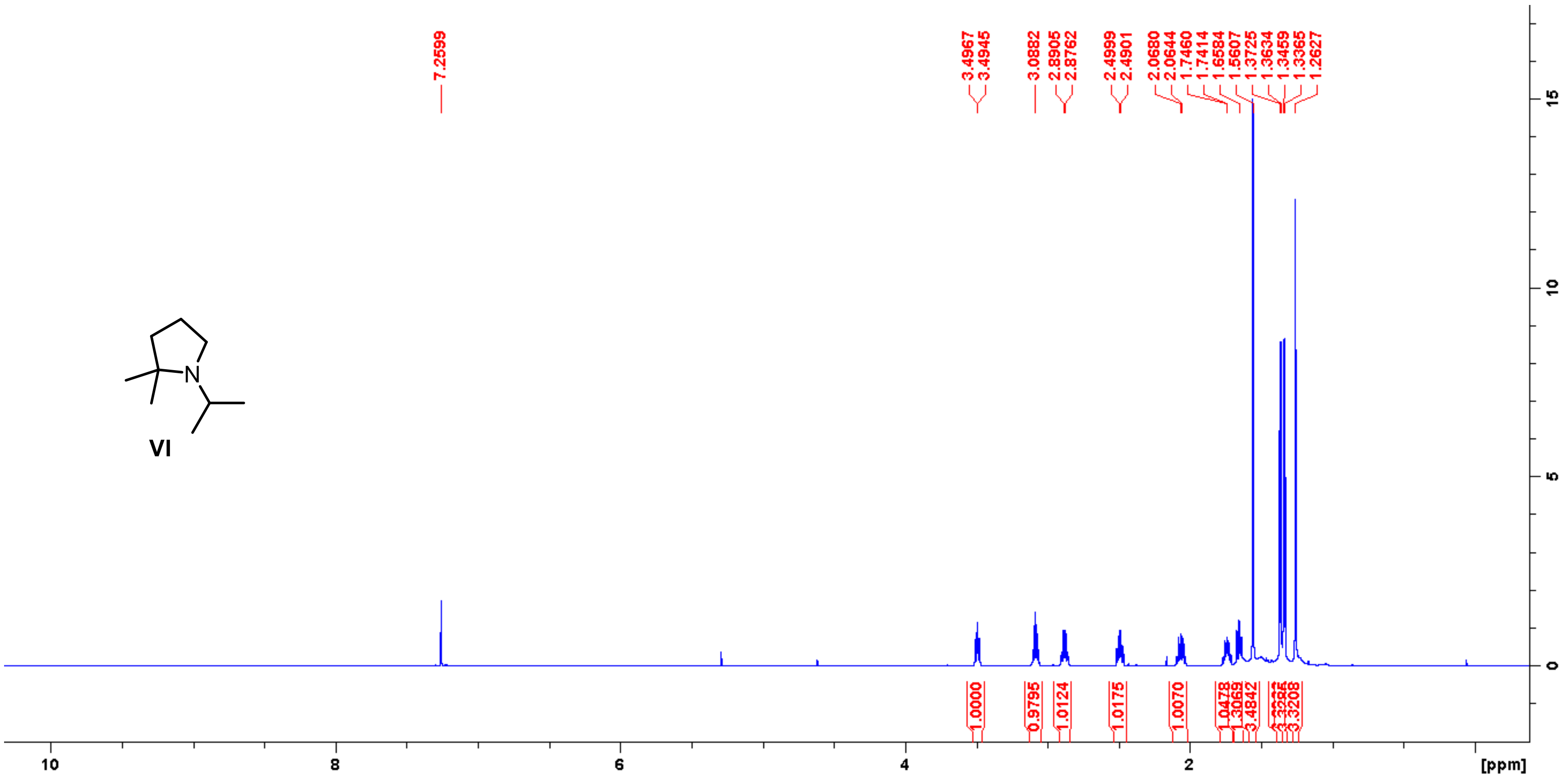

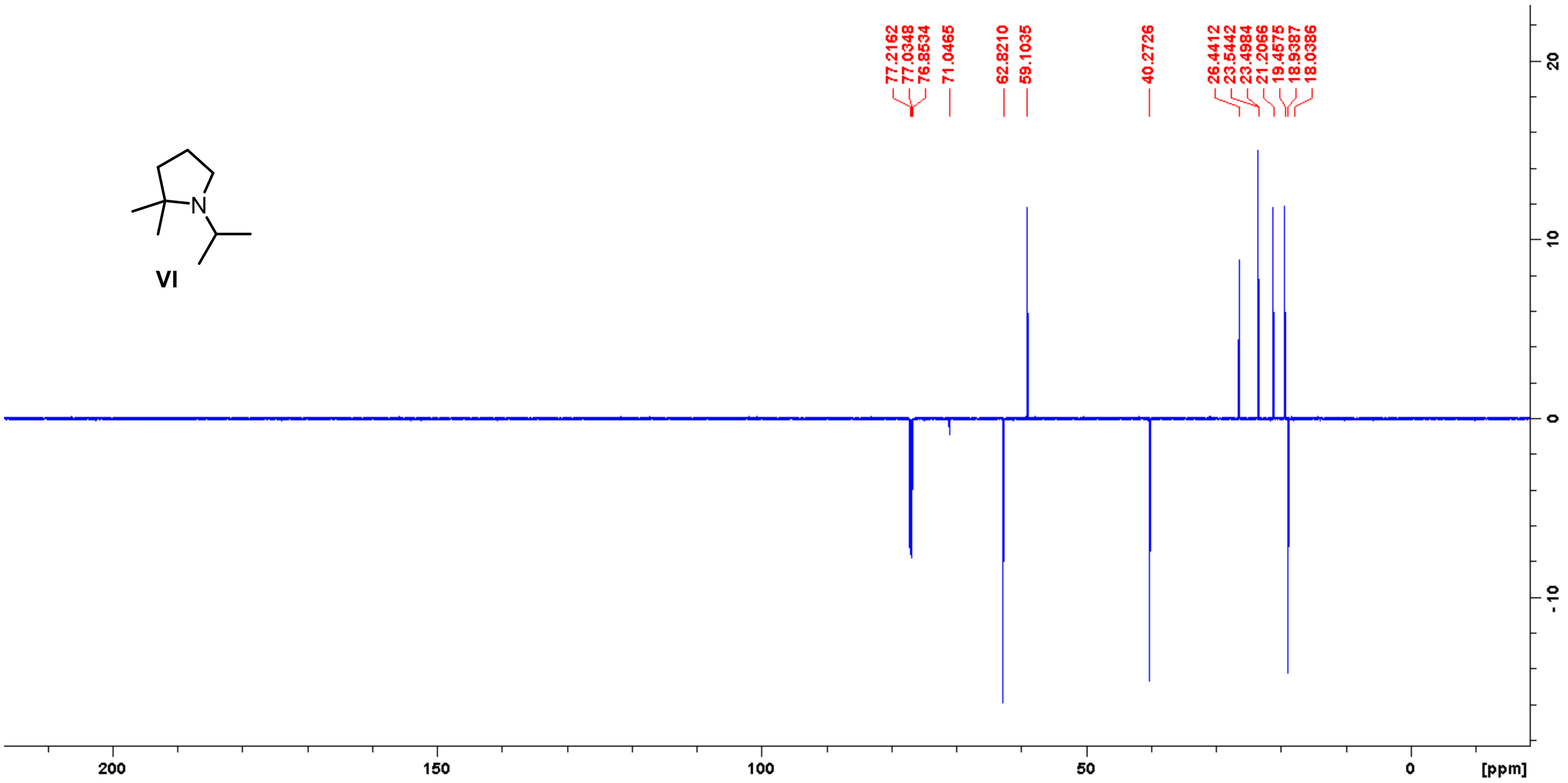

4.3 Post-functionalizations

(*E*)-1-(Pyrrolidin-1-yl)-pent-2-ene-1,4-dione (3a)

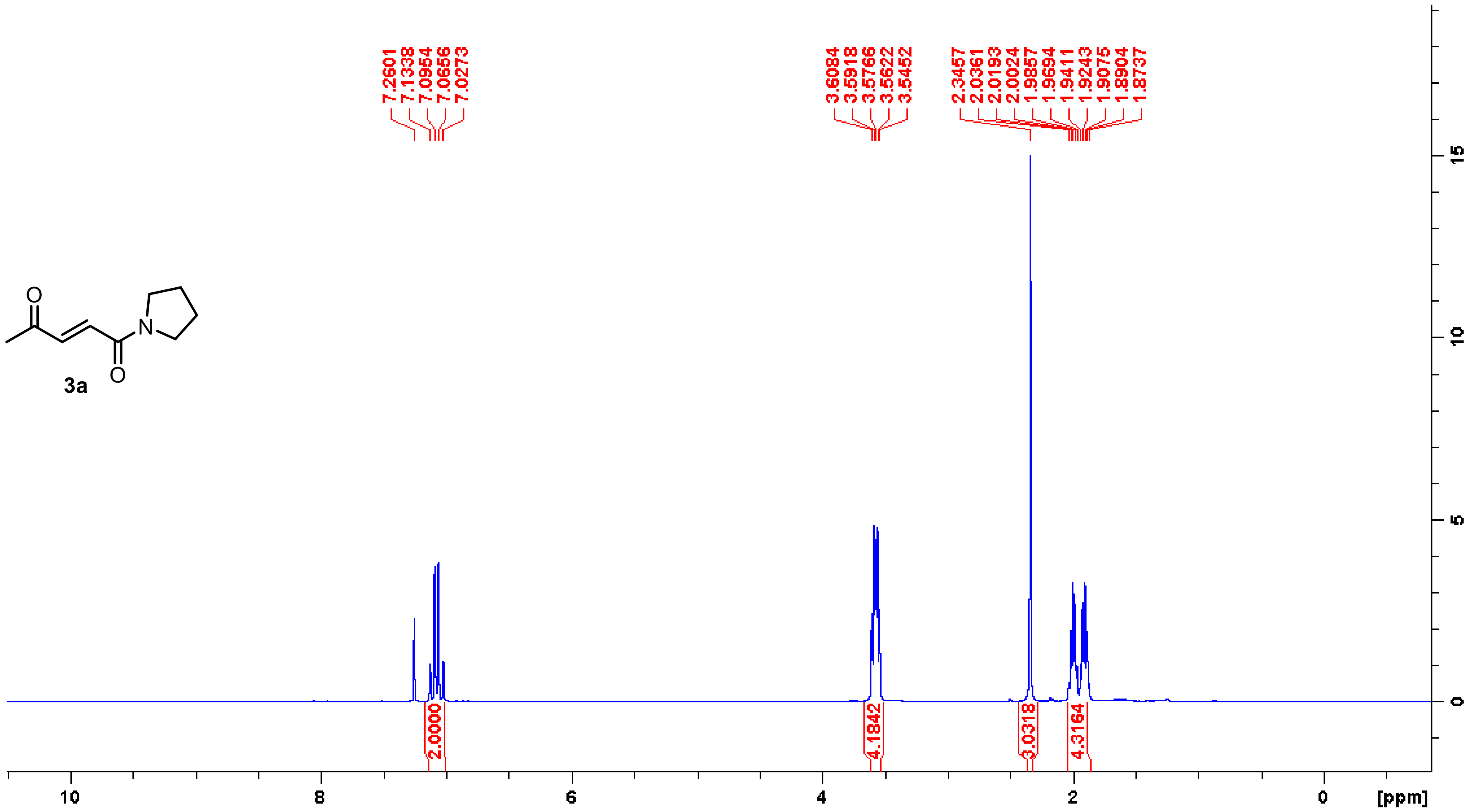

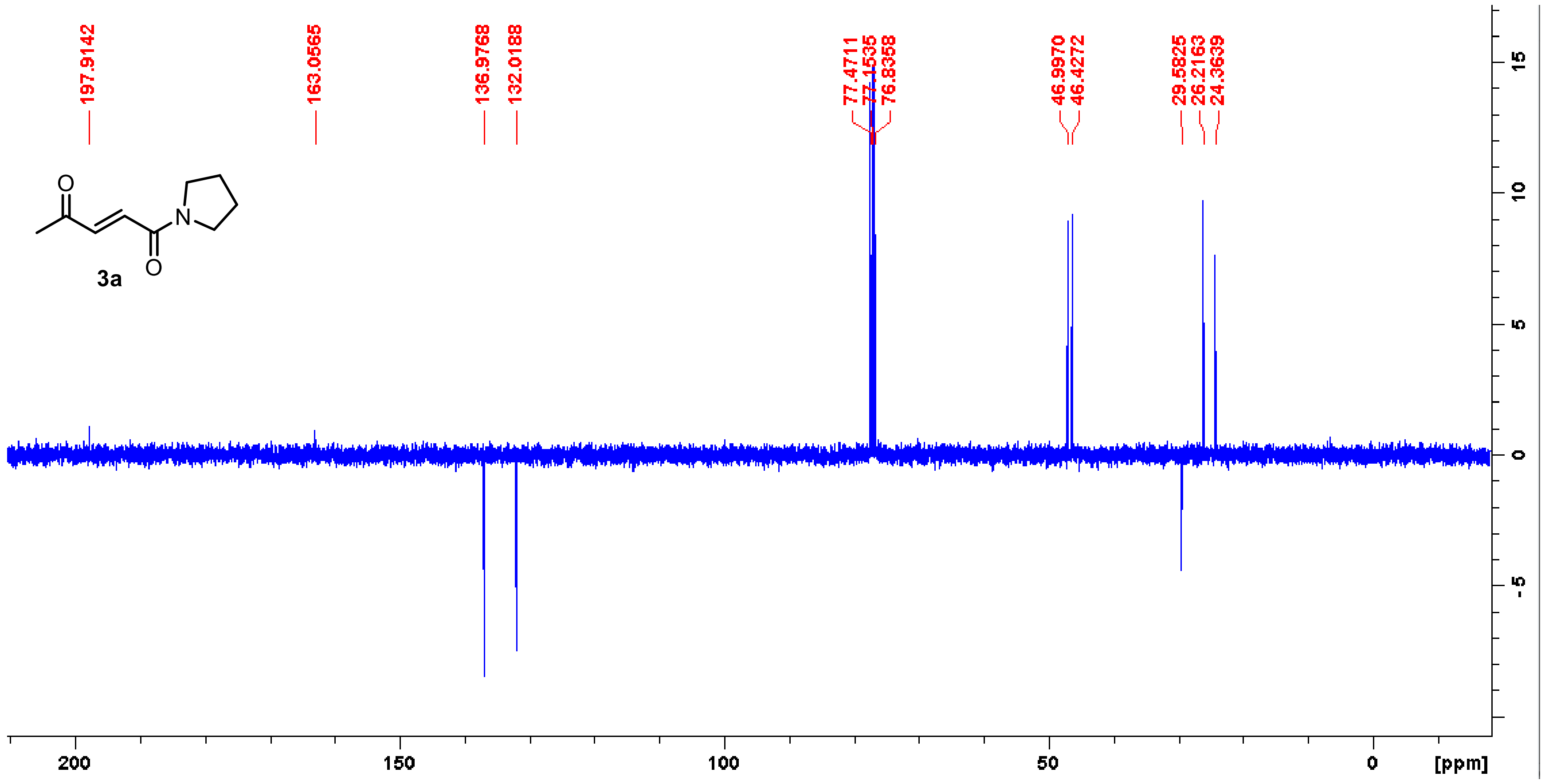

(*Z*)-1-(Pyrrolidin-1-yl)pent-2-ene-1,4-dione (3b)

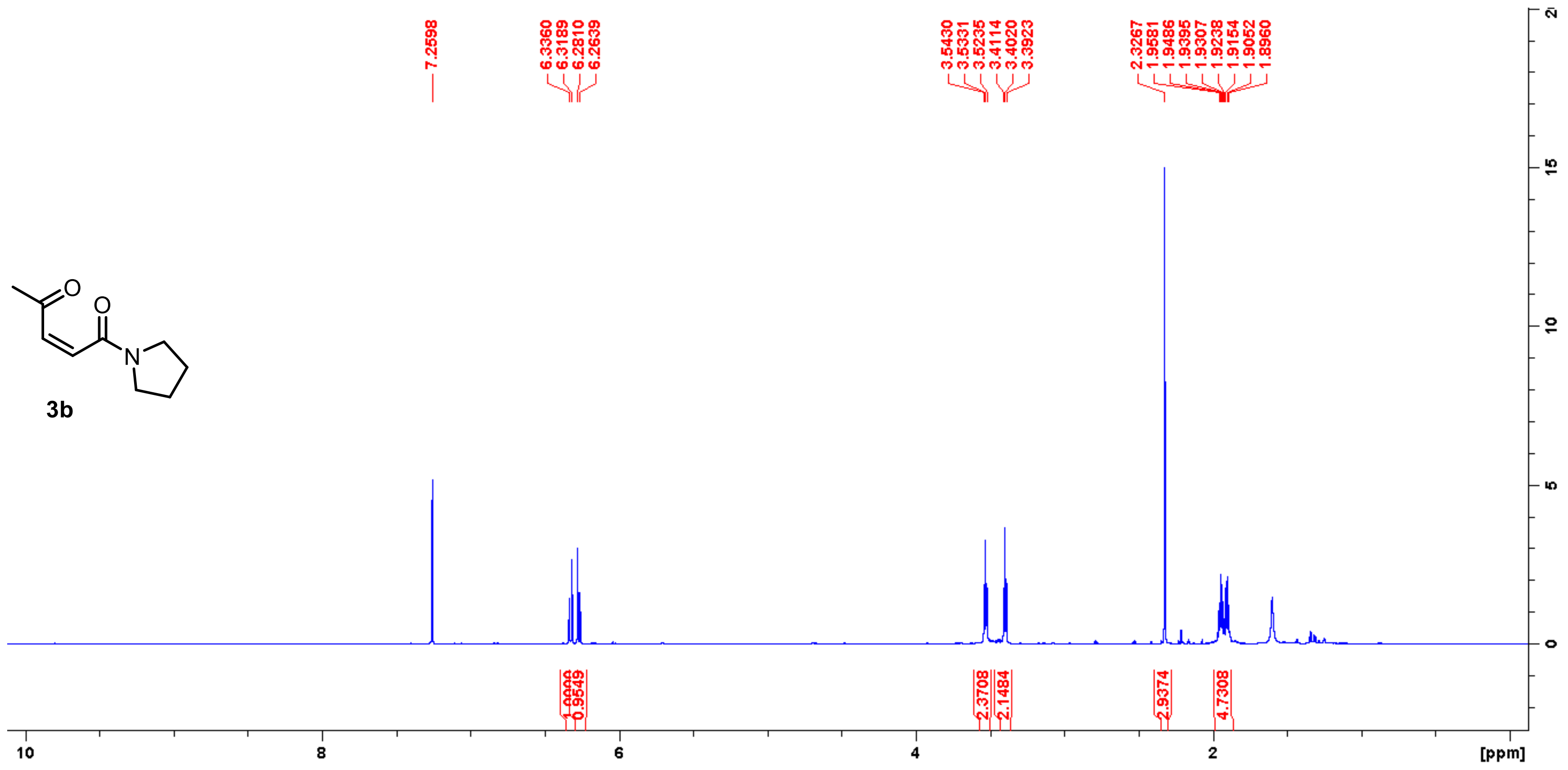

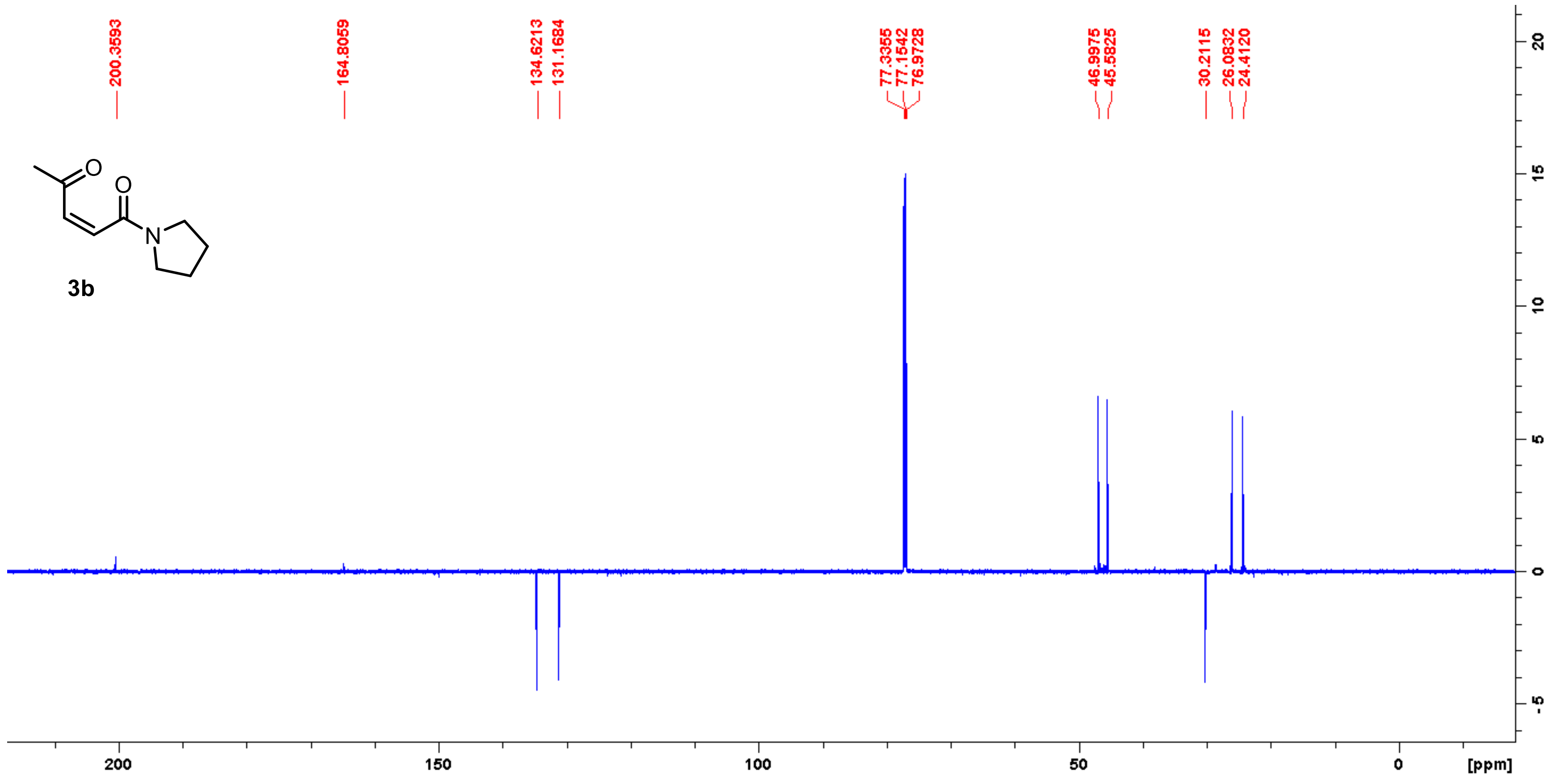

(*E*)-3-((2R)-2-Methyl-5-(prop-1-en-2-yl)-cyclopentyl)-1-(pyrrolidin-1-yl)-prop-2-en-1-one (4a; diastereomers present)

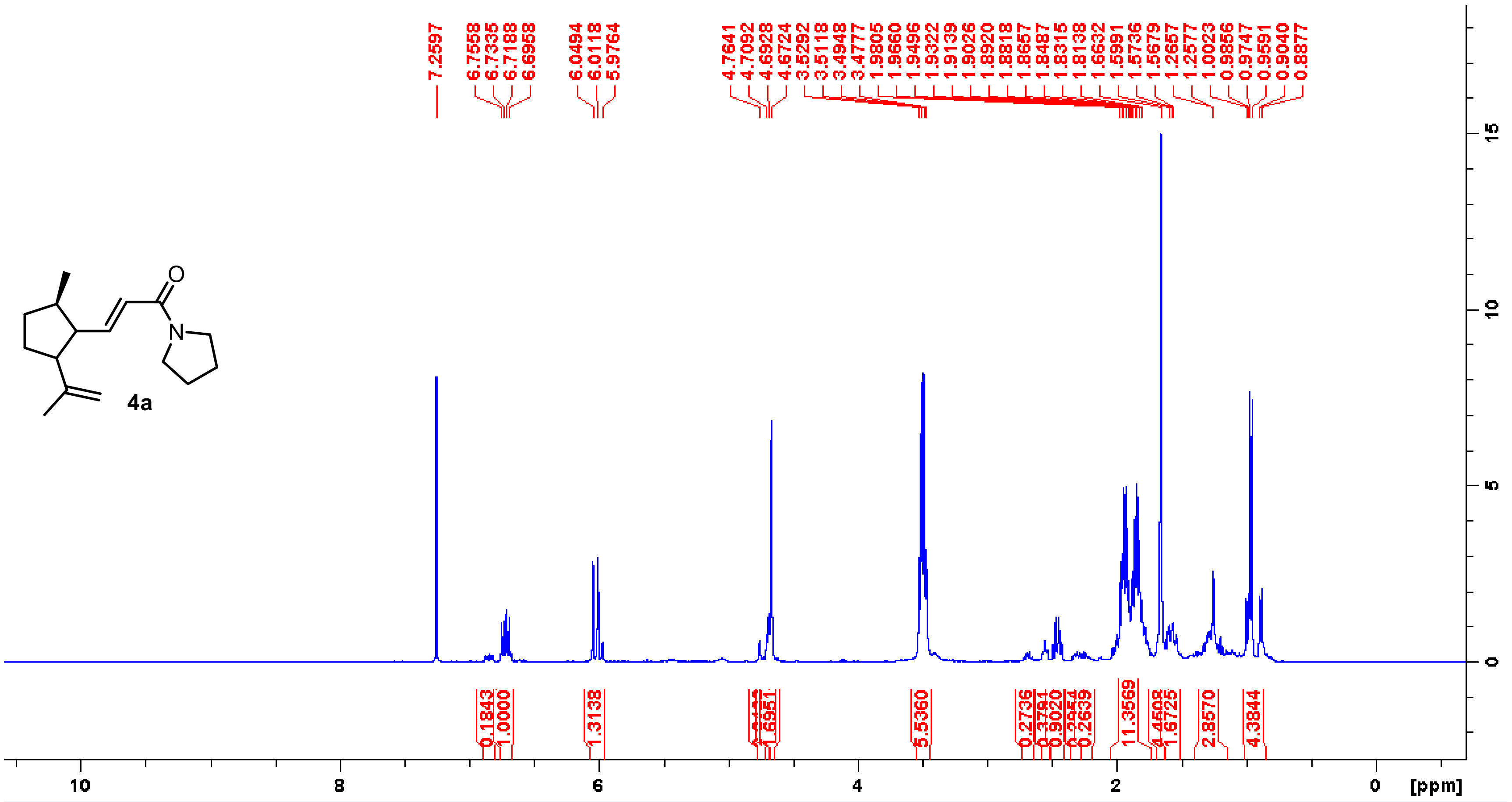

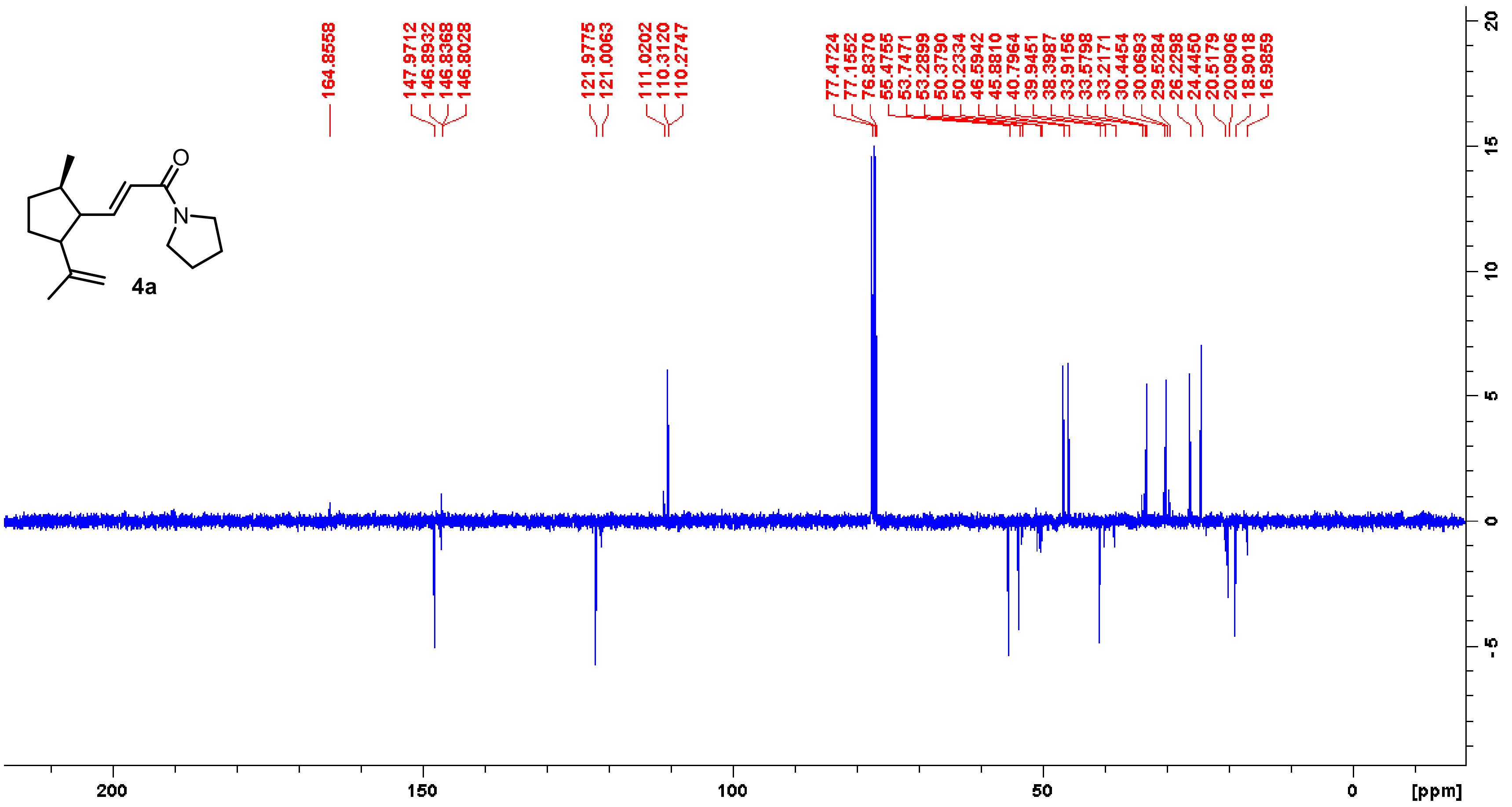

(*E*)-1-Butyl-3-(prop-1-en-1-yl)-4-(prop-1-en-2-yl)-pyrrolidin-2-one (4b; diastereomers present)

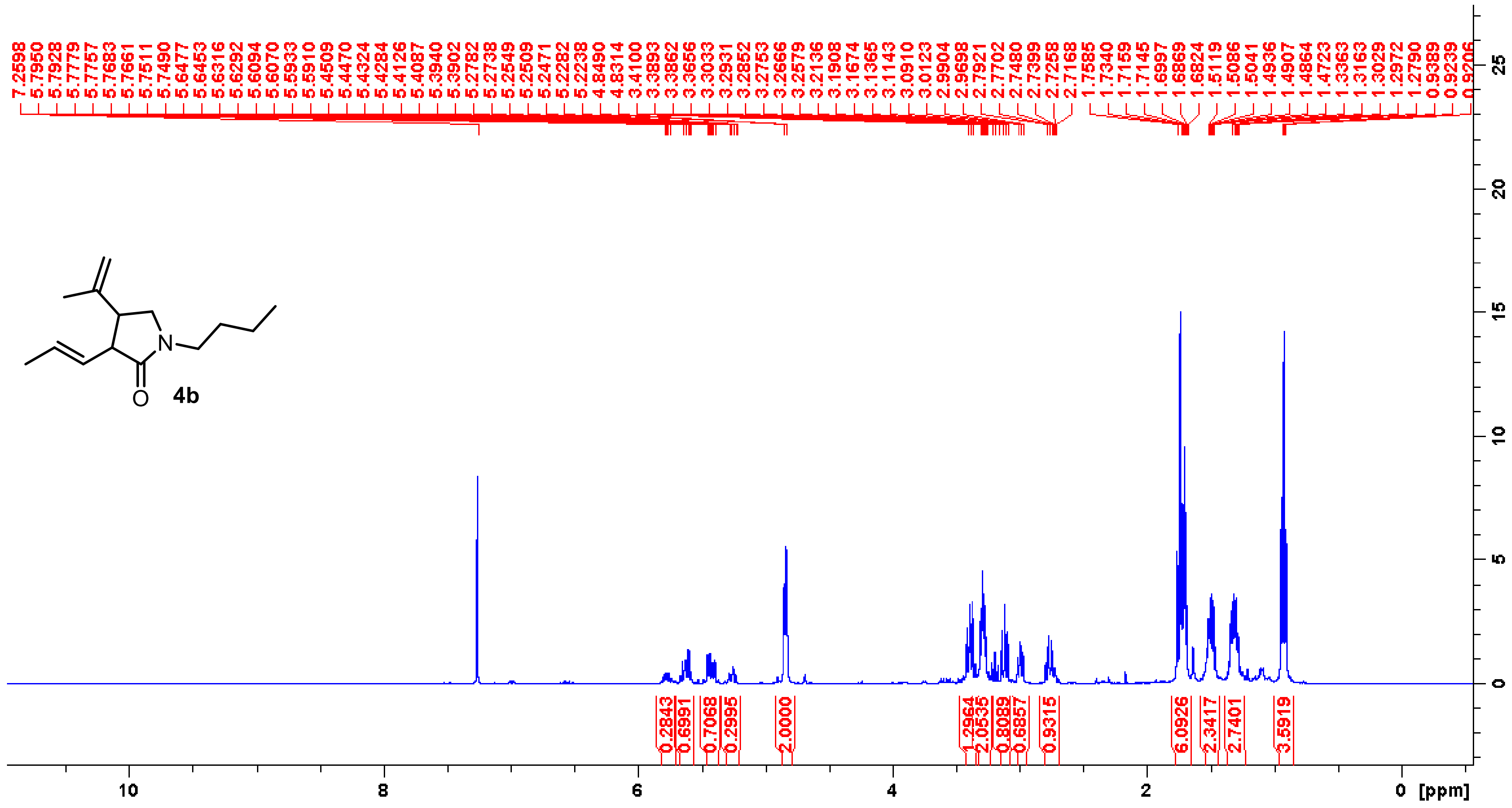

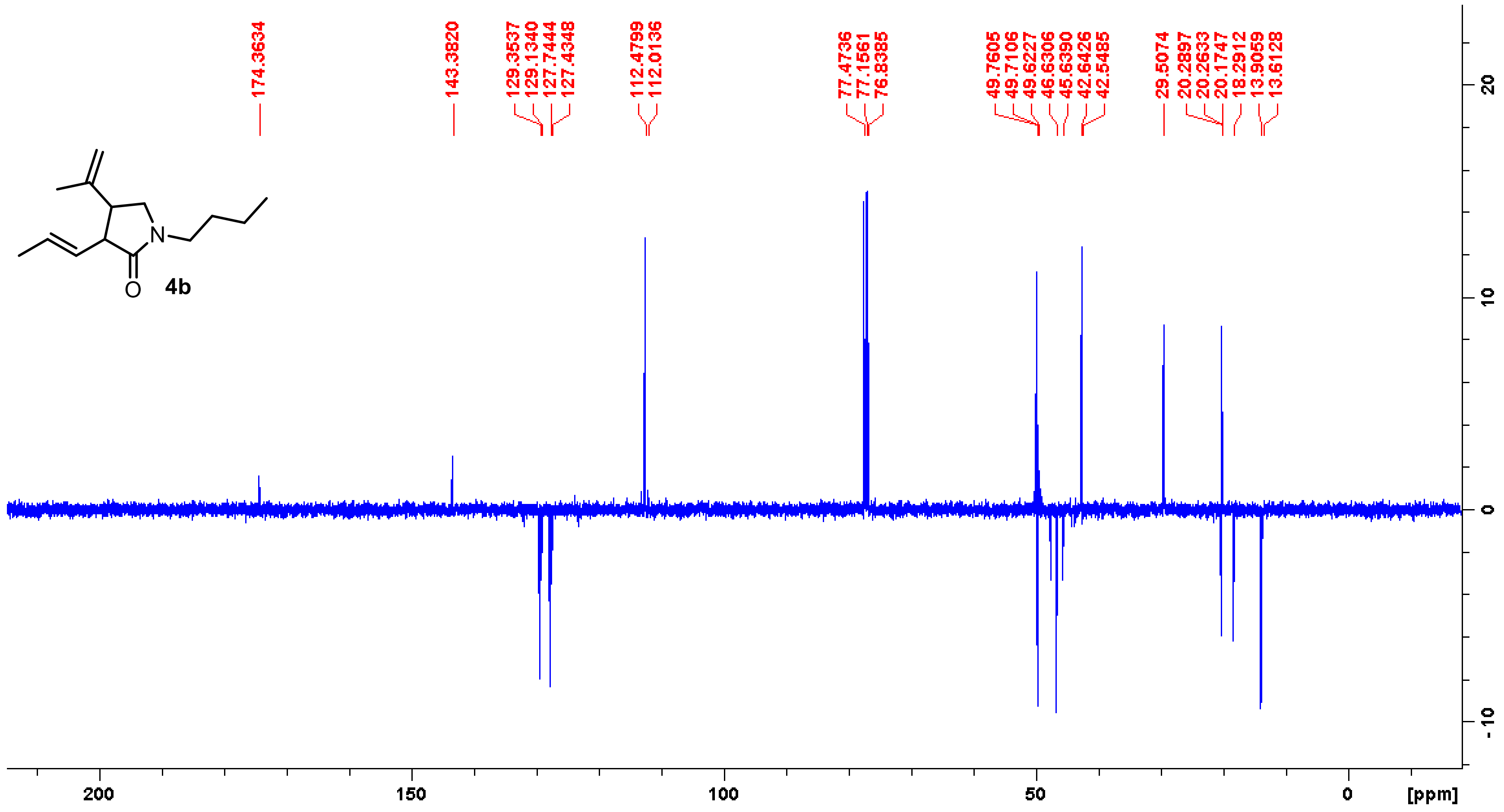

1-Butyl-3-((*R*,*E*)-3,7-dimethylocta-1,6-dien-1-yl)-4-(prop-1-en-2-yl)-pyrrolidin-2-one (4c; diastereomers present)

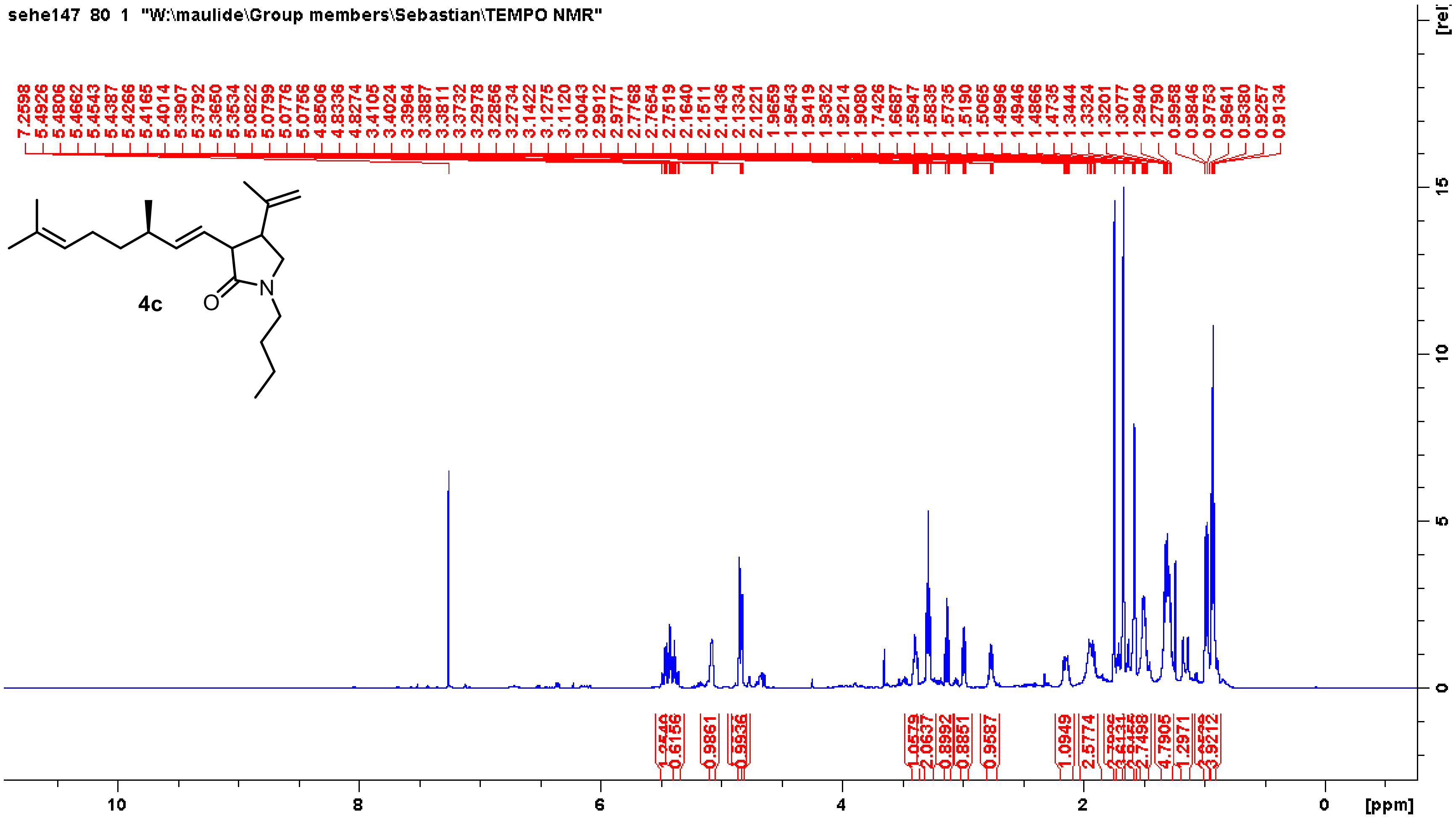

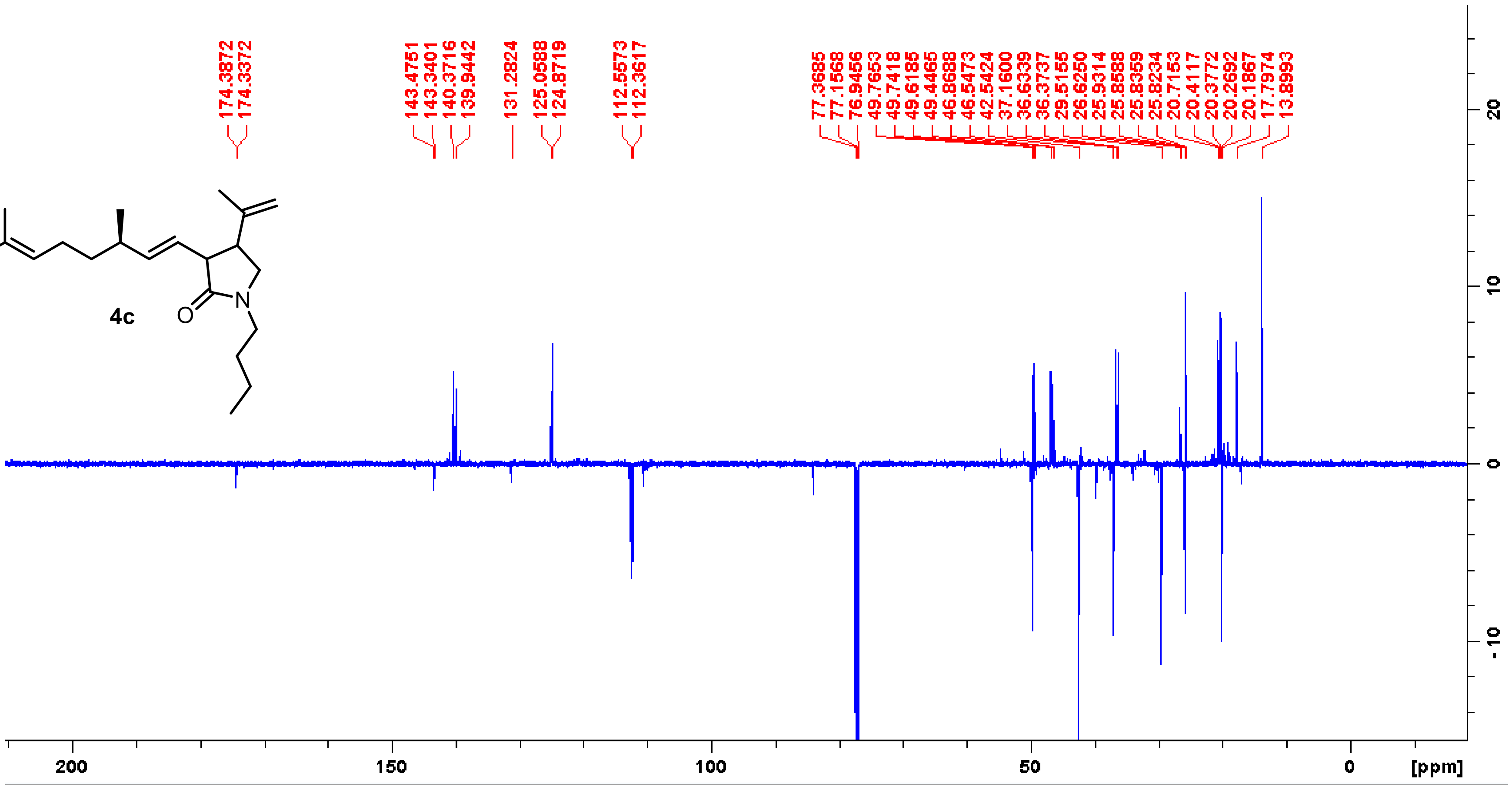

(*E*)-1-Methyl-3-(prop-1-en-1-yl)-4-(((2,2,6,6-tetramethylpiperidin-1-yl)-oxy)-methyl)-pyrrolidin-2-one (4d; diastereomers present)

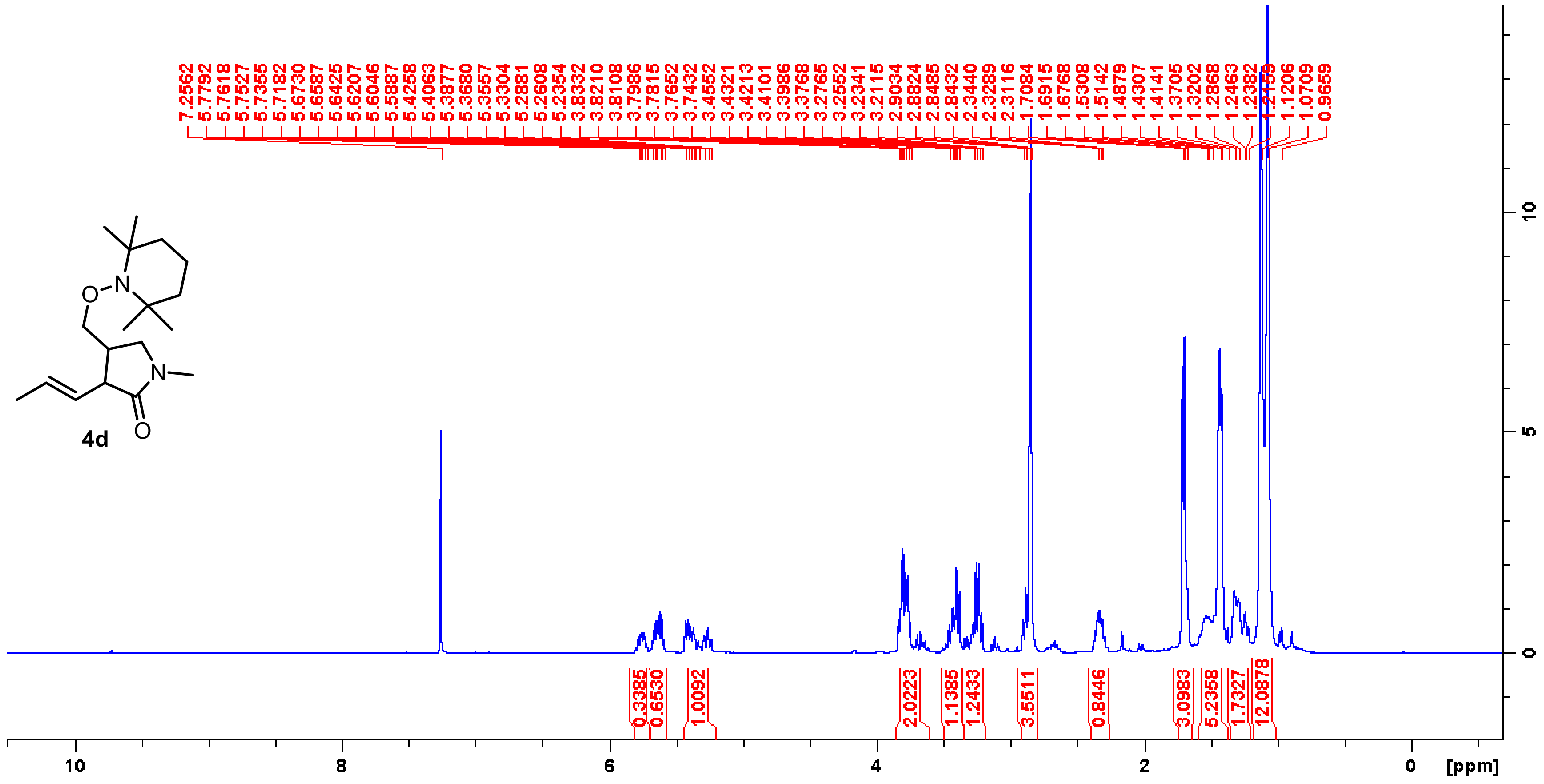

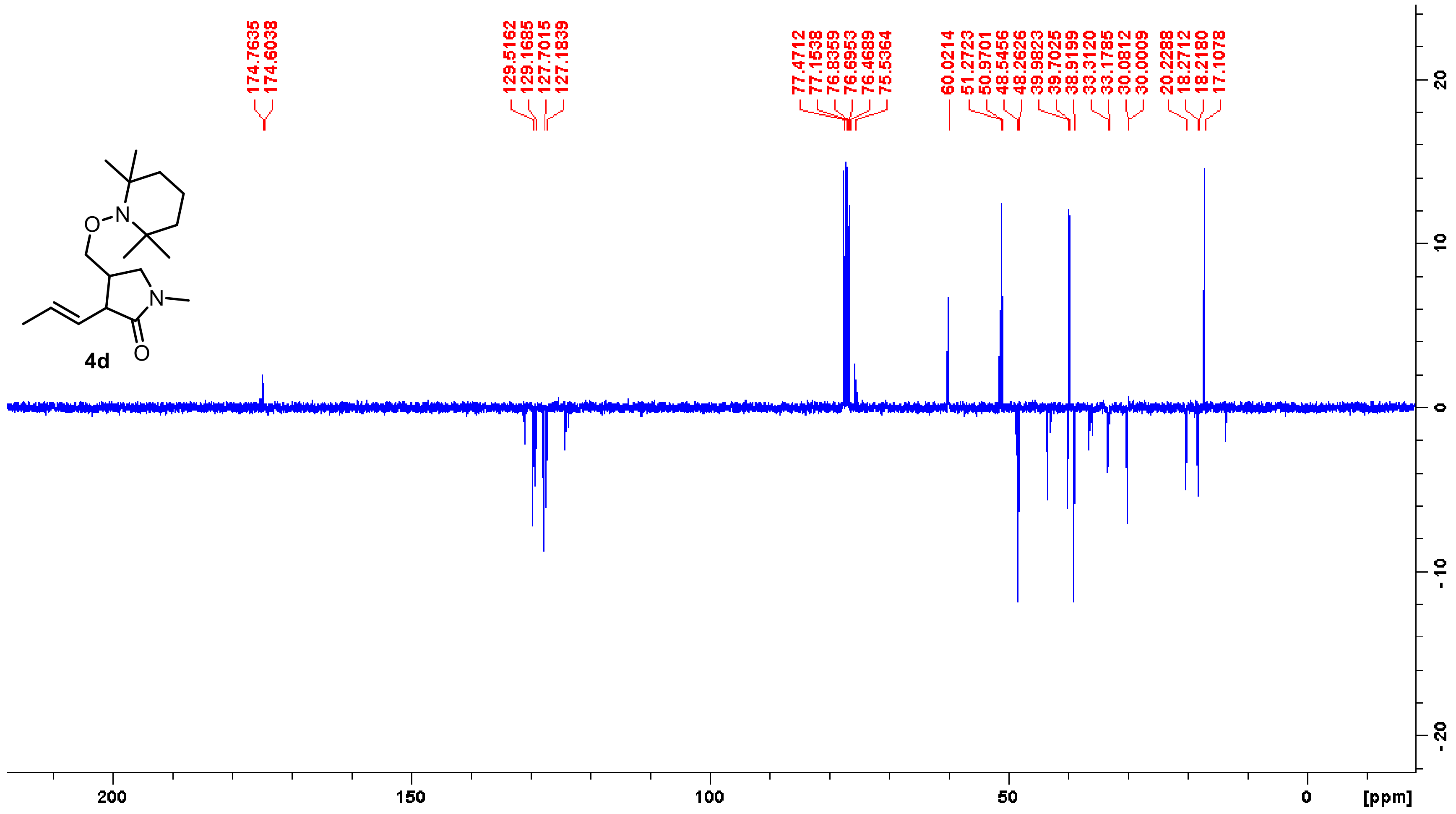

(*E*)-*N*,*N*-Dimethyl-4-oxododec-2-enamide (3c)

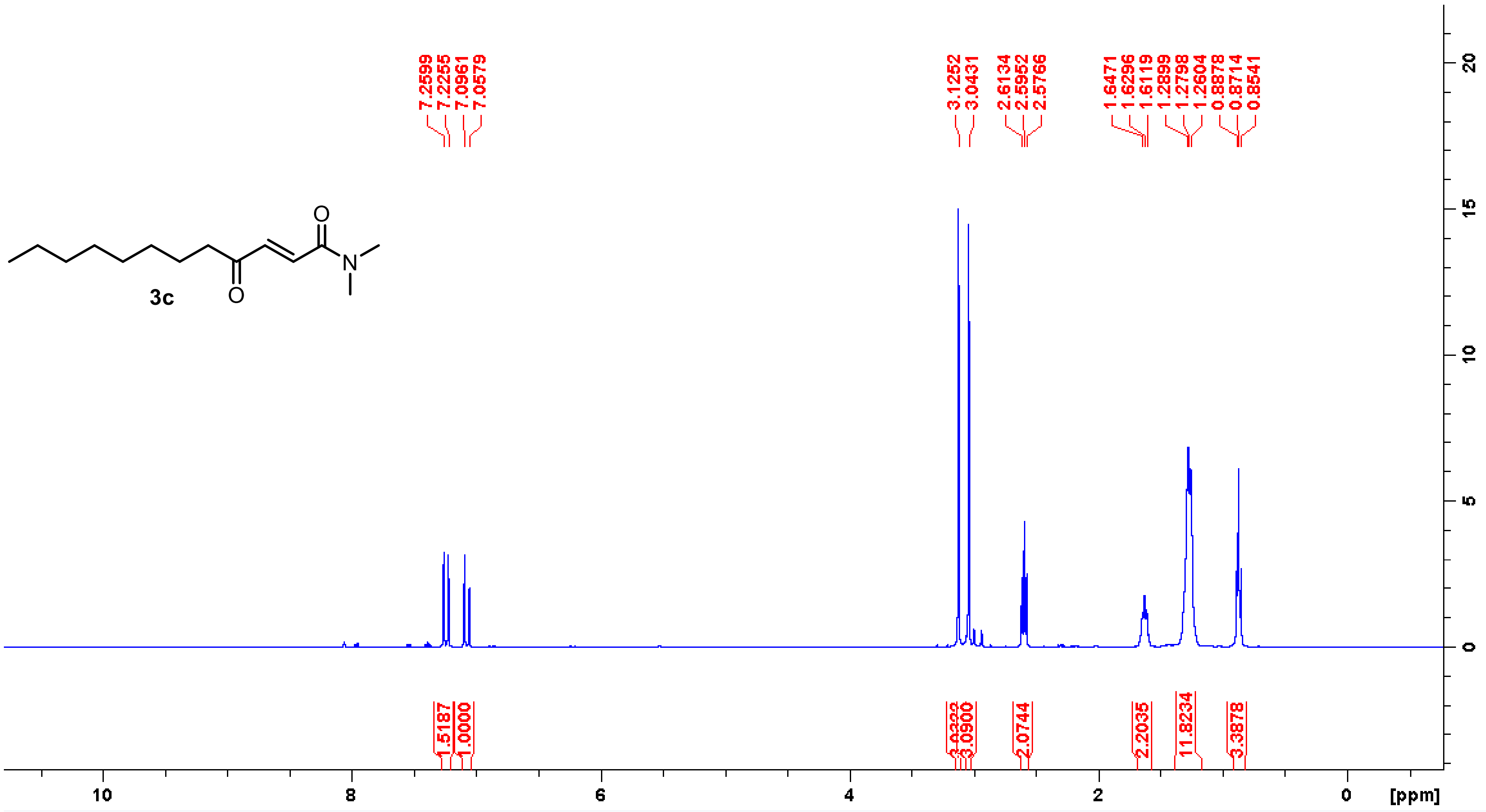

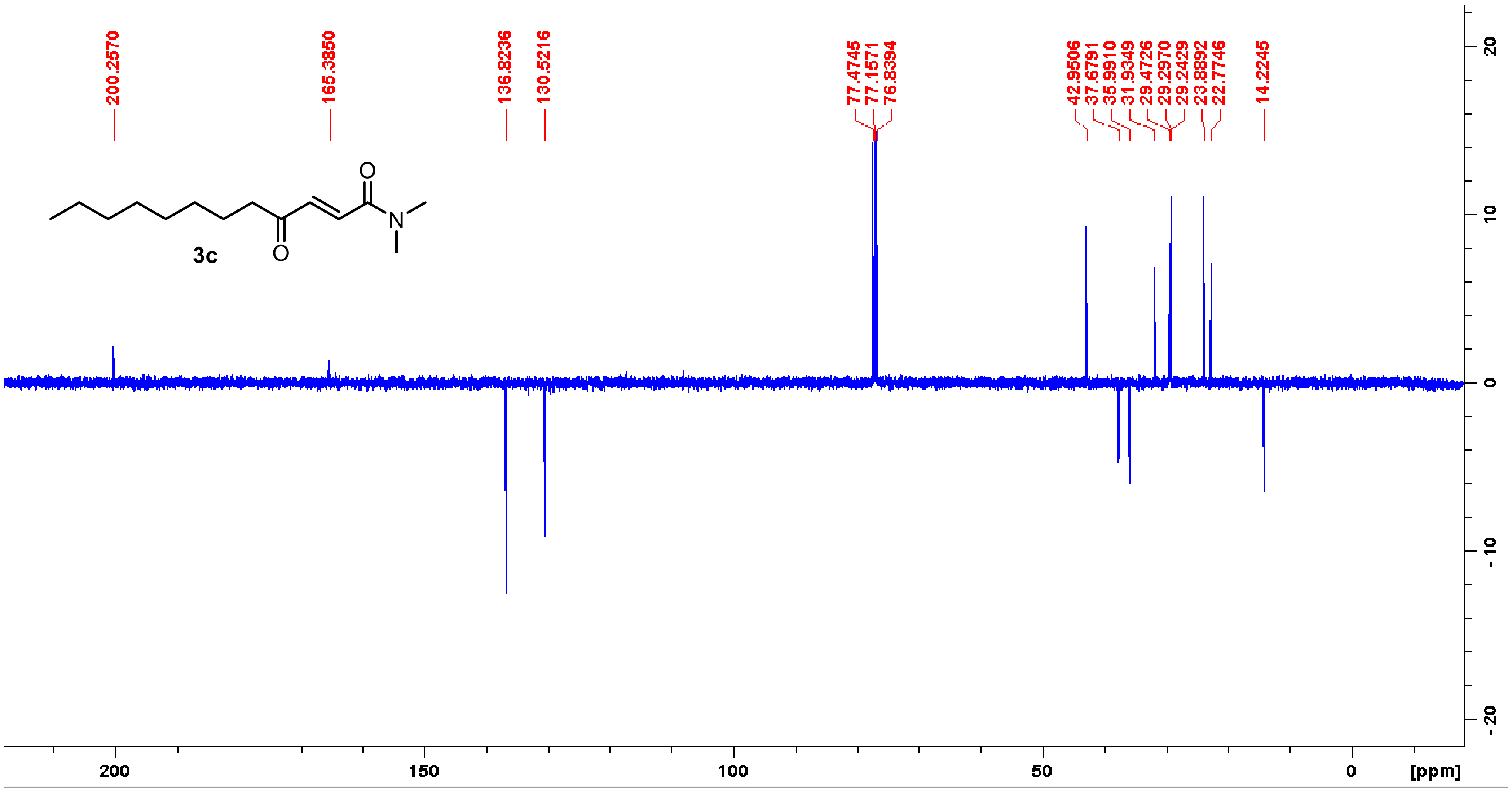

(*E*)-1-(Pyrrolidin-1-yl)dodec-2-ene-1,4-dione (3d)

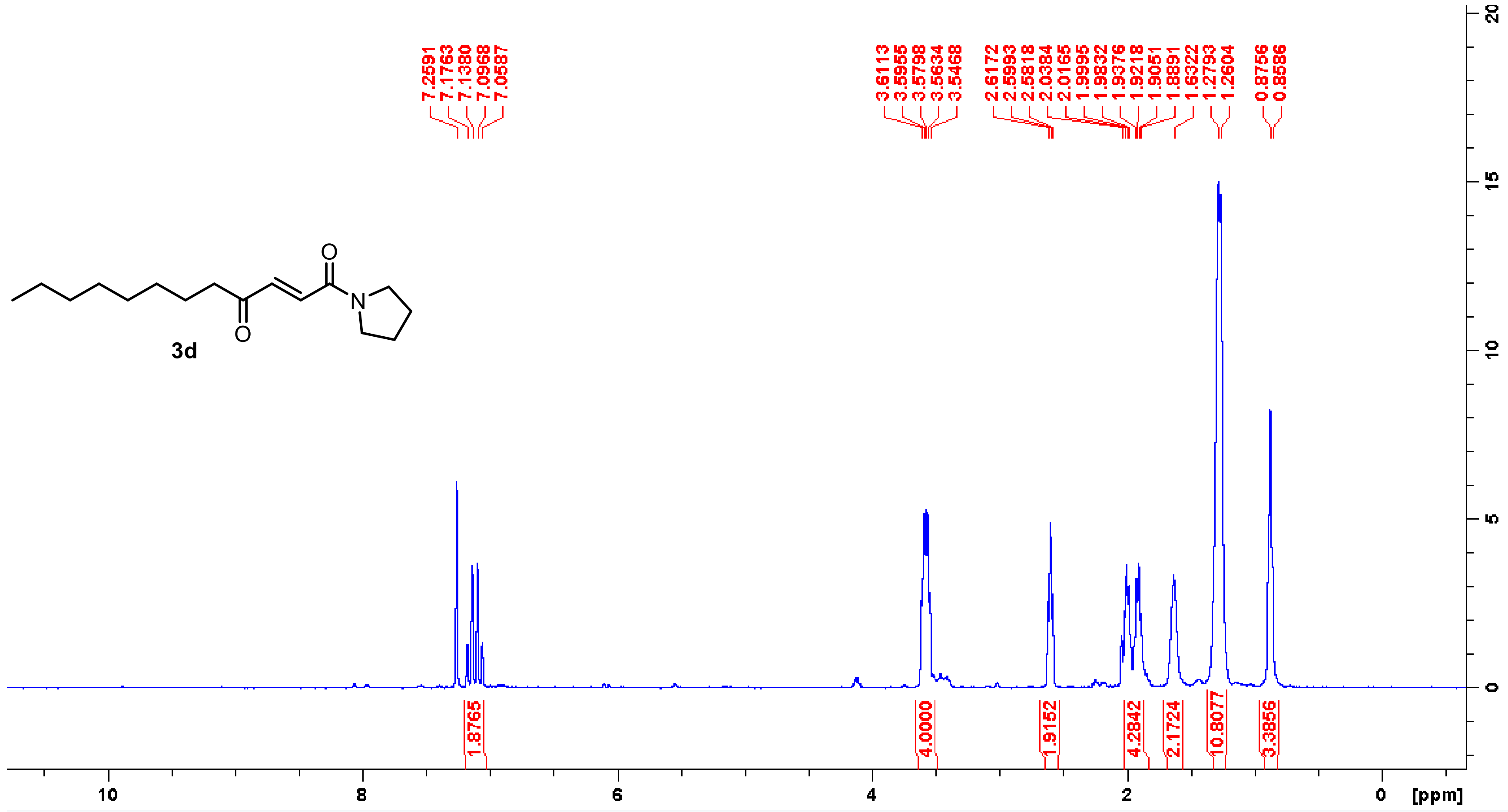

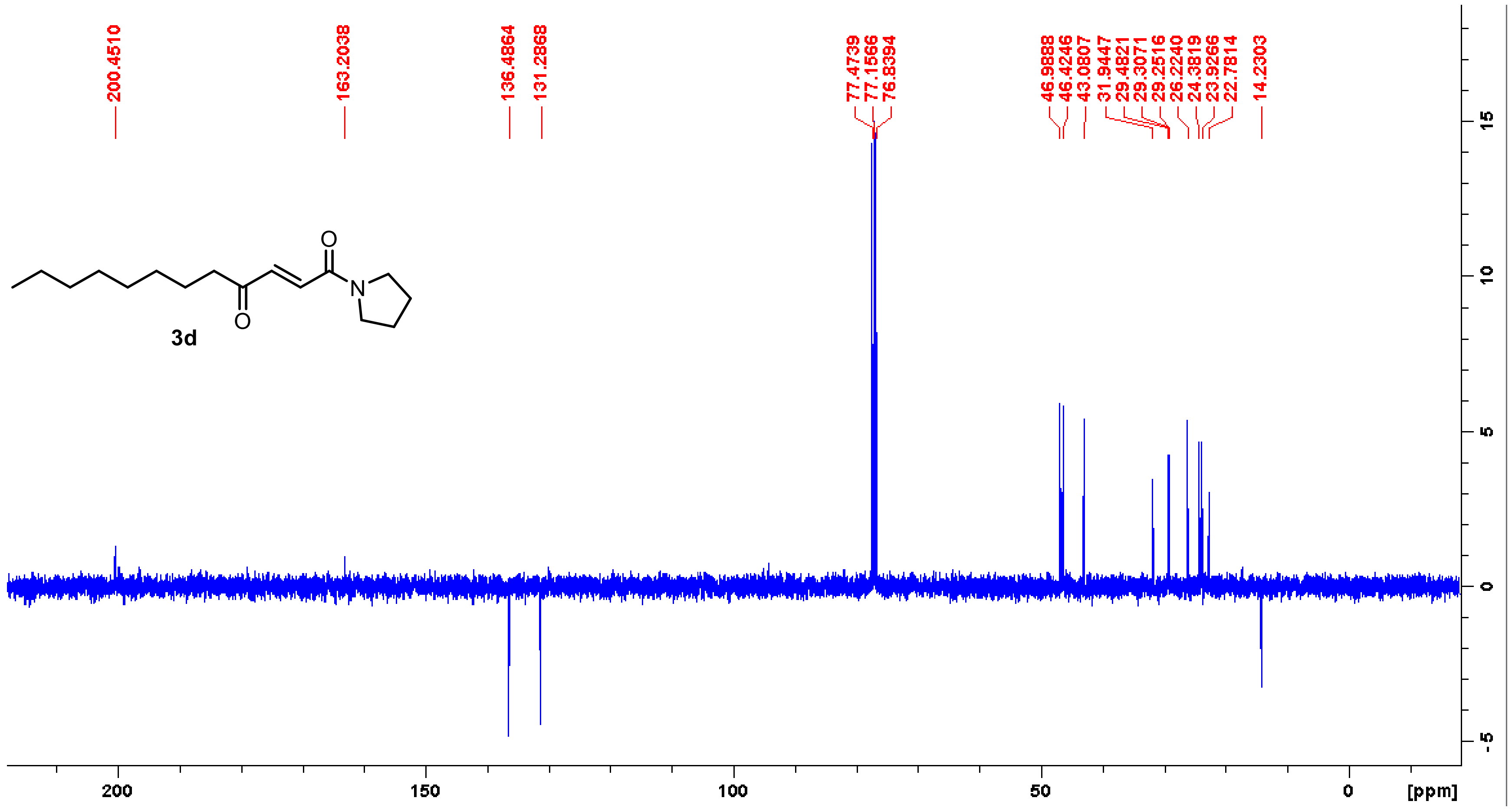

(*E*)-1-(Pyrrolidin-1-yl)penta-2,4-dien-1-one (12)

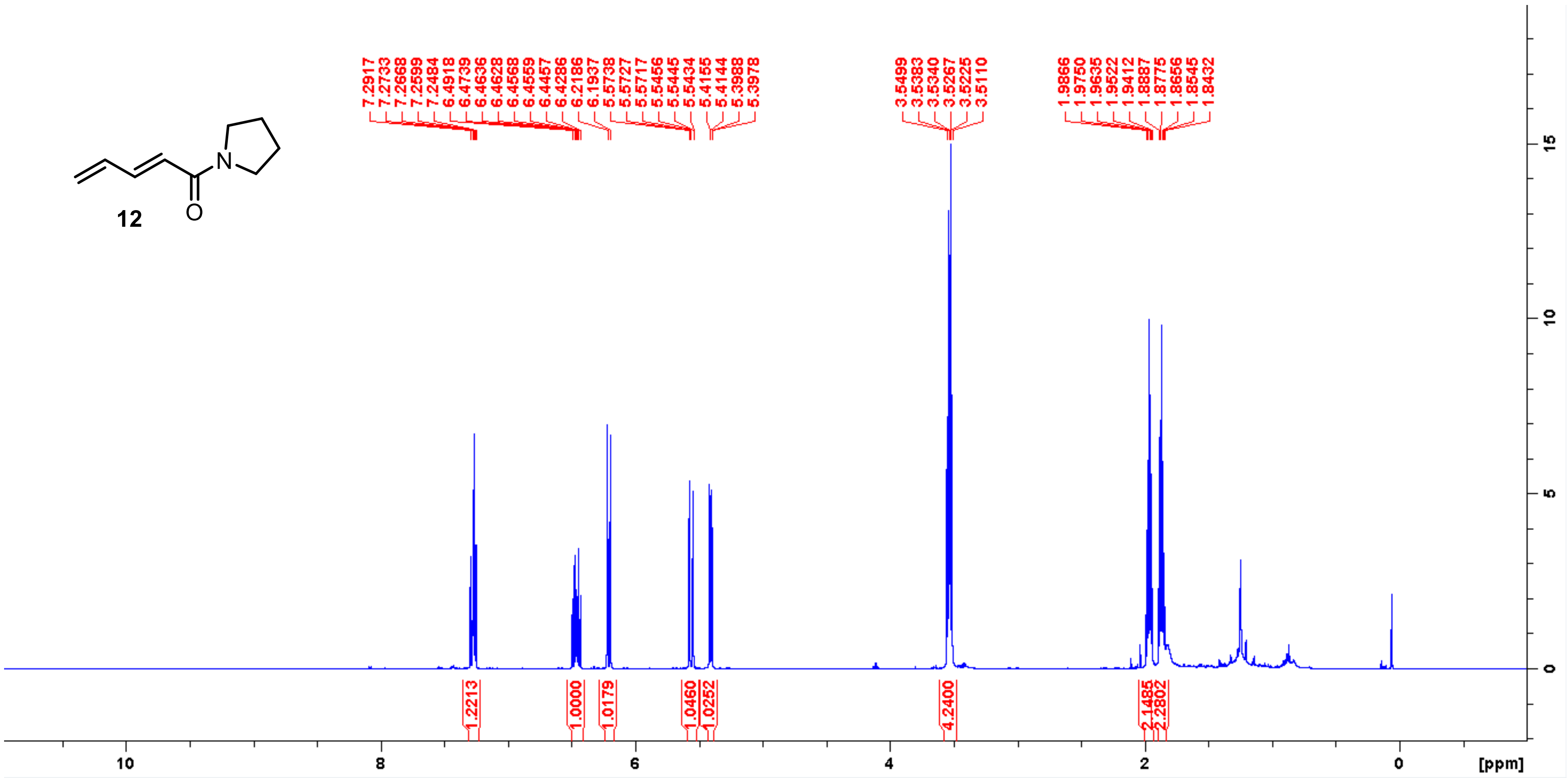

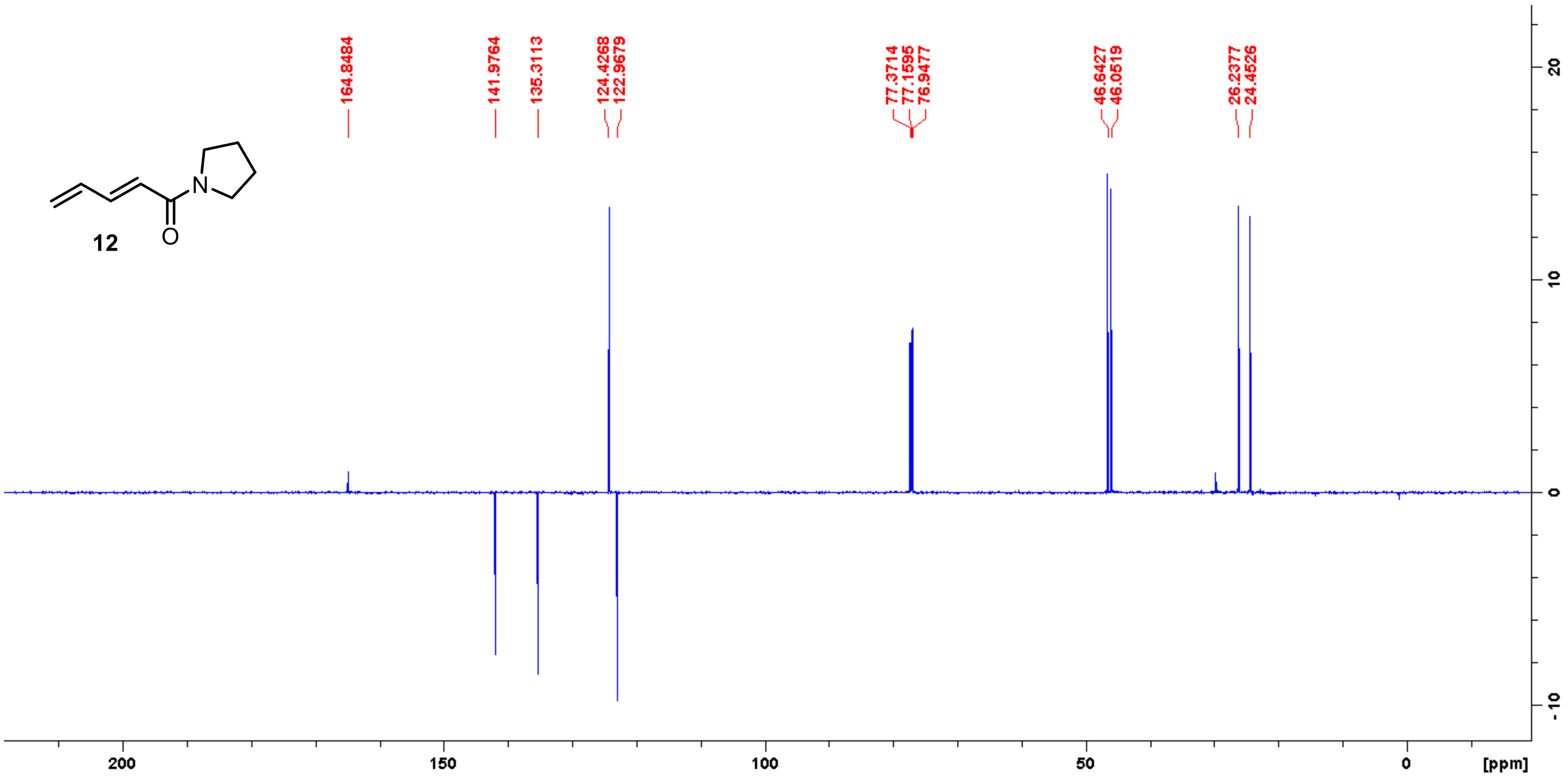

Supplement: Supplementary file 1 — Supporting Information [file ANIE-60-19123-s001.pdf]
